# Enantioselective Oxidative Rearrangements with Chiral Hypervalent Iodine Reagents

**DOI:** 10.1002/chem.201504844

**Published:** 2016-01-21

**Authors:** Michael Brown, Ravi Kumar, Julia Rehbein, Thomas Wirth

**Affiliations:** ^1^ School of ChemistryCardiff UniversityPark Place, Main BuildingCardiffCF10 3ATUK; ^2^ Organische ChemieUniversität HamburgMartin-Luther-King-Platz 620146HamburgGermany

**Keywords:** alkenes, arylation, hypervalent iodine, oxidation, rearrangement

## Abstract

A stereoselective hypervalent iodine‐promoted oxidative rearrangement of 1,1‐disubstituted alkenes has been developed. This practically simple protocol provides access to enantioenriched α‐arylated ketones without the use of transition metals from readily accessible alkenes.

## Introduction

**Scheme 1:**
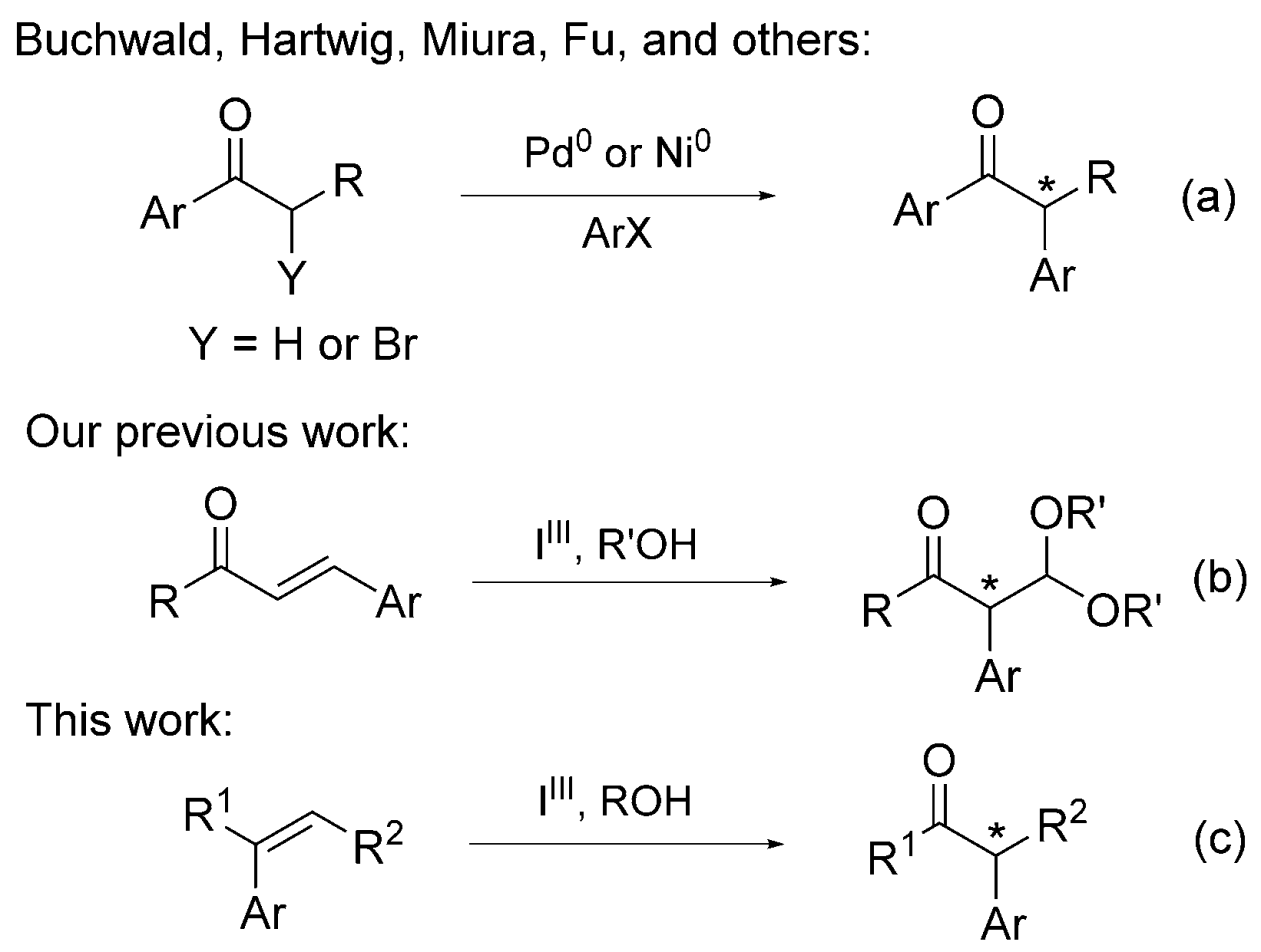
Asymmetric strategies to α‐arylated ketones.

Rearrangements induced by iodine(III) reagents are very versatile protocols to induce complexity and new stereocentres into molecules. Hypervalent iodine reagents exhibit attractive features of low cost, low toxicity and are environmentally benign.^1^ Their highly electrophilic nature^2^ coupled with the ability of the aryliodine(III) moiety to act as an excellent leaving group have seen their employment as much safer alternatives to more toxic heavy metal‐based oxidants, such as lead(IV) acetate, mercury(II) and thallium(III) salts. However, it remains a challenge to develop techniques to rival the synthetic utility of first‐ and second‐row transition‐metal catalysts. Great effort has been invested in the development of efficient methods for the synthesis of α‐aryl carbonyl compounds, in part due to their importance to the pharmaceutical industry.^3^ Driven primarily by the groups of Buchwald,^4^ Hartwig,^5^ Miura^6^ and Fu,^7^ palladium(0)^8^ and nickel(0)^9^‐catalyzed intermolecular arylation and cross‐coupling methods have emerged as powerful synthetic tools (Scheme 1 a).^10^ Increasing emphasis has recently been placed on the use of copper(I) catalysis,^11^ gold catalysis^12^ and more sustainable routes to α‐arylated ketones, such as metal‐free intermolecular reactions^13^ and rearrangements.^14^

## Results and Discussion

**Figure 1:**
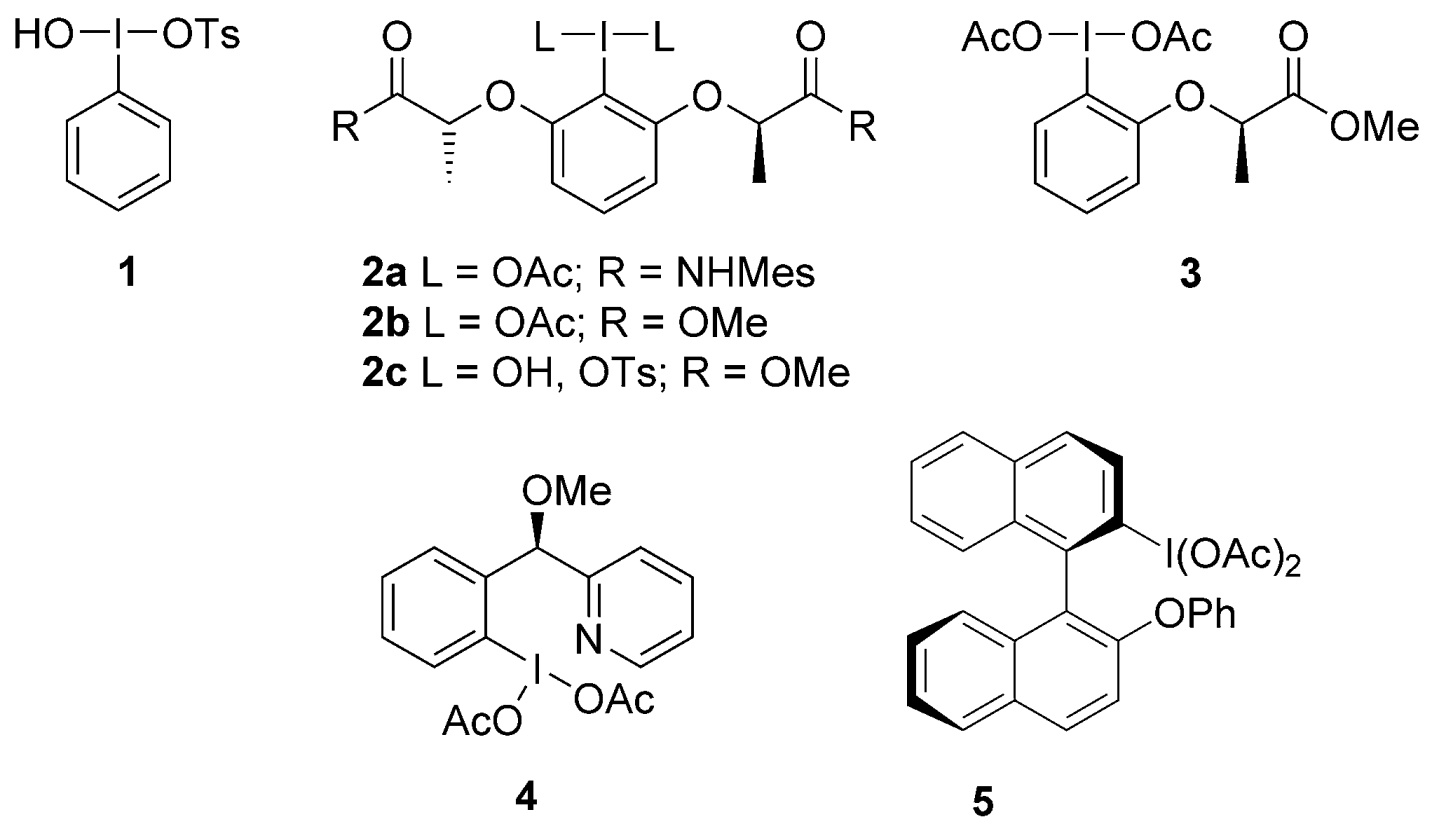
Hypervalent iodine reagents.

Hypervalent iodine reagents are known for their ability to promote efficient oxidation reactions of unsaturated systems. Following the seminal report by Koser et al. describing the oxidative rearrangement of 1,1‐diphenylethene with hydroxy(tosyloxy)iodobenzene (**1**; Figure 1),^15^ hypervalent iodine reagents have been utilised to generate cationic intermediates leading to oxidative skeletal rearrangements.^16^ Ring‐contraction and ring‐expansion reactions of cyclic alkenes have been demonstrated using **1**,^17^ and 1,2‐aryl shifts of acrylamide derivatives,^18^ arylalkenes^19^ and 1,1‐diphenylalkenes^20^ have been performed in a racemic fashion using **1** or its derivatives. Although chiral hypervalent iodine reagents in stereoselective reactions have received much attention,^21^ their use in stereoselective rearrangements are still scarce. We recently reported the first highly stereoselective rearrangement of chalcones to α‐aryl acetals promoted by hypervalent iodine reagent **2 a** 22 in the presence of trimethylsilyltriflate (TMSOTf; Scheme 1 b).^23^ As part of our studies on asymmetric α‐arylation strategies, herein, we report the transformation of aryl alkenes to α‐aryl ketones (Scheme 1 c). This transformation can also be performed using other reagents,^24^ but the important progress described herein is the first use of chiral iodine(III) derivatives under base‐free conditions to achieve the enantioselective synthesis of tertiary stereocenters of enolizable products.

We initially investigated the reaction of 1,1‐diphenylpentene **4 a** with reagent **2 b** 25 in dichloromethane/2,2,2‐trifluoroethanol (TFE; 10:1) in the presence of methanol as a nucleophilic oxygen source. No reaction occurred in the absence of any activating agent. When *p*‐toluenesulfonic acid monohydrate was added to **2 b** before addition of the alkene, ketone **7 a** resulting from an 1,2‐aryl migration and concomitant oxidation was obtained with good stereoselectivity albeit in moderate yield (Table 1, entry 2).

**Table 1:** Optimization of rearrangement conditions.

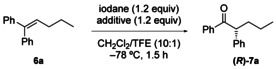					
Entry	Iodane^[a]^	Additive	MeOH [equiv]	Yield of **7 a** [%]	*ee* of **7 a** [%]
1	**2 b**	–	6	n.r.^[b]^	–
2	**2 b**	TsOH**⋅**H_2_O	6	49^[c]^	81
3	**2 b**	TsOH**⋅**H_2_O	6	78	92
4	**2 b**	TsOH**⋅**H_2_O	–	81^[d]^	92
5	**2 b**	TfOH	6	84	90
6	**2 b**	TMSOTf	6	86	90
7	**2 b**	TsOH**⋅**H_2_O	3	87	94
8	**2 a**	TsOH**⋅**H_2_O	3	86^[e]^	88
9	**3**	TsOH**⋅**H_2_O	3	80	83
10	**2 b** ^[f]^	TsOH**⋅**H_2_O	3	85	92
11	**2 b**	TsOH**⋅**H_2_O	–^[g]^	83	89
12	**2 b**	(+)‐10‐camphorsulfonic acid	3	81	89
13	**2 b**	(−)‐10‐camphorsulfonic acid	3	84	88
14	**4**	TsOH**⋅**H_2_O	3	55^[h]^	16
15	**5**	TsOH**⋅**H_2_O	3	77	44

[a] [Iodane]=0.09 m. [b] Reaction conditions: RT, 6 h. [c] Hypervalent iodine reagent prepared at 0 °C. [d] Reaction time 9 h. [e] Reaction time 5 h. [f] [Iodane]=0.2 m. [g] H_2_O (3 equiv); reaction time 9 h. [h] Reaction conditions: −78 to 0 °C, (*S*)‐**7 a** was obtained.

Addition of *p*‐toluenesulfonic acid monohydrate to a mixture of diacetate **2 b** and alkene at −78 °C to generate reagent **2 c** in situ gave superior results (Table 1, entry 3). The postulated enantiopure hydroxy(tosyloxy) derivative **2 c** could not be satisfactorily characterized by NMR spectroscopy due to its instability, but its formation was evidenced by a shift in UV/Vis spectrum absorption maxima in the reaction solvent system (see the Supporting Information). In the absence of methanol, the reaction proceeded satisfactorily, but required nine hours for completion (Table 1, entries 4 and 11). Limiting the amount of methanol to three equivalents minimized methoxy addition across the double bond and further improved the yield. Under these conditions, iodoarene **2 b** gave the highest enantioselectivity (Table 1, entry 7). Triflic acid (TfOH) and trimethylsilyltriflate (TMSOTf) also proved to be potent activators for **2 b**, although addition of six equivalents of methanol was required for these reactions to reach completion. It is assumed that rapid methanolysis of TMSOTf generates triflic acid in situ as the activating agent. Attempts to further improve the enantioselectivity of this transformation employing chiral activators, such as (1*R*)‐(−)‐ and (1*S*)‐(+)‐10‐camphorsulfonic acid, did not improve the outcome (Table 1, entries 12 and 13). Reaction of **6 a** with other chiral iodanes **4** and **5** gave the desired ketone with reduced enantioselectivities. Reagent **4** 26 led to the formation of (*S*)‐**7 a** with 16 % enantiomeric excess, whereas 44 % *ee* could be achieved by using iodane **5** 27 (Table 1, entries 14 and 15). In each reaction, the reduced iodoarene could be recovered (85–90 %) and was re‐oxidized without loss of enantiomeric purity.

**Scheme 2:**
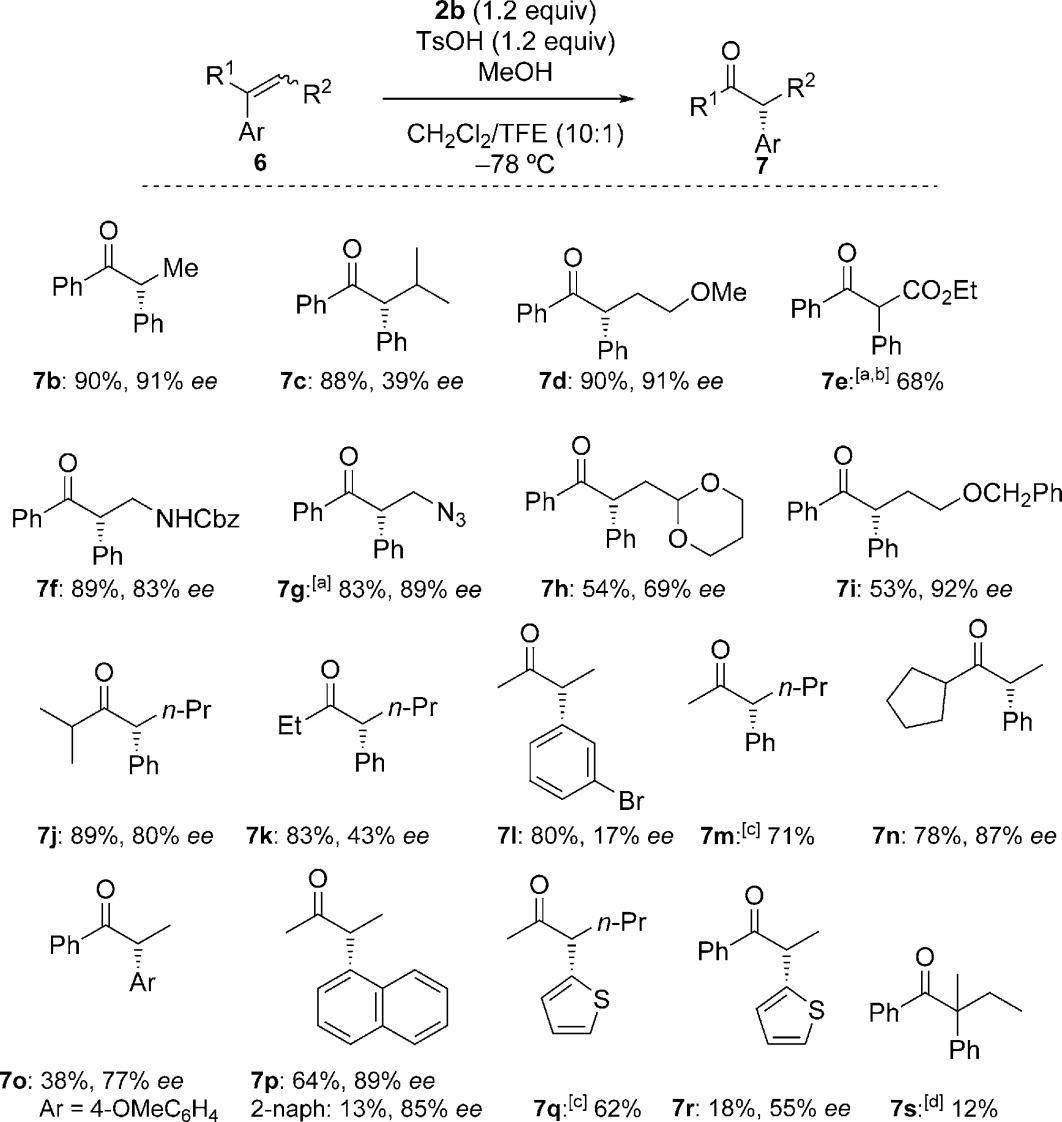
Scope of the oxidative rearrangement. [a] Conditions: TMSOTf (1.2 equiv), MeOH (6 equiv). [b] Obtained as the racemate. [c] Enantiomers inseparable under the HPLC conditions employed. [d] Yield obtained on reaction with (diacetoxyiodo)benzene as racemate.

Under the optimized reaction conditions, a range of 1,1‐ diphenyl alkenes (**6 b**–**i**) gave the corresponding α‐phenyl ketones (**7 b**–**i)** in good yields (Scheme 2).^28^ The absolute configuration of **7 b, k** and **o** was determined by comparison of the optical rotation with known compounds (see the Supporting Information). Using the identical enantiopure reagent **2 b**, the same direction of asymmetric induction is assumed in the other products **7**. Sterically hindered *iso*‐propyl substituted styrene **6 c** rearranged with only moderate enantioselectivity, which was not improved by the use of a less sterically encumbered reagent **3**,^29^ which gave **7 c** in 88 % yield, and only 39 % *ee* was obtained. It is noteworthy that access to ketone **7 c** is not possible by the intermolecular catalytic Negishi coupling protocol reported by Lou and Fu.7c 1,2‐Substituted styrenes **6 j**–**r** (used as a mixture of *E* and *Z* isomers) gave the expected rearranged ketones in good yields. It should be noted that in the case of **7 k**–**p**, products resulting from alkyl‐group migration were not observed. In the reaction of **7 j**, traces of an *iso*‐propyl migration product were observed in the ^1^H NMR spectrum of the crude mixture, but not in sufficient quantities to allow characterization.

When alkene **6 n** containing cyclopentyl ring was subjected to the rearrangement reaction conditions, rearranged ketone **7 n** was obtained in good yield and high selectivity (87 % *ee*). Unfortunately, 1‐cyclopropyl‐2‐methylstyrene bearing a cyclopropyl ring as substituent, gave a complex reaction mixture. Furthermore, oxidation of (*E*/*Z*)‐1‐(but‐2‐en‐2‐yl)naphthalene (**6 p**) with reagent **2 b** gave a mixture of 3‐(naphthalene‐1‐yl)butan‐2‐one (**7 p**; 89 % *ee*) and 3‐(naphthalene‐2‐yl)butan‐2‐one with 85 % *ee*.

Heterocyclic substituted alkenes were also used in the oxidative rearrangement. An *E*/*Z* mixture of the alkene **6 q** bearing a thiophene moiety gave rearranged product **7 q** through thienyl migration in good yields. Compound **6 r** gave the phenyl migrated product **7 r** in 18 % yield with 55 % *ee* together with various side products. Alkenes with pyridyl substituent, (*E*)‐2‐(1‐phenylprop‐1‐en‐1‐yl)pyridine and (*Z*)‐2‐(1‐phenylprop‐1‐en‐1‐yl)pyridine, afforded only trace amounts of the rearranged products.

Stereoselective rearrangements were also attempted with *tetra*‐substituted alkenes to construct quaternary carbon stereogenic centres. 1,1‐Diphenyl‐2‐methylbut‐1‐ene (**6 s**), prepared by a one‐pot cross‐Pinacol coupling/rearrangement reaction,^30^ was also exposed to the rearrangement reaction. The use of (diacetoxyiodo)benzene as iodine(III) reagent gave the rearranged product in 12 % isolated yield after nine hours; however, the rearranged ketone by using reagent **2 b** was not obtained in sufficient quantities to allow full characterization.

**Scheme 3:**
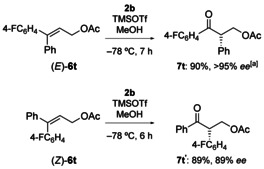
Chemoselectivity of the oxidative rearrangement. [a] Minor enantiomer was not detected.

When excess of alkene **6 m** (*E*/*Z*: 1:2.5; 2 equiv) was subjected to the standard reaction conditions, unreacted **6 m** was recovered with an enriched *E*/*Z* ratio of 3:1, suggesting that (*Z*)‐aryl substituent migration is faster at −78 °C. In addition, (*E*)‐**6 t** and (*Z*)‐**6 t** were independently synthesized and rearranged efficiently to ketones **7 t** and **t′**, respectively, with high stereoselectivities (Scheme 3). Under the standard reaction conditions (−78 °C), the (*Z*)‐aryl substituent migrates selectively with **2 b**; however, when the reaction is conducted at higher temperature (−20 °C), (*E*)‐aryl substituent migration became competitive ((*Z*)‐**6 t**→**7 t′**/**t** (8:1)), as was determined by ^1^H NMR spectroscopy. A similar trend in chemoselectivity was obtained with the Koser reagent. The reaction of (*Z*)‐**6 t** at −78 °C gave a ratio of **7 t′**/**t** (5:1), which diminished to **7 t′**/**t** (1:1) when the reaction was performed at room temperature.

**Scheme 4:**
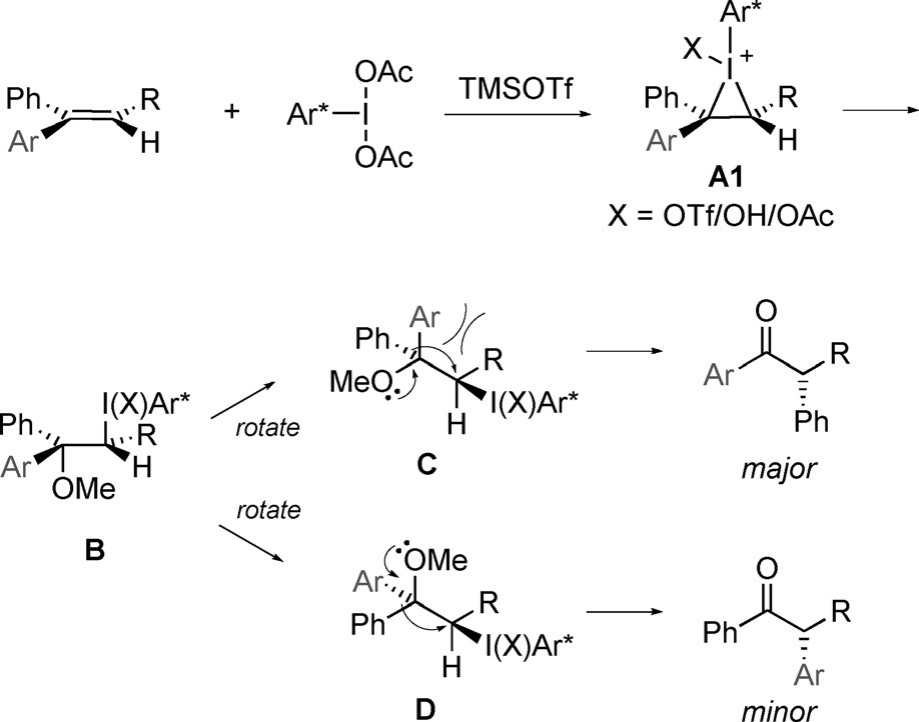
Plausible reaction pathway to explain the observed regio‐ and stereochemical outcome.

Taking this evidence into account, a plausible mechanistic pathway is proposed in Scheme 4. Electrophilic addition of iodine **2 c** to the alkene followed by ring opening with methanol would result in λ^3^‐iodane **B**. Following bond rotation to **C**, reductive elimination of the aryliodonio moiety gave a 1,2‐aryl migration with stereochemical inversion at this centre to give the observed product. However, it is not entirely clear why only conformer **C** is reactive at lower temperature. It is likely that relief of steric interactions between the Ar and R groups in **C** contribute to an increased propensity for this conformer to rearrange, providing the (*Z*)‐aryl migration product.

**Scheme 5:**
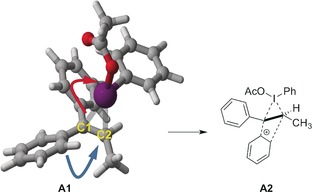
According to natural bond orbital (NBO) data of **A1**, the formation of a non‐classical carbenium ion **A2** as a direct precursor for product formation is feasible.

Key intermediate in the proposed mechanism is the cyclic iodonium ion **A**, because its restricted conformational space allows a regio‐ and stereoselective nucleophilic attack, as was demonstrated by using methanol (Scheme 4). At the same time, it may serve as the starting point for the formation of a non‐classical carbenium ion that could lead also to the major product observed (Scheme 5).

**Figure 2:**
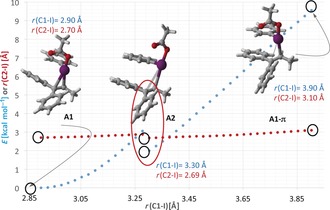
Relaxed PES scan by sequentially changing the C1−I bond length.

DFT calculations were employed to analyse the bonding situation and the stability of **A1** in comparison to the “open” form, (carbenium ion) and the nonclassical form **A2**. Although iodonium ions are usually preferred over carbenium ions in the addition of iodine electrophiles to alkenes,^31^ the structures described herein with at least a pronounced carbenium ion character in the benzylic position could be possible intermediates (Figure 2). To elucidate the relative energy of the cyclic and open cationic form, relaxed potential energy surface (PES) scans were performed^32^ by driving the C1−I bond (0.025 Å over 20 steps, two consecutive scans) starting from minimum **A1** 33 (*r*(C1−I) 2.90 Å, *r*(C2−I) 2.70 Å). The circled points were subjected to geometry optimization and frequency analysis; however, only **A1** could be confirmed as a ground‐state structure (Figure 2).

As was expected, the energy rises with increasing *r*(C1−I), but with 9.6 kcal mol^−1^ for a full acyclic carbenium ion (*r*(C1−I) 3.40 Å, *r*(C2−I) 2.73 Å), the anticipated barrier for the processes of ring opening should be accessible at room temperature. However, the associated structure could neither be characterized as a transition state nor as a ground state on the free energy surface, leaving room for other mechanistic scenarios, as indicated in Scheme 5. A second interesting feature of the PES scan plot is the shallow minimum at *r*(C1−I) 3.30 Å, *r*(C2−I) 2.69 Å (Figure 2). Starting geometry optimizations at this local minimum on the electronic energy surface did not lead to a stable minimum geometry with more carbenium‐ion character on the PES. On the contrary, the optimized structures relaxed back to the structure **A1**. Also, extensive search and optimizations under consideration of arene participation (**A2**) to stabilize a more carbenium‐ion‐like intermediate did not result in the identification of local minima (see the Supporting Information). To exclude that overbinding inherent to the M06‐2X^34^ functional due to the incorporation of dispersion corrections is biasing the geometry search, B3LYP^35^ was used as a complementary method, because it has no dispersion correction. The obtained B3LYP‐structures of **A1**‐π, though looser (+7 % for *r*(C1−I) and +1 % for *r*(C2−I) compared to the M06‐2X structure) and more asymmetric^36^ in their C−I bonds (*r*(C1−I) 3.10 Å, *r*(C2−I) 2.73 Å) did not differ qualitatively. Consequently, the proposed iodonium ion **A1** is believed to be the most prevalent structure in the reaction mechanism.

A number of commercially available symmetrical ketones was used to prepare alkenes **6 u**–**bb** to determine the effect of arene‐substitution pattern on the enantioselectivity of the rearrangement, as shown in Table 2. *ortho*‐Substitution as in **6 aa** completely shuts down the migration pathway with **2 b**; even with PhI(OAc)_2_ and TMSOTf upon heating at reflux for six hours, only a trace of the expected ketone was observed.

**Table 2:** Scope of the rearrangement with respect to 1,1‐diarylalkene.

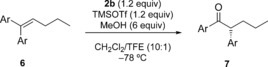					
Entry	Substrate	Ar	*t* [h]	Yield of **7** [%]	*ee* of **7** [%]
1	**6 u**	3‐Cl‐C_6_H_4_	6	68	68
2	**6 v**	3‐CF_3_‐C_6_H_4_	7	41	46
3	**6 w**	4‐Cl‐C_6_H_4_	6	79	77
4	**6 x**	4‐Br‐C_6_H_4_	6	82	83
5	**6 y**	4‐Me‐C_6_H_4_	1	90	86
6	**6 z**	4‐F‐C_6_H_4_	7	73	87
7	**6 aa**	2‐Cl‐C_6_H_4_	6	0	–
8	**6 bb**	4‐OMe‐C_6_H_4_	2	0^[a]^	–

[a] Major product: 1,1‐di(4‐methoxyphenyl)pentan‐2‐one (72 % yield).

Moderately, electron‐rich alkene **6 y** rearranges rapidly under the reaction conditions. Highly electron‐rich alkenes, such as anisole derivative **6 bb**, are preferential oxidized at the alkene *C*_2_ position, leading to 1,1‐di(4‐methoxyphenyl)pentan‐2‐one (72 % yield) as the major product (Table 2, entry 8).

The employment of catalytic amounts of iodine reagent in combination with an external oxidant for hypervalent iodine‐mediated transformations is an active area of research.^37^ It has been shown that electron‐rich 1‐alkyl‐1‐arylethenes can rearrange to ketones with sub‐stoichiometric quantities of 4‐methyl‐iodobenzenesulfonic acid.24b We found that more electron‐rich 1,1‐diarylalkenes, such as **6 a**, suffered from direct reaction with the terminal oxidants in preference to the iodine(I)→iodine(III) oxidation of chiral iodoarenes. Several oxidants including oxone, selectfluor, sodium perborate, peracetic acid and *tert*‐butyl hydroperoxide were investigated, and best results were obtained using *meta*‐chloroperoxybenzoic acid (*m*CPBA) and **2 a** (20 mol %, employed as the iodine(I) compound leading to ketone **7 a** in 34 % yield with 74 % *ee*.

**Scheme 6:**
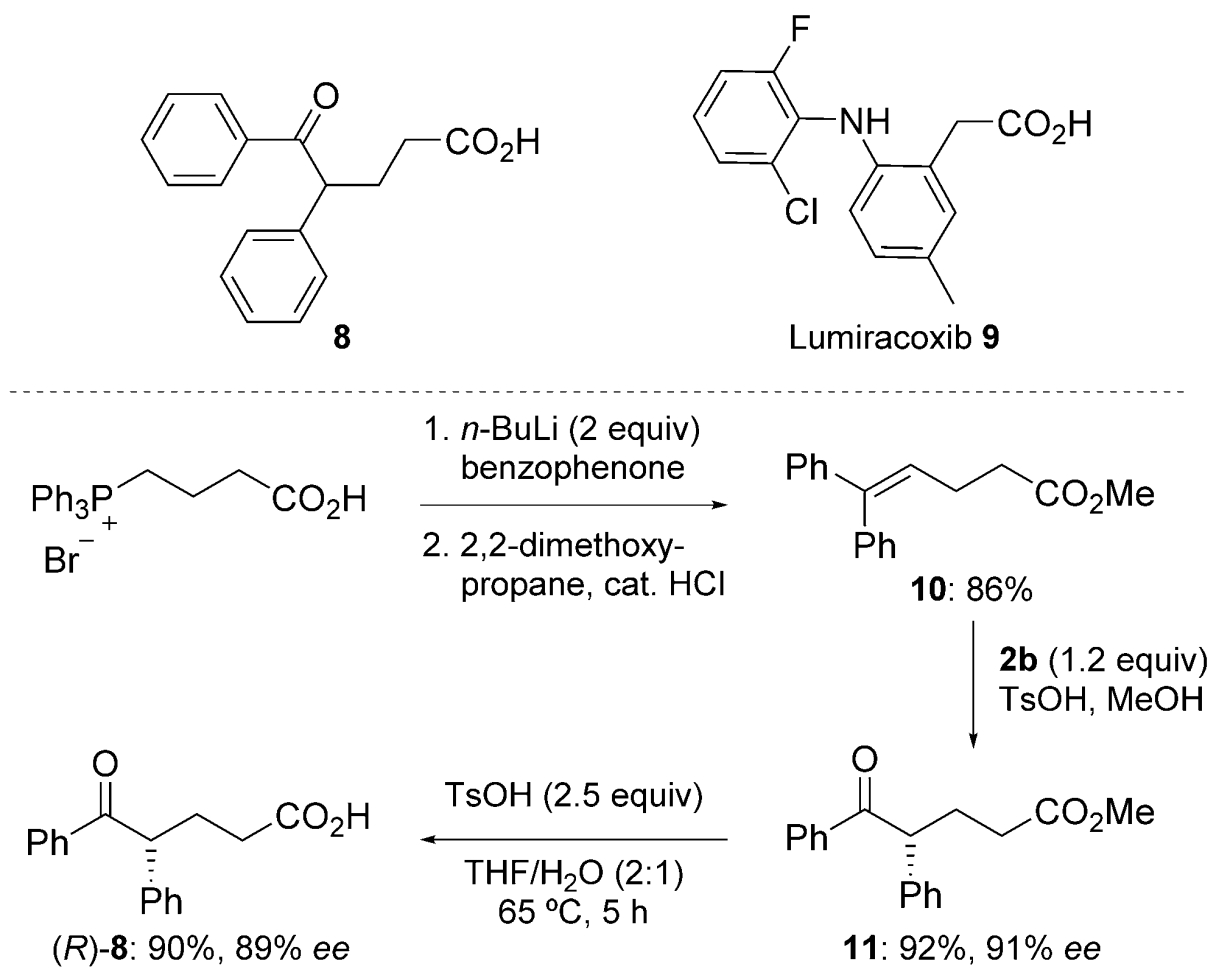
Asymmetric synthesis of lumiracoxib analogue (*R*)‐**8**.

The stereoselective rearrangement protocol described above was examined in a pharmaceutical context. Non‐steroidal anti‐inflammatory drugs (NSAIDs) have found widespread clinical application for their antipyretic, analgesic and anti‐inflammatory effects. Of these, cyclooxygenase‐2 (COX‐2)‐selective inhibitors have an established medicinal efficacy and have been the focus of recent studies to determine their therapeutic effects in tumour cell genesis and growth.^38^ Racemic aryl ketone **8** (Scheme 6) has been identified as an analogue of the highly selective COX‐2 inhibitor lumiracoxib (**9**; Novartis).^39^ From commercially available materials, *rac*‐**8** can be efficiently synthesized (89 % yield from methyl 5,5‐diphenylpent‐4‐enoate) and (*R*)‐**8** can be synthesized with high enantioselectivity (89 % *ee*), as shown in Scheme 6. This methodology avoids the potential toxicity from contamination with trace amounts of transition metals, which otherwise would be required for its synthesis. The conditions for the ester hydrolysis of **11** had to be carefully selected. Hydrolysis under basic conditions or with dilute hydrochloric acid led to unacceptable levels of racemization, but employing *p*‐toluenesulfonic acid avoided racemization almost completely. In addition, access to (*S*)‐**8** should be possible using the reagent *ent*‐**2 b** derived from commercially available (+)‐methyl lactate.

## Conclusion

We have developed a highly enantioselective oxidative rearrangement of various 1,1‐disubstituted alkenes employing chiral hypervalent iodine reagents. This method provides an attractive metal‐free route to α‐arylated ketones. The wider scope of such oxidative rearrangements is currently under investigation and will be reported in due course.

## Experimental Section

**Representative procedure for 7 a**: To the solution of alkene **6 a** (0.18 mmol), reagent **2 b** (0.20 mmol) and methanol (0.54 mmol) in CH_2_Cl_2_/TFE (10:1; 1.5 mL) at −78 °C, TsOH**⋅**H_2_O (0.18 mmol) was added. The reaction mixture was stirred until completion (TLC), then quenched with a 1:1 mixture of saturated aqueous NaHCO_3_ and saturated aqueous Na_2_S_2_O_3_ (0.5 mL). Then water (4 mL) was added, and the aqueous phase was extracted with CH_2_Cl_2_ (3×5 mL). The combined organic layers were filtered through a TELOS Phase Separator and concentrated under vacuum to give the crude product. Column chromatography (hexane/ethyl acetate 9:1→4:1) gave (*R*)‐**7 a** (87 %, 94 % *ee*) as a colourless oil.

## Supporting information

As a service to our authors and readers, this journal provides supporting information supplied by the authors. Such materials are peer reviewed and may be re‐organized for online delivery, but are not copy‐edited or typeset. Technical support issues arising from supporting information (other than missing files) should be addressed to the authors.

Supplementary

## References

[1a] Hypervalent Iodine Chemistry (Topics in Current Chemistry, Vol. 224 (Ed.: T. Wirth), Springer, Berlin, 2003;

[1b] V. V. Zhdankin , Hypervalent Iodine Chemistry, Wiley, Chichester, 2014.

[2a] M. Brown , U. Farid , T. Wirth , Synlett 2013, 24, 424–431;

[2b] D.-Q. Dong , S.-H. Hao , Z.-L. Wang , C. Chen , Org. Biomol. Chem. 2014, 12, 4278–4289. 24827449 10.1039/c4ob00318g

[3] C. A. Busacca , C. H. Senanayake , Transition Metal-Catalyzed Couplings in Process Chemistry: Case Studies from the Pharmaceutical Industry (Eds.: J. Magano, J. R. Dunetz), Wiley-VCH, Weinheim, 2003.

[4a] M. Palucki , S. L. Buchwald , J. Am. Chem. Soc. 1997, 119, 11108–11109;

[4b] J. Åhman , J. P. Wolfe , M. V. Troutman , M. Palucki , S. L. Buchwald , J. Am. Chem. Soc. 1998, 120, 1918–1919;

[4c] R. Martín , S. L. Buchwald , Angew. Chem. Int. Ed. 2007, 46, 7236–7239; 10.1002/anie.200703009 17722128

[5a] B. C. Hamann , J. F. Hartwig , J. Am. Chem. Soc. 1997, 119, 12382–12383;

[5b] X. Liao , Z. Weng , J. F. Hartwig , J. Am. Chem. Soc. 2008, 130, 195–200. 18076166 10.1021/ja074453g PMC2551326

[6] T. Satoh , Y. Kawamura , M. Miura , M. Nomura , Angew. Chem. Int. Ed. Engl. 1997, 36, 1740–1742;

[7a] X. Dai , N. A. Strotman , G. C. Fu , J. Am. Chem. Soc. 2008, 130, 3302–3303; 18302392 10.1021/ja8009428

[7b] P. M. Lundin , J. Esquivias , G. C. Fu , Angew. Chem. Int. Ed. 2009, 48, 154–156; 10.1002/anie.200804888 PMC2790061 19040243

[7c] S. Lou , G. C. Fu , J. Am. Chem. Soc. 2010, 132, 1264–1266. 20050651 10.1021/ja909689t PMC2814537

[8a] E. Marelli , M. Corpet , S. R. Davies , S. P. Nolan , Chem. Eur. J. 2014, 20, 17272–17276; 25414140 10.1002/chem.201404900

[8b] S. M. Raders , J. M. Jones , J. G. Semmes , S. P. Kelley , R. D. Rogers , K. H. Shaughnessy , Eur. J. Org. Chem. 2014, 7395–7404;

[8c] H. K. Potukuchi , A. P. Spork , T. J. Donohoe , Org. Biomol. Chem. 2015, 13, 4367–4373; 25789887 10.1039/c5ob00055f PMC4424829

[8d] L. Fan , S. Takizawa , Y. Takeuchi , K. Takenaka , H. Sasai , Org. Biomol. Chem. 2015, 13, 4837–4840. 25806855 10.1039/c5ob00382b

[9a] R. Takise , K. Muto , J. Yamaguchi , K. Itami , Angew. Chem. Int. Ed. 2014, 53, 6791–6794; 10.1002/anie.201403823 24845077

[9b] J. A. Fernández-Salas , E. Marelli , D. B. Cordes , A. M. Z. Slawin , S. P. Nolan , Chem. Eur. J. 2015, 21, 3906–3909. 25611197 10.1002/chem.201406457

[10] C. C. C. Johansson , T. J. Colacot , Angew. Chem. Int. Ed. 2010, 49, 676–707; 10.1002/anie.200903424 20058282

[11a] C. He , S. Guo , L. Huang , A. Lei , J. Am. Chem. Soc. 2010, 132, 8273–8275; 20518466 10.1021/ja1033777

[11b] A. E. Allen , D. W. C. MacMillan , J. Am. Chem. Soc. 2011, 133, 4260–4263; 21388207 10.1021/ja2008906 PMC3082494

[11c] A. Bigot , A. E. Williamson , M. J. Gaunt , J. Am. Chem. Soc. 2011, 133, 13778–13781; 21848264 10.1021/ja206047h

[11d] J. S. Harvey , S. P. Simonovich , C. R. Jamison , D. W. C. MacMillan , J. Am. Chem. Soc. 2011, 133, 13782–13785. 21848265 10.1021/ja206050b PMC3211083

[12] C. H. Cheon , O. Kanno , F. D. Toste , J. Am. Chem. Soc. 2011, 133, 13248–13251. 21815666 10.1021/ja204331w PMC3161142

[13a] B. Prüger , G. E. Hofmeister , C. B. Jacobsen , D. G. Alberg , M. Nielsen , K. A. Jørgensen , Chem. Eur. J. 2010, 16, 3783–3790; 20175160 10.1002/chem.200902911

[13b] X. Huang , N. Maulide , J. Am. Chem. Soc. 2011, 133, 8510–8513; 21574566 10.1021/ja2031882

[13c] Z. Jia , E. Gálvez , R. M. Sebastián , R. Pleixats , Á. Álvarez-Larena , E. Martin , A. Vallribera , A. Shafir , Angew. Chem. Int. Ed. 2014, 53, 11298–11301; 10.1002/anie.201405982 25196839

[13d] M. Pichette Drapeau , I. Fabre , L. Grimaud , I. Ciofini , T. Ollevier , M. Taillefer , Angew. Chem. Int. Ed. 2015, 54, 10587–10591; 10.1002/anie.201502332 26136406

[13e] C. Dey , E. Lindstedt , B. Olofsson , Org. Lett. 2015, 17, 4554–4557. 26352796 10.1021/acs.orglett.5b02270

[14a] M. Ochiai , Y. Kitagawa , N. Takayama , Y. Takaoka , M. Shiro , J. Am. Chem. Soc. 1999, 121, 9233–9234;

[14b] F. J. Fañanás , M. Alvarez-Pérez , F. Rodríguez , Chem. Eur. J. 2005, 11, 5938–5944; 15973744 10.1002/chem.200500070

[14c] P.-O. Norrby , T. B. Petersen , M. Bielawski , B. Olofsson , Chem. Eur. J. 2010, 16, 8251–8254. 20564301 10.1002/chem.201001110

[15a] G. F. Koser , L. Rebrovic , R. H. Wettach , J. Org. Chem. 1981, 46, 4324–4326;

[15b] L. Rebrovic , G. F. Koser , J. Org. Chem. 1984, 49, 2462–2472.

[16a] G. Maertens, S. Canesi, *Top. Curr. Chem* **2016**, DOI: 10.1007/128 2015 657; 10.1007/128_2015_657 26287122

[16b] F. V. Singh , T. Wirth , Synthesis 2013, 45, 2499–2511;

[16c] K. C. Guérard , A. Guérinot , C. Bouchard-Aubin , M.-A. Ménard , M. Lepage , M. A. Beaulieu , S. Canesi , J. Org. Chem. 2012, 77, 2121–2133. 22332792 10.1021/jo300169k

[17a] F. A. Siqueira , E. E. Ishikawa , A. Fogaça , A. T. Faccio , V. M. T. Carneiro , R. R. S. Soares , A. Utaka , I. R. M. Tébéka , M. Bielawski , B. Olofsson , L. F. Silva , J. Braz. Chem. Soc. 2011, 22, 1795–1807;

[17b] A. Ahmad , P. Scarassati , N. Jalalian , B. Olofsson , L. F. Silva , Tetrahedron Lett. 2013, 54, 5818–5820.

[18a] L. Liu , L. Du , D. Zhang-Negrerie , Y. Du , K. Zhao , Org. Lett. 2014, 16, 5772–5775; 25343425 10.1021/ol502834g

[18b] L. Liu , D. Zhang-Negrerie , Y. Du , K. Zhao , Synthesis 2015, 47, 2924–2930.

[19a] A. C. Boye , D. Meyer , C. K. Ingison , A. N. French , T. Wirth , Org. Lett. 2003, 5, 2157–2159; 12790553 10.1021/ol034616f

[19b] F. V. Singh , J. Rehbein , T. Wirth , ChemistryOpen 2012, 1, 245–250. 24551514 10.1002/open.201200037 PMC3922481

[20] M. W. Justik , G. F. Koser , Tetrahedron Lett. 2004, 45, 6159–6163.

[21] For leading reviews, see:

[21a] R. Kumar, T. Wirth, *Top. Curr. Chem* **2016**, DOI: 10.1007/128 2015 639; 10.1007/128_2015_639 26044514

[21b] F. Berthiol , Synthesis 2015, 47, 587–603;

[21c] A. Parra , S. Reboredo , Chem. Eur. J. 2013, 19, 17244–17260; 24272963 10.1002/chem.201302220

[21d] H. Liang , M. Ciufolini , Angew. Chem. Int. Ed. 2011, 50, 11849–11851; 10.1002/anie.201106127 22052680

[22] M. Uyanik , T. Yasui , K. Ishihara , Angew. Chem. Int. Ed. 2010, 49, 2175–2177; 10.1002/anie.200907352 20196156

[23] U. Farid , F. Malmedy , R. Claveau , L. Albers , T. Wirth , Angew. Chem. Int. Ed. 2013, 52, 7018–7022; 10.1002/anie.201302358 23653165

[24a] M. Zhu , Y. Zhao , Chin. Chem. Lett. 2015, 26, 248–250;

[24b] V. C. Purohit , S. P. Allwein , R. P. Bakale , Org. Lett. 2013, 15, 1650–1653. 23489019 10.1021/ol400432x

[25] M. Fujita , Y. Yoshida , K. Miyata , A. Wakisaka , T. Sugimura , Angew. Chem. Int. Ed. 2010, 49, 7068–7071; 10.1002/anie.201003503 20718062

[26] P. Mizar , A. Laverny , M. El-Sherbini , U. Farid , M. Brown , F. Malmedy , T. Wirth , Chem. Eur. J. 2014, 20, 9910–9913. 25042733 10.1002/chem.201403891 PMC4736455

[27] M. Brown , M. Delorme , F. Malmedy , J. Malmgren , B. Olofsson , T. Wirth , Synlett 2015, 26, 1573–1577.

[28] *Endo*- and *exo*-cyclic aryl alkenes have been shown to give rearrangement products on treatment with hydroxy(tosyloxy)iodobenzene **1** (see Ref. [19]). With reagents **2 b** and **3**, poor chemoselectivities were observed on reaction with model substrates 1-phenyl-1-cyclohexene and 9-butylidenefluorene.

[29] M. Fujita , S. Okuno , H. J. Lee , T. Sugimura , T. Okuyama , Tetrahedron Lett. 2007, 48, 8691–8694.

[30] U. Scheffler , R. Mahrwald , Helv. Chim. Acta 2012, 95, 1970–1975.

[31] S. Q. Jin , R. Z. Liu , Int. J. Quantum Chem. 1984, 25, 699–705.

[32] Gaussian 09, Revision D.01, M. J. Frisch, G. W. Trucks, H. B. Schlegel, G. E. Scuseria, M. A. Robb, J. R. Cheeseman, G. Scalmani, V. Barone, B. Mennucci, G. A. Petersson, H. Nakatsuji, M. Caricato, X. Li, H. P. Hratchian, A. F. Izmaylov, J. Bloino, G. Zheng, J. L. Sonnenberg, M. Hada, M. Ehara, K. Toyota, R. Fukuda, J. Hasegawa, M. Ishida, T. Nakajima, Y. Honda, O. Kitao, H. Nakai, T. Vreven, J. A. Montgomery, Jr., J. E. Peralta, F. Ogliaro, M. Bearpark, J. J. Heyd, E. Brothers, K. N. Kudin, V. N. Staroverov, R. Kobayashi, J. Normand, K. Raghavachari, A. Rendell, J. C. Burant, S. S. Iyengar, J. Tomasi, M. Cossi, N. Rega, J. M. Millam, M. Klene, J. E. Knox, J. B. Cross, V. Bakken, C. Adamo, J. Jaramillo, R. Gomperts, R. E. Stratmann, O. Yazyev, A. J. Austin, R. Cammi, C. Pomelli, J. W. Ochterski, R. L. Martin, K. Morokuma, V. G. Zakrzewski, G. A. Voth, P. Salvador, J. J. Dannenberg, S. Dapprich, A. D. Daniels, Ö. Farkas, J. B. Foresman, J. V. Ortiz, J. Cioslowski, D. J. Fox, Gaussian, Inc. Wallingford CT, **2009**. M06–2X/D95 V for H and D95 V [d] for all other atoms except iodine (LANL2DZ(d,p) with ECPs LANL2DZ).

[33] Conformational searches for **A1** were conducted in respect to the orientation of the phenyl ring attached to the iodide. π-stacking in **A1**-**π** cannot override steric repulsion and leads to a small energy difference in favour of **A1**.

[34] Y. Zhao , D. G. Truhlar , Theor. Chem. Acc. 2008, 120, 215–241. M06–2X has been chosen to model the various iodonium and carbenium ions because of its reasonable performance in standard non-covalent interaction tests. See:

[35a] C. T. Lee , W. T. Yang , R. G. Parr , Phys. Rev. B 1988, 37, 785–789; 10.1103/physrevb.37.785 9944570

[35b] A. D. Becke , J. Chem. Phys. 1993, 98, 5648–5652;

[35c] P. J. Stephens , F. J. Devlin , C. F. Chabalowski , M. J. Frisch , J. Phys. Chem. 1994, 98, 11623–11627;

[35d] A. D. Becke , Phys. Rev. A 1988, 38, 3098–3100; 9900728 10.1103/physreva.38.3098

[35e] W. J. Hehre , R. Ditchfie , J. A. Pople , J. Chem. Phys. 1972, 56, 2257–2261;

[35f] P. C. Hariharan , J. A. Pople , Theor. Chim. Acta 1973, 28, 213–222.

[36] Bonding asymmetry was determined by comparing the calculated C1−I and C2−I bond lengths with the C1′−I, C2′−I bond length of the parent iodonium ion **A1** for each level of theory.

[37a] M. Uyanik , T. Yasui , K. Ishihara , Angew. Chem. Int. Ed. 2013, 52, 9215–9218; 10.1002/anie.201303559 23873650

[37b] T. Dohi , N. Takenaga , T. Nakae , Y. Toyoda , M. Yamasaki , M. Shiro , H. Fujioka , A. Maruyama , Y. Kita , J. Am. Chem. Soc. 2013, 135, 4558–4566; 23445490 10.1021/ja401074u

[37c] P. Mizar , A. Burrelli , E. Günther , M. Söftje , U. Farooq , T. Wirth , Chem. Eur. J. 2014, 20, 13113–13116. 25156303 10.1002/chem.201404762

[38a] B. Singh , A. Lucci , J. Surg. Res. 2002, 108, 173–179; 12472107 10.1006/jsre.2002.6532

[38b] L. W. C. Chow , W. T. Y. Loo , M. Toi , Biomed. Pharmacother. 2005, 59, S281–S284; 10.1016/s0753-3322(05)80046-0 16507393

[38c] Y. Pan , S. Xu , X. Jia , H. Xu , Y. Zhang , Exp. Oncol. 2007, 29, 23–29. 17431384

[39] R. Bartzatt , Antiinflamm. Antiallergy Agents Med. Chem. 2014, 13, 17–28. 23984829 10.2174/18715230113129990019

