# Supplementary material for: Enantioselective Oxidative Rearrangements with Chiral Hypervalent Iodine Reagents
Source: Chemistry. 2016 Jan 21;22(12):4030–5. doi: 10.1002/chem.201504844 (PMC4797713; doi:10.1002/chem.201504844)
Supplement: Supplementary file 1 — Supplementary [file CHEM-22-4030-s001.pdf]

CHEMISTRY
A European Journal

Supporting Information

Enantioselective Oxidative Rearrangements with Chiral Hypervalent Iodine Reagents

Michael Brown,[a] Ravi Kumar,[a] Julia Rehbein,[b] and Thomas Wirth*[a]

chem_201504844_sm_miscellaneous_information.pdf

General	S2
Preparation of hypervalent iodine reagents	S3–S4
Experimental procedures and characterization data for compounds **6-11**	S5–S47
Assignment of Absolute Configuration	S48
^{1}H and ^{13}C NMR spectra	S49–S76
Calculations	S77–S93
References	S94–S95

General

All starting materials were purchased from commercial suppliers and used without further purification and all solvents used were dried and purified by standard techniques. Reactions requiring the exclusion of moisture were carried out under an atmosphere of argon or nitrogen in oven-dried glassware. Flash chromatography was carried out using Merck silica gel (35-70 μm) or on a Biotage Isolera Four platform using SNAP Ultra (25 μm) cartridges. Melting points were recorded on a Gallenkamp MPD350 apparatus. IR measurements were taken using a Perkin-Elmer 1600 FTIR spectrometer of solids (neat) and of oils (film). UV-Vis spectra were recorded using a Shimadzu UV-2600 Spectrophotometer. NMR spectra were recorded on Bruker DPX 300, Bruker DPX 400, Bruker DPX 500 or Oxford 300 spectrometer. ^{1}H NMR spectra were measured at 250, 300, 400 and 500 MHz. ^{13}C NMR spectra were measured at 75, 100, 125 and 150 MHz using $CDCl_3$ as the solvent and internal reference. Coupling constants *J* are given in Hz. Multiplicity as follows: s = singlet, d = doublet, t = triplet, q = quartet, sext = sextet, sept = septet, m = multiplet, br = broad. Low Resolution Mass Spectrometry (LRMS) was carried out using a Varian Saturn 2 GC-MS, Waters LCR Premier XE TOF or Voyager DE-STR spectrometer. High Resolution Mass Spectrometry (HRMS) was carried out at the EPSRC National Mass Spectrometry Facility, Swansea. Optical rotations were measured with a UniPol L polarimeter and are quoted in units 10^{-1} deg cm^{-2} g^{-1}. High-performance liquid chromatography (HPLC) analysis was conducted using Shimadzu LC-10 AD coupled diode array-detector SPD-MA-10A-VP.

Preparation of hypervalent iodine reagents

(2*R*,2'*R*)-2,2'-{[2-(Diacetoxy)iodo-1,3-phenylene]bis(oxy)}bis(*N*-mesitylpropanamide) (2a)

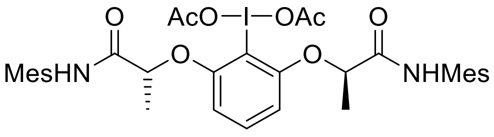

Prepared according to the literature.[1] Mp. 118-120 °C

^{1}H NMR (400 MHz, $CDCl_3$): δ = 8.35 (2H, s), 7.58 (1H, t, *J* = 8 Hz), 6.93 (1H, t, *J* = 8 Hz), 6.80 (4H, s), 5.15 (2H, q, *J* = 7 Hz), 2.22 (6H, s), 1.90-1.80 (m, 12H), 1.89 (6H, d, *J* = 7 Hz), 1.50 (6H, s) ppm.

(2*R*,2'*R*)-Diethyl 2,2'-{[2-(diacetoxy)iodo-1,3-phenylene]bis(oxy)}dipropanoate (2b)

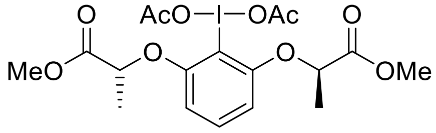

Prepared by Selectflour® oxidation of (2*R*,2'*R*)-dimethyl 2,2'-(2-iodo-1,3-phenylene)bis(oxy)dipropanoate with an optical rotation of $[\alpha]_D^{20}$ = –16.6 (c = 1.2, $CHCl_3$).[2]

^{1}H NMR (500 MHz, $CDCl_3$): δ = 7.39 (1H, t, *J* = 8 Hz), 6.58 (2H, d, *J* = 8 Hz), 4.87 (2H, q, *J* = 7 Hz), 3.75 (6H, s), 1.97 (6H, s), 1.68 (6H, d, *J* = 7 Hz) ppm; ^{13}C NMR (125 MHz, $CDCl_3$): δ = 176.9 (2C), 171.3 (2C), 156.7 (2C), 135.2 (2C), 107.0 106.3, 74.5 (2C), 52.5 (2C), 20.4 (2C), 18.3 (2C) ppm.

(1*R*)-(1-(Methoxycarbonyl)ethoxy)-2-(diacetoxyiodo)benzene (3)

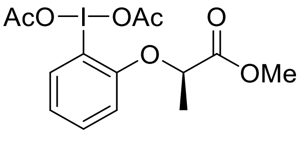

Prepared according to the literature.[3]

^{1}H NMR (300 MHz, $CDCl_3$): δ = 8.15 (1H, d, *J* = 8 Hz), 7.53 (1H, t, *J* = 8 Hz), 7.07 (1H, t, *J* = 8 Hz), 7.00 (1H, d, *J* = 8 Hz), 4.90 (1H, q, *J* = 7 Hz), 3.77 (3H, s), 1.98 (6H, s),

1.70 (3H, d, $J = 7$ Hz) ppm; ^{13}C NMR (75 MHz, $CDCl_3$): δ = 176.7 (2C), 171.3, 154.6, 137.9, 134.3, 123.7, 114.0, 113.3, 74.4, 52.6, 20.4 (2C), 18.4 ppm.

UV-Vis characterisation of hypervalent iodine reagent 2c

Hypervalent iodine reagent **2b** (11 mg, 0.02 mol) was dissolved in CH_2Cl_2/2,2,2-trifluoroethanol (10:1), (1 mL). An aliquot was taken and diluted to the desired concentration. Concurrently, TsOH•H_2O (4 mg, 0.02 mol) was dissolved in CH_2Cl_2/2,2,2-trifluoroethanol (10:1), (1 mL) and diluted accordingly.

To a separate solution of **2b** (11 mg, 0.02 mol) in CH_2Cl_2/2,2,2-trifluoroethanol (1 mL) was added TsOH•H_2O (4 mg, 0.02 mol) to give a yellow solution and the spectrum recorded. This solution was diluted by a factor of 15 and the spectrum re-recorded.

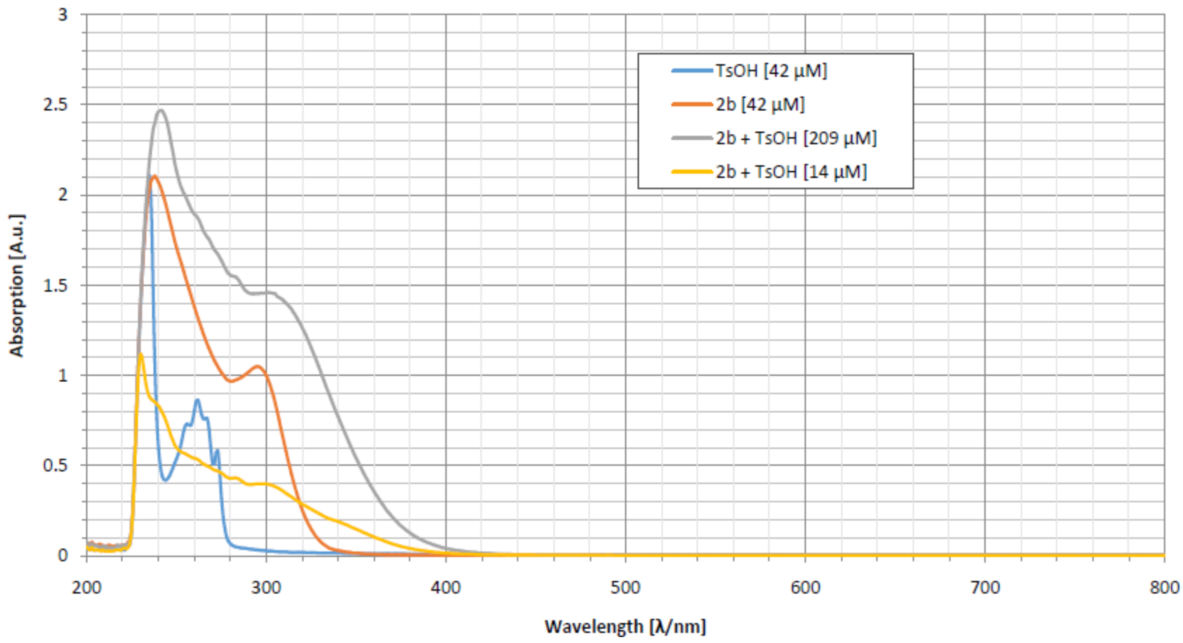

General procedure for Wittig olefination:

To the suspension of alkylphosphonium bromide (2.51 mmol) in THF at 0 ºC was added dropwise *n*-BuLi (2.51 mmol) and the reaction stirred until dissolution of the salt. The appropriate ketone (2.10 mmol) was added dropwise in THF (5 mL) and the reaction allowed to warm to room temperature and stirred until completion (tlc). The reaction was quenched with sat. NH_4Cl and extracted with diethyl ether (3×15 mL). Combined organic extracts were washed with brine (15 mL), dried ($MgSO_4$), filtered and concentrated under vacuum. Column chromatography (hexane:ethyl acetate, 1:0→10:1) afforded the product alkene which was stored under argon to minimise aerial oxidation.

1,1-Diphenylpentene (6a)

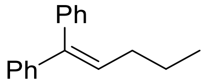

Butyltriphenylphosphonium bromide (2.63 g, 6.59 mmol), *n*-BuLi (2.1 M in hexane, 3.14 mL, 6.59 mmol) and benzophenone (1 g, 5.49 mmol) were reacted according to general olefination procedure to afford **6a** as a colourless oil (891 mg, 73%).

^{1}H NMR (300 MHz, $CDCl_3$): δ = 7.46-7.24 (10H, m), 6.17 (1H, t, *J* = 7 Hz), 2.17 (2H, q, *J* = 7 Hz), 1.54 (2H, sext, *J* = 7 Hz), 0.98 (3H, t, *J* = 7 Hz) ppm; ^{13}C NMR (75 MHz, $CDCl_3$): δ = 143.0, 141.6, 140.4, 130.2, 130.0 (2C), 128.2 (2C), 128.2 (2C), 127.3 (2C), 126.9, 126.8, 31.9, 23.3, 14.0 ppm. Data in agreement with literature.[4]

1,1-Diphenylpropene (6b)

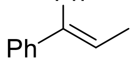

Ethyltriphenylphosphonium bromide (1.50 g, 4.04 mmol), *n*-BuLi (2.5 M in hexane, 1.62 mL, 4.04 mmol) and benzophenone (614 mg, 3.37 mmol) were reacted according to general olefination procedure to afford 6**b** as a colourless solid (458 mg, 70%). Mp.: 48.5-50 °C.

^{1}H NMR (400 MHz, $CDCl_3$): δ = 7.30 (2H, t, *J* = 7 Hz), 7.25-7.10 (8H, m), 6.10 (1H, q, *J* = 7 Hz), 1.69 (3H, d, *J* = 7 Hz) ppm; ^{13}C NMR (75 MHz, $CDCl_3$): δ = 143.0, 142.4,

140.0, 130.1 (2C), 128.2 (2C), 128.1 (2C), 127.2 (2C), 126.9, 126.8, 124.2, 15.8 ppm. Data in agreement with literature.[5]

3-Methyl-1,1-diphenyl-1-butene (6c)

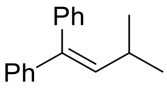

2-Methyl-isopropyltriphenylphosphonium bromide[6] (2.00 g, 5.01 mmol), *n*-BuLi (2.1 M in hexane, 2.39 mL, 5.01 mmol) and benzophenone (761 mg, 4.17 mmol) were reacted according to general olefination procedure to afford **6c** as a colourless oil (713 mg, 77%).

^{1}H NMR (500 MHz, $CDCl_3$): δ = 7.31-7.28 (2H, m), 7.25-7.21 (1H, m), 7.19-7.10 (7H, m), 5.82 (1H, d, *J* = 10 Hz), 2.42-2.32 (1H, m), 0.94 (6H, d, *J* = 7 Hz) ppm; ^{13}C NMR (75 MHz, $CDCl_3$): δ = 142.7, 140.6, 139.1, 137.3, 129.8 (2C), 128.2 (3C), 128.2 (2C), 127.2 (2C), 126.9, 126.8, 28.8, 23.3 ppm. Data in agreement with literature.[7]

4-Methoxy-1,1-diphenyl-1-butene (6d)

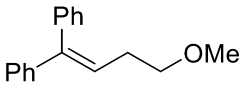

To a suspension of sodium hydride (60% in mineral oil, 107 mg, 2.67 mmol) in THF (5 mL) at 0 ºC was added dropwise a solution of 4,4-diphenyl-3-buten-1-ol[8] (200 mg, 0.89 mmol) in THF (1 mL) and the reaction stirred for 30 minutes. Iodomethane (278 µL, 4.46 mmol) was added dropwise and the reaction stirred at room temperature overnight. The reaction was quenched with sat. NH_4Cl and extracted with diethyl ether (3×5 mL). Combined organic extracts were washed with brine (5 mL), dried ($MgSO_4$), filtered and concentrated under vacuum. Column chromatography (hexane:ethyl acetate, 9:1 → 4:1) afforded **6d** as a colourless oil (199 mg, 94%).

IR (film): 2872, 1447, 1277, 1111, 748, 692 cm^{-1}.

^{1}H NMR (300 MHz, $CDCl_3$): δ = 7.32-7.10 (10H, m), 6.05 (1H, t, *J* = 7 Hz), 3.38 (2H, t, *J* = 7 Hz), 3.25 (3H, s), 3.23 (2H, q, *J* = 7 Hz) ppm; ^{13}C NMR (75 MHz, $CDCl_3$): δ = 143.3, 142.5, 140.0, 129.9 (2C), 128.3 (2C), 128.1 (2C), 127.3 (2C), 127.0 (2C), 125.8, 72.5, 58.7, 30.4 ppm.

MS (EI^+) *m/z* = 238 (M^+, 100); HRMS (ES^+) *m/z* calcd for $C_{17}H_{19}O$ $(M+H)^+$: 239.1430; found: 239.1428.

Ethyl-3,3-diphenylacrylate (6e)

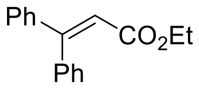

Prepared from triethyl phosphonoacetate (327 μL, 1.65 mmol), sodium hydride (60% in mineral oil, 66 mg, 2.67 mmol) and benzophenone (250 mg, 1.37 mmol) in THF (5 ml) according to the literature.[9] Column chromatography (hexane:ethyl acetate, 4:1→2:1) afforded **6e** as a colourless oil (200 mg, 56%).

^{1}H NMR (400 MHz, $CDCl_3$): δ = 7.32-7.21 (8H, m), 7.15-7.12 (2H, m), 6.29 (1H, s), 3.98 (2H, q, *J* = 7 Hz), 1.03 (3H, t, *J* = 7 Hz) ppm; ^{13}C NMR (75 MHz, $CDCl_3$): δ = 166.1, 156.5, 140.8, 139.0, 129.4, 129.1 (2C), 128.4 (2C), 128.3 (2C), 128.1, 127.9 (2C), 117.5, 60.0, 14.0 ppm. Data in agreement with literature.[9]

(*N*-Benzyloxycarbonyl)-3,3-diphenyl-2-propene-1-amine (6f)

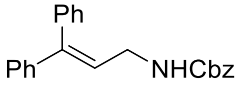

To a biphasic mixture of 3,3-diphenyl-2-propen-1-amine[10] (396 mg, 1.89 mmol) and $NaHCO_3$ (795 mg, 9.46 mmol) in H_2O (5 mL) and Et_2O (5 mL) at 0 ºC was added dropwise benzyl chloroformate (327 μL, 2.18 mmol) and the reaction stirred at room temperature overnight. The reaction was brought to pH 7 by the addition of sat. NH_4Cl solution and extracted with diethyl ether (3×10 mL). Combined organic extracts were washed with brine (5 mL), dried ($MgSO_4$), filtered and concentrated under vacuum. Column chromatography (hexane:ethyl acetate, 9:1→4:1) afforded **6f** as a colourless solid (462 mg, 71%). Mp.: 83-84 ºC.

IR (neat): 3329, 3030, 1697, 1518, 1244, 754, 694, 498 cm^{-1}.

^{1}H NMR (500 MHz, $CDCl_3$): δ = 7.42-7.18 (15H, m), 6.13 (1H, t, *J* = 7 Hz), 5.14 (2H, s), 4.87-4.64 (1H, m), 3.92 (2H, t, *J* = 7 Hz) ppm; ^{13}C NMR (125 MHz, $CDCl_3$): δ = 156.3, 144.5, 141.6, 139.0, 136.6, 129.7 (2C), 128.6 (2C), 128.4 (2C), 128.2 (5C), 127.6 (2C), 127.5 (2C), 124.9, 66.8, 40.5 ppm.

MS (ES$^+$) m/z = 366 ([M+Na]$^+$, 100); HRMS (EI$^+$) m/z calcd for $C_{23}H_{21}NO_2Na$ (M+Na)$^+$: 336.1470; found: 336.1460.

3-Azido-1,1-diphenyl-1-propene (6g)

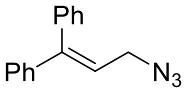

To a solution of 3-bromo-1,1-diphenyl-1-propene[11] (121 mg, 0.44 mmol) in DMF (5 mL) was added sodium azide (144 mg, 2.21 mmol) and the reaction stirred for 16 h. The reaction mixture was filtered through a pad of Celite (ethyl acetate). The filtrate was concentrated under vacuum and the product purified by column chromatography (ethyl acetate/hexane 10:1 → 4:1) to afford **6g** as a colourless oil (93 mg, 89%).

IR (film) 3022, 2087, 1444, 1237, 758, 694 cm^{-1}.

^{1}H NMR (500 MHz, $CDCl_3$): δ = 7.46-7.40 (3H, m), 7.36-7.30 (5H, m), 7.23-7.20 (2H, m), 6.22 (1H, t, J = 7 Hz), 3.90 (2H, d, J = 7 Hz) ppm; ^{13}C NMR (125 MHz, $CDCl_3$): δ = 147.2, 141.2, 138.4, 129.8 (2C), 128.5 (2C), 128.3 (2C), 128.1, 127.9, 127.7 (2C), 121.3, 49.8 ppm.

HRMS (APCI$^+$) m/z calcd for $C_{15}H_{14}N_3$ (M+H)$^+$: 236.1182; found: 236.1181.

2-(3,3-Diphenylallyl)-1,3-dioxane (6h)

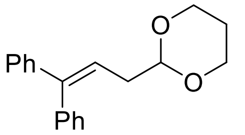

2-(1,3-Dioxanyl)ethyltriphenylphosphonium bromide (1.50 g, 3.29 mmol), *n*-BuLi (2.1 M in hexane, 1.57 mL, 3.29 mmol) and benzophenone (500 mg, 2.75 mmol) were reacted according to general olefination procedure to afford **6h** as a colourless solid (453 mg, 59%). Mp.: 67-69 ºC.

IR (neat): 2962, 2846, 1381, 1242, 1134, 1006, 763, 698 cm^{-1}.

^{1}H NMR (400 MHz, $CDCl_3$): δ = 7.33-7.39 (m, 2H), 7.31 (dt, 1H, J = 0.8 Hz, J = 7 Hz), 7.19-7.28 (m, 7H), 6.18 (t, 1H, J = 7 Hz), 4.61 (t, 1H, J = 5 Hz), 4.10-4.15 (m, 2H), 3.79 (dt, 2H, J = 12 Hz, J = 2 Hz), 2.46 (dd, 2H, J = 7 Hz, J = 2 Hz), 2.05-2.17 (m, 1H), 1.34-1.38

(m, 1H) ppm; ^{13}C NMR (100 MHz, $CDCl_3$): δ = 143.5, 142.5, 140.0, 129.9, 128.2, 128.0, 127.3, 127.0, 123.2, 101.9, 67.0, 35.9, 25.8 ppm.

HRMS (EI^+) *m/z* calcd for $C_{19}H_{10}O_2$ $(M)^+$: 280.1463; found: 280.1457.

4-Benzyloxy-1,1-diphenyl-but-1-ene (6i)

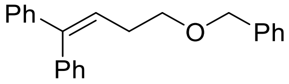

(3-(Benzyloxy)propyl)triphenylphosphonium bromide (1.61 g, 3.29 mmol), *n*-BuLi (2.1 M in hexane, 1.57 mL, 3.29 mmol) and benzophenone (500 mg, 2.75 mmol) were reacted according to general olefination procedure to afford **6i** as a colourless oil (430 mg, 50%).

IR: 3024, 2854, 1597, 1492, 1361, 1277, 1099, 763, 702 cm^{-1}.

^1H NMR (400 MHz, $CDCl_3$): δ = 7.10-7.31 (m, 15H), 6.07 (t, 1H, J = 7 Hz), 4.43 (s, 2H), 3.49 (t, 2H, J = 7 Hz), 2.38 (q, 2H, J = 7 Hz) ppm; ^{13}C NMR (125 MHz, $CDCl_3$): δ = 143.3, 142.6, 140.0, 138.5, 137.6, 132.5, 130.1, 130.0, 128.4, 128.3, 128.2, 128.1, 127.7, 127.7, 127.3, 127.0 (2C), 125.9, 72.9, 70.0, 30.5ppm. Data in agreement with literature.[12]

HRMS (EI^+) *m/z* calcd for $C_{23}H_{22}O$ $(M)^+$: 314.1671; found: 314.1671.

2-Methyl-3-phenyl-4-heptene (6j) (*E*/*Z*: 1:2.1)

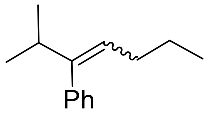

Butyltriphenylphosphonium bromide (1.0 g, 2.50 mmol), *n*-BuLi (1.6 M in hexane, 1.57 mL, 2.50 mmol) and isobutyrophenone (313 μL, 2.09 mmol) were reacted according to general olefination procedure to afford **6j** as a colourless oil (192 mg, 49%).

IR (film): 2961, 2870, 1458, 752, 702, 496 cm^{-1}.

^1H NMR (500 MHz, $CDCl_3$): δ = 7.25-7.12 (3.3H, m), 7.09-7.07 (1.5H, m), 7.00-6.98 (0.5H, m), 5.32 (0.3H(*E*), t, *J* = 7 Hz), 5.17 (0.8H(*Z*), t, *J* = 7 Hz), 2.97 (0.8H(*Z*), sept, *J* = 7 Hz), 2.46 (0.3H(E), sept, *J* = 7 Hz), 2.10 (1.6H(*Z*), q, *J* = 7 Hz), 1.72 (0.5H(*E*), q, *J* = 7 Hz), 1.38 (1.6H(*Z*), sept, *J* = 7 Hz), 1.24 (0.5H(*E*), sept, *J* = 7 Hz), 0.96 (4.6H(*Z*), d, *J* = 7 Hz), 0.93 (1.6H(*Z*), d, *J* = 7 Hz), 0.89 (2.4H(*Z*), t, *J* = 7 Hz), 0.74 (0.8H(*E*), t, *J* = 7 Hz) ppm; ^{13}C NMR (125 MHz, $CDCl_3$): δ = 147.5, 146.8, 143.6, 141.6, 129.0, 128.9, 128.7, 127.7, 127.4, 126.1, 126.0, 124.6, 35.9, 30.8, 29.8, 29.2, 23.2, 23.1, 21.9, 21.8, 13.9, 13.7 ppm.

MS (EI$^+$) m/z = 188 (M$^+$, 100); HRMS (APCI$^+$) m/z calcd for $C_{14}H_{21}$ (M+H)$^+$: 189.1638; found: 189.1637.

3-Phenyl-2-pentene (6k) (*E*/*Z*: 1:1.7)

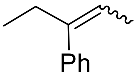

Ethyltriphenylphosphonium bromide (1.50 g, 4.04 mmol), *n*-BuLi (2.5 M in hexane, 1.62 mL, 4.04 mmol) and propiophenone (448 µL, 3.37 mmol) were reacted according to general olefination procedure to afford **6k** as a volatile colourless oil (458 mg, 70%).

^{1}H NMR (400 MHz, $CDCl_3$): δ = 7.42-7.27 (3.6H, m), 7.21 (1.2H, m), 5.79 (0.4H(*E*), q, *J* = 7 Hz), 5.59 (0.6H(*Z*), q, *J* = 7 Hz), 2.58 (0.7H(*E*), q, *J* = 7 Hz), 2.40 (1.2H(*Z*), q, *J* = 7 Hz), 1.86 (1.1H, d, *J* = 7 Hz), 1.62 (2.0H, d, *J* = 7 Hz), 1.07-1.00 (3.0H, m) ppm; ^{13}C NMR (125 MHz, $CDCl_3$): δ = 143.5, 143.2, 142.4, 141.4, 128.5, 128.1, 128.0, 126.4, 126.3, 126.2, 122.0, 119.8, 32.0, 22.6, 14.6, 13.9, 13.2, 13.1 ppm. Data in agreement with literature.[13]

2-(3-Bromophenyl)-2-hexene (6l) (*E*/*Z*: 1:2.1)

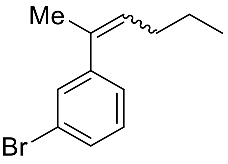

Butyltriphenylphosphonium bromide (1.50 g, 3.76 mmol), *n*-BuLi (1.8 M in hexane, 2.09 mL, 3.76 mmol) and 3'-bromoacetophenone (414 µL, 3.13 mmol) were reacted according to general olefination procedure to afford **6l** as a colourless oil (599 mg, 80%).

IR (film): 2959, 2928, 1589, 1553, 1472, 779, 698 cm^{-1}.

^{1}H NMR (500 MHz, $CDCl_3$): δ = 7.53 (0.3H, s), 7.39-7.30 (2.1H, m), 7.22-7.16 (1.1H, m), 7.11 (0.7H, d, *J* = 8 Hz), 5.81 (0.3H(*E*), t, *J* = 7 Hz), 5.50 (0.7H(*Z*), t, *J* = 7 Hz), 2.19 (0.7H(*E*), q, *J* = 7 Hz), 2.01 (3.1H, s), 1.94 (1.5H(*Z*), q, *J* = 7 Hz), 1.50 (0.7H(*E*), sext, *J* = 7 Hz), 1.37 (1.4H(*Z*), sext, *J* = 7 Hz), 0.97 (1.0H(*E*), t, *J* = 7 Hz), 0.87 (2.1H(*Z*), t, *J* = 7 Hz) ppm; ^{13}C NMR (125 MHz, $CDCl_3$): δ = 146.3, 144.5, 134.8, 133.6, 131.0, 129.6, 129.4, 129.3, 128.8, 126.7, 124.2, 122.4, 122.1, 31.1, 30.9, 25.4, 23.1, 22.7, 15.7, 13.9, 13.7 ppm.

MS (EI$^+$) m/z = 240 [M(^{81}Br)$^+$, 50], 238 [M(^{79}Br)$^+$, 50]; HRMS (ES$^+$) m/z calcd for $C_{12}H_{16}{}^{79}Br$ (M+H)$^+$: 239.0431; found: 239.0430.

2-Phenyl-2-hexene (6m) (*E*/*Z*: 1:2.5)

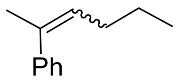

Butyltriphenylphosphonium bromide (1.50 g, 3.76 mmol), *n*-BuLi (1.8 M in hexane, 2.09 mL, 3.76 mmol) and acetophenone (367 μL, 3.13 mmol) were reacted according to general olefination procedure to afford **4k** as a colourless oil (277 mg, 74%).

^{1}H NMR (300 MHz, $CDCl_3$): δ = 7.44-7.21 (5.2H, m), 5.83 (0.3H(*E*), t, *J* = 7 Hz), 5.51 (0.6H(*Z*), t, *J* = 7 Hz), 2.23 (0.6H(*E*), q, *J* = 7 Hz), 2.07 (2.7H, s), 2.00 (1.3H(*Z*), q, *J* = 7 Hz), 1.56-1.49 (0.8H(*E*), m), 1.40 (1.4H(*Z*), sext, *J* = 7 Hz), 1.00 (1.2H(*E*), t, *J* = 7 Hz), 0.89 (2.0H(*Z*), t, *J* = 7 Hz) ppm; ^{13}C NMR (75 MHz, $CDCl_3$): δ = 144.1, 142.3, 136.1, 134.7, 128.6, 128.3, 128.2, 128.0, 127.8, 126.5, 126.4, 125.6, 125.1, 31.2, 30.9, 25.6, 23.3, 22.8, 15.8, 14.0, 13.8 ppm. Data in agreement with literature.[14]

1-Cyclopentyl-1-phenylpropene (6n) (*E/Z:* 2:3)

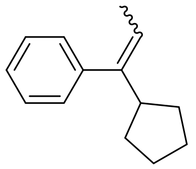

Ethyl triphenylphosphonium bromide (1.22 g, 3.29 mmol), *n*-BuLi (2.1 M in hexane, 1.57 mL, 3.29 mmol) and cyclopentyl phenyl ketone (479 mg, 2.75 mmol) were reacted according to general olefination procedure to afford **6n** as a colourless oil (302 mg, 59%).

IR (neat): 2951, 2866, 1492, 1438, 1072, 829, 759, 702 cm^{-1}.

^{1}H NMR (400 MHz, $CDCl_3$): δ = 7.95-8.00 (m, 2H), 7.51-7.58 (m, 1H), 7.43-7.50 (m, 2H), 7.05-7.35 (m, 6H), 5.53 (dq, 1H, *J* = 6 Hz, *J* = 1 Hz), 5.41 (q, 1H, *J* = 7 Hz), 3.68-3.77 (m, 1H), 2.99-3.10 (m, 1H), 2.59-2.71 (m, 1H), 1.88-1.96 (m, 4H), 1.77 (d, 3H, *J* = 7 Hz), 1.50-1.62 (m, 7H), 1.46 (dd, 3H, *J* = 7 Hz, *J* = 1 Hz), 1.23-1.43 (m, 4H) ppm; ^{13}C NMR (100 MHz, $CDCl_3$): δ = 145.3, 144.9, 144.0, 141.7, 129.0, 128.7, 127.9, 127.5, 126.1, 126.0, 123.8, 119.0, 48.3, 41.1, 31.6, 31.2, 25.2, 24.6, 14.6, 13.6 ppm.

HRMS (EI^+) *m/z* calcd for $C_{14}H_{18}$ $(M)^+$: 186.1409; found: 186.1407.

1-(4-Anisyl)-1-phenylpropene (6o) (*E/Z*: 1:1)

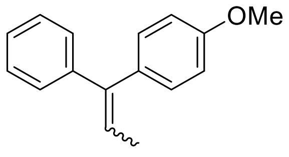

Ethyl triphenylphosphonium bromide (1.22 g, 3.29 mmol), *n*-BuLi (2.1 M in hexane, 1.57 mL, 3.29 mmol) and 4-methoxybenzophenone (584 mg, 2.75 mmol) were reacted according to general olefination procedure to afford **6** as a white solid (370 mg, 60%). Mp.: 47-49 ºC.

^{1}H NMR (400 MHz, $CDCl_3$): δ = 7.34-7.38 (m, 2H), 7.09-7.30 (m, 12H), 6.90-6.92 (m, 2H), 6.78-6.81 (m, 2H), 6.05-6.14 (2q, 2H), 3.84 (s, 3H), 3.78 (s, 3H), 1.73 (d, 3H, *J* = 6 Hz), 1.73 (d, 3H, *J* = 6 Hz) ppm; ^{13}C NMR (100 MHz, $CDCl_3$): δ = 159.0, 158.8, 143.8, 142.4, 142.2, 140.7, 136.1, 132.7, 131.6, 130.5, 128.7, 128.6, 128.5, 127.7, 127.2, 127.1, 124.3, 122.9, 113.9, 113.8, 55.7, 55.6, 16.3, 16.1 ppm. Data in agreement with literature.[15]

1-(But-2-en-2-yl)naphthalene (6p) (E/Z: 7:3)

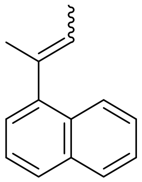

Ethyl triphenylphosphonium bromide (1.22 g, 3.29 mmol), *n*-BuLi (2.1 M in hexane, 1.57 mL, 3.29 mmol) and 1-acetylnapthalene (468 mg, 2.75 mmol) were reacted according to general olefination procedure to afford **6** as a colourless oil (285 mg, 57%).

^{1}H NMR (400 MHz, $CDCl_3$): δ = 7.94-7.96 (m, 1H), 7.72-7.88 (m, 4H), 7.39-7.48 (m, 4H), 7.21-7.24 (m, 2H), 5.79 (qq, 1H, *J* = 7 Hz, *J* = 1 Hz, E-isomer), 5.57 (qq, 1H, *J* = 7 Hz, *J* = 1 Hz, Z-isomer), 2.09 (t, 3H, *J* = 1 Hz), 1.86 (dd, 3H, *J* = 7 Hz, *J* = 1 Hz), 1.56 (s, 3H), 1.33 (dq, 3H, *J* = 7 Hz, *J* = 1 Hz) ppm; ^{13}C NMR (100 MHz, $CDCl_3$): δ = 144.7, 141.0, 136.3, 136.2, 134.3, 134.2, 131.8, 131.3, 128.9, 128.8, 128.1, 127.2, 126.5, 126.3, 126.1 (2C), 126.0 (2C), 125.7, 125.5, 125.4, 123.8, 26.6, 19.2, 15.4, 14.5 ppm. Data in agreement with literature.[16]

2-(Hex-2-en-2-yl)thiophene (6q) (*E/Z*: 1:4)

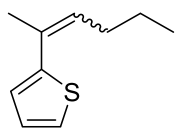

Butyl triphenylphosphonium bromide (1.313 g, 3.29 mmol), *n*-BuLi (2.1 M in hexane, 1.57 mL, 3.29 mmol) and 2-acetylthiophene (347 mg, 2.75 mmol) were reacted according to general olefination procedure to afford **6q** as a colourless oil (287 mg, 63%).

IR (neat): 2958, 2927, 2870, 1462, 1377, 852, 694 cm^{-1}.

^{1}H NMR (400 MHz, $CDCl_3$): δ = 7.24-7.60 (m, 1H), 7.08 (dd, 1H, *J* = 2 Hz, *J* = 1 Hz, *E*-isomer), 7.01-7.03 (m, 1H), 6.98-7.00 (m, 1H), 6.94-6.96 (m, 2H, *E*-isomer), 5.94 (dt, 1H, *J* = 6 Hz, *J* = 1 Hz, E-isomer), 5.50 (dt, 1H, *J* = 6 Hz, *J* = 1 Hz, *Z*-isomer), 2.27 (dq, 2H, *J* = 6 Hz, *J* = 1 Hz), 2.12-2.20 (m, 3H), 1.47 (sext, 2H, *J* = 6 Hz), 0.95 (t, 3H, *J* = 6 Hz), 0.94 (t, 3H, *J* = 6 Hz) ppm; ^{13}C NMR (100 MHz, $CDCl_3$): δ = 129.6, 128.2, 127.2, 126.5, 125.4, 125.0, 124.2, 31.7, 31.2, 25.7, 23.2, 23.0, 14.0 ppm.

HRMS (EI^+) *m/z* calcd for $C_{10}H_{14}S$ $(M)^+$: 166.0816; found: 166.0815.

2-(1-Phenylprop-1-enyl)thiophene (6r) (*E/Z*: 1:1)

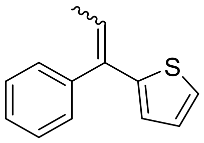

Ethyl triphenylphosphonium bromide (1.22 g, 3.29 mmol), *n*-BuLi (2.1 M in hexane, 1.57 mL, 3.29 mmol) and 2-benzoylthiophene (517 mg, 2.75 mmol) were reacted according to general olefination procedure to afford **6** as a colourless oil (323 mg, 57%).

IR (neat): 3020, 2908, 1492, 1438, 1222, 829, 763, 702 cm^{-1}.

^{1}H NMR (400 MHz, $CDCl_3$): δ = 7.29-7.47 (m, 11H), 7.15 (dd, 1H, *J* = 5 Hz, *J* = 1 Hz), 7.10 (dd, 1H, *J* = 4 Hz, *J* = 2 Hz), 6.97 (dd, 1H, *J* = 4 Hz, *J* = 2 Hz), 6.92 (dd, 1H, *J* = 4 Hz, *J* = 1 Hz), 6.61 (dd, 1H, *J* = 4 Hz, *J* = 1 Hz), 6.30 (q, 1H, *J* = 7 Hz), 6.15 (q, 1H, *J* = 7 Hz), 2.00 (d, 3H, *J* = 7 Hz), 1.73 (d, 3H, *J* = 7 Hz) ppm; ^{13}C NMR (100 MHz, $CDCl_3$): δ = 147.4, 143.3, 141.6, 139.1, 136.7, 135.8, 129.7, 128.3, 128.1, 127.9, 127.6, 127.4, 127.2, 127.0, 126.7, 125.5, 124.7, 123.5, 123.2, 16.0, 15.3 ppm.

HRMS (EI^+) *m/z* calcd for $C_{13}H_{12}S$ $(M)^+$: 200.0660; found: 200.0655.

2-Methyl-1,1-diphenylbut-1-ene (6s)

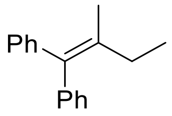

2-Methyl-1,1-diphenylbut-1-ene (**6s**) was prepared *via* a one-pot cross-Pinacol coupling/rearrangement procedure (yield: 28%).[17]

^{1}H NMR (400 MHz, $CDCl_3$): δ = 7.26-7.31 (m, 4H), 7.15-7.22 (m, 6H), 2.14 (q, 2H, *J* = 8 Hz), 1.79 (s, 3H), 1.07 (t, 3H, *J* = 8 Hz) ppm; ^{13}C NMR (100 MHz, $CDCl_3$): δ = 143.4, 137.0, 136.5, 129.7, 129.5, 128.0, 127.9, 126.1, 126.0, 28.5, 19.0, 13.3 ppm.

HRMS (EI^+) *m/z* calcd for $C_{17}H_{18}$ $(M)^+$: 222.1409; found: 222.1402.

Stereoselective Synthesis of (*Z*)-(6t) and (*E*)-(6t)[18]

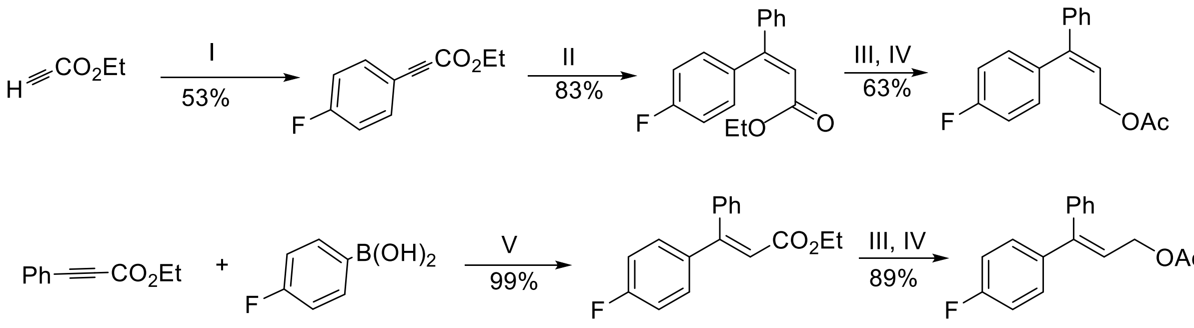

Conditions: I: 4−F−C_6H_4−$B(OH)_2$, CuI, Ag_2O, Cs_2CO_3, 80 °C, 48 h;[19] II: Ph−$B(OH)_2$, Cu(I)OAc, MeOH, rt, 16 h;[20] III: $NaBH_4$, $ZnCl_2$, NEt_3, THF, reflux 3 h;[18] IV: Ac_2O, pyridine, rt, 16 h; V: Cu(I)OAc, MeOH, rt, 36 h.[20]

(*Z*)-3-(4-Fluorophenyl)-3-phenylallyl acetate (*Z*)-(6t)

^{1}H NMR (500 MHz, $CDCl_3$): δ = 7.30-7.27 (3H, m), 7.24-7.22 (2H, m), 7.17-7.14 (2H, m), 7.07 (2H, t, *J* = 9 Hz), 6.17 (1H, t, *J* = 7 Hz), 4.62 (2H, d, *J* = 7 Hz), 2.07 (3H, s) ppm; ^{13}C NMR (75 MHz, $CDCl_3$): δ = 171.0, 162.4 (d, $J_{C\text{-}F}$ = 247 Hz), 145.5, 141.4, 134.5 (d, $J_{C\text{-}F}$ = 3 Hz), 131.4 (2C, d, $J_{C\text{-}F}$ = 8 Hz), 128.3 (2C), 128.0, 127.7 (2C), 122.7, 115.4 (2C, d, $J_{C\text{-}F}$ = 22 Hz), 62.5, 21.1 ppm; ^{19}F NMR (282 MHz, $CDCl_3$): δ = −113.9 ppm.

MS (EI^+) *m/z* = 270 (M^+, 100). Data in agreement with literature.[21]

(*E*)-3-(4-Fluorophenyl)-3-phenylallyl acetate (*E*)-(6t)

^{1}H NMR (300 MHz, $CDCl_3$): δ = 7.43-7.35 (3H, m), 7.25-7.16 (4H, m), 6.98 (2H, t, *J* = 9 Hz), 6.13 (2H, t, *J* = 7 Hz), 4.61 (2H, d, *J* = 7 Hz), 2.09 (3H, s) ppm; ^{13}C NMR (75 MHz, $CDCl_3$): δ = 171.0, 162.6 (d, J_{C-F} = 247 Hz), 145.4, 138.4, 137.6 (d, J_{C-F} = 4 Hz), 129.6 (2C), 129.4 (2C, d, J_{C-F} = 7 Hz), 128.4 (2C), 128.0, 122.2, 115.1 (2C, d, J_{C-F} = 22 Hz), 62.6, 21.2 ppm; ^{19}F NMR (282 MHz, $CDCl_3$): δ = −114.2 ppm.

MS (EI^+) *m/z* = 270 (M^+, 100). Data in agreement with literature.[21]

1,1-Bis(3-chlorophenyl)pentene (6u)

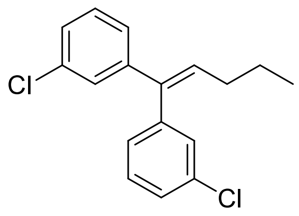

Butyltriphenylphosphonium bromide (1.00 g, 2.50 mmol), *n*-BuLi (1.6 M in hexane, 1.56 mL, 2.50 mmol) and 3,3'-dichlorobenzophenone (524 mg, 2.09 mmol) were reacted according to general olefination procedure to afford **6u** as a colourless oil (519 mg, 85%).

IR (film): 2955, 1591, 1562, 1472, 1078, 783, 716, 691 cm^{-1}.

^{1}H NMR (500 MHz, $CDCl_3$): δ = 7.33-7.31 (2H, m), 7.22-7.17 (4H, m), 7.08-7.04 (2H, m), 6.12 (1H, t, *J* = 8 Hz), 2.09 (2H, q, *J* = 8 Hz), 1.48 (2H, sext, *J* = 7 Hz), 0.92 (3H, t, *J* = 7 Hz) ppm; ^{13}C NMR (125 MHz, $CDCl_3$): δ = 144.1, 141.5, 139.4, 134.2 (2C), 132.2, 129.9, 129.6, 129.4, 128.1, 127.4, 127.1, 127.0, 125.4, 31.8, 22.9, 13.8 ppm.

MS (EI^+) *m/z* = 294 ([M(^{37}Cl,^{37}Cl)+Na]$^+$, 8), 292 ([M(^{37}Cl,^{35}Cl)+Na]$^+$, 42), 290 ([M(^{35}Cl,^{35}Cl)+Na]$^+$, 50); HRMS (EI^+) *m/z* calcd for $C_{17}H_{16}{}^{35}Cl_2$ $(M)^+$: 290.0629; found: 290.0629.

1,1-Bis(3-(trifluoromethyl)phenyl)pentene (6v)

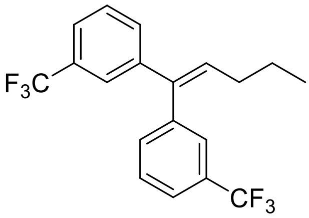

Butyltriphenylphosphonium bromide (1.00 g, 2.50 mmol), *n*-BuLi (1.75 M in hexane, 1.43 mL, 2.50 mmol) and 3,3'-bis(trifluoromethyl)benzophenone (524 mg, 2.09 mmol) were

reacted according to general olefination procedure to afford **6v** as a colourless oil (347 mg, 46%).

IR (film): 2965, 1319, 1161, 1111, 1069, 795, 706 cm^{-1}.

^{1}H NMR (400 MHz, $CDCl_3$): δ = 7.62 (1H, d, J = 8 Hz), 7.55-7.50 (3H, m), 7.46 (1H, s), 7.41-7.36 (2H, m), 7.31 (1H, d, J = 8 Hz), 6.22 (1H, t, J = 8 Hz), 2.11 (2H, q, J = 8 Hz), 1.56-1.47 (2H, sext, J = 8 Hz), 0.93 (3H, t, J = 8 Hz) ppm; ^{13}C NMR (125 MHz, $CDCl_3$): δ = 142.9 (2C), 140.2, 139.4, 133.3, 133.1, 131.0 (q, $J_{C\text{-}F}$ = 32 Hz), 130.9 (q, $J_{C\text{-}F}$ = 32 Hz), 128.9, 128.7, 126.6 (q, $J_{C\text{-}F}$ = 4 Hz), 124.4 (q, $J_{C\text{-}F}$ = 4 Hz), 124.1 (q, $J_{C\text{-}F}$ = 272 Hz), 124.0 (q, $J_{C\text{-}F}$ = 272 Hz), 123.8 (q, $J_{C\text{-}F}$ = 4 Hz), 123.6 (q, $J_{C\text{-}F}$ = 4 Hz), 31.8, 22.9, 13.8 ppm; ^{19}F NMR (282 MHz, $CDCl_3$): δ = −62.5 ($2CF_3$) ppm.

MS (EI^+) m/z = 358 (M^+, 100); HRMS ($APCI^+$) m/z calcd for $C_{19}H_{17}F_6$ $(M+H)^+$: 359.1229; found: 359.1226.

1,1-Bis(4-chlorophenyl)pentene (6w)

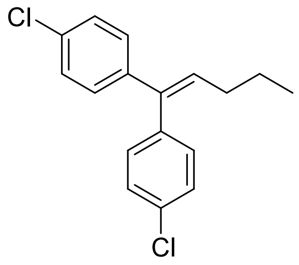

Butyltriphenylphosphonium bromide (1.02 g, 2.55 mmol), *n*-BuLi (1.7 M in hexane, 1.50 mL, 2.55 mmol) and 4,4'-dichlorobenzophenone (533 mg, 2.12 mmol) were reacted according to general olefination procedure to afford **6w** as a colourless oil (478 mg, 77%).

IR (film): 2959, 2864, 1491, 1402, 1088, 1013, 827, 815, 697 cm^{-1}.

^{1}H NMR (500 MHz, $CDCl_3$): δ = 7.37 (2H, d, J = 8 Hz), 7.24 (2H, d, J = 9 Hz), 7.14 (2H, d, J = 8 Hz), 7.11 (2H, d, J = 9 Hz), 6.10 (1H, t, J = 8 Hz), 2.10 (2H, q, J = 8 Hz), 1.48 (2H, sext, J = 7 Hz), 0.93 (3H, t, J = 7 Hz) ppm; ^{13}C NMR (125 MHz, $CDCl_3$): δ = 141.0, 139.6, 138.3, 133.0, 132.9, 131.2 (2C), 131.1, 128.5 (2C), 128.4 (2C), 128.3 (2C), 31.8, 23.0, 13.8 ppm.

MS (EI^+) m/z = 294 ($[M(^{37}Cl,^{37}Cl)+Na]^+$, 6), 292 ($[M(^{37}Cl,^{35}Cl)+Na]^+$, 36), 290 ($[M(^{35}Cl,^{35}Cl)+Na]^+$, 56); HRMS ($APCI^+$) m/z calcd for $C_{17}H_{17}{}^{35}Cl_2$ $(M+H)^+$: 291.0702; found: 291.0704.

1,1-Bis(4-bromophenyl)pentene (6x)

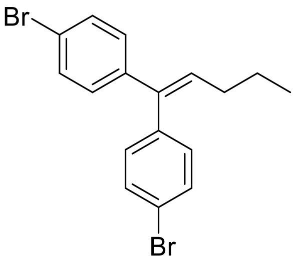

Butyltriphenylphosphonium bromide (1.00 g, 2.50 mmol), *n*-BuLi (1.6 M in hexane, 1.56 mL, 2.50 mmol) and 4,4'-dibromobenzophenone (710 mg, 2.09 mmol) were reacted according to general olefination procedure to afford **6x** as a colourless oil (630 mg, 79%).

IR (film): 2960, 1483, 1062, 1005, 816, 478 cm^{-1}.

^{1}H NMR (500 MHz, $CDCl_3$): δ = 7.51 (2H, d, *J* = 8 Hz), 7.39 (2H, d, *J* = 8 Hz), 7.08-7.03 (4H, m), 6.09 (1H, t, *J* = 8 Hz), 2.08 (2H, q, *J* = 8 Hz), 1.47 (2H, sext, *J* = 7 Hz), 0.91 (3H, t, *J* = 7 Hz) ppm; ^{13}C NMR (125 MHz, $CDCl_3$): δ = 141.3 (2C), 139.6, 138.7, 131.61 (2C), 131.5 (2C), 131.3 (2C), 128.8 (2C), 121.2, 121.0, 31.9, 23.0, 13.8 ppm.

MS (EI^+) *m/z* = 382 ([M(^{81}Br,^{81}Br)]$^+$, 25), 380 ([M(^{79}Br,^{81}Br)]$^+$, 50), 378 ([M(^{79}Br,^{79}Br)]$^+$, 25); HRMS ($APCI^+$) *m/z* calcd for $C_{17}H_{17}{}^{79}Br_2$ $(M+H)^+$: 378.9692; found: 378.9697.

1,1-Bis(4-methylphenyl)pentene (6y)

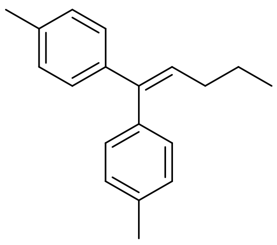

Butyltriphenylphosphonium bromide (1.00 g, 2.50 mmol), *n*-BuLi (2.45 M in hexane, 1.02 mL, 2.50 mmol) and 4,4'-dimethylbenzophenone (439 mg, 2.09 mmol) were reacted according to general olefination procedure to afford **6y** as a colourless oil (392 mg, 75%).

IR (film): 2955, 1491, 1458, 760, 698 cm^{-1}.

^{1}H NMR (500 MHz, $CDCl_3$): δ = 7.20-7.18 (2H, d, *J* = 8 Hz), 7.15 (2H, dd, *J* = 8, 2 Hz), 7.09 (4H, d, *J* = 8 Hz), 6.04 (1H, t, *J* = 7 Hz), 2.41 (3H, s), 2.34 (3H, s), 2.12 (2H, q, *J* =

7 Hz), 1.48 (2H, sext, J = 7 Hz), 0.93 (3H, t, J = 7 Hz) ppm; ^{13}C NMR (125 MHz, $CDCl_3$): δ = 141.3, 140.4, 137.6, 136.4, 136.3, 129.9 (2C), 129.1, 128.8 (2C), 128.7 (2C), 127.1 (2C), 31.8, 32.3, 21.2, 21.0, 13.9 ppm.

MS (EI$^+$) m/z = 250 (M$^+$, 100); HRMS (EI$^+$) m/z calcd for $C_{19}H_{22}$ (M$^+$): 250.1722; found: 250.1720.

1,1-Bis(4-fluorophenyl)pentene (6z)

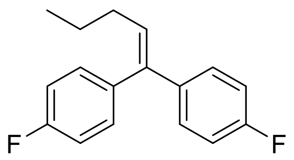

Butyltriphenylphosphonium bromide (1.313 g, 3.29 mmol), n-BuLi (2.1 M in hexane, 1.57 mL, 3.29 mmol) and 4,4'-fluorobenzophenone (600 mg, 2.75 mmol) were reacted according to general olefination procedure to afford **6z** as a colourless oil (447 mg, 63%).

IR (neat): 2958, 2873, 1658, 1600, 1508, 1276, 1230, 1157, 837, 767, 578 cm^{-1}.

^{1}H NMR (400 MHz, $CDCl_3$): δ = 7.03-7.17 (m, 6H), 6.92-6.97 (m, 2H), 6.01 (t, 1H, J = 7 Hz), 2.06 (q, 2H, J = 7 Hz), 1.46 (sext, 2H, J = 7 Hz), 0.89 (t, 3H, J = 7 Hz) ppm; ^{13}C NMR (100 MHz, $CDCl_3$): δ = 165.8 (d, J = 253 Hz), 134.1 (d, J = 3 Hz), 132.9 (d, J = 9 Hz), 128.4 (d, J = 8 Hz), 127.8 (d, J = 8 Hz), 116.0 (d, J = 22 Hz), 115.8 (d, J = 21 Hz), 115.4 (d, J = 21 Hz), 32.8, 19.9, 14.4 ppm.

HRMS (EI$^+$) m/z calcd for $C_{17}H_{16}F_2$ (M)$^+$: 258.1220; found: 258.1218.

1,1-Bis(2-chlorophenyl)pentene (6aa)

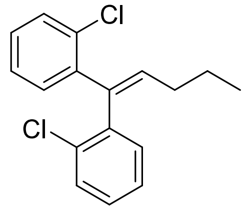

Butyltriphenylphosphonium bromide (1.00 g, 2.50 mmol), n-BuLi (1.75 M in hexane, 1.43 mL, 2.50 mmol) and 2,2'-dichlorobenzophenone (524 mg, 2.09 mmol) were reacted according to general olefination procedure to afford **6aa** as a colourless oil (343 mg, 47%).

IR (film): 2961, 1468, 1427, 1059, 1030, 748, 731 cm^{-1}.

^{1}H NMR (500 MHz, $CDCl_3$): δ = 7.43-7.36 (2H, m), 7.33-7.16 (6H, m), 6.04 (1H, t, *J* = 8 Hz), 2.09 (2H, app q, *J* = 7 Hz), 1.52 (2H, sext, *J* = 7 Hz), 0.95 (3H, t, *J* = 7 Hz) ppm; ^{13}C NMR (125 MHz, $CDCl_3$): δ = 141.0, 138.7, 137.2, 135.9, 133.8, 132.8, 132.2, 131.5, 130.0, 129.7, 128.4, 128.0, 126.3, 126.2, 31.8, 22.3, 13.8 ppm.

MS (EI$^+$) *m/z* = 294 ([M(^{37}Cl,^{37}Cl)+Na]$^+$, 6), 292 ([M(^{37}Cl,^{35}Cl)+Na]$^+$, 44), 290 ([M(^{35}Cl,^{35}Cl)+Na]$^+$, 50); HRMS (APCI$^+$) *m/z* calcd for $C_{17}H_{16}{}^{35}Cl_2$ (M)$^+$: 290.0624; found: 290.0624.

1,1-Bis(4-methoxyphenyl)pentene (6bb)

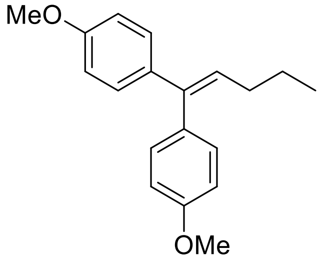

Butyltriphenylphosphonium bromide (1.00 g, 2.50 mmol), *n*-BuLi (2.5 M in hexane, 1.00 mL, 2.50 mmol) and 4,4'-dimethoxybenzophenone (506 mg, 2.09 mmol) were reacted according to general olefination procedure to afford **6bb** as a colourless oil (520 mg, 88%).

IR (film): 2953, 2829, 1508, 1238, 1176, 1031, 825 cm^{-1}.

^{1}H NMR (500 MHz, $CDCl_3$): δ = 7.18 (2H, d, *J* = 8 Hz), 7.12 (2H, d, *J* = 8 Hz), 6.93 (2H, d, *J* = 8 Hz), 6.82 (2H, d, *J* = 8 Hz), 5.97 (1H, t, *J* = 7 Hz), 3.86 (3H, s), 3.81 (3H, s), 2.12 (2H, q, *J* = 7 Hz), 1.48 (2H, sext, *J* = 7 Hz), 0.93 (3H, t, *J* = 7 Hz) ppm; ^{13}C NMR (125 MHz, $CDCl_3$): δ = 158.6, 158.4, 140.5, 136.1, 132.9, 131.1 (2C), 128.4 (2C), 128.3, 113.5 (2C), 113.4 (2C), 55.3, 55.2, 31.9, 23.4, 14.0 ppm.

MS (EI$^+$) *m/z* = 282 (M$^+$, 100); HRMS (EI$^+$) *m/z* calcd for $C_{19}H_{22}O_2$ (M$^+$): 282.1620; found: 282.1620.

Methyl 5,5-diphenyl-4-pentenoate (10)

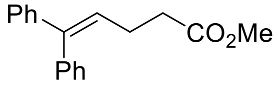

To the suspension of (3-carboxypropyl)triphenylphosphonium bromide (1.5 g, 3.49 mmol) in THF (20 mL) at 0 ºC was added dropwise *n*-BuLi (2.45 M in hexane, 2.85 mL, 6.98 mmol)

and the reaction stirred for 30 mins. Benzophenone (605 mg, 3.32 mmol) in THF (5 mL) was added dropwise. The reaction allowed to warm to room temperature and stirred until completion (tlc), then brought to pH 1 with aqueous HCl and extracted with diethyl ether (3×15 mL). Combined organic extracts were washed with brine (15 mL), dried ($MgSO_4$), filtered and concentrated under vacuum. The residue was dissolved in 2,2-dimethoxypropane (15 mL) and concentrated HCl (0.25 mL) was added dropwise. After 18 h the reaction was concentrated under vacuum. Column chromatography (hexane:ethyl acetate, 10:1 → 4:1) afforded **10** as a colourless oil (760 mg, 86%) which was stored under argon.

IR (film): 2941, 1736, 1441, 1155, 760, 694 cm^{-1}.

^{1}H NMR (500 MHz, $CDCl_3$): δ = 7.30 (2H, t, *J* = 8 Hz), 7.23 (1H, t, *J* = 7 Hz), 7.19-7.08 (7H, m), 5.97 (1H, t, *J* = 7 Hz), 3.58 (3H, s), 2.39-2.34 (4H, m) ppm; ^{13}C NMR (125 MHz, $CDCl_3$): δ = 173.3, 143.0, 142.4, 139.8, 129.8 (2C), 128.3 (2C), 128.1 (2C), 127.3 (3C), 127.1 (2C), 51.5, 34.3, 25.3 ppm.

MS (EI$^+$) *m/z* = 266 (M$^+$, 100); HRMS (EI$^+$) *m/z* calcd for $C_{18}H_{18}O_2$ (M)$^+$: 266.1307; found: 266.1308.

Asymmetric oxidative rearrangement

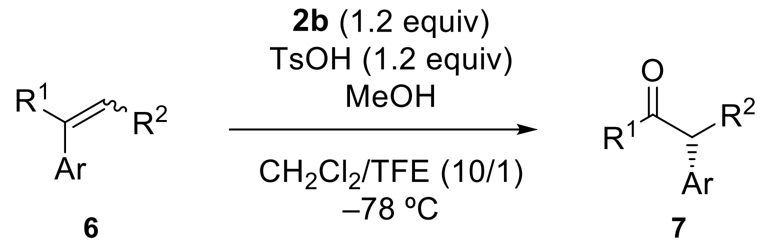

General procedure for the oxidative rearrangement:

To the solution of alkene **6** (0.09 mmol), reagent **2b** (57 mg, 0.11 mmol) and methanol (0.27 mmol or 0.54 mmol) in CH_2Cl_2:TFE (10:1 v/v) (1.5 mL) at –78 ºC was added the activating acid (0.11 mmol). The reaction was stirred until completion (tlc), then quenched with a 1:1 mixture of aqueous sat. $NaHCO_3$ and sat. $Na_2S_2O_3$ (0.5 mL). Water (4 mL) was added and the aqueous phase was extracted with CH_2Cl_2 (3×5 mL). The combined organic layers were filtered through a TELOS® Phase Separator and concentrated under vacuum to give the crude product. Column chromatography (hexane:ethyl acetate, 9:1 → 4:1) afforded ketone **7**.

Racemic ketones were prepared according following the above procedure with $PhI(OAc)_2$ in place of reagent **2b**.

(2*R*)-1,2-Diphenyl-1-pentanone (7a)

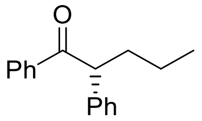

1,1-Diphenylpentene **6a** (20 mg, 0.09 mmol), reagent **2b** (57 mg, 0.11 mmol), TsOH•H_2O (20 mg, 0.11 mmol) and methanol (11 µL, 0.27 mmol) were reacted according to the general procedure at –78 ºC for 1.5 h to give **7a** as a colourless oil (19 mg, 87%).

IR (film): 2955, 1678, 1267, 1206, 750, 694 cm^{-1}.

^{1}H NMR (400 MHz, $CDCl_3$): δ = 7.89 (2H, d, *J* = 8 Hz), 7.41 (1H, t, *J* = 8 Hz), 7.31 (2H, t, *J* = 8 Hz), 7.25-7.18 (4H, m), 7.14-7.10 (1H, m), 4.49 (1H, t, *J* = 7 Hz), 2.13-2.04 (1H, m), 1.79-1.70 (1H, m), 1.30-1.12 (2H, m), 0.84 (3H, t, *J* = 7 Hz) ppm; ^{13}C NMR (75 MHz, $CDCl_3$): δ = 200.2, 139.8, 137.0, 132.8, 128.9 (2C), 128.7 (2C), 128.5 (2C), 128.3 (2C), 127.0, 53.4, 36.2, 20.9, 14.1 ppm.

MS (ES^+) *m/z* = 239 ($[M+H]^+$, 100); HRMS (EI^+) *m/z* calcd for $C_{17}H_{18}O$ $(M)^+$: 238.1358; found: 238.1358. Data in agreement with the literature.[22]

$[\alpha]_D^{20}$ = −150.0 (c = 1.0, $CHCl_3$); HPLC: YMC Chiral Amylose-C column, hexane/*i*-PrOH = 99.5/0.5, 1.0 mL/min, 10 ºC, 243 nm; t_R (*S*) = 9.4 min, t_R (*R*) = 12.2 min; 94% *ee*.

Table 1, entry 7, 94% *ee*

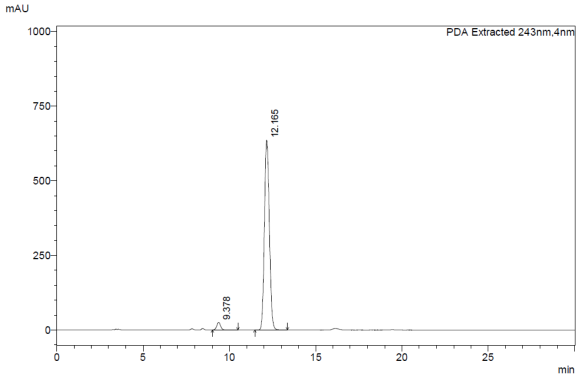

Peak#	Unit	Ret. Time	Area%
1		9.378	2.973
2		12.165	97.027
Total			100.000

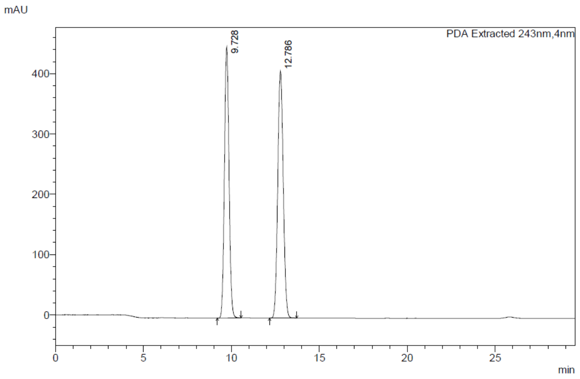

Peak#	Unit	Ret. Time	Area%
1		9.728	47.728
2		12.786	52.272
Total			100.0

Table 1, entry 9 (reagent 3), 83% *ee*

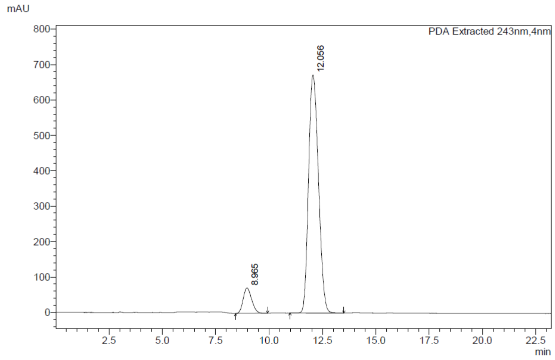

Peak#	Unit	Ret. Time	Area%
1		8.965	8.282
2		12.056	91.718
Total			100.000

Table 1, entry 8 (reagent 2a), 88% *ee*

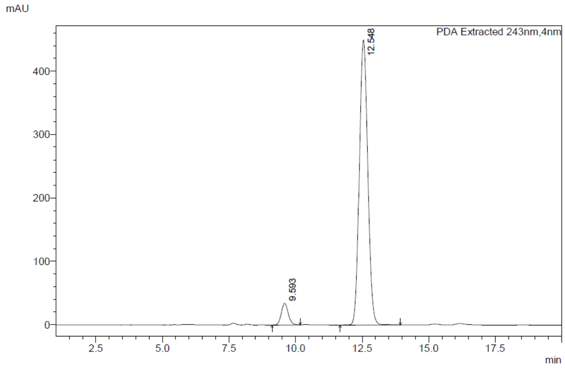

Peak#	Unit	Ret. Time	Area%
1		9.593	5.883
2		12.548	94.117
Total			100.000

Table 1, entry 11, 89% *ee*

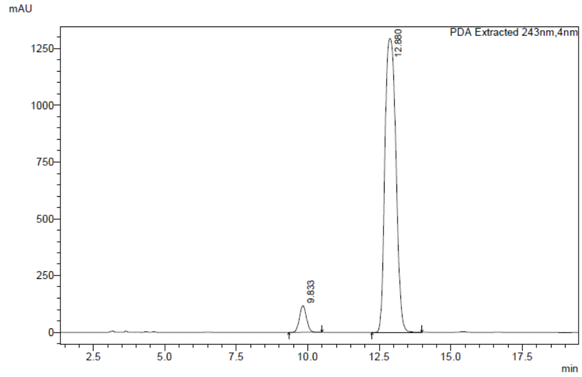

Peak#	Unit	Ret. Time	Area%
1		9.833	5.467
2		12.880	94.533
Total			100.000

(2*R*)-1,2-Diphenylpropan-1-one (7b)

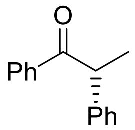

1,1-Diphenylpropene **6b** (36 mg, 0.19 mmol), reagent **2b** (117 mg, 0.22 mmol), TsOH•H_2O (42 mg, 0.22 mmol) and methanol (23 µL, 0.56 mmol) were reacted according to the general procedure at –78 ºC for 1.5 h to give **7b** as a colourless oil (35 mg, 90%).

IR (film): 2930, 1678, 1447, 1215, 953, 752, 691 cm^{-1}.

^{1}H NMR (400 MHz, $CDCl_3$): δ = 7.98 (2H, t, *J* = 7 Hz), 7.49 (1H, t, *J* = 8 Hz), 7.39 (2H, t, *J* = 8 Hz), 7.31-7.30 (4H, m), 7.23 (1H, app sext, *J* = 4 Hz), 4.71 (1H, q, *J* = 7 Hz), 1.56 (3H, d, *J* = 7 Hz) ppm; ^{13}C NMR (75 MHz, $CDCl_3$): δ = 200.4, 141.5, 136.5, 132.8, 129.0 (2C), 128.8 (2C), 128.5 (2C), 127.8 (2C), 126.9, 47.9, 19.6 ppm.

MS (EI^+) *m/z* = 210 (M^+, 100); HRMS (ES^+) *m/z* calcd for $C_{15}H_{15}O$ $(M+H)^+$: 211.1117; found: 211.1117. Data in agreement with the literature.[23]

$[\alpha]_D^{20}$ = −189.0 (c = 1.0, $CHCl_3$); HPLC: YMC Chiral Amylose-C column, hexane/*i*-PrOH = 98/2, 1.0 mL/min, 10 ºC, 276 nm; t_R (*S*) = 7.5 min, t_R (*R*) = 8.3 min; 91% *ee*.

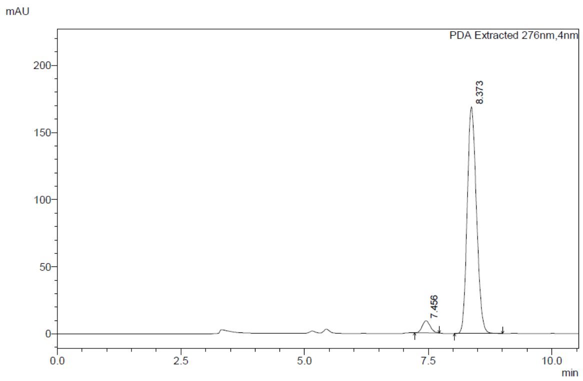

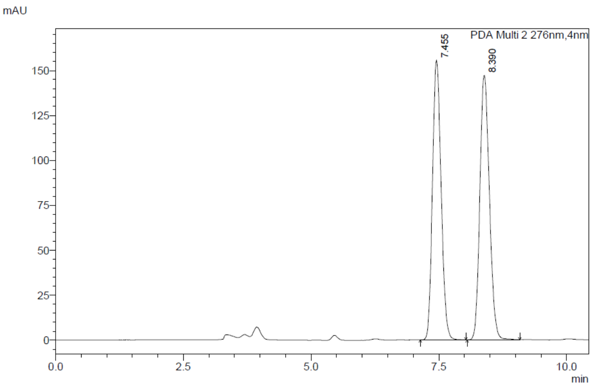

Peak#	Unit	Ret. Time	Area%
1		7.456	4.434
2		8.373	95.566
Total			100.000

Peak#	Unit	Ret. Time	Area%
1		7.455	48.846
2		8.390	51.154
Total			100.000

(2*R*)-3-Methyl-1,2-diphenylbutan-1-one (7c)

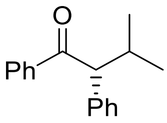

3-Methyl-1,1-diphenyl-1-butene **6c** (19 mg, 0.09 mmol), reagent **2b** (54 mg, 0.10 mmol), TsOH•H_2O (20 mg, 0.10 mmol) and methanol (10 µL, 0.26 mmol) were reacted according to the general procedure at –78 ºC for 6 h to give **7c** as a colourless solid (18 mg, 88%). Mp. 81-82 ºC.

IR (film): 2959, 1678, 1446, 1207, 1007, 745, 692 cm^{-1}.

^{1}H NMR (500 MHz, $CDCl_3$): δ = 7.99 (2H, d, J = 7 Hz), 7.50 (1H, t, J = 7 Hz), 7.41 (2H, t, J = 8 Hz), 7.35 (2H, d, J = 7 Hz), 7.29 (2H, t, J = 7 Hz), 7.20 (1H, t, J = 7 Hz), 4.22 (1H, d, J = 10 Hz), 2.65-2.55 (1H, m), 1.02 (3H, d, J = 6 Hz), 0.77 (3H, d, J = 7 Hz) ppm; ^{13}C NMR (75 MHz, $CDCl_3$): δ = 200.7, 138.6, 137.6, 132.8, 128.8 (2C), 128.7 (2C), 128.5 (4C), 127.0, 61.4, 31.9, 22.0, 20.6 ppm.

MS (ES$^+$) m/z = 239 ([M+H]$^+$, 100); HRMS (ES$^+$) m/z calcd for $C_{17}H_{19}O$ (M+H)$^+$: 239.1430; found: 239.1432.

$[\alpha]_D^{20}$ = –64.0 (c = 1.0, $CHCl_3$); HPLC: Daicel Chiralcel AD column (5 cm), hexane/*i*-PrOH = 99.5/0.5, 0.5 mL/min, 246 nm; 10 ºC, t_R (*S*) = 3.0 min, t_R (*R*) = 4.3 min; 39% *ee*.

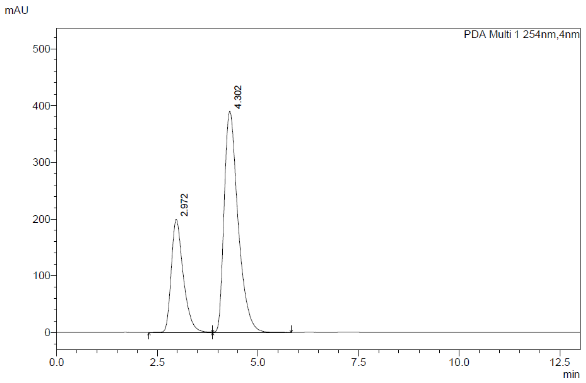

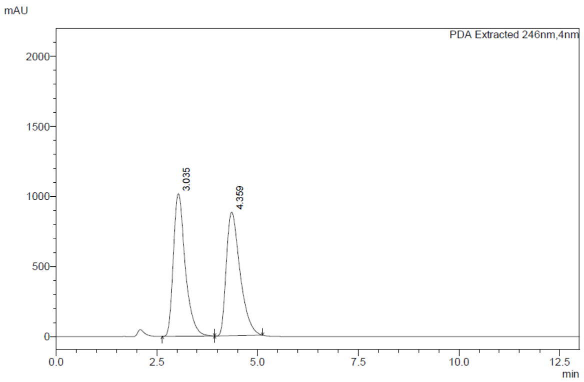

Peak#	Unit	Ret. Time	Area%
1		2.972	30.740
2		4.302	69.260
Total			100.000

Peak#	Unit	Ret. Time	Area%
1		3.035	49.808
2		4.359	50.192
Total			100.000

(2*R*)-4-Methoxy-1,2-diphenylbutan-1-one (7d)

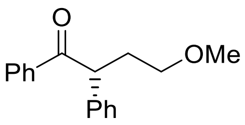

4-Methoxy-1,1-diphenyl-1-butene **6d** (26 mg, 0.11 mmol), reagent **2b** (68 mg, 0.13 mmol), TsOH•H_2O (25 mg, 0.13 mmol) and methanol (13 µL, 0.33 mmol) were reacted according to the general procedure at –78 ºC for 2 h to give **7d** as a colourless oil (25 mg, 90%).

IR (film): 2924, 1674, 1442, 1267, 1115, 756, 696 cm^{-1}.

^{1}H NMR (300 MHz, CDCl$_3$): δ = 7.90 (2H, t, J = 7 Hz), 7.40 (1H, t, J = 7 Hz), 7.30 (2H, t, J = 7 Hz), 7.25-7.19 (4H, m), 7.15-7.10 (1H, m), 4.77 (1H, t, J = 7 Hz), 3.33-3.27 (1H, m), 3.23-3.15 (4H, m), 2.44-2.33 (1H, m), 2.03-1.93 (1H, m) ppm; ^{13}C NMR (75 MHz, CDCl$_3$): δ = 199.9, 139.1, 136.8, 132.9, 129.0 (2C), 128.8 (2C), 128.5 (2C), 128.4 (2C), 127.1, 69.9, 58.6, 49.7, 33.7 ppm.

MS (EI$^+$) m/z = 254 (M$^+$, 100); HRMS (ES$^+$) m/z calcd for $C_{17}H_{19}O_2$ (M+H)$^+$: 255.1380; found: 255.1378.

$[\alpha]_D^{20}$ = –20.0 (c = 1.0, CHCl$_3$); HPLC: Daicel Chiralcel AD column (5 cm), hexane/*i*-PrOH = 99.5/0.5, 0.3 mL/min, 10 ºC, 249 nm; t$_R$ (*S*) = 15.7 min, t$_R$ (*R*) = 20.4 min; 91% *ee*.

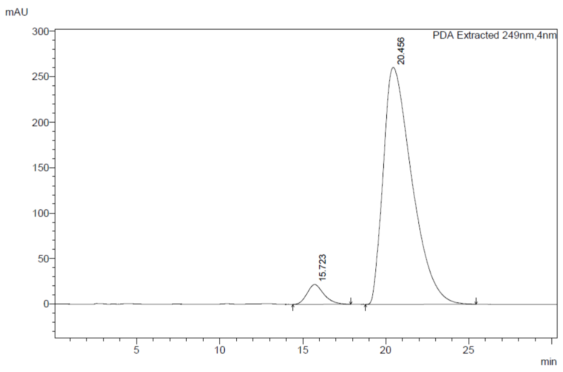

Peak#	Unit	Ret. Time	Area%
1		15.723	4.674
2		20.456	95.326
Total			100.000

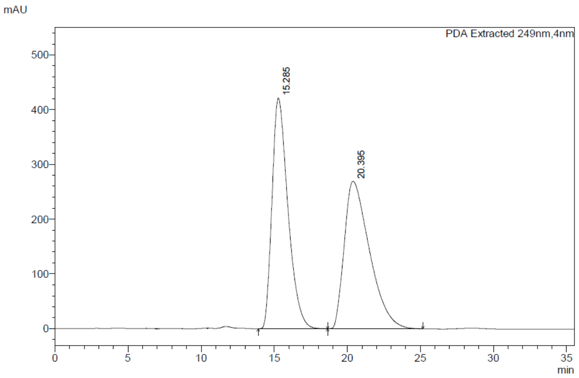

Peak#	Unit	Ret. Time	Area%
1		15.285	49.653
2		20.395	50.347
Total			100.000

Ethyl 3-oxo-2,3-diphenylpropanoate (7e)

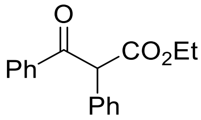

Ethyl-3,3-diphenylacrylate **6e** (25 mg, 0.10 mmol), reagent **2b** (63 mg, 0.12 mmol), TMSOTf (22 μL, 0.12 mmol) and methanol (24 μL, 0.59 mmol) were reacted according to the general procedure at –78 ºC for 4.5 h to give **7e** as a colourless oil (18 mg, 68%). Mp. 87-88 ºC.

IR (neat): 2974, 1734, 1672, 1261, 1179, 1157, 698 cm^{-1}.

^{1}H NMR (400 MHz, CDCl$_3$): δ = 7.89 (2H, t, J = 7 Hz), 7.46 (1H, t, J = 7 Hz), 7.37-7.18 (7H, m), 5.54 (1H, s), 4.21-4.09 (2H, m), 1.17 (3H, t, J = 7 Hz) ppm; ^{13}C NMR (125

MHz, $CDCl_3$): δ = 193.3, 168.8, 135.8, 133.5, 133.0, 129.6 (2C), 128.9 (2C), 128.8 (2C), 128.7 (2C), 128.1, 61.7, 60.6, 14.0 ppm.

MS (EI^+) *m/z* = 268 (M^+, 100); HRMS (EI^+) *m/z* calcd for $C_{17}H_{16}O_3$ $(M)^+$: 268.1099; found: 268.1100.

$[\alpha]_D^{20}$ = 0.0 (c = 1.0, $CHCl_3$). Data in agreement with the literature.[24]

(2*S*)-(*N*-Benzyloxycarbonyl)-3-oxo-2,3-diphenylpropane-1-amine (7f)

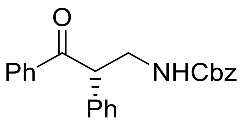

(*N*-Benzyloxycarbonyl)-3,3-diphenyl-2-propene-1-amine **6f** (30 mg, 0.09 mmol), reagent **2b** (55 mg, 0.10 mmol), TsOH•H_2O (20 mg, 0.10 mmol) and methanol (11 μL, 0.26 mmol) were reacted according to the general procedure at –78 ºC for 4 h to give **7f** as a colourless oil (28 mg, 89%).

IR (neat): 3338, 3030, 2940, 1705, 1670, 1508, 1448, 1213, 750, 687 cm^{-1}.

^1H NMR (400 MHz, $CDCl_3$): δ = 7.84 (2H, d, *J* = 8 Hz), 7.40 (1H, t, *J* = 8 Hz), 7.30-7.14 (12H, m), 5.14 (1H, br t, *J* = 5 Hz), 5.04-4.95 (2H, m), 4.83-4.80 (1H, m), 3.72-3.66 (1H, m), 3.63-3.57 (1H, m) ppm; ^{13}C NMR (125 MHz, $CDCl_3$): δ = 199.1, 156.4, 136.8, 136.5, 136.2, 133.2, 129.2 (2C), 128.9 (2C), 128.6 (2C), 128.5 (2C), 128.3 (2C), 128.1, 128.0 (2C), 127.6, 66.7, 54.0, 44.2 ppm.

MS (ES^+) *m/z* = 382 ($[M+Na]^+$, 100); HRMS (EI^+) *m/z* calcd for $C_{23}H_{21}NO_3$ $(M)^+$: 359.1521; found: 359.1519.

$[\alpha]_D^{20}$ = –100.0 (c = 1.0, $CHCl_3$); HPLC: YMC Chiral Amylose-C column column, hexane/*i*-PrOH = 96/4, 1.0 mL/min, 10 ºC, 257 nm; t_R (*R*) = 7.9 min, t_R (*S*) = 9.0 min; 83% *ee*.

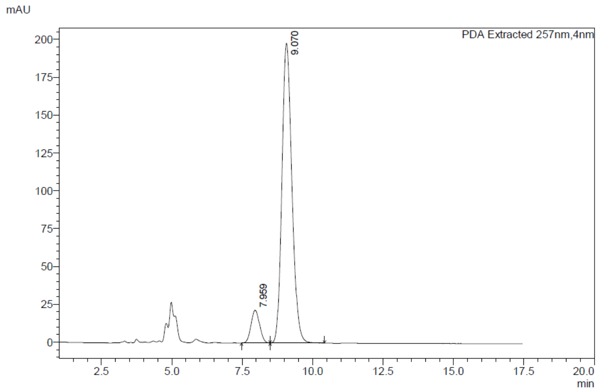

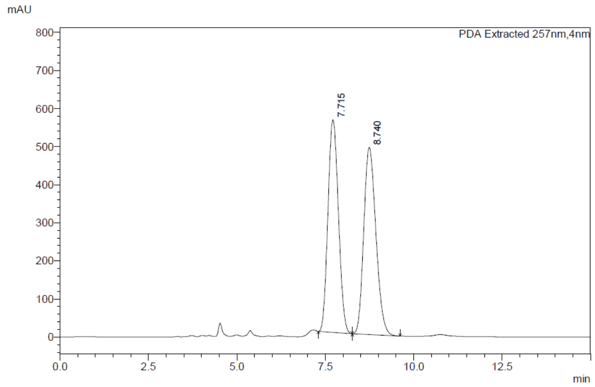

Peak#	Unit	Ret. Time	Area%
1		7.959	8.584
2		9.070	91.416
Total			100.000

Peak#	Unit	Ret. Time	Area%
1		7.715	49.482
2		8.740	50.518
Total			100.000

(2*S*)-3-Azido-1,2-diphenylpropan-1-one (7g)

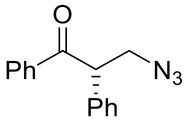

3-Azido-1,1-diphenyl-1-propene **6g** (17 mg, 0.07 mmol), reagent **2b** (46 mg, 0.09 mmol), TsOH•H_2O (16 mg, 0.09 mmol) and methanol (9 μL, 0.22 mmol) were reacted according to the general procedure at –78 ºC for 3 h to give **7g** as a colourless oil (15 mg, 83%).

IR (neat): 2975, 2095, 1676, 1447, 1279, 746 cm^{-1}.

^{1}H NMR (500 MHz, $CDCl_3$): δ = 7.88 (2H, d, *J* = 7 Hz), 7.42 (1H, t, *J* = 7 Hz), 7.32 (2H, t, *J* = 8 Hz), 7.28-7.17 (5H, m), 4.69 (1H, dd, *J* = 9, 4 Hz), 4.04 (1H, dd, *J* = 9, 4 Hz), 3.51 (1H, app dd, *J* = 6, 6 Hz) ppm; ^{13}C NMR (125 MHz, $CDCl_3$): δ = 197.5, 136.2, 135.9, 133.4, 129.4 (2C), 128.9 (2C), 128.7 (2C), 128.3 (2C), 128.1, 53.9, 53.6 ppm.

HRMS ($APCI^{+}$) *m/z* calcd for $C_{15}H_{14}N_3O$ $(M+H)^{+}$: 252.1131; found: 252.1135.

$[\alpha]_D^{20}$ = –150.0 (c = 1.0, $CHCl_3$); HPLC: YMC Chiral Amylose-C column, hexane/*i*-PrOH = 99.5/0.5, 1.0 mL/min, 10 ºC, 245 nm; t_R (*S*) = 15.9 min, t_R (*R*) = 20.1 min; 89% *ee*.

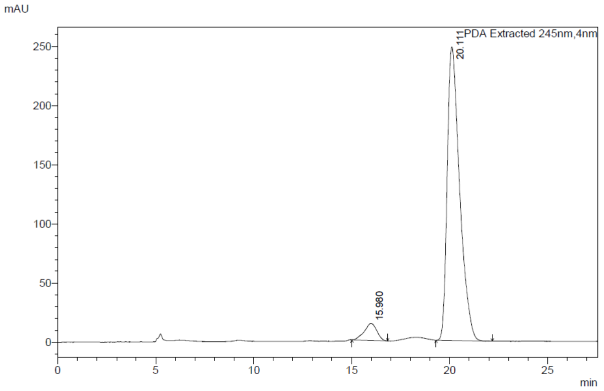

Peak#	Unit	Ret. Time	Area%
1		15.980	5.498
2		20.111	94.502
Total			100.000

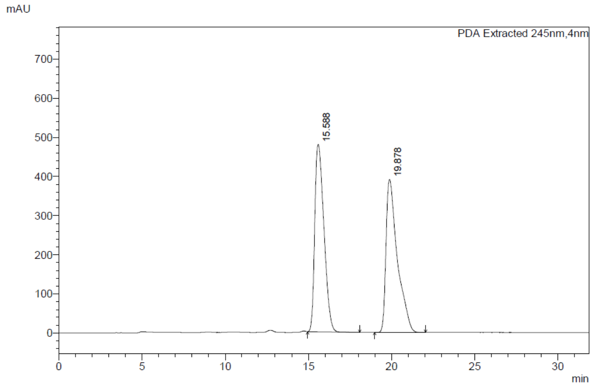

Peak#	Unit	Ret. Time	Area%
1		15.588	49.753
2		19.878	50.247
Total			100.000

(2*R*)-3-(1,3-Dioxan-2-yl)-1,2-diphenylpropan-1-one (7h)

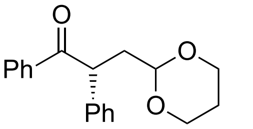

2-(3,3-Diphenylallyl)-1,3-dioxane **6h** (50 mg, 0.18 mmol), reagent **2b** (115 mg, 0.22 mmol), $TsOH•H_2O$ (42 mg, 0.22 mmol) and methanol (22 μL, 0.54 mmol) were reacted according to the general procedure at –78 ºC for 8 h to give **7h** as a colourless oil (28 mg, 54%).

IR (neat): 2924, 2850, 1681, 1597, 1450, 1211, 1107, 1006, 702 cm^{-1}.

^1H NMR (400 MHz, $CDCl_3$): δ = 7.91-7.94 (m, 2H), 7.21-7.45 (m, 7H), 7.13-7.17 (m, 1H), 4.83 (t, 1H, J = 7 Hz), 4.37 (t, 1H, J = 6 Hz), 4.06 (dq, 1H, J = 5 Hz, J = 12 Hz), 3.93 (q, 1H, J = 5 Hz), 3.58-3.69 (m, 2H), 2.46-2.53 (m, 1H), 1.97-2.06 (m, 2H), 1.21-1.26 (m, 1H) ppm; ^{13}C NMR (75 MHz, $CDCl_3$): δ = 199.4, 139.5, 136.7, 132.8, 129.0, 128.8, 128.5, 128.3, 127.1, 100.2, 66.8 (2C), 48.2, 38.9, 25.8 ppm.

HRMS (EI^+) *m/z* calcd for $C_{23}H_{22}O_2$ $(M)^+$: 296.1412; found: 296.1418.

$[\alpha]_D^{20}$ = −85.0 (c = 1.0, $CHCl_3$); HPLC: YMC Chiral Amylose-C column, hexane/*i*-PrOH = 95.0/5.0, 1.0 mL/min, 10 ºC, 239 nm; t_R (*S*) = 20.8 min, t_R (*R*) = 31.3 min; 69% *ee*.

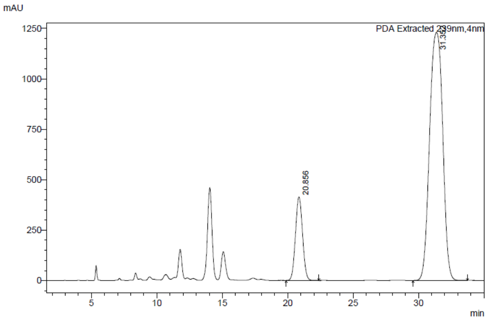

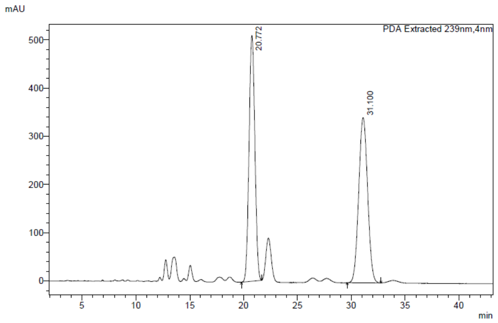

Peak#	Unit	Ret. Time	Area%
1		20.856	15.412
2		31.352	84.588
Total			100.000

Peak#	Unit	Ret. Time	Area%
1		20.772	49.382
2		31.100	50.618
Total			100.000

(2*R*)-4-(Benzyloxy)-1,2-diphenylbutan-1-one (7i)

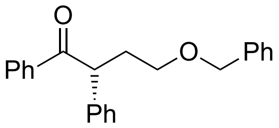

4-Benyloxy-1,1-diphenyl-pentene **6i** (31 mg, 0.10 mmol), reagent **2b** (63 mg, 0.12 mmol), TsOH•H_2O (23 mg, 0.12 mmol) and methanol (12 μL, 0.30 mmol) were reacted according to the general procedure at –78 ºC for 8 h to give **7i** as a colourless oil (17 mg, 53%).

IR (neat): 3028, 2924, 2854, 1681, 1597, 1450, 1269, 1107, 736, 702 cm^{-1}.

^1H NMR (400 MHz, $CDCl_3$): δ = 7.92-7.94 (m, 2H), 7.43-7.46 (m, 1H), 7.33-7.36 (m, 2H), 7.23-7.28 (m, 9H), 7.15-7.18 (m, 1H), 4.86 (t, 1H, J = 6 Hz), 4.41 (s, 2H), 3.44-3.48 (m, 1H), 3.33-3.38 (m, 1H), 2.45-2.52 (m, 1H), 2.04-2.10 (m, 1H) ppm; ^{13}C NMR (100 MHz, $CDCl_3$): δ = 200.3, 139.6, 138.8, 137.2, 133.2, 129.3, 129.2, 129.0, 128.8, 128.7, 128.1, 128.0, 127.5, 73.4, 67.9, 50.1, 34.2 ppm.

HRMS (EI^+) *m/z* calcd for $C_{23}H_{22}O_2$ $(M)^+$: 330.1620; found: 330.1621.

$[\alpha]_D^{20}$ = −32.0 (c = 1.0, $CHCl_3$); HPLC: YMC Chiral Amylose-C column, hexane/*i*-PrOH = 99.0/1.0, 1.0 mL/min, 10 ºC, 232 nm; t_R (*S*) = 18.7 min, t_R (*R*) = 19.8 min; 92% *ee*.

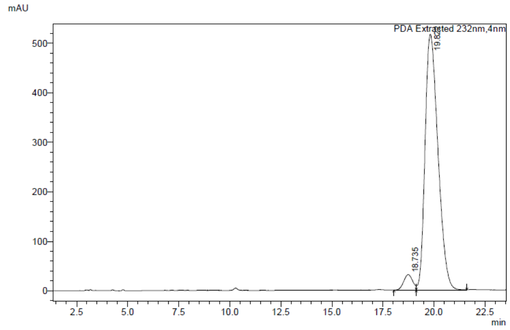

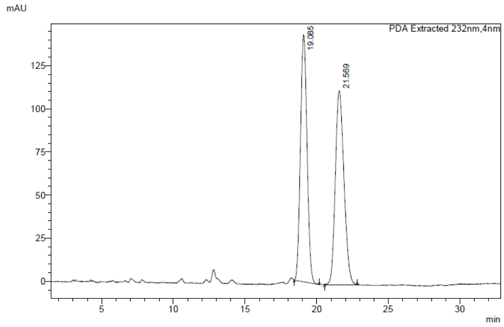

Peak#	Unit	Ret. Time	Area%
1		18.735	4.038
2		19.822	95.962
Total			100.000

Peak#	Unit	Ret. Time	Area%
1		19.085	49.266
2		21.569	50.734
Total			100.000

(4*R*)-2-Methyl-4-phenylheptan-3-one (7j)

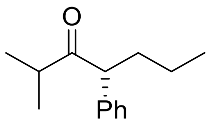

2-Methyl-3-phenyl-4-heptene **6j** (18 mg, 0.10 mmol), reagent **2b** (60 mg, 0.11 mmol), TsOH•H_2O (22 mg, 0.11 mmol) and methanol (12 μL, 0.29 mmol) were reacted according to the general procedure at –78 ºC for 4.5 h to give **7j** as a colourless oil (17 mg, 89%).

IR (film): 2959, 2872, 1709, 1455, 1032, 758, 700 cm^{-1}.

^{1}H NMR (400 MHz, $CDCl_3$): δ = 7.25-7.13 (5H, m), 3.72 (1H, t, *J* = 7 Hz), 2.58 (1H, sept, *J* = 7 Hz), 1.96-1.87 (1H, m), 1.61-1.52 (1H, m), 1.19-1.04 (2H, m), 1.00 (3H, d, *J* = 7 Hz), 0.84-0.79 (6H, m) ppm; ^{13}C NMR (125 MHz, $CDCl_3$): δ = 214.2, 139.4, 128.7 (2C), 128.3 (2C), 127.0, 57.0, 39.9, 35.0, 20.8, 18.9, 18.1, 14.0 ppm.

MS (EI^+) *m/z* = 204 (M^+, 100); HRMS (EI^+) *m/z* calcd for $C_{14}H_{20}O$ $(M)^+$: 204.1514; found: 204.1514.

$[\alpha]_D^{20}$ = –243.0 (c = 1.0, $CHCl_3$); HPLC: Daicel Chiralcel OD-H column, hexane/*i*-PrOH = 99.75/0.25, 1.0 mL/min, 10 ºC, 223 nm; t_R (*S*) = 4.6 min, t_R (*R*) = 4.9 min; 80% *ee*.

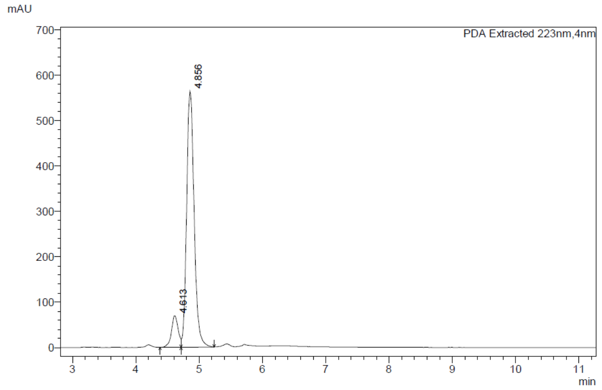

Peak#	Unit	Ret. Time	Area%
1		4.613	10.056
2		4.856	89.944
Total			100.000

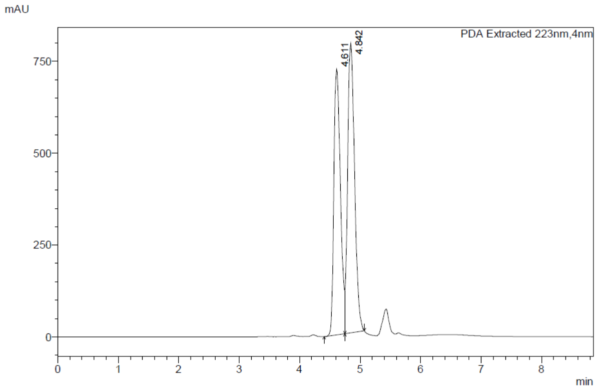

Peak#	Unit	Ret. Time	Area%
1		4.611	46.716
2		4.842	53.284
Total			100.000

(2*R*)-2-Phenylpentan-3-one (7k)

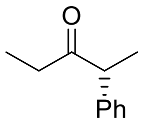

3-Phenyl-2-pentene **6k** (13 mg, 0.09 mmol), reagent **2b** (56 mg, 0.11 mmol), TsOH•H_2O (22 mg, 0.11 mmol) and methanol (11 µL, 0.27 mmol) were reacted according to the general procedure at –78 ºC for 2 h to give **7k** as a colourless oil (12 mg, 83%).

IR (film): 2974, 2936, 1713, 1456, 758, 698 cm^{-1}.

^{1}H NMR (400 MHz, $CDCl_3$): δ = 7.28-7.23 (2H, m), 7.20-7.12 (3H, m), 3.69 (1H, q, *J* = 7 Hz), 2.39-2.22 (2H, m), 1.32 (3H, d, *J* = 7 Hz), 0.89 (3H, t, *J* = 7 Hz) ppm; ^{13}C NMR (125 MHz, $CDCl_3$): δ = 211.4, 140.9, 128.9 (2C), 127.8 (2C), 127.0, 52.7, 34.2, 17.5, 8.0 ppm.

MS (EI^+) *m/z* = 162 (M^+, 100); HRMS (ES^+) *m/z* calcd for $C_{11}H_{14}O$ $(M+H)^+$: 163.1117; found: 163.1115. Data in agreement with the literature.[25]

$[\alpha]_D^{20}$ = –111.1 (c = 1.0, $CHCl_3$); HPLC: Daicel Chiralcel OJ column, hexane/*i*-PrOH = 99.5/0.5, 1.0 mL/min, 10 ºC, 211 nm; t_R (*S*) = 10.9 min, t_R (*R*) = 14.0 min; 43% *ee*.

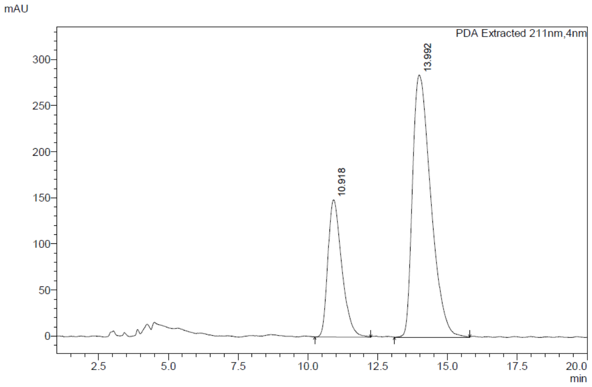

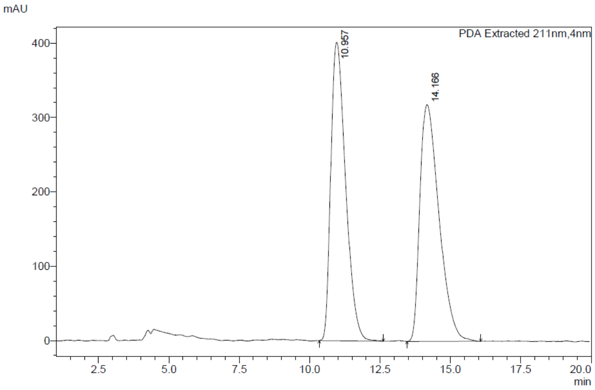

Peak#	Unit	Ret. Time	Area%
1		10.918	28.449
2		13.992	71.551
Total			100.000

Peak#	Unit	Ret. Time	Area%
1		10.957	49.351
2		14.166	50.649
Total			100.000

(3*R*)-3-(3-Bromophenyl)hexan-2-one (7l)

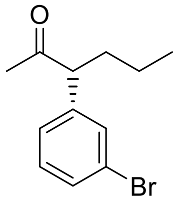

2-(3-Bromophenyl)-hex-2-ene **6l** (21 mg, 0.09 mmol), reagent **2b** (55 mg, 0.11 mmol), TsOH•H_2O (20 mg, 0.11 mmol) and methanol (11 µL, 0.26 mmol) were reacted according to the general procedure at –78 ºC for 3 h to give **7l** as a colourless oil (18 mg, 80%).

IR (film): 2963, 2158, 1713, 1354, 1161, 779, 698 cm^{-1}.

^{1}H NMR (400 MHz, $CDCl_3$): δ = 7.42-7.37 (2H, m), 7.21(1H, t, *J* = 8 Hz), 7.16-7.14 (1H, m), 3.59 (1H, t, *J* = 7 Hz), 2.08 (3H, s), 2.04-1.92 (1H, m), 1.70-1.61 (1H, m), 1.27- 1.13 (2H, m), 0.89 (3H, t, *J* = 7 Hz) ppm; ^{13}C NMR (125 MHz, $CDCl_3$): δ = 207.7, 141.4, 131.3, 130.4 (2C), 126.8, 122.9, 59.1, 34.0, 29.1, 20.6, 13.9 ppm.

MS (EI^+) *m/z* = 256 [M(^{81}Br)$^+$, 50], 254 [M(^{79}Br)$^+$, 50]; HRMS (ES^+) *m/z* calcd for $C_{12}H_{16}O^{79}Br$ $(M+H)^+$: 255.0379; found: 255.0380.

$[\alpha]_D^{20}$ = –20.0 (c = 1.0, $CHCl_3$); HPLC: Daicel Chiralcel OJ column, hexane/*i*-PrOH = 99.5/0.5, 1.0 mL/min, 10 ºC, 227 nm; t_R (*S*) = 8.1 min, t_R (*R*) = 9.0 min; 17% *ee*.

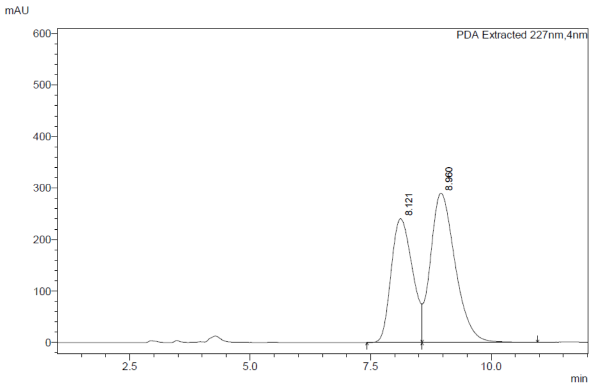

Peak#	Unit	Ret. Time	Area%
1		8.121	41.688
2		8.960	58.312
Total			100.000

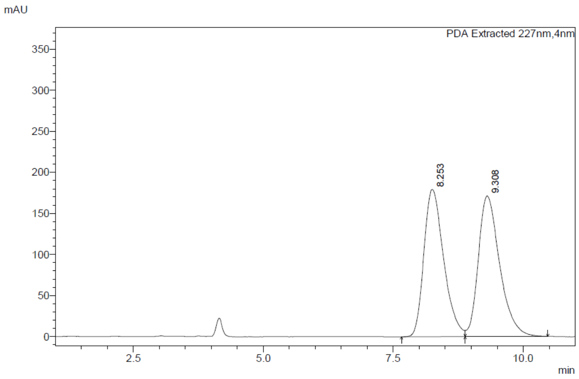

Peak#	Unit	Ret. Time	Area%
1		8.253	49.164
2		9.308	50.836
Total			100.000

(3*R*)-3-Phenylhexan-2-one (7m)

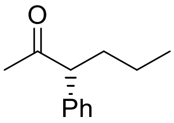

2-Phenyl-2-hexene **6m** (27 mg, 0.17 mmol), reagent **2b** (106 mg, 0.20 mmol), TsOH•H_2O (38 mg, 0.20 mmol) and methanol (20 µL, 0.51 mmol) were reacted according to the general procedure at –78 ºC for 1 h to give **7m** as a colourless oil (21 mg, 71%).

IR (film): 2956, 2931, 1709, 1350, 1159, 700, 547 cm^{-1}.

^{1}H NMR (300 MHz, $CDCl_3$): δ = 7.29-7.12 (5H, m), 3.55 (1H, t, *J* = 7 Hz), 1.98-1.87 (4H, m), 1.67-1.57 (1H, m), 1.20-1.07 (2H, m), 0.82 (3H, t, *J* = 7 Hz) ppm; ^{13}C NMR (75 MHz, $CDCl_3$): δ = 208.8, 139.1, 128.9 (2C), 128.3 (2C), 127.2, 59.5, 33.9, 29.1, 20.6, 14.0 ppm.

MS (ES$^+$) *m/z* = 375 ([2M+Na]$^+$, 100); HRMS (ES$^+$) *m/z* calcd for $C_{12}H_{17}O$ (M+H)$^+$: 177.1274; found: 177.1271.

$[\alpha]_D^{20}$ = +28.5 (c = 1.0, $CHCl_3$). Enantiomers inseparable by HPLC on Diacel Chiralpak AD-H, OD-H, OB-H and OJ columns.

(2*R*)-1-Cyclopentyl-2-phenylpropan-1-one (7n)

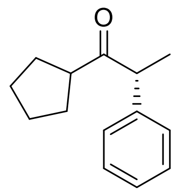

1-Cyclopentyl-1-phenylpropene **6n** (34 mg, 0.18 mmol), reagent **2b** (115 mg, 0.22 mmol), TsOH•H_2O (42 mg, 0.22 mmol) and methanol (22 μL, 0.54 mmol) were reacted according to the general procedure at –78 ºC for 9 h to give **7n** as a colourless oil (28 mg, 78%).

IR (neat): 2862, 1705, 1597, 1496, 1450, 1257, 1165, 1072, 1026, 802, 732, 702 cm^{-1}.

^{1}H NMR (400 MHz, $CDCl_3$): δ = 7.23-7.26 (m, 2H), 7.13-7.19 (m, 3H), 3.78 (q, 1H, *J* = 6 Hz), 2.78-2.85 (m, 1H), 1.71-1.81 (m, 1H), 1.35-1.65 (m, 8H), 1.31 (d, 3H, *J* = 6 Hz) ppm; ^{13}C NMR (100 MHz, $CDCl_3$): δ = 214.1, 141.2, 129.2, 128.5, 127.4, 53.0, 50.3, 30.9, 29.5, 26.5, 18.4 ppm.

HRMS (EI^+) *m/z* calcd for $C_{14}H_{18}O$ $(M)^+$: 202.1358; found: 202.1360.

$[\alpha]_D^{20}$ = −183.0 (c = 1.0, $CHCl_3$); HPLC: YMC Chiral Amylose-C column, hexane/*i*-PrOH = 99.0/1.0, 1.0 mL/min, 10 ºC, 229 nm; t_R (*S*) = 5.6 min, t_R (*R*) = 6.0 min; 87% *ee*.

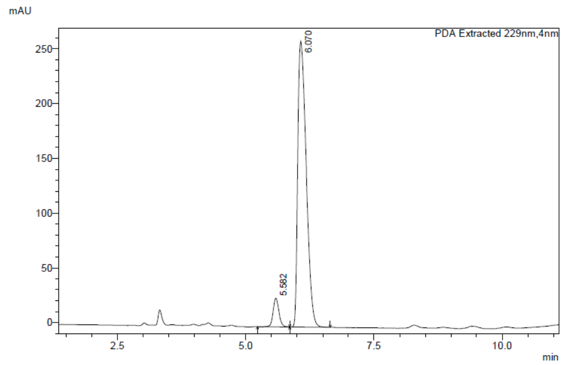

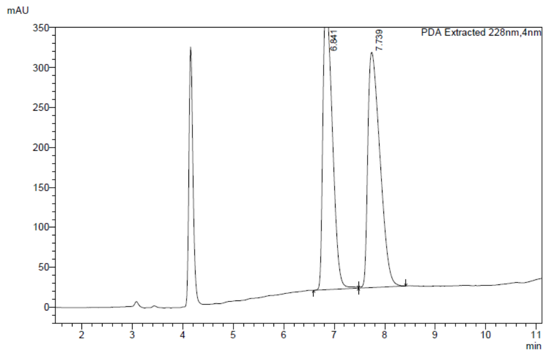

Peak#	Unit	Ret. Time	Area%
1		5.582	6.517
2		6.070	93.483
Total			100.000

Peak#	Unit	Ret. Time	Area%
1		6.841	48.771
2		7.739	51.229
Total			100.000

(2*R*)-2-(4-Anisyl)-1-phenylpropan-1-one (7o)

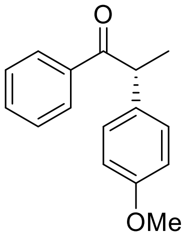

1-(4-Anisyl)-1-phenylpropene **6** (60 mg, 0.27 mmol), reagent **2b** (173 mg, 0.33 mmol), TsOH•H_2O (63 mg, 0.33 mmol) and methanol (33 µL, 0.81 mmol) were reacted according to the general procedure at –78 ºC for 6 h to give **7** as a colourless oil (24 mg, 38%).

^{1}H NMR (400 MHz, $CDCl_3$): δ = 7.93-7.95 (m, 2H), 7.45-7.48 (m, 1H), 7.35-7.39 (m, 2H), 7.18-7.20 (m, 2H), 6.80-6.83 (m, 2H), 4.63 (q, 1H, *J* = 6 Hz), 3.75 (s, 3H, OCH_3), 1.50 (d, 2H, *J* = 6 Hz) ppm; ^{13}C NMR (100 MHz, $CDCl_3$): δ = 200.7, 158.5, 136.5, 133.5, 132.6, 128.8, 128.7, 128.5, 114.4, 55.2, 46.9, 19.5 ppm. Data in agreement with literature.[23]

HRMS (EI^+) *m/z* calcd for $C_{16}H_{16}O_2$ $(M)^+$: 240.1150; found: 240.1149.

$[\alpha]_D^{20}$ = −126.0 (c = 1.0, $CHCl_3$); HPLC: YMC Chiral Amylose-C column, hexane/*i*-PrOH = 99.0/1.0, 1.0 mL/min, 10 ºC, 229 nm; t_R (*S*) = 17.8 min, t_R (*R*) = 25.3 min; 77% *ee*.

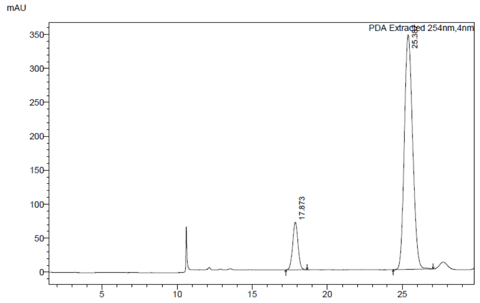

Peak#	Unit	Ret. Time	Area%
1		17.873	11.565
2		25.387	88.435
Total			100.000

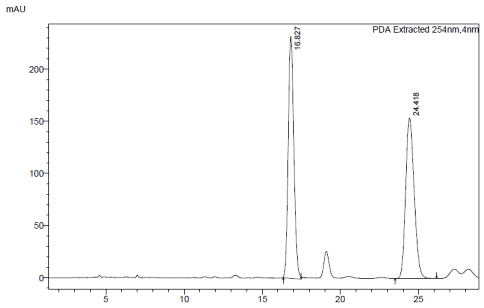

Peak#	Unit	Ret. Time	Area%
1		16.827	49.876
2		24.418	50.124
Total			100.000

(*2R*)-3-(Naphthalen-1-yl)butan-2-one (7p)

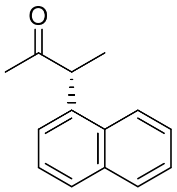

1-(But-2-en-2-yl)naphthalene **6p** (33 mg, 0.18 mmol), reagent **2b** (115 mg, 0.22 mmol), TsOH•H_2O (42 mg, 0.22 mmol) and methanol (22 µL, 0.54 mmol) were reacted according to the general procedure at –78 ºC for 9 h to give **7p** as a colourless oil (23 mg, 64%).

IR (film): 2966, 1712, 1508, 1261, 1103, 806, 783 cm^{-1}.

^{1}H NMR (400 MHz, $CDCl_3$): δ = 8.04 (d, 1H, J = 8 Hz), 7.88-7.90 (m, 1H), 7.78-7.83 (m, 1H), 7.68-7.7.69 (d, 1H, *J* = 1 Hz, 2-nap), 7.43-7.57 (m, 3H), 7.31 (dd, 1H, *J* = 1 Hz, *J* =

7 Hz), 4.44 (q, 1H, *J* = 7 Hz, 1-nap), 3.91 (q, 1H, *J* = 7 Hz, 2-nap), 2.07 (s, 3H, CH_3, 2-nap), 2.00 (s, 3H, CH_3, 1-nap), 1.54 (d, 3H, *J* = 7 Hz, CH_3, 1-nap), 1.47 (d, 3H, *J* = 7 Hz, CH_3, 2-nap) ppm; ^{13}C NMR (100 MHz, $CDCl_3$): δ = 210.0 (1-nap), 207.6 (2-nap), 143.1, 138.7, 137.4, 134.6, 133.6, 131.9, 130.4, 130.2, 129.6, 128.6, 128.3, 128.1, 127.0, 126.3, 126.2, 125.6, 123.5, 50.5, 31.4, 28.5, 19.7, 17.4 ppm. Data in agreement with literature.[26]

HRMS (EI^+) *m/z* calcd for $C_{14}H_{14}O$ $(M)^+$: 198.1045; found: 198.1045.

HPLC: YMC Chiral Amylose-C column, hexane/*i*-PrOH = 99.0/1.0, 1.0 mL/min, 10 ºC, 254 nm; t_R (*S*) = 6.9 min, t_R (*R*) = 7.5 min; 89% *ee*. For (*2R*)-3-(naphthalen-2-yl)butan-2-one: 272 nm; t_R (*S*) = 8.1 min, t_R (*R*) = 8.6 min; 85% *ee*.

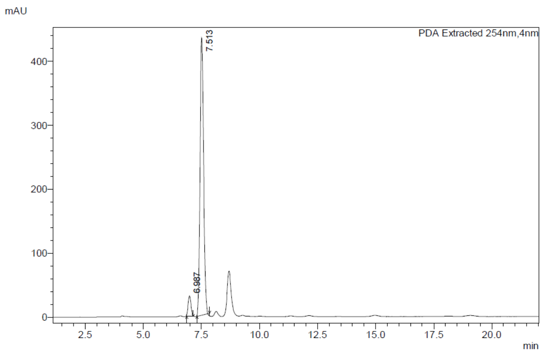

Peak#	Unit	Ret. Time	Area%
1		6.987	5.773
2		7.513	94.227
Total			100.000

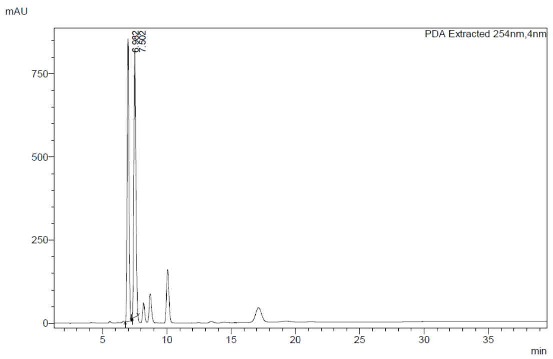

Peak#	Unit	Ret. Time	Area%
1		6.982	48.296
2		7.502	51.704
Total			100.000

(*2S*)-3-(Thiophen-2-yl)hexan-2-one (7q)

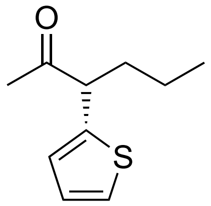

2-(Hex-2-en-2-yl)thiophene **6q** (30 mg, 0.18 mmol), reagent **2b** (115 mg, 0.22 mmol), TsOH•H_2O (42 mg, 0.22 mmol) and methanol (22 μL, 0.54 mmol) were reacted according to the general procedure at –78 ºC for 9 h to give **7q** as a colourless oil (20 mg, 63%).

IR (film): 2958, 2931, 1716, 1354, 1261, 1099, 1037, 817, 702 cm^{-1}.

^{1}H NMR (400 MHz, $CDCl_3$): δ = 7.14 (dd, 1H, *J* = 4 Hz, *J* = 1 Hz), 6.90 (dd, 1H, *J* = 4 Hz, *J* = 3 Hz), 6.81 (dd, 1H, *J* = 3 Hz, *J* = 1 Hz), 3.83 (t, 1H, *J* = 6 Hz), 2.06 (s, 3H), 1.90-

1.97 (m, 1H), 1.65-1.72 (m, 1H), 1.16-1.26 (m, 2H), 0.84 (t, 3H, *J* = 6 Hz) ppm; ^{13}C NMR (100 MHz, $CDCl_3$): δ = 207.5, 141.6, 127.0, 125.5, 124.7, 54.2, 35.0, 28.2, 20.6, 13.9 ppm.

HRMS (EI^+) *m/z* calcd for $C_{10}H_{14}OS$ $(M)^+$: 182.0765; found: 182.0765.

HPLC: Enantiomers inseparable by HPLC on Diacel Chiralpak AD-H, OD-H, OB-H and OJ columns.

$[\alpha]_D^{20}$ = –14.0 (c = 1.0, $CHCl_3$). Enantiomers inseparable by HPLC on Diacel Chiralpak AD-H, OD-H, OB-H and OJ columns.

(*2S*)-Phenyl-2-(thiophen-2-yl)propan-1-one (7r)

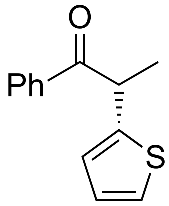

2-(1-Phenylprop-1-enyl)thiophene **6r** (72 mg, 0.36 mmol), reagent **2b** (230 mg, 0.44 mmol), $TsOH{\bullet}H_2O$ (84 mg, 0.4 mmol) and methanol (44 µL, 1.08 mmol) were reacted according to the general procedure at –78 ºC for 9 h to give **7r** as a colourless oil (14 mg, 18%).

IR (film): 3066, 2978, 2931, 1689, 1597, 1446, 1230, 702 cm^{-1}.

^{1}H NMR (400 MHz, $CDCl_3$): δ = 7.93 (dd, 2H, *J* = 8 Hz, *J* = 2 Hz), 7.42-7.49 (m, 1H), 7.37 (t, 2H, *J* = 7 Hz), 7.10 (dd, 1H, *J* = 5 Hz, *J* = 1 Hz), 6.80-6.85 (m, 2H), 4.93 (q, 1H, *J* = 7 Hz), 1.54 (d, 3H, *J* = 7 Hz) ppm. Data in agreement with literature.[27]

HRMS ($APCI^+$) *m/z* calcd for $C_{13}H_{13}OS$ $(M+H)^+$: 217.0642; found: 217.0683.

HPLC: YMC Chiral Amylose-C column, hexane/*i*-PrOH = 99.0/1.0, 1.0 mL/min, 10 ºC, nm; t_R (*S*) = 11.0 min, t_R (*R*) = 12.2 min; 55% *ee*.

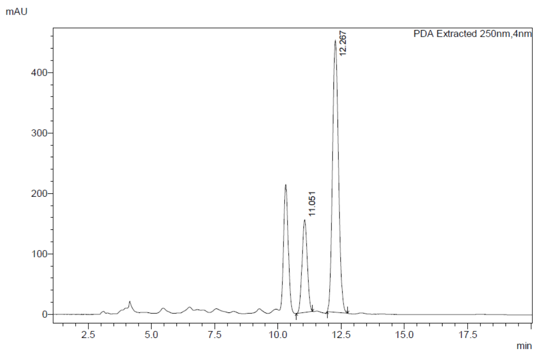

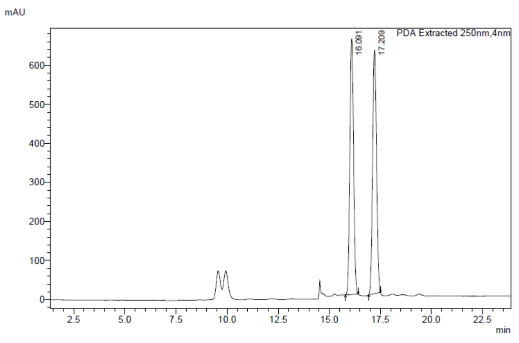

Peak#	Unit	Ret. Time	Area%
1		11.051	22.650
2		12.267	77.350
Total			100.000

Peak#Unit	Ret. Time	Area%
1	16.091	50.079
2	17.209	49.921
Total		100.000

2-Methyl-1,2-diphenylbutan-1-one (7s)

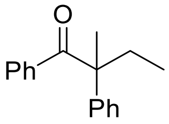

2-Methyl-1,1-diphenylbut-1-ene **6s** (40 mg, 0.18 mmol), (diacetoxyiodo)benzene (71 mg, 0.22 mmol), TsOH•H_2O (42 mg, 0.22 mmol) and methanol (22 µL, 0.54 mmol) were reacted according to the general procedure at rt for 9 h to give **7s** as a colourless oil (5 mg, 12%).

^{1}H NMR (400 MHz, $CDCl_3$): δ = 7.42-7.46 (m, 2H), 7.28-7.36 (m, 5H), 7.18-7.23 (m, 3H), 2.12-2.20 (m, 1H), 2.05-2.10 (m, 1H), 1.54 (s, 3H), 0.74 (t, 3H, *J* = 6 Hz) ppm; HRMS (EI^+) *m/z* calcd for $C_{17}H_{18}O$ $(M)^+$: 238.1358; found: 238.1359. Data in agreement with the literature.[28]

(2*S*)-3-(4-Fluorophenyl)-3-oxo-2-phenylpropyl acetate (7t)

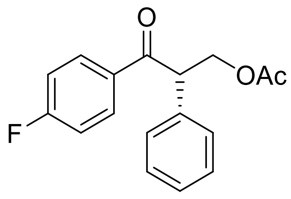

(*E*)-3-(4-Fluorophenyl)-3-phenylallyl acetate **(*E*)-(6t)** (18 mg, 0.07 mmol), reagent **2b** (42 mg, 0.08 mmol), TMSOTf (15 µL, 0.08 mmol) and methanol (16 µL, 0.39 mmol) were reacted according to the general procedure at –78 ºC for 6 h to give **7t** as a colourless oil (17 mg, 89%).

IR (film): 2947, 1736, 1682, 1599, 1231, 1155, 1040, 700 cm^{-1}.

^{1}H NMR (400 MHz, $CDCl_3$): δ = 7.92 (2H, dd, *J* = 9, J_{H-F} = 5 Hz), 7.28-7.18 (5H, m), 6.99 (2H, t, J_{H-F} = 9 Hz), 4.83 (1H, dd, *J* = 9, 3 Hz), 4.69 (1H, dd, *J* = 9, 3 Hz), 4.32 (1H, app dd, *J* = 6, 6 Hz), 1.93 (3H, s) ppm; ^{13}C NMR (125 MHz, $CDCl_3$): δ = 195.5, 170.8, 165.7 (d, J_{C-F} = 255 Hz), 135.3, 132.7 (d, J_{C-F} = 3 Hz), 131.4 (2C, d, J_{C-F} = 9 Hz), 129.3 (2C), 128.4

(2C), 128.0, 115.8 (2C, d, $J_{C\text{-}F}$ = 22 Hz), 65.5, 52.5, 20.8 ppm; ^{19}F NMR (282 MHz, $CDCl_3$): δ = −104.4 ppm.

MS (ES^+) *m/z* = 309 ($[M+Na]^+$, 100); HRMS (ES^+) *m/z* calcd for $C_{17}H_{15}FO_3Na$ $(M+Na)^+$: 309.0903; found: 309.0895.

$[\alpha]_D^{20}$ = –140.0 (c = 1.0, $CHCl_3$); HPLC: Daicel Chiralcel OB-H column, hexane/*i*-PrOH = 96/4, 0.5 mL/min, 10 ºC, 246 nm; t_R (*S*) = 55.1 min; t_R (*R*) = 78.9 min, >95% *ee*.

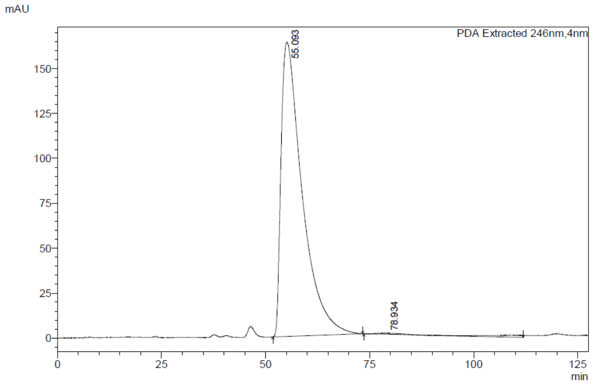

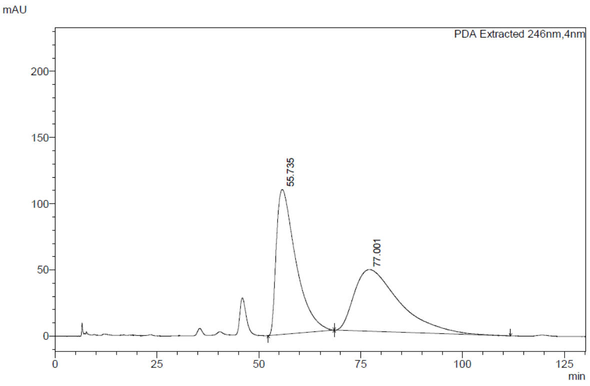

Peak#	Unit	Ret. Time	Area%
1		55.093	98.543
2		78.934	1.457
Total			100.000

Peak#	Unit	Ret. Time	Area%
1		55.735	50.346
2		77.001	49.654
Total			100.000

(2*S*)-2-(4-Fluorophenyl)-3-oxo-3-phenylpropyl acetate (7t′)

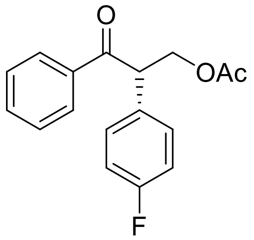

(*Z*)-3-(4-Fluorophenyl)-3-phenylallyl acetate **(*Z*)-(6t)** (22 mg, 0.08 mmol), reagent **2b** (51 mg, 0.10 mmol), TMSOTf (18 μL, 0.10 mmol) and methanol (20 μL, 0.49 mmol) were reacted according to the general procedure at –78 ºC for 7 h to give **7t′** as a colourless oil (21 mg, 90%).

IR (film): 2955, 1734, 1680, 1506, 1217, 1042, 696 cm^{-1}.

^{1}H NMR (400 MHz, $CDCl_3$): δ = 7.95 (2H, d, *J* = 7 Hz), 7.54 (1H, t, *J* = 7 Hz), 7.42 (2H, t, *J* = 7 Hz), 7.33-7.28 (3H, m), 7.02 (2H, t, $J_{H\text{-}F}$ = 9 Hz), 4.96 (1H, app dd, *J* = 6, 9 Hz),

4.74 (1H, app dd, J = 9, 11 Hz), 4.40-4.36 (1H, m), 2.00 (3H, s) ppm; ^{13}C NMR (75 MHz, $CDCl_3$): δ = 197.1, 170.9, 162.3 (d, $J_{C\text{-}F}$ = 247 Hz), 136.0, 133.4, 131.1 (d, $J_{C\text{-}F}$ = 3 Hz), 130.0 (2C, d, $J_{C\text{-}F}$ = 8 Hz), 128.7 (4C), 116.2 (2C, d, $J_{C\text{-}F}$ = 22 Hz), 65.4, 51.4, 20.9 ppm; ^{19}F NMR (282 MHz, $CDCl_3$): δ = −114.0 ppm.

MS (ES$^+$) *m/z* = 309 ([M+Na]$^+$, 100); HRMS (ES$^+$) *m/z* calcd for $C_{17}H_{15}O_3F$ (M)$^+$: 286.1005; found: 286.1007.

$[\alpha]_D^{20}$ = –104.0 (c = 1.0, $CHCl_3$); HPLC: Daicel Chiralcel OJ column, hexane/*i*-PrOH = 99/1, 1.0 mL/min, 10 ºC, 243 nm; t_R (*R*) = 72.7 min, t_R (*S*) = 83.0 min; 89% *ee*.

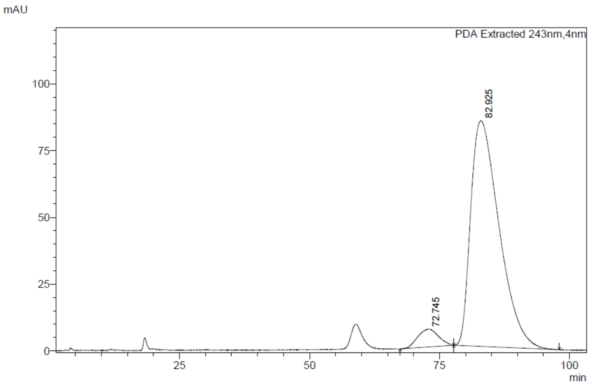

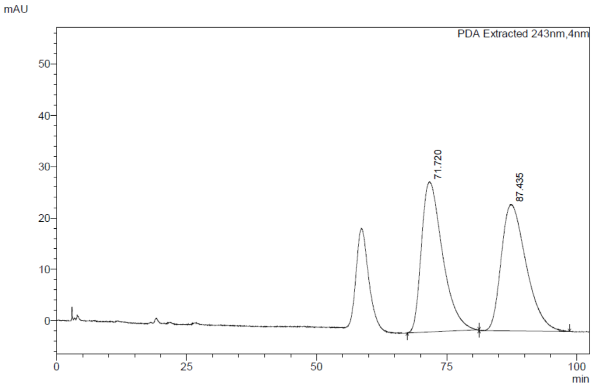

Peak#	Unit	Ret. Time	Area%
1		72.745	5.485
2		82.925	94.515
Total			100.000

Peak#	Unit	Ret. Time	Area%
1		71.720	50.166
2		87.435	49.834
Total			100.000

(2*R*)-1,2-Bis(3-chlorophenyl)pentan-1-one (7u)

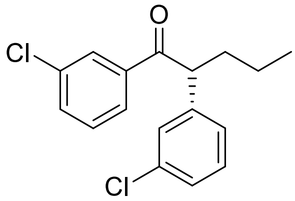

1,1-Bis(3-chlorophenyl)pentene **6u** (32 mg, 0.11 mmol), reagent **2b** (69 mg, 0.13 mmol), TMSOTf (24 μL, 0.13 mmol) and methanol (27 μL, 0.66 mmol) were reacted according to the general procedure at –78 ºC for 6 h to give **7u** as a colourless oil (23 mg, 68%). Recovered **6u** (2 mg, 6%).

IR (film): 2955, 1510, 1454, 1111, 818, 766, 746 cm^{-1}.

^{1}H NMR (400 MHz, $CDCl_3$): δ = 7.94 (1H, s), 7.83 (1H, d, J = 8 Hz), 7.51 (1H, d, J = 8 Hz), 7.39 (1H, t, J = 8 Hz), 7.32 (1H, s), 7.29 (1H, s), 7.27-7.19 (2H, m), 4.49 (1H, t, J = 7 Hz), 2.19-2.10 (1H, m), 1.87-1.78 (1H, m), 1.38-1.23 (2H, m), 0.95 (3H, t, J = 7 Hz) ppm;

^{13}C NMR (125 MHz, $CDCl_3$): δ = 198.3, 141.2, 138.4, 135.0, 134.8, 133.0, 130.2, 129.9, 128.7, 128.3, 127.4, 126.6, 126.4, 53.2, 36.1, 20.8, 14.0 ppm.

MS (EI$^+$) *m/z* = 310 (M[^{37}Cl,^{37}Cl]$^+$, 7), 308 (M[^{37}Cl,^{35}Cl]$^+$, 38), 306 (M[^{35}Cl,^{35}Cl]$^+$, 56); HRMS (EI$^+$) *m/z* calcd for $C_{17}H_{16}{}^{35}Cl_2O$ (M)$^+$: 306.0578; found: 306.0577.

$[\alpha]_D^{20}$ = –80.0 (c = 1.0, $CHCl_3$); HPLC: Daicel Chiralcel OD-H column, hexane/*i*-PrOH = 99.5/0.5, 1.0 mL/min, 10 ºC, 238 nm; t_R (*S*) = 5.1 min, t_R (*R*) = 7.2 min; 68% *ee*.

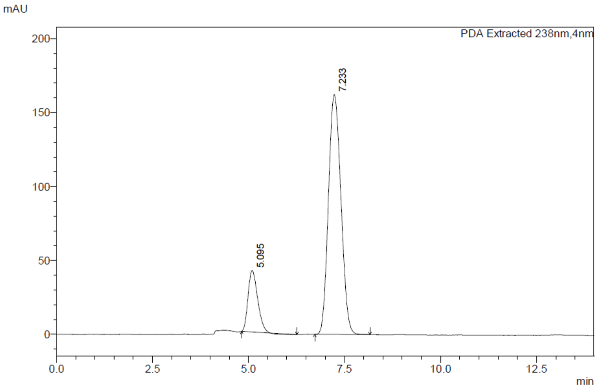

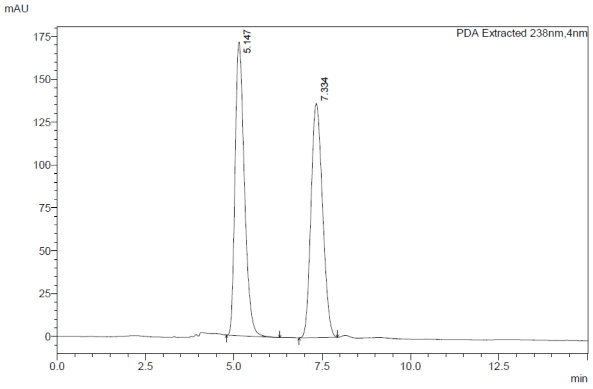

Peak#	Unit	Ret. Time	Area%
1		5.095	16.228
2		7.233	83.772
Total			100.000

Peak#	Unit	Ret. Time	Area%
1		5.147	50.366
2		7.334	49.634
Total			100.000

(2*R*)-1,2-Bis(3-(trifluoromethyl)phenyl)pentan-1-one (7v)

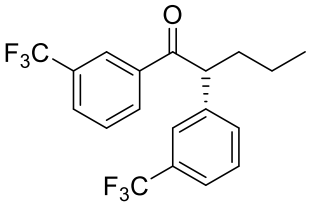

1,1-Bis(3-(trifluoromethyl)phenyl)pentene **6v** (40 mg, 0.11 mmol), reagent **2b** (71 mg, 0.13 mmol), TMSOTf (24 µL, 0.13 mmol) and methanol (29 µL, 0.67 mmol) were reacted according to the general procedure at –78 ºC for 7 h to give **7v** as a colourless oil (17 mg, 41%). Recovered **6v** (11 mg, 28%).

IR (film): 2965, 1682, 1568, 1206, 1078, 785, 677, 422 cm^{-1}.

^{1}H NMR (400 MHz, $CDCl_3$): δ = 8.22 (1H, s), 8.12 (1H, d, *J* = 8 Hz), 7.78 (1H, d, *J* = 8 Hz), 7.60-7.56 (2H, m), 7.51 (2H, app d, *J* = 8 Hz), 7.46-7.42 (1H, m), 4.62 (1H, t, *J* = 7 Hz), 2.24-2.15 (1H, m), 1.90-1.81 (1H, m), 1.38-1.22 (2H, m), 0.94 (3H, t, *J* = 7 Hz) ppm; ^{13}C NMR (125 MHz, $CDCl_3$, ^{19}F decoupled): δ = 198.6, 138.4, 135.4, 132.1 (2C), 131.9 (2C),

131.8, 131.4, 130.1 (2C), 129.9 (2C), 128.2, 121.1, 52.8, 35.9, 20.7, 14.0 ppm; ^{19}F NMR (282 MHz, $CDCl_3$): δ = −62.5 (3F), −62.8 (3F) ppm.

MS (EI$^+$) *m/z* = 374 (M$^+$, 100); HRMS (APCI$^+$) *m/z* calcd for $C_{19}H_{17}F_6O$ $(M+H)^+$: 375.1178; found: 375.1180.

$[\alpha]_D^{20}$ = –40.0 (c = 1.0, $CHCl_3$); HPLC: YMC AD-H column, hexane/*i*-PrOH = 99.75/0.25 1.0 mL/min, 10 ºC, 359 nm; t_R (*S*) = 5.9 min, t_R (*R*) = 6.5 min; 46% *ee*.

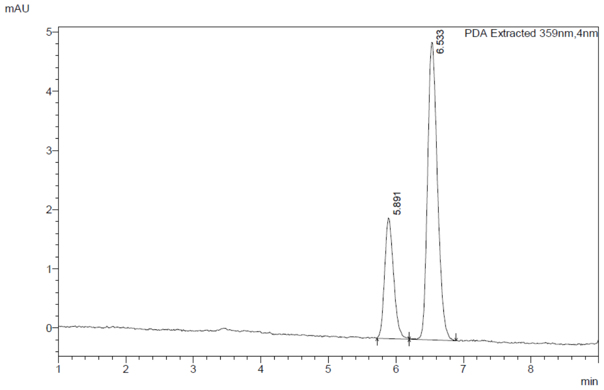

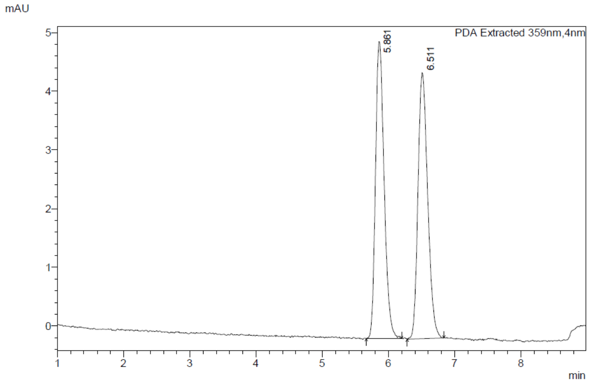

Peak#	Unit	Ret. Time	Area%
1		5.891	26.868
2		6.533	73.132
Total			100.000

Peak#	Unit	Ret. Time	Area%
1		5.861	49.954
2		6.511	50.046
Total			100.000

(2*R*)-1,2-Bis(4-chlorophenyl)pentan-1-one (7w)

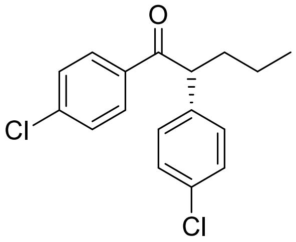

1,1-Bis(4-chlorophenyl)pentene **6w** (23 mg, 0.08 mmol), reagent **2b** (50 mg, 0.09 mmol), TMSOTf (17 μL, 0.09 mmol) and methanol (20 μL, 0.47 mmol) were reacted according to the general procedure at –78 ºC for 6 h to give **7w** as a colourless oil (19 mg, 79%).

IR (film): 2957, 2155, 1680, 1589, 1487, 1092, 1011, 800 cm^{-1}.

^{1}H NMR (500 MHz, $CDCl_3$): δ = 7.80 (2H, d, *J* = 9 Hz), 7.30 (2H, d, *J* = 9 Hz), 7.21-7.18 (2H, m), 7.15-7.13 (2H, m), 4.39 (1H, t, *J* = 7 Hz), 2.07-2.00 (1H, m), 1.74-1.67 (1H, m), 1.27-1.11 (2H, m), 0.84 (3H, t, *J* = 7 Hz) ppm; ^{13}C NMR (125 MHz, $CDCl_3$): δ = 198.5, 139.4, 137.9, 134.9, 133.0, 130.0 (2C), 129.5 (2C), 129.1 (2C), 128.9 (2C), 52.7, 35.9, 20.7, 14.0 ppm.

MS (EI$^+$) m/z = 310 (M[^{37}Cl,^{37}Cl]$^+$, 7), 308 (M[^{37}Cl,^{35}Cl]$^+$, 38), 306 (M[^{35}Cl,^{35}Cl]$^+$, 56); HRMS (EI$^+$) m/z calcd for $C_{17}H_{16}{}^{35}Cl_2O$ (M)$^+$: 306.0578; found: 306.0575.

$[\alpha]_D{}^{20}$ = –36.0 (c = 1.0, $CHCl_3$); HPLC: YMC Chiral Amylose-C column, hexane/*i*-PrOH = 99.5/0.5, 1.0 mL/min, 10 ºC, 262 nm; t_R (*S*) = 13.1 min, t_R (*R*) = 14.5 min; 77% *ee*.

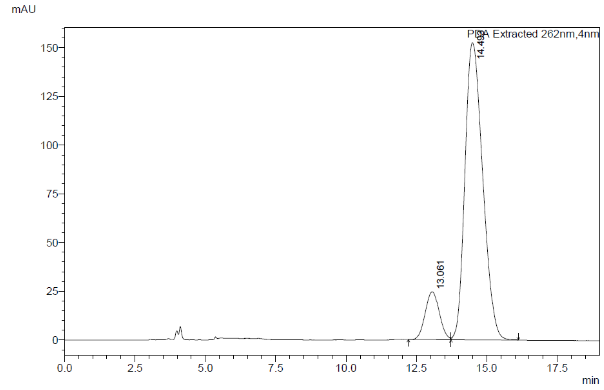

Peak#	Unit	Ret. Time	Area%
1		13.061	11.398
2		14.493	88.602
Total			100.000

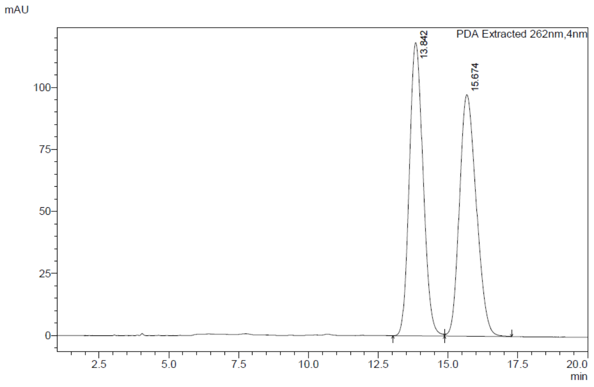

Peak#	Unit	Ret. Time	Area%
1		13.842	49.839
2		15.674	50.161
Total			100.000

(2*R*)-1,2-Bis(4-bromophenyl)pentan-1-one (7x)

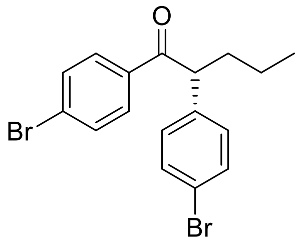

1,1-Bis(4-bromophenyl)pentene **6x** (42 mg, 0.11 mmol), reagent **2b** (70 mg, 0.13 mmol), TMSOTf (24 μL, 0.13 mmol) and methanol (29 μL, 0.66 mmol) were reacted according to the general procedure at –78 ºC for 6 h to give **7x** as a colourless oil (36 mg, 82%).

IR (film): 2955, 1678, 1585, 1485, 1394, 1070, 1011, 793, 739 cm^{-1}.

^{1}H NMR (500 MHz, $CDCl_3$): δ = 7.79 (2H, d, J = 9 Hz), 7.55 (2H, d, J = 9 Hz), 7.42 (2H, d, J = 9 Hz), 7.16 (2H, d, J = 9 Hz), 4.45 (1H, t, J = 7 Hz), 2.15-2.08 (1H, m), 1.82-1.74 (1H, m), 1.35-1.20 (2H, m), 0.92 (3H, t, J = 7 Hz) ppm; ^{13}C NMR (125 MHz, $CDCl_3$): δ = 198.6, 138.4, 135.4, 132.0 (2C), 131.9 (2C), 130.1 (2C), 129.9 (2C), 128.2, 121.1, 52.8, 35.9, 20.7, 14.0 ppm.

MS (ES$^-$) m/z = 397 ([M(^{81}Br,^{81}Br)−H]$^-$, 395 ([M(^{79}Br,^{81}Br)−H]$^-$, 50), 393 ([M(^{79}Br,^{79}Br)−H]$^-$, 25). HRMS (EI$^+$) m/z calcd for $C_{17}H_{16}{}^{79}Br_2O$ (M)$^+$: 393.9568; found: 393.9569.

$[\alpha]_D^{20}$ = –20.0 (c = 1.0, $CHCl_3$); HPLC: YMC Chiral Amylose-C column, hexane/*i*-PrOH = 99.5/0.5, 1.0 mL/min, 10 ºC, 262 nm; t_R (*S*) = 10.7 min, t_R (*R*) = 13.5 min; 83% *ee*.

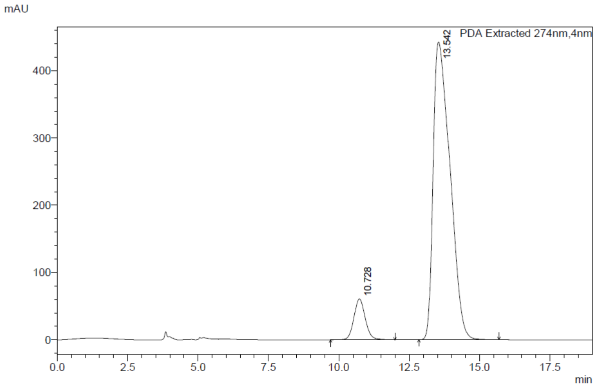

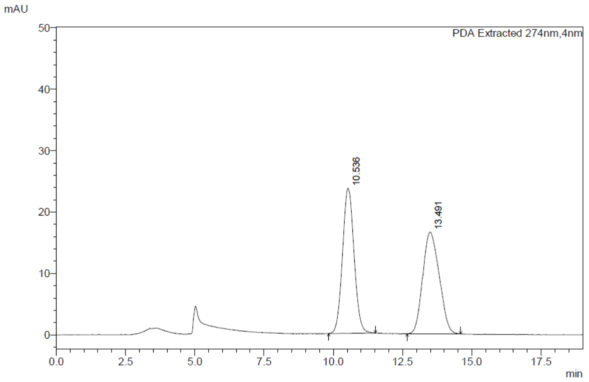

Peak#	Unit	Ret. Time	Area%
1		10.728	8.461
2		13.542	91.539
Total			100.000

Peak#	Unit	Ret. Time	Area%
1		10.536	49.925
2		13.491	50.075
Total			100.000

(2*R*)-1,2-Bis(4-methylphenyl)pentan-1-one (7y)

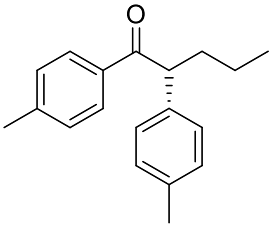

1,1-Bis(4-methylphenyl)pentene **6y** (25 mg, 0.10 mmol), reagent **2b** (63 mg, 0.12 mmol), TMSOTf (22 μL, 0.12 mmol) and methanol (26 μL, 0.60 mmol) were reacted according to the general procedure at –78 ºC for 1 h to give **7y** as a colourless oil (24 mg, 90%).

IR (film): 2965, 1674, 1604, 1179, 814, 799, 549 cm^{-1}.

^{1}H NMR (400 MHz, $CDCl_3$): δ = 7.88 (2H, d, J = 8 Hz), 7.21-7.18 (4H, m), 7.10 (2H, d, J = 8 Hz), 4.51 (1H, t, J = 7 Hz), 2.36 (3H, s), 2.29 (3H, s), 2.18-2.09 (1H, m), 1.84-1.75 (1H, m), 1.37-1.21 (2H, m), 0.92 (3H, t, J = 7 Hz) ppm; ^{13}C NMR (75 MHz, $CDCl_3$): δ = 199.9, 143.5, 137.0, 136.5, 134.5, 129.5 (2C), 129.2 (2C), 128.8 (2C), 128.1 (2C), 52.8, 36.1, 21.6, 21.1, 20.9, 14.1 ppm.

MS (EI$^+$) *m/z* = 266 (M$^+$, 100); HRMS (EI$^+$) *m/z* calcd for $C_{19}H_{22}O$ (M)$^+$: 266.1671; found: 266.1673.

$[\alpha]_D^{20}$ = –68.6 (c = 1.0, $CHCl_3$); HPLC: YMC Chiral Amylose-C column, hexane/*i*-PrOH = 99.5/0.5, 1.0 mL/min, 10 ºC, 251 nm; t_R (*S*) = 13.0 min, t_R (*R*) = 17.9 min; 86% *ee*.

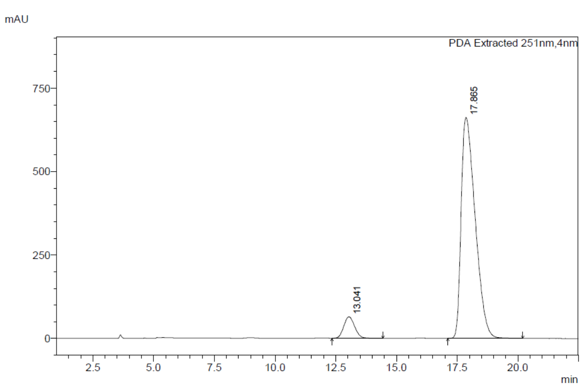

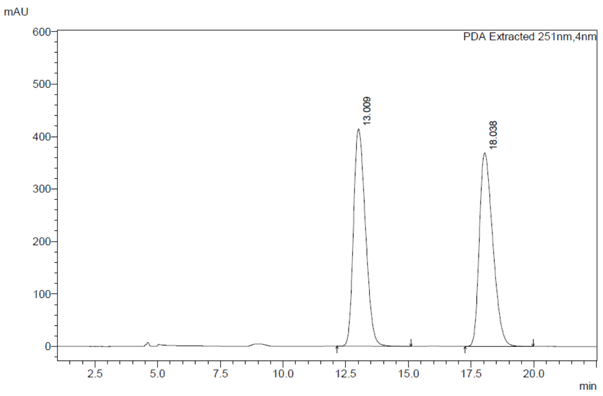

Peak#	Unit	Ret. Time	Area%
1		13.041	7.065
2		17.865	92.935
Total			100.000

Peak#	Unit	Ret. Time	Area%
1		13.009	49.907
2		18.038	50.093
Total			100.000

(2*R*)-1,2-Bis(4-fluorophenyl)pentan-1-one (7z)

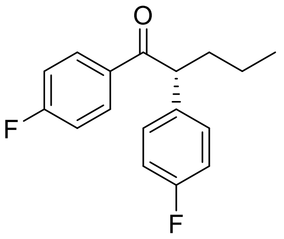

1,1-Bis(4-fluorophenyl)pentene **(6z)** (48 mg, 0.18 mmol), reagent **2b** (115 mg, 0.22 mmol), TMSOTf (41 μL, 0.22 mmol) and methanol (29 μL, 0.72 mmol) were reacted according to the general procedure at –78 ºC for 8 h to give **7z** as a colourless oil (37 mg, 73%).

IR (neat): 2924, 1681, 1597, 1508, 1267, 1234, 1157, 833 cm^{-1}.

^{1}H NMR (400 MHz, $CDCl_3$): δ = 7.87-7.92 (m, 2H), 7.16-7.20 (m, 2H), 6.97-7.03 (m, 2H), 6.88-6.94 (m, 2H), 4.42 (t, 1H, *J* = 7 Hz), 2.00-2.08 (m, 1H), 1.66-1.75 (m, 1H), 1.12-1.27 (m, 2H), 0.84 (t, 3H, *J* = 7 Hz) ppm; ^{13}C NMR (100 MHz, $CDCl_3$): δ = 198.9, 166.0 (d, *J* = 258 Hz), 162.3 (d, *J* = 235 Hz), 135.7 (d, *J* = 3 Hz), 133.5 (d, *J* = 3 Hz), 131.6 (d, *J* = 8 Hz), 130.1 (d, *J* = 8 Hz), 130.0 (d, *J* = 11 Hz), 116.0 (d, *J* = 21 Hz), 53.0, 36.6, 21.2, 14.4 ppm.

HRMS (APCI$^+$) *m/z* calcd for $C_{17}H_{16}F_2O$ (M+H)$^+$: 275.1247; found: 275.1237

$[\alpha]_D^{20}$ = −75.0 (c = 1.0, $CHCl_3$); HPLC: YMC Chiral Amylose-C column, hexane/*i*-PrOH = 99.5/0.5, 1.0 mL/min, 10 ºC, 265 nm; t_R (*R*) = 12.3 min, t_R (*S*) = 13.3 min; 87% *ee*.

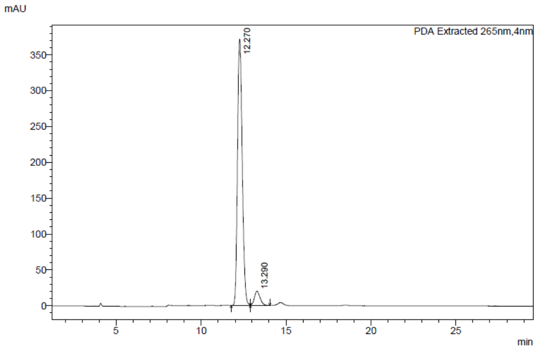

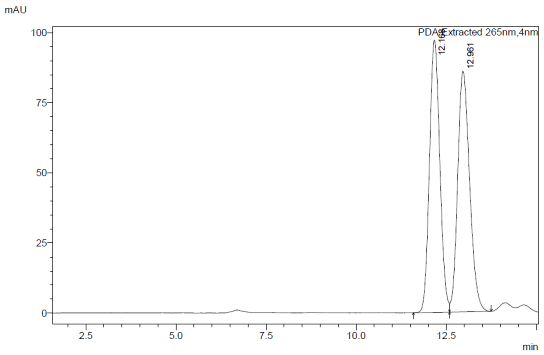

Peak#	Unit	Ret. Time	Area%
1		12.270	93.865
2		13.290	6.135
Total			100.00

Peak#	Unit	Ret. Time	Area%
1		12.168	49.610
2		12.961	50.390
Total			100.000

Synthesis of Lumiracoxib analogue (*R*)-8

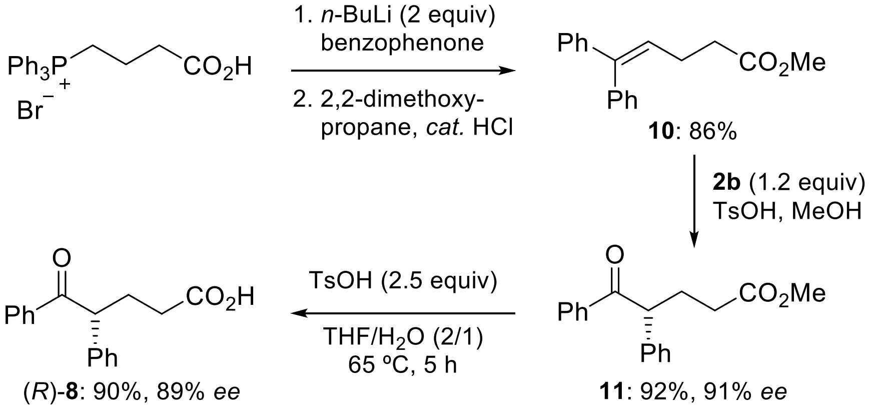

(4*R*)-Methyl 5-oxo-4,5-diphenylpentanoate (11)

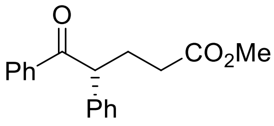

Methyl 5,5-diphenyl-4-pentenoate **10** (125 mg, 0.47 mmol) was reacted with reagent **2b** (297 mg, 0.56 mmol), $TsOH•H_2O$ (107 mg, 0.56 mmol) and methanol (57 μL, 1.41 mmol) in CH_2Cl_2: TFE (10:1 v/v) (5 mL) according to the general oxidative rearrangement procedure at –78 ºC for 2 h to give **11** as a colourless oil (122 mg, 92%).

IR (film): 2947, 1728, 1678, 1447, 1211, 1165, 692 cm^{-1}.

^{1}H NMR (500 MHz, $CDCl_3$): δ = 7.96 (2H, d, *J* = 8 Hz), 7.48 (1H, t, *J* = 8 Hz), 7.39 (2H, t, *J* = 8 Hz), 7.30 (2H, s), 7.29 (2H, s), 7.22 (1H, app sext, *J* = 5 Hz), 4.68 (1H, t, *J* = 7 Hz), 3.99 (3H, s), 2.48 (1H, app sext, *J* = 7 Hz), 2.32 (2H, t, *J* = 7 Hz), 2.18 (1H, app sext, *J* = 7 Hz) ppm; ^{13}C NMR (125 MHz, $CDCl_3$): δ = 199.2, 173.6, 138.8, 136.7, 132.9, 129.0 (2C), 128.7 (2C), 128.5 (2C), 128.2 (2C), 127.3, 52.5, 51.5, 31.6, 28.8 ppm.

MS (EI$^+$) *m/z* = 282 (M^+, 100); HRMS (EI$^+$) *m/z* calcd for $C_{18}H_{18}O_3$ $(M)^+$: 282.1256; found: 282.1255.

$[\alpha]_D^{20}$ = –194.6 (c = 1.2, $CHCl_3$); HPLC: YMC Chiral Amylose-C column, hexane/*i*-PrOH = 95/5, 1.0 mL/min, 10 ºC, 244 nm; t_R (*R*) = 14.0 min; t_R (*S*) = 15.0 min; 91% *ee*.

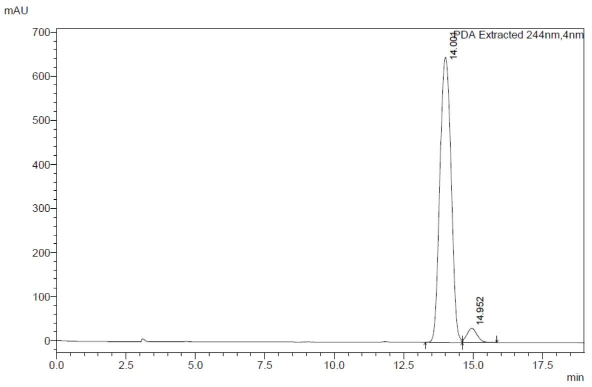

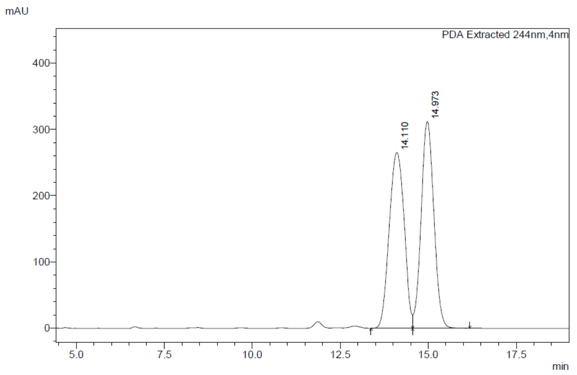

Peak#	Unit	Ret. Time	Area%
1		14.001	95.681
2		14.952	4.319
Total			100.000

Peak#	Unit	Ret. Time	Area%
1		14.110	50.020
2		14.973	49.980
Total			100.00

(4*R*)-5-Oxo-4,5-diphenylpentanoic acid (*R*)-8

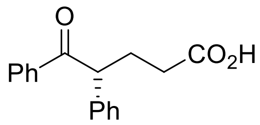

To a solution of methyl ester **11** (10.0 mg, 0.04 mmol) in THF (1 mL) was added TsOH•H_2O (17 mg, 0.09 mmol) and H_2O (0.5 mL), and the resulting mixture heated to at 65 ºC for 5 h. After allowing the reaction to cool to room temperature, the following workup was completed promptly. Diethyl ether (5 mL) and 2M HCl (3 mL) was added, the phases separated and the aqueous phase extracted with diethyl ether (2×5 mL). The combined organic layers were filtered through a TELOS® Phase Separator and concentrated under vacuum to give **8** (8.6 mg mg, 90%) as a colourless solid. Mp. 130-132 ºC.

IR (neat): 2920 (br), 2631, 1721, 1667, 1290, 1236, 1198, 694 cm^{-1}.

^{1}H NMR (400 MHz, $CDCl_3$): δ = 7.88 (2H, d, *J* = 7 Hz), 7.41 (1H, t, *J* = 7 Hz), 7.31 (2H, d, *J* = 7 Hz), 7.25-7.12 (5H, m), 4.60 (1H, t, *J* = 7 Hz), 2.39 (1H, app sext, *J* = 7 Hz), 2.29 (2H, t, *J* = 7 Hz), 2.11 (1H, app sext, *J* = 7 Hz) ppm; ^{13}C NMR (75 MHz, $CDCl_3$): δ = 199.2, 179.2, 138.6, 136.4, 133.1, 129.2 (2C), 128.8 (2C), 128.6 (2C), 128.3 (2C), 127.4, 52.3, 31.4, 28.4 ppm.

MS (EI^+) *m/z* = 268 (M^+, 100); HRMS (EI^+) *m/z* calcd for $C_{17}H_{16}O_3$ $(M)^+$: 268.1099; found: 268.1100.

$[\alpha]_D^{20}$ = –94.2 (c = 1.0, $CHCl_3$); HPLC: YMC Chiral Amylose-C column, hexane/*i*-PrOH/TFA = 95/5/0.1, 1.0 mL/min, 10 ºC, 271 nm; t_R (*S*) = 53.1 min, t_R (*R*) = 58.7 min; 89% *ee*.

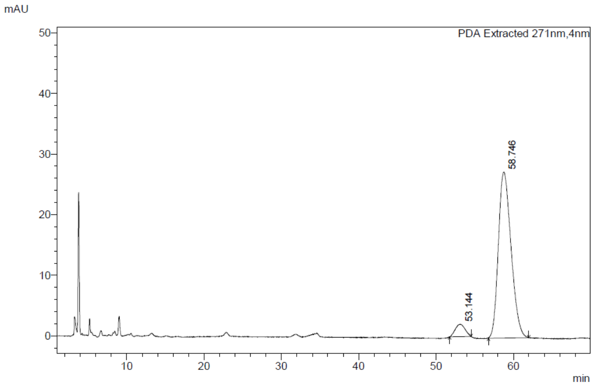

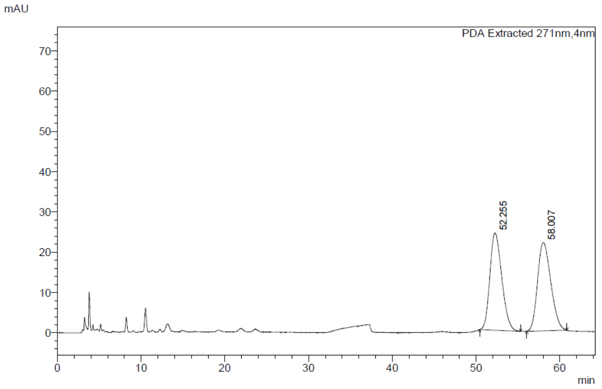

Peak#	Unit	Ret. Time	Area%
1		53.144	5.406
2		58.746	94.594
Total			100.000

Peak#	Unit	Ret. Time	Area%
1		52.255	49.708
2		58.007	50.292
Total			100.000

Assignment of Absolute Configuration

1,2-Diphenylpropan-1-one (*R*)-(7b)

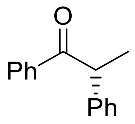

$[\alpha]_D^{20}$ = −189.0 (c = 1.0, $CHCl_3$); 91% *ee*.

Lit.[23] For **(*S*)-(7b)** $[\alpha]_D^{20}$ = +190 (c 1.08, $CHCl_3$); 96% *ee*

Lit.[29] For **(*S*)-(7b)** $[\alpha]_D^{22}$ = +67.4 (c 0.36, $CHCl_3$); 35% *ee*

2-Phenylpentan-3-one (*R*)-(7i)

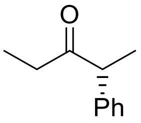

$[\alpha]_D^{20}$ = –111.1 (c = 1.0, $CHCl_3$); 43% *ee*

Lit.[25] For **(*R*)-(7i)** $[\alpha]_D^{22}$ = –47 (c 1.0, $CHCl_3$); 73% *ee*

(2*R*)-2-(4-Anisyl)-1-phenylpropan-1-one (7o)

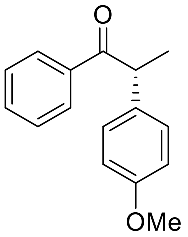

$[\alpha]_D^{20}$ = −126.0 (c = 1.0, $CHCl_3$); 77% *ee*

Lit.[26] For **(*S*)-(7i)** $[\alpha]_D^{22}$ = +161.0 (c = 1.0, $CHCl_3$); 95% *ee*

^{1}H and ^{13}C NMR spectra

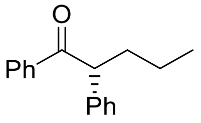

7a

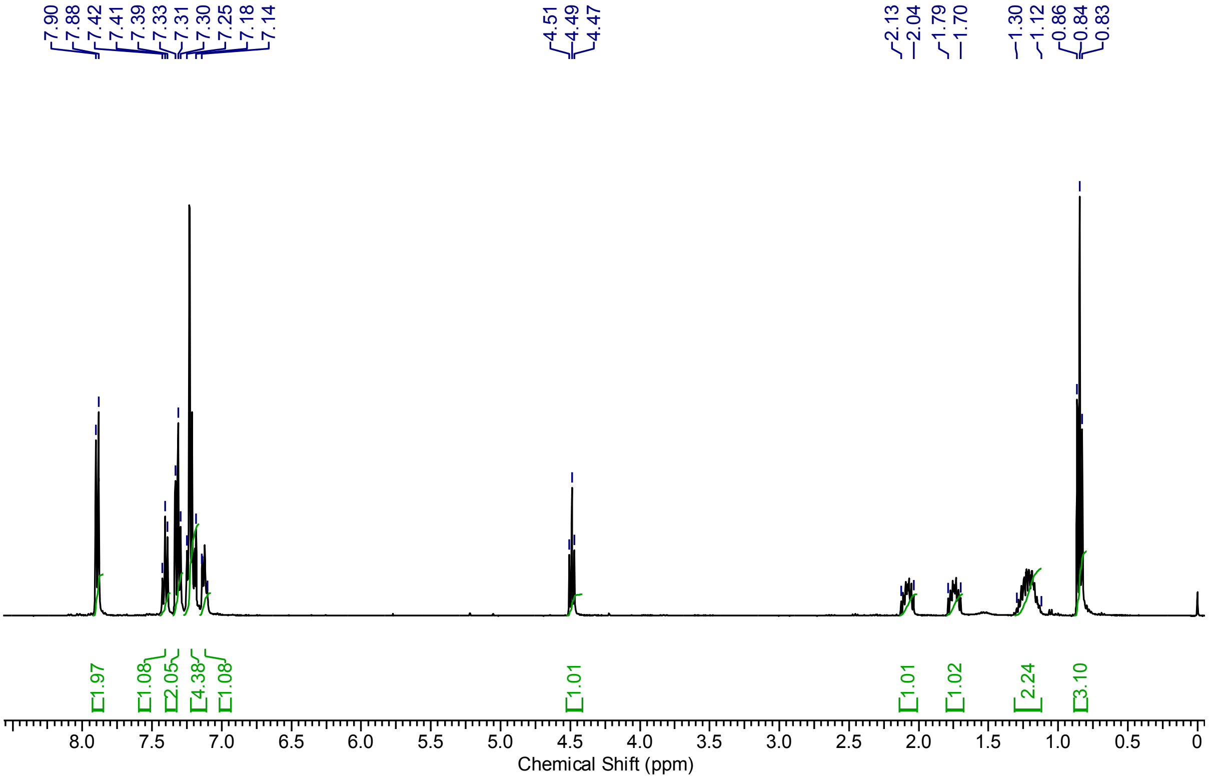

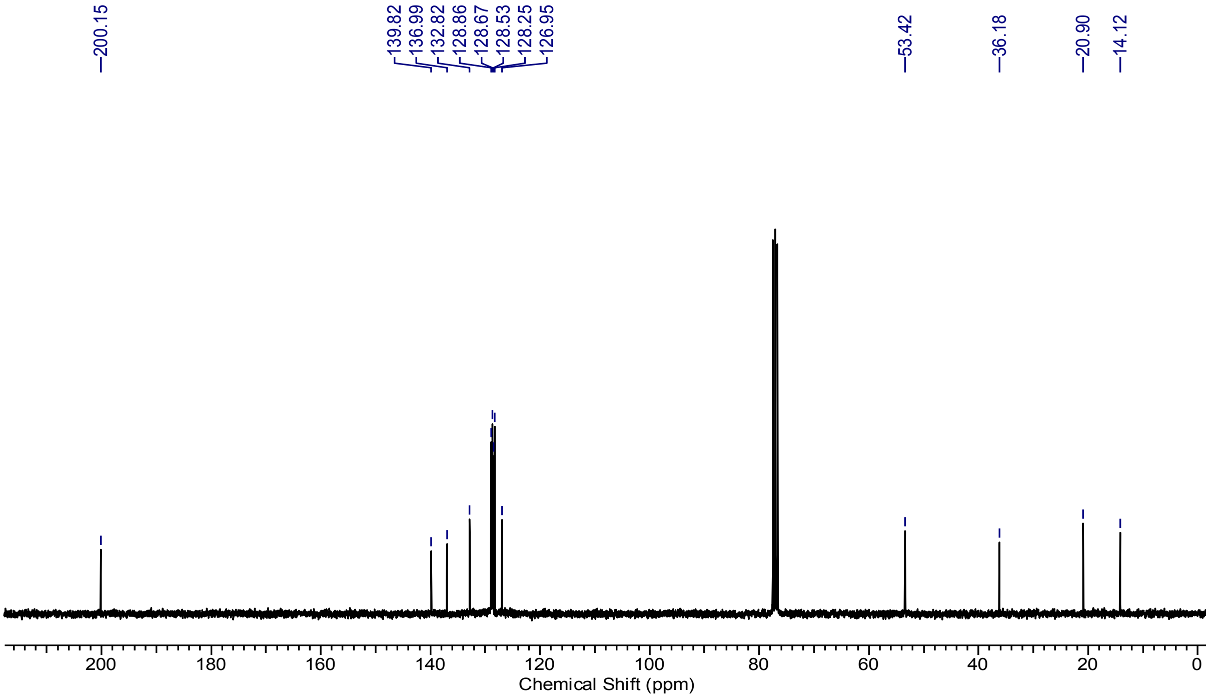

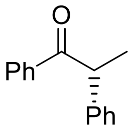
O
Ph
Ph

7b

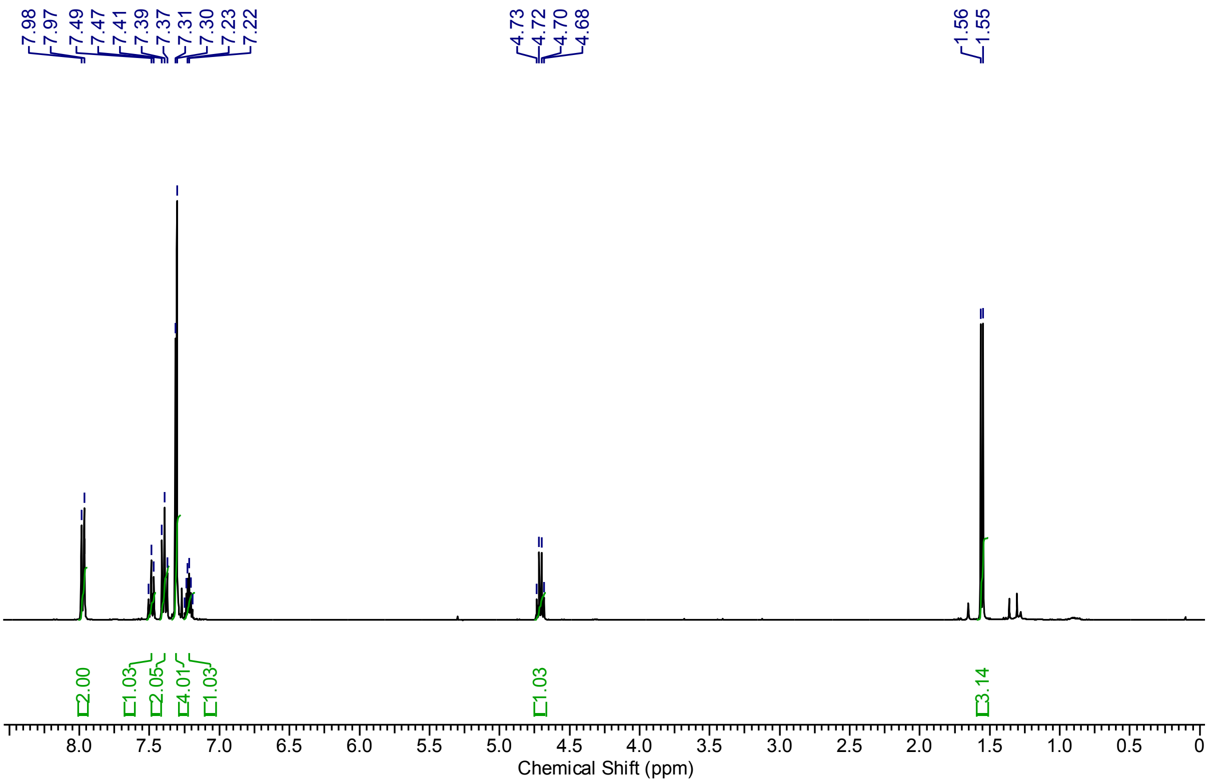
7.98
7.97
7.49
7.47
7.41
7.39
7.37
7.31
7.30
7.23
7.22
4.73
4.72
4.70
4.68
1.56
1.55
2.00
1.03
2.05
4.01
1.03
1.03
3.14
8.0
7.5
7.0
6.5
6.0
5.5
5.0
4.5
4.0
3.5
3.0
2.5
2.0
1.5
1.0
0.5
0
Chemical Shift (ppm)

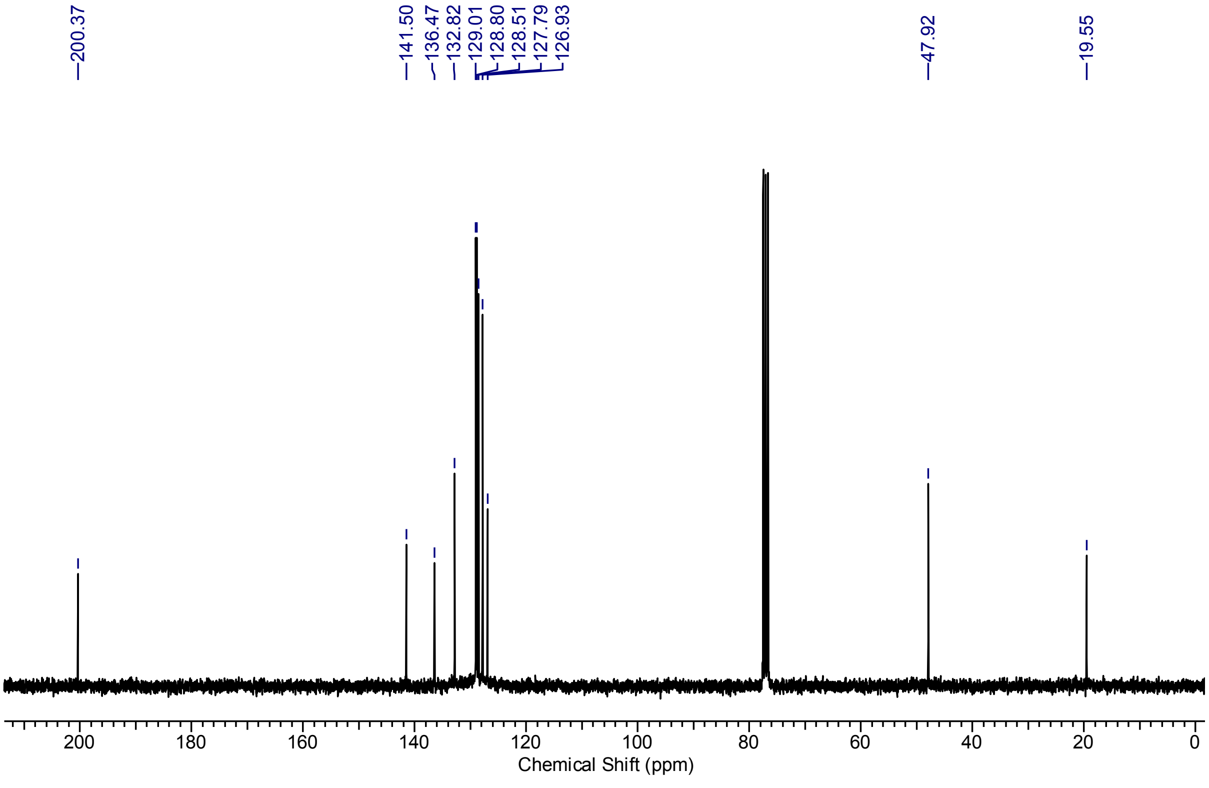
200.37
141.50
136.47
132.82
129.01
128.80
128.51
127.79
126.93
47.92
19.55
200
180
160
140
120
100
80
60
40
20
0
Chemical Shift (ppm)

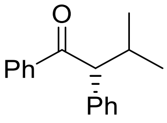
O
Ph
Ph

7c

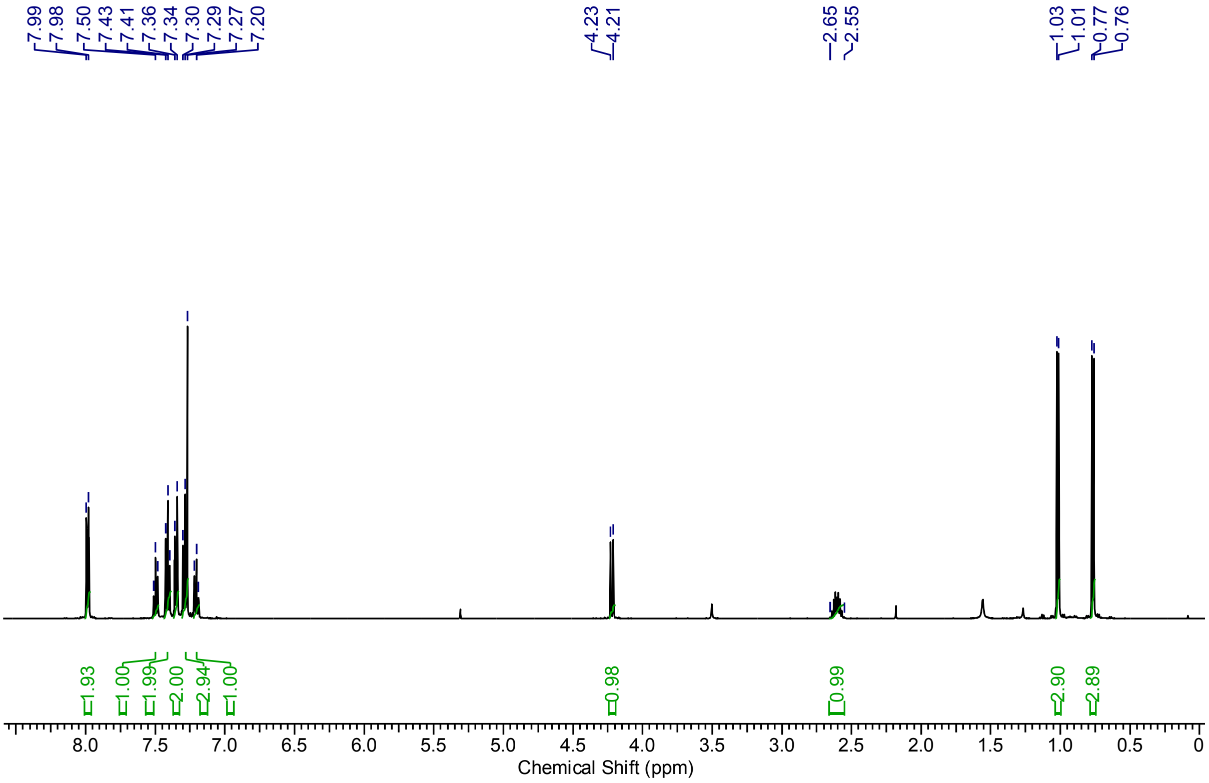
7.99 7.98 7.50 7.43 7.41 7.36 7.34 7.30 7.29 7.27 7.20
4.23 4.21
2.65 2.55
1.03 1.01 0.77 0.76
1.93 1.00 1.99 2.00 2.94 1.00
0.98
0.99
2.90 2.89
8.0 7.5 7.0 6.5 6.0 5.5 5.0 4.5 4.0 3.5 3.0 2.5 2.0 1.5 1.0 0.5 0
Chemical Shift (ppm)

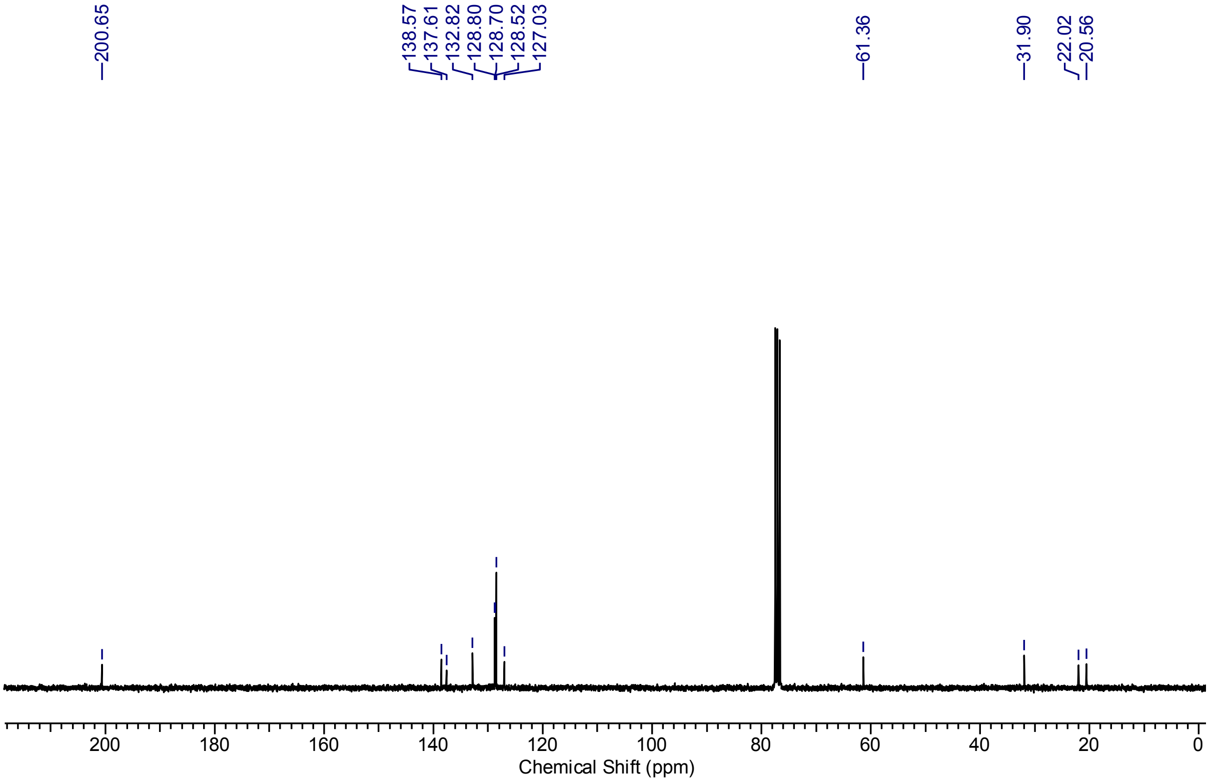
200.65
138.57 137.61 132.82 128.80 128.70 128.52 127.03
61.36
31.90
22.02 20.56
200 180 160 140 120 100 80 60 40 20 0
Chemical Shift (ppm)

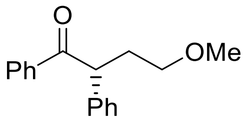
O
Ph
OMe
Ph

7d

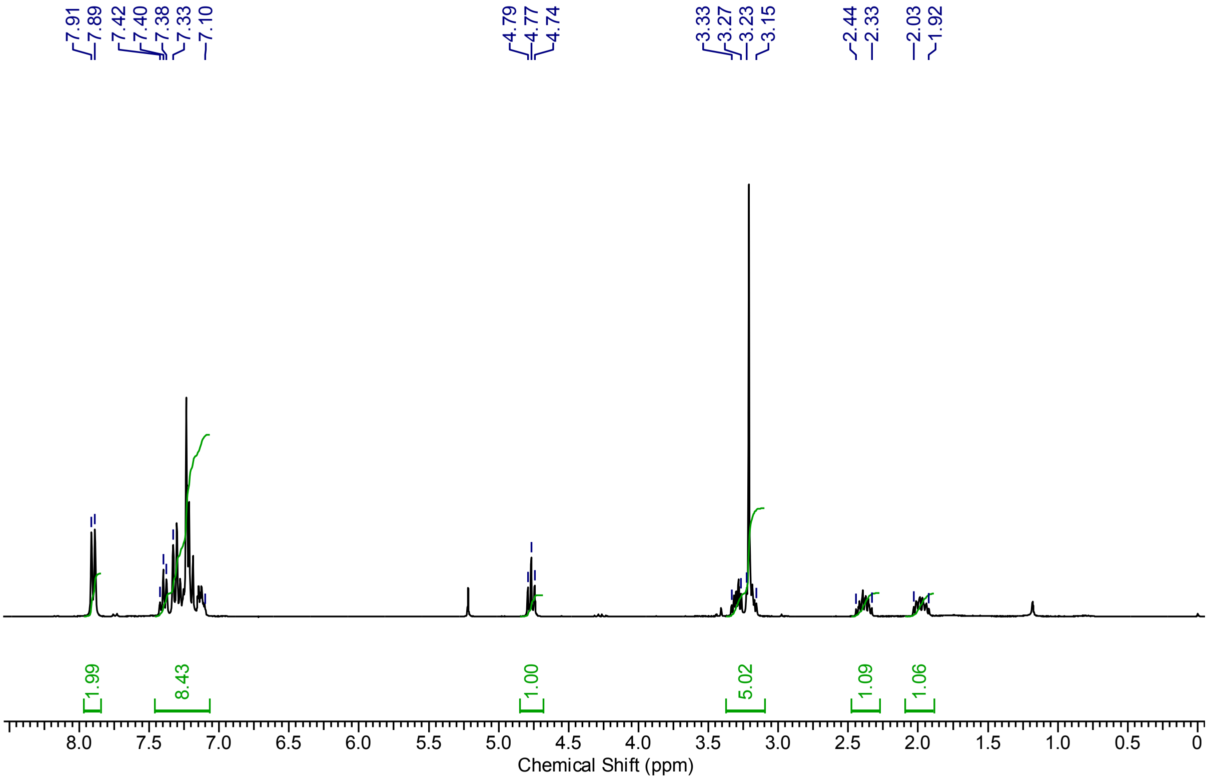
7.91
7.89
7.42
7.40
7.38
7.33
7.10
4.79
4.77
4.74
3.33
3.27
3.23
3.15
2.44
2.33
2.03
1.92
1.99
8.43
1.00
5.02
1.09
1.06
8.0 7.5 7.0 6.5 6.0 5.5 5.0 4.5 4.0 3.5 3.0 2.5 2.0 1.5 1.0 0.5 0
Chemical Shift (ppm)

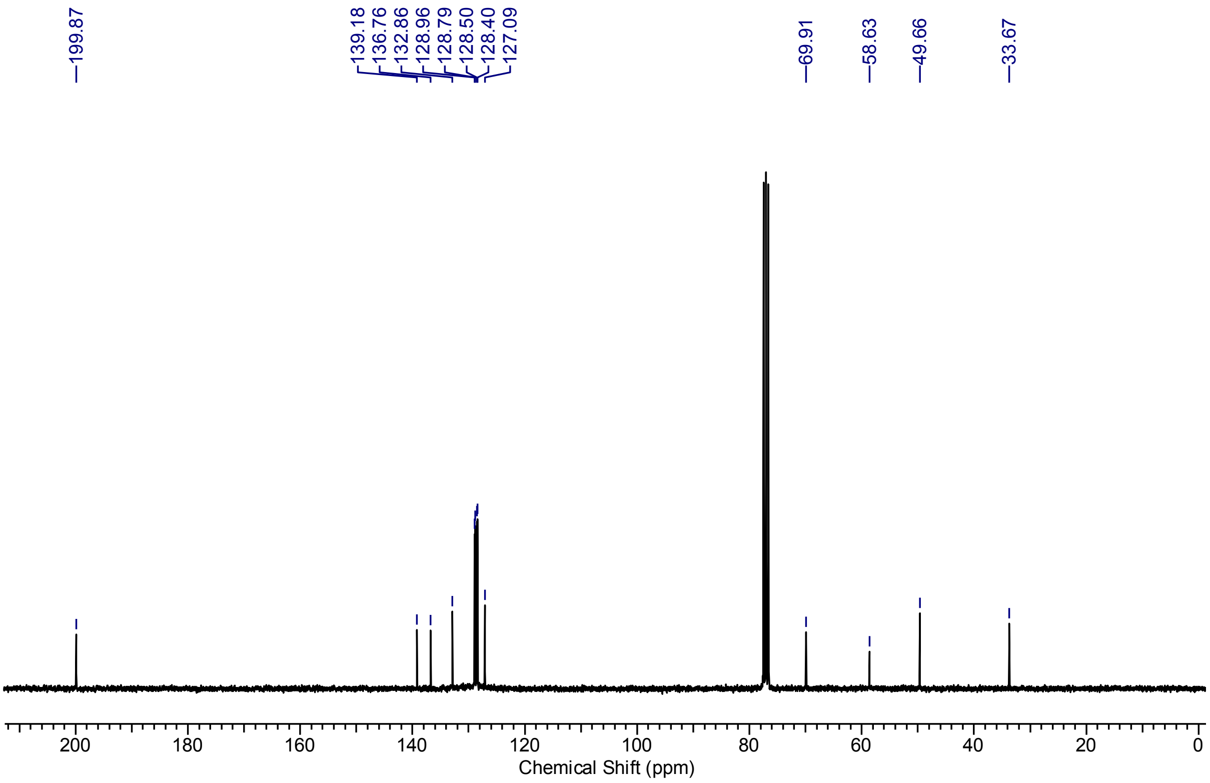
199.87
139.18
136.76
132.86
128.96
128.79
128.50
128.40
127.09
69.91
58.63
49.66
33.67
200 180 160 140 120 100 80 60 40 20 0
Chemical Shift (ppm)

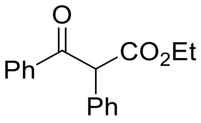
O
CO2Et
Ph
Ph

7e

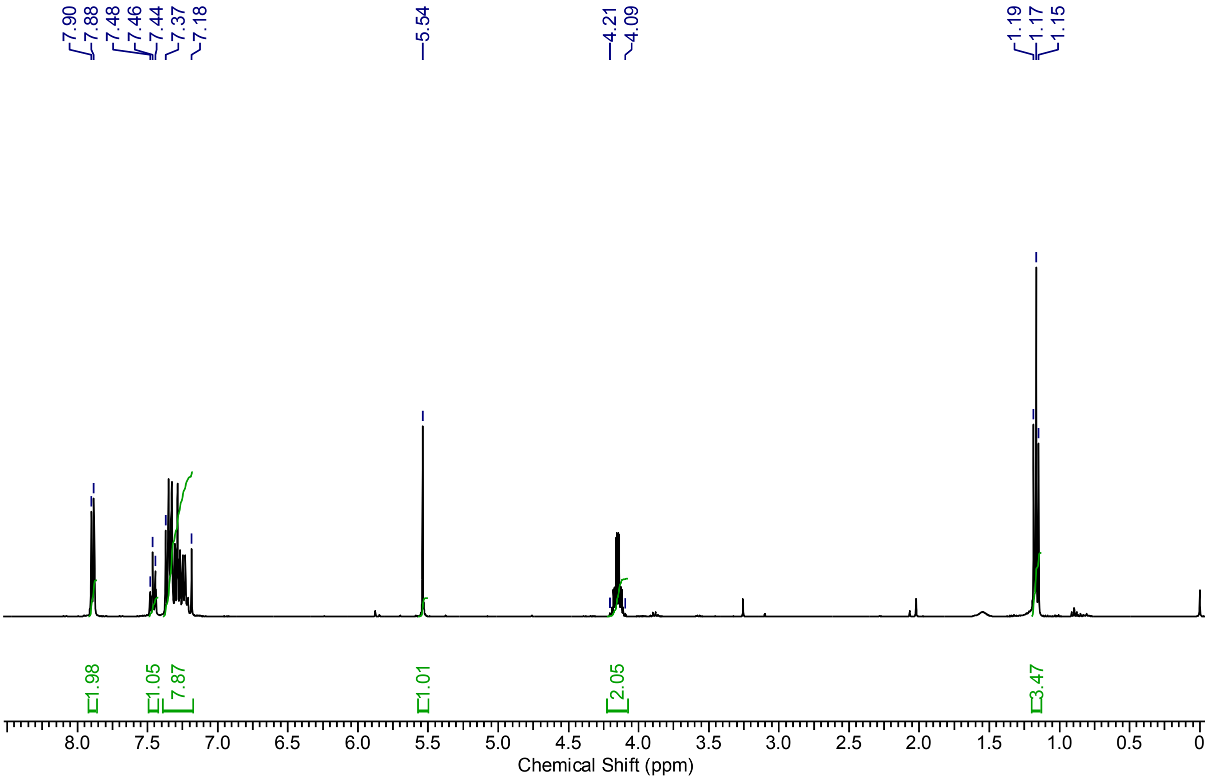
7.90
7.88
7.48
7.46
7.44
7.37
7.18
5.54
4.21
4.09
1.19
1.17
1.15
1.98
1.05
7.87
1.01
2.05
3.47
8.0
7.5
7.0
6.5
6.0
5.5
5.0
4.5
4.0
3.5
3.0
2.5
2.0
1.5
1.0
0.5
0
Chemical Shift (ppm)

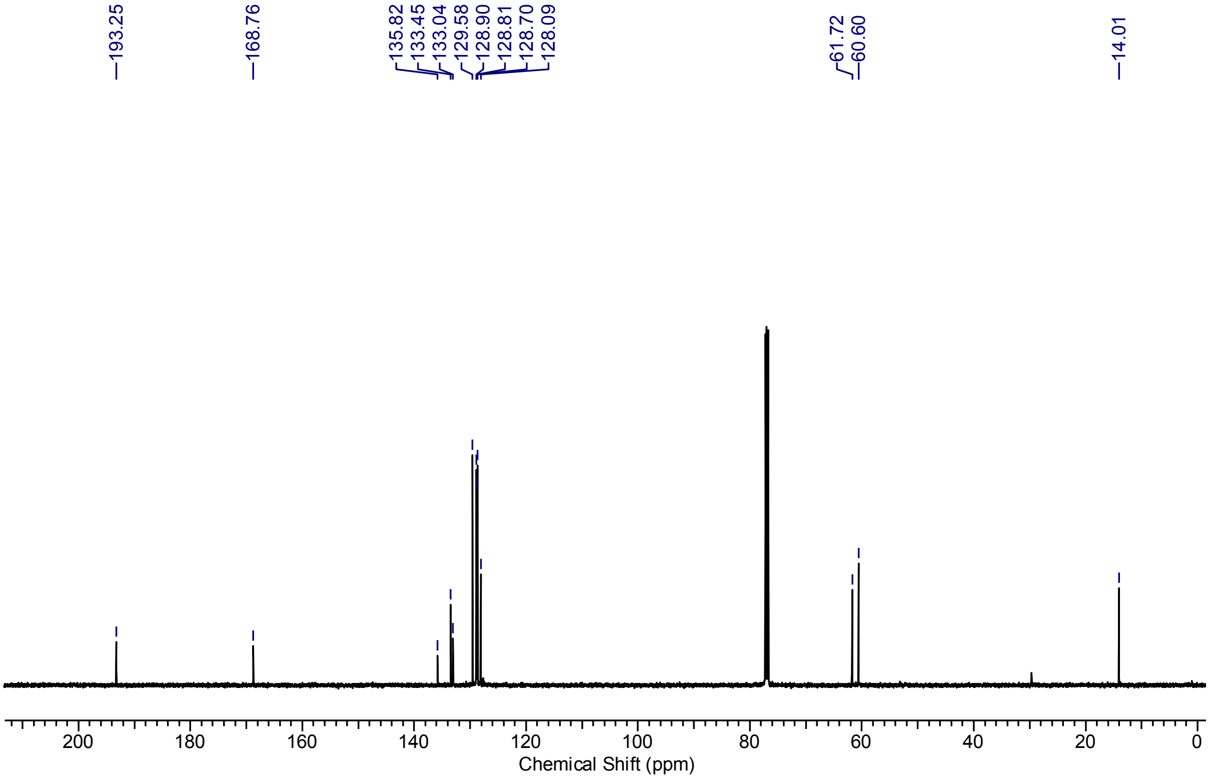
193.25
168.76
135.82
133.45
133.04
129.58
128.90
128.81
128.70
128.09
61.72
60.60
14.01
200
180
160
140
120
100
80
60
40
20
0
Chemical Shift (ppm)

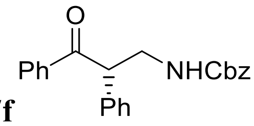
O
Ph
NHCbz
Ph

7f

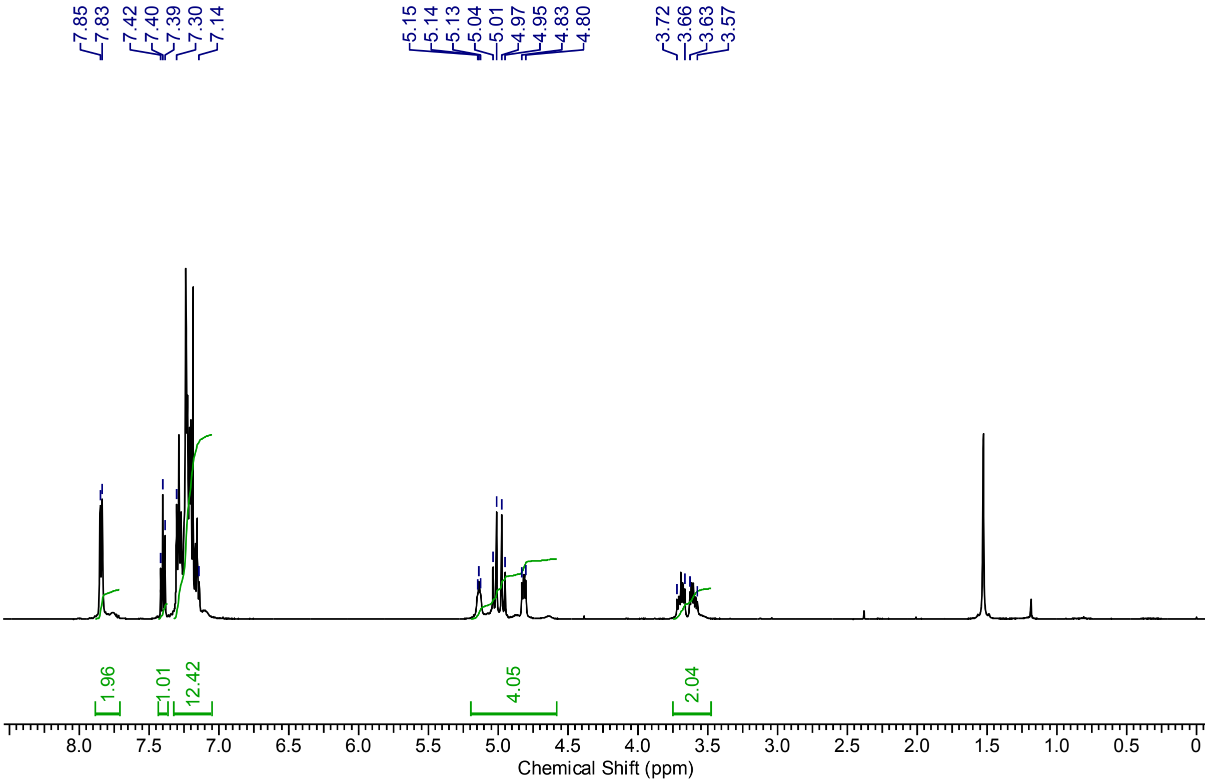
7.85
7.83
7.42
7.40
7.39
7.30
7.14
5.15
5.14
5.13
5.04
5.01
4.97
4.95
4.83
4.80
3.72
3.66
3.63
3.57
1.96
1.01
12.42
4.05
2.04
8.0
7.5
7.0
6.5
6.0
5.5
5.0
4.5
4.0
3.5
3.0
2.5
2.0
1.5
1.0
0.5
0
Chemical Shift (ppm)

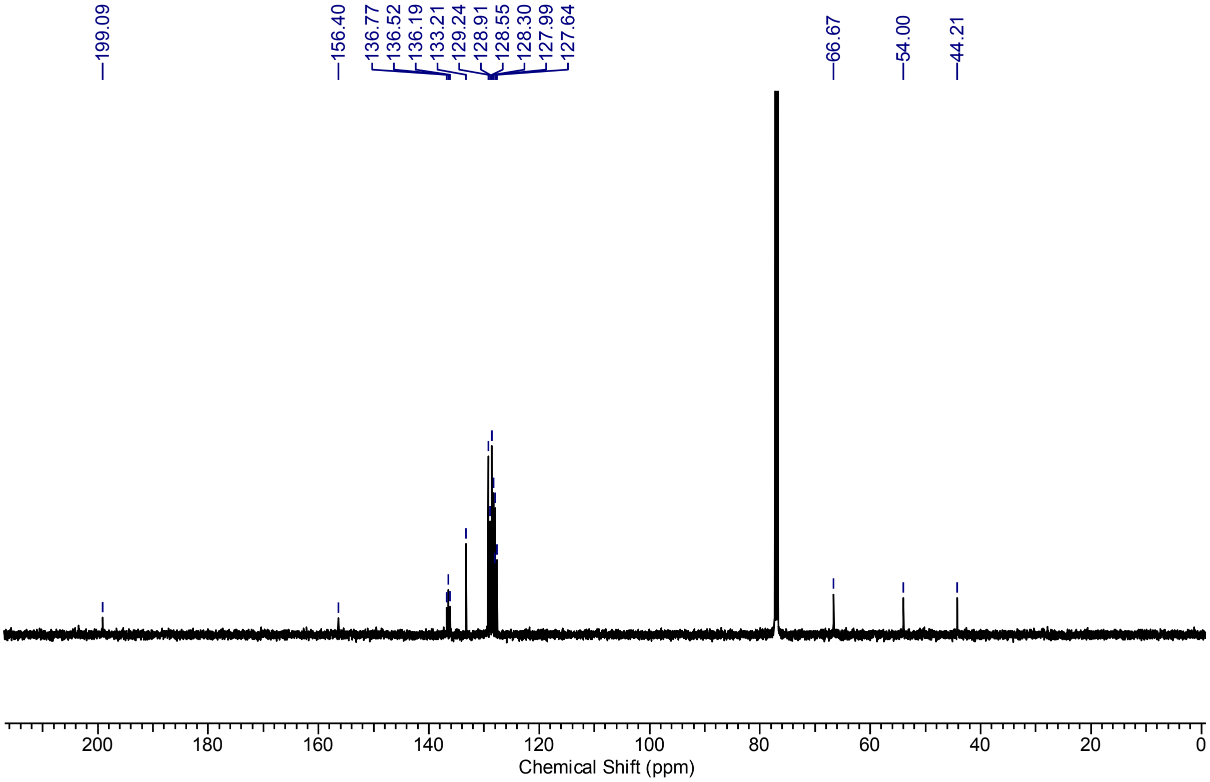
199.09
156.40
136.77
136.52
136.19
133.21
129.24
128.91
128.55
128.30
127.99
127.64
66.67
54.00
44.21
200
180
160
140
120
100
80
60
40
20
0
Chemical Shift (ppm)

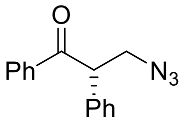
O
Ph
N3
Ph

7g

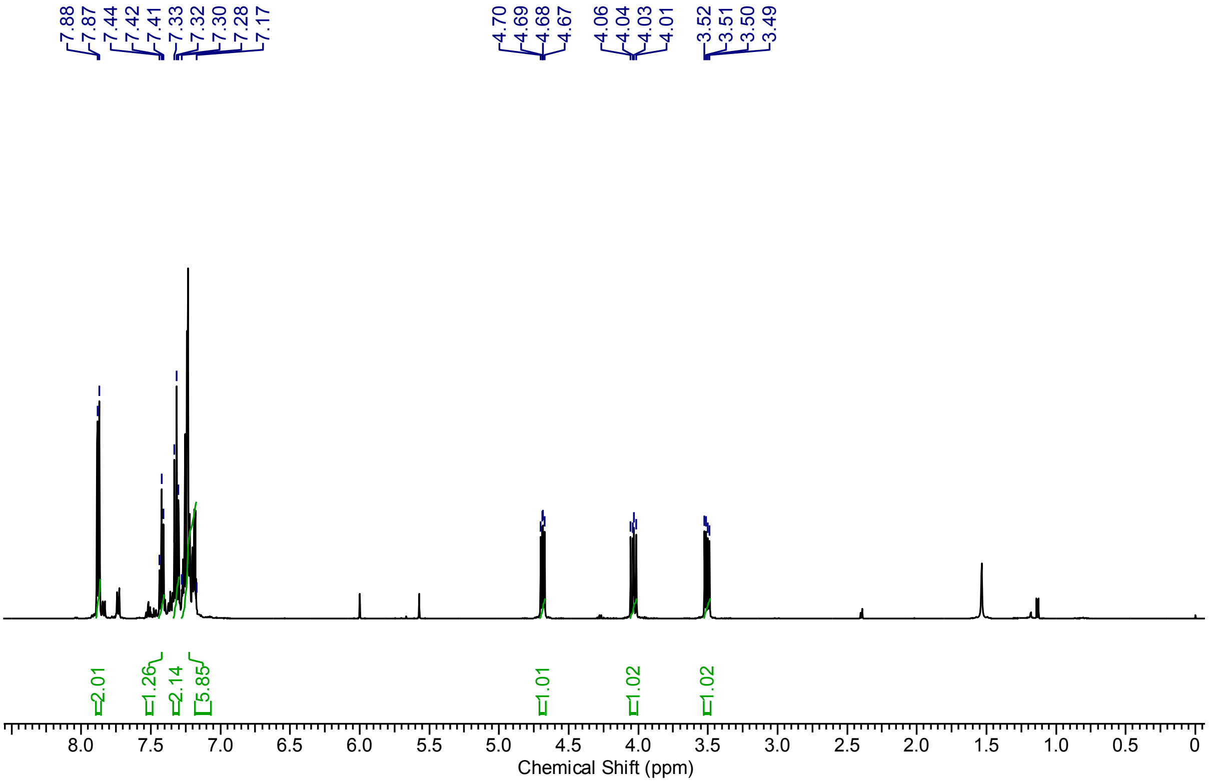
7.88
7.87
7.44
7.42
7.41
7.33
7.32
7.30
7.28
7.17
4.70
4.69
4.68
4.67
4.06
4.04
4.03
4.01
3.52
3.51
3.50
3.49
2.01
1.26
2.14
5.85
1.01
1.02
1.02
8.0
7.5
7.0
6.5
6.0
5.5
5.0
4.5
4.0
3.5
3.0
2.5
2.0
1.5
1.0
0.5
0
Chemical Shift (ppm)

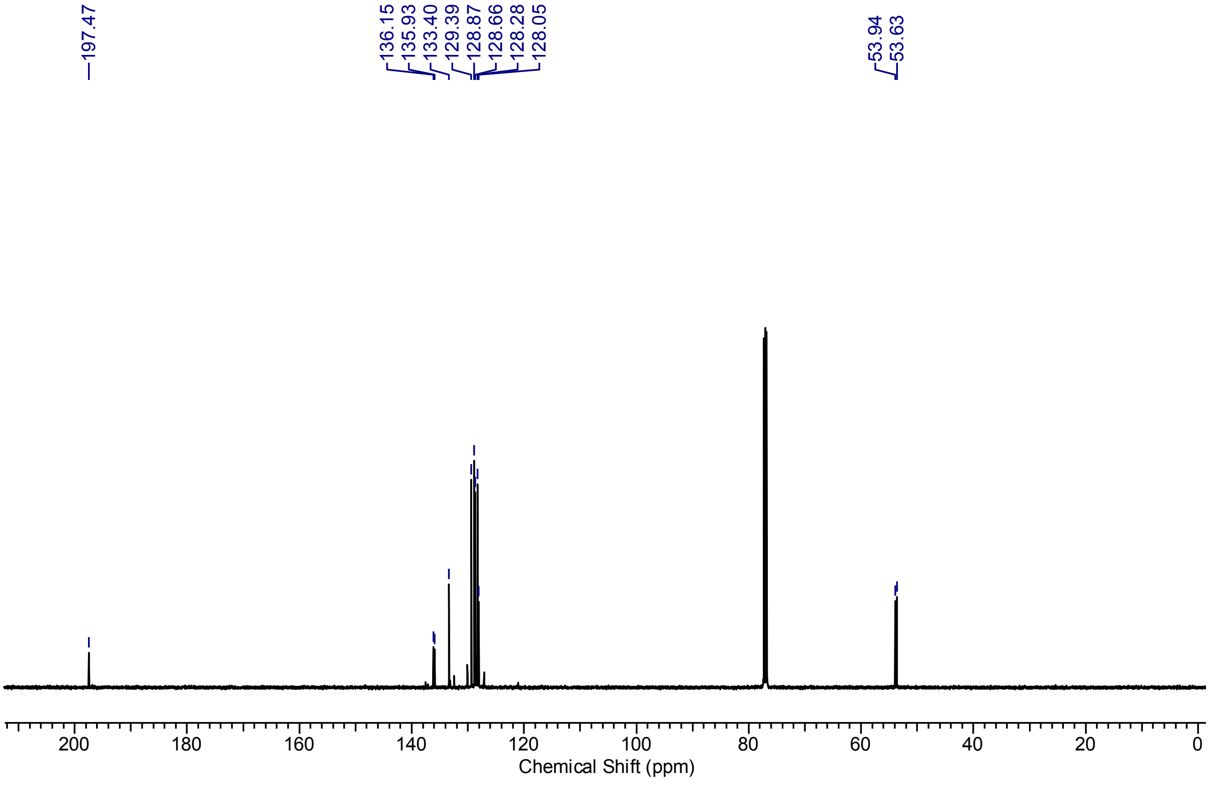
197.47
136.15
135.93
133.40
129.39
128.87
128.66
128.28
128.05
53.94
53.63
200
180
160
140
120
100
80
60
40
20
0
Chemical Shift (ppm)

7h

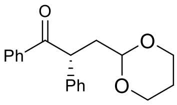
O
Ph
Ph
O
O

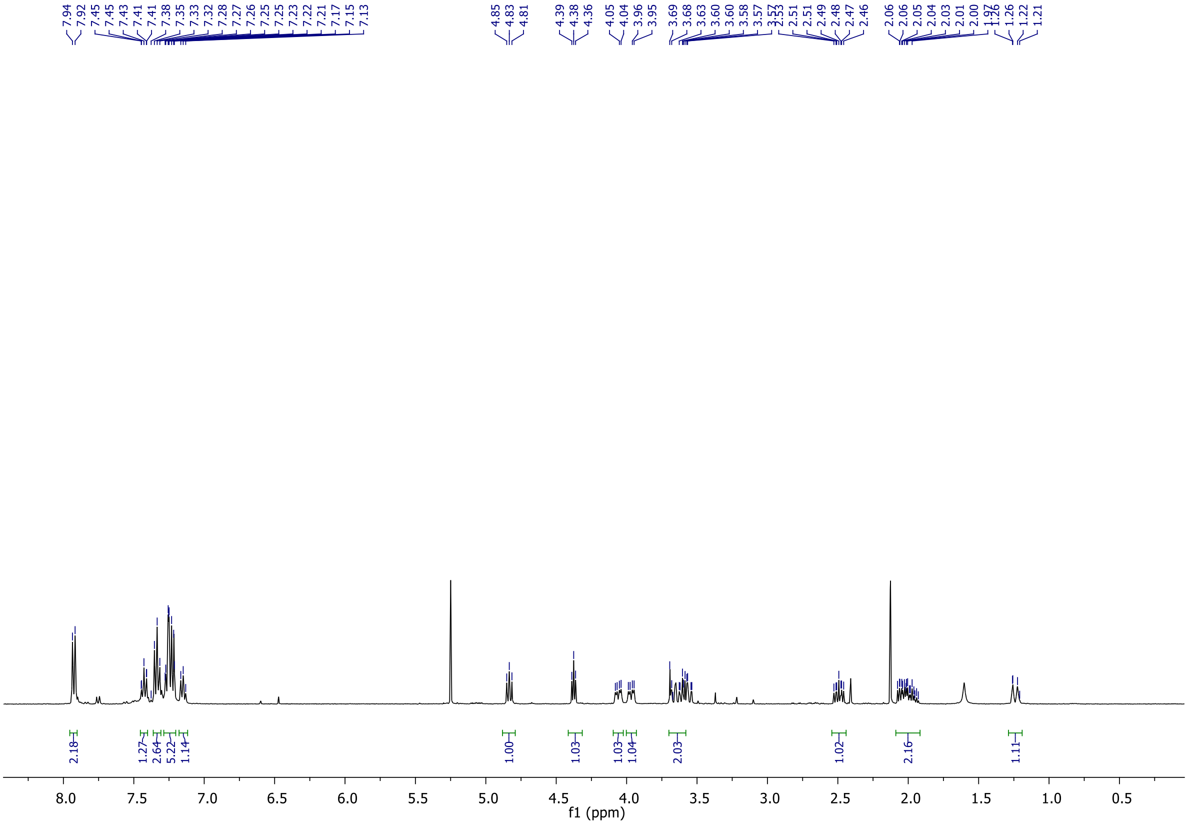
7.94 7.92 7.45 7.45 7.43 7.41 7.41 7.38 7.35 7.33 7.32 7.28 7.27 7.26 7.25 7.25 7.23 7.22 7.21 7.17 7.15 7.13
4.85 4.83 4.81 4.39 4.38 4.36 4.05 4.04 3.96 3.95 3.69 3.68 3.63 3.60 3.60 3.58 3.57 2.57 2.53 2.51 2.51 2.49 2.48 2.47 2.46
2.06 2.06 2.05 2.04 2.03 2.01 2.00 1.97 1.26 1.22 1.21
2.18 1.27 2.64 5.22 1.14 1.00 1.03 1.03 1.04 2.03 1.02 2.16 1.11
8.0 7.5 7.0 6.5 6.0 5.5 5.0 4.5 4.0 3.5 3.0 2.5 2.0 1.5 1.0 0.5
f1 (ppm)

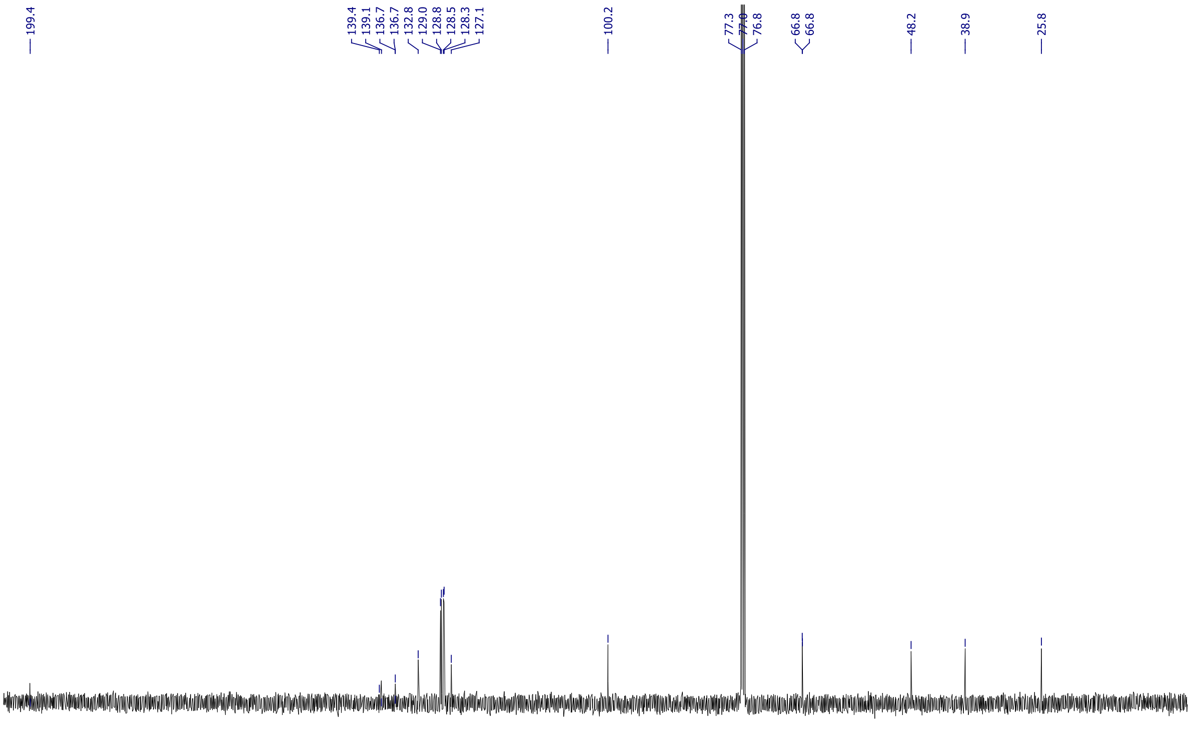
199.4
139.4 139.1 136.7 136.7 132.8 129.0 128.8 128.5 128.3 127.1
100.2
77.3 77.0 76.8
66.8 66.8
48.2
38.9
25.8
200 190 180 170 160 150 140 130 120 110 100 90 80 70 60 50 40 30 20 10
chemical shift (ppm)

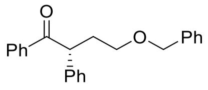
O
Ph
O
Ph
Ph

7i

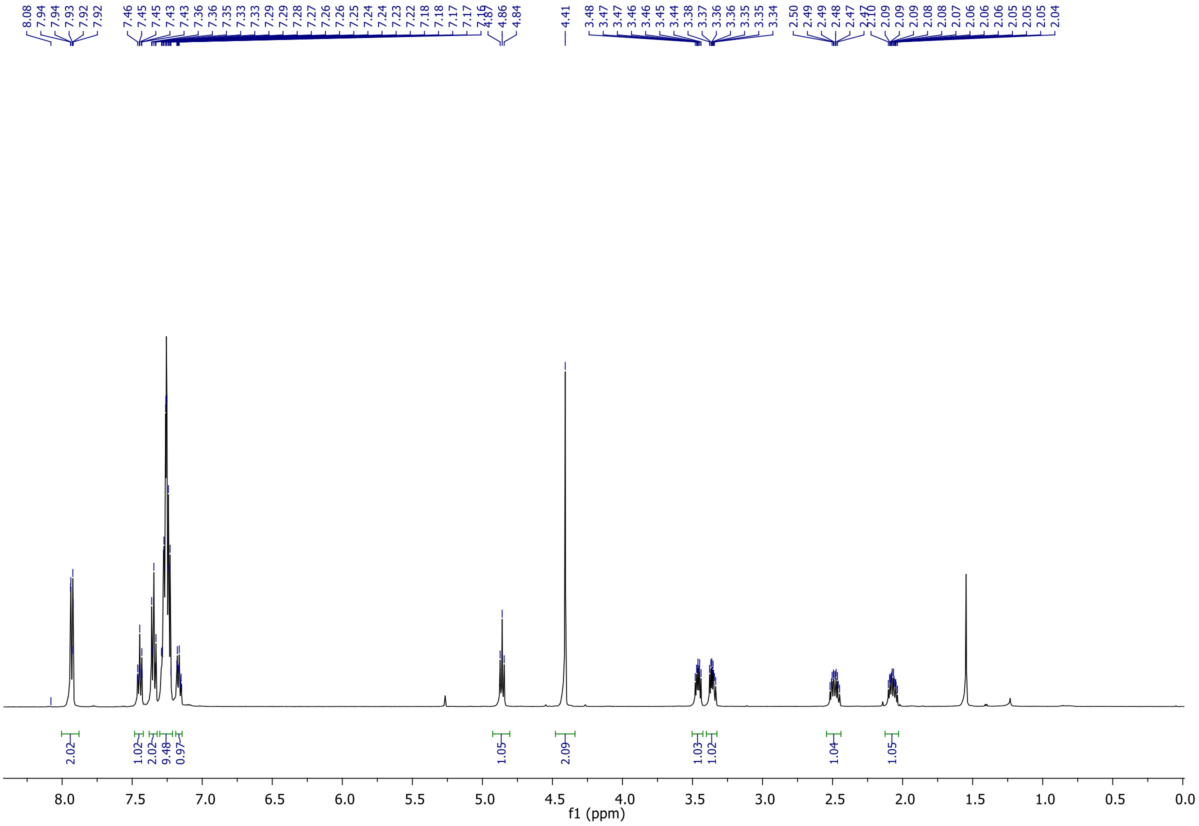
2.02
1.02
2.02
9.48
0.97
1.05
2.09
1.03
1.02
1.04
1.05
f1 (ppm)

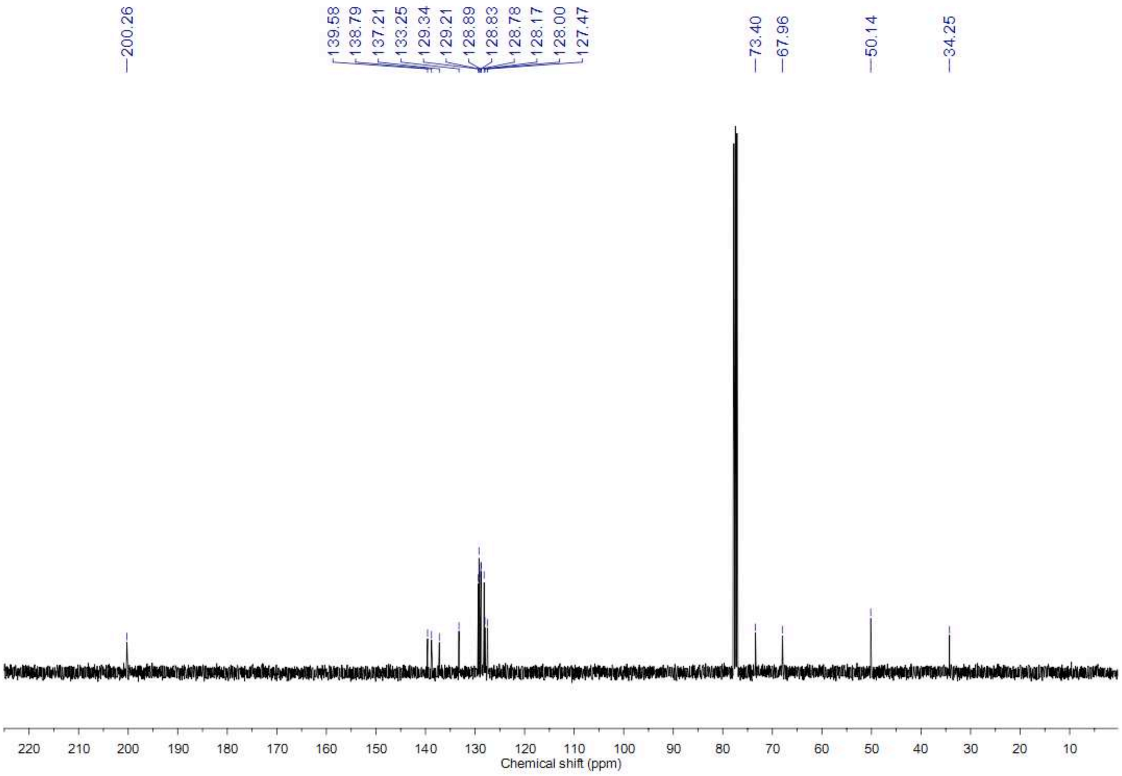
200.26
139.58
138.79
137.21
133.25
129.34
129.21
128.89
128.83
128.78
128.17
128.00
127.47
73.40
67.96
50.14
34.25
Chemical shift (ppm)

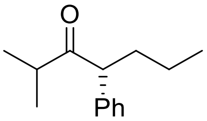
O
Ph

7j

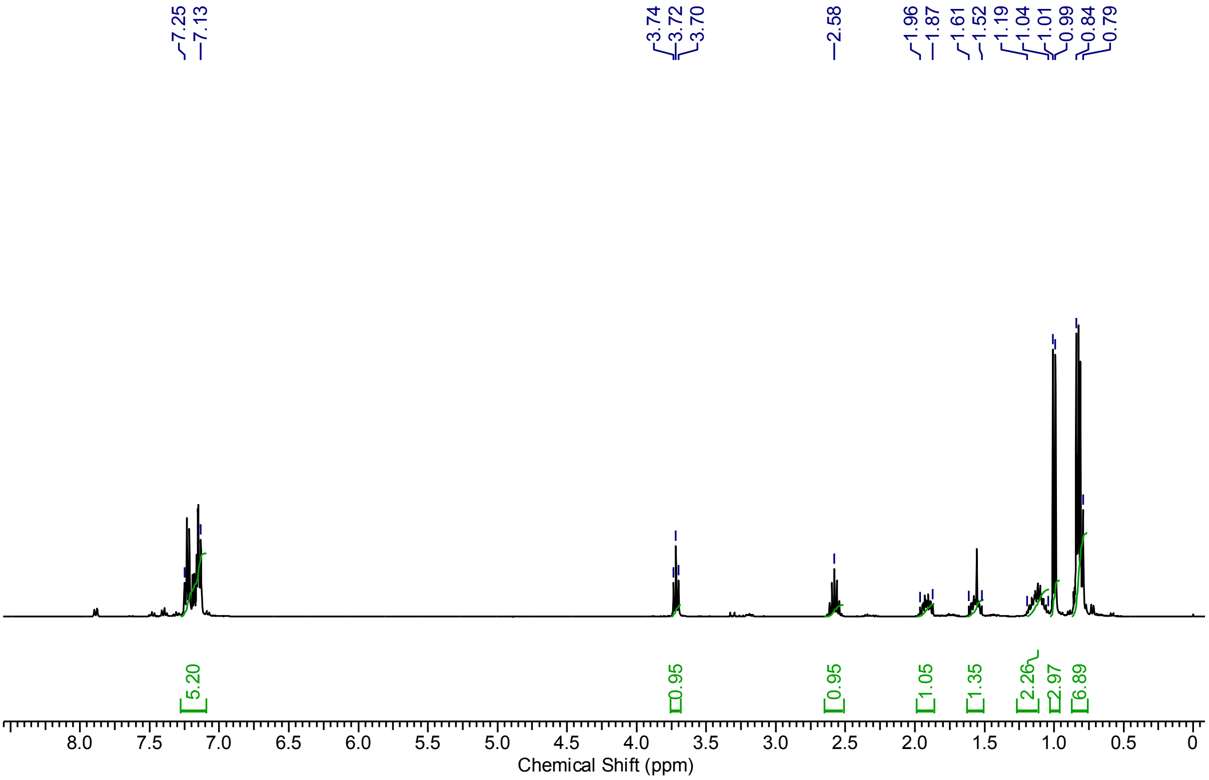
7.25
7.13
3.74
3.72
3.70
2.58
1.96
1.87
1.61
1.52
1.19
1.04
1.01
0.99
0.84
0.79
5.20
0.95
0.95
1.05
1.35
2.26
2.97
6.89
8.0
7.5
7.0
6.5
6.0
5.5
5.0
4.5
4.0
3.5
3.0
2.5
2.0
1.5
1.0
0.5
0
Chemical Shift (ppm)

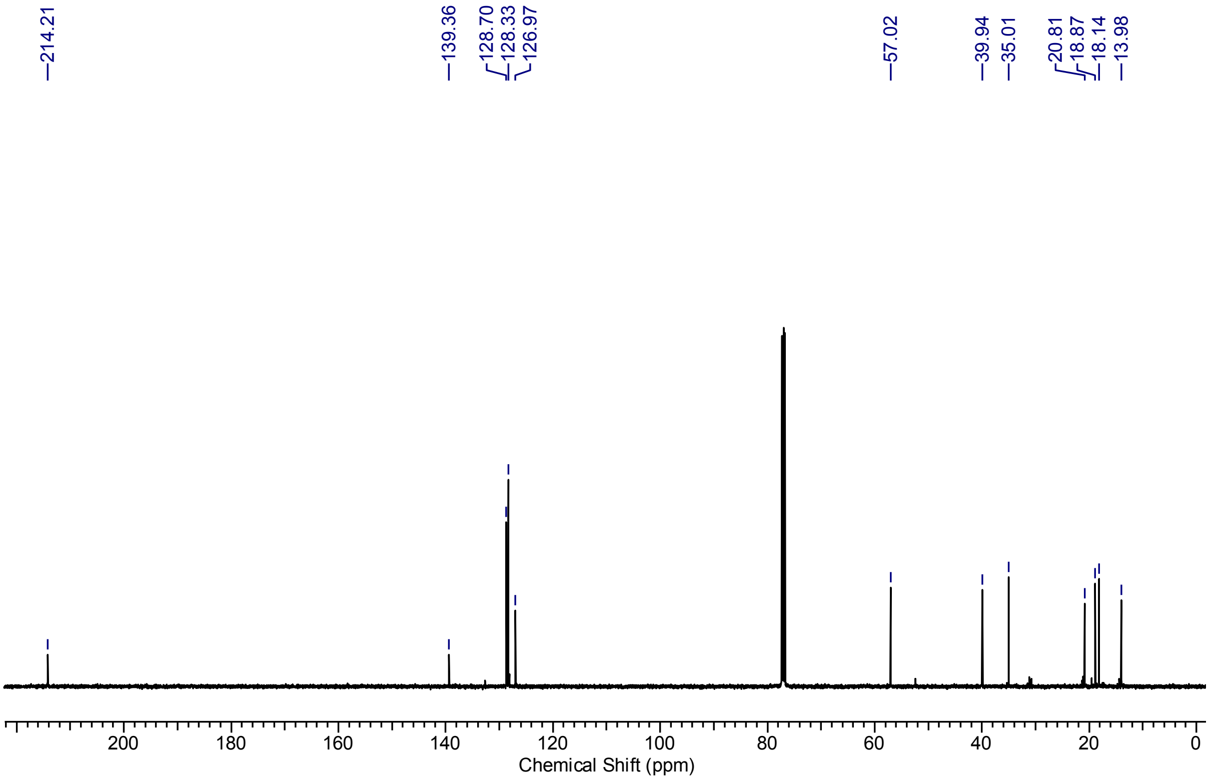
214.21
139.36
128.70
128.33
126.97
57.02
39.94
35.01
20.81
18.87
18.14
13.98
200
180
160
140
120
100
80
60
40
20
0
Chemical Shift (ppm)

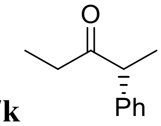
O
Ph

7k

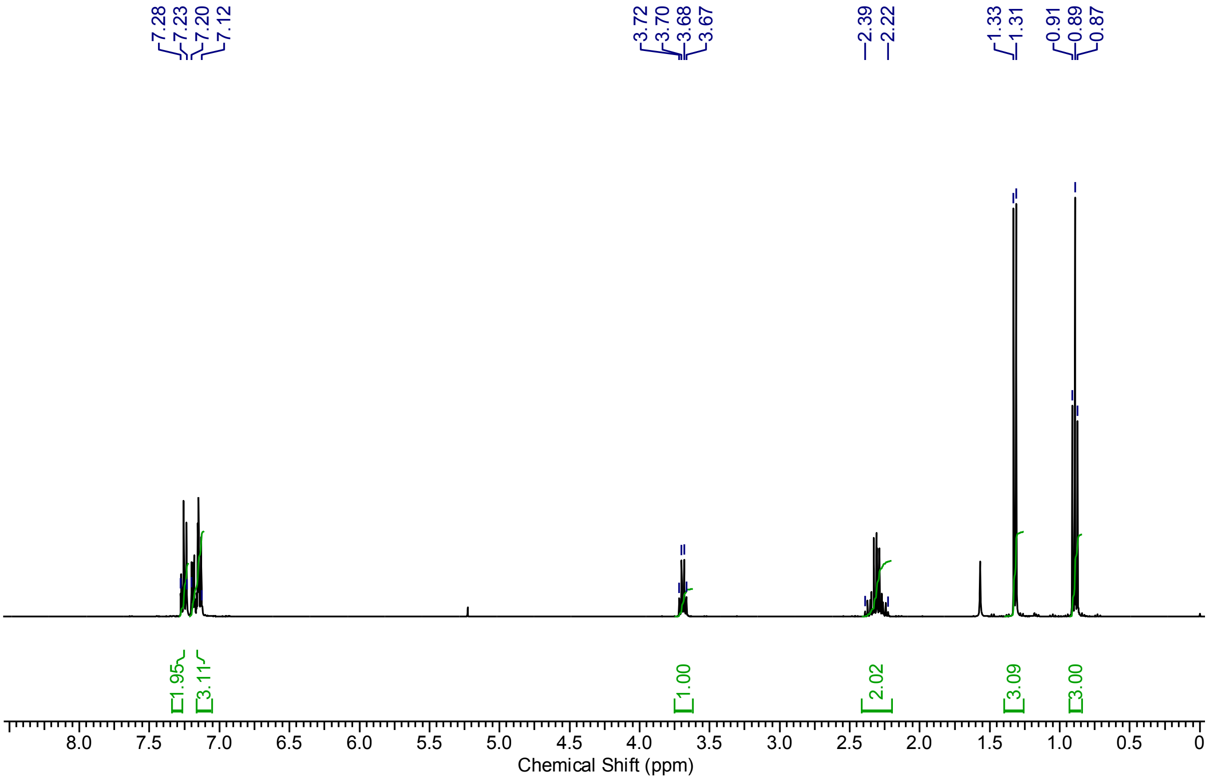
7.28
7.23
7.20
7.12
3.72
3.70
3.68
3.67
2.39
2.22
1.33
1.31
0.91
0.89
0.87
1.95
3.11
1.00
2.02
3.09
3.00
8.0 7.5 7.0 6.5 6.0 5.5 5.0 4.5 4.0 3.5 3.0 2.5 2.0 1.5 1.0 0.5 0
Chemical Shift (ppm)

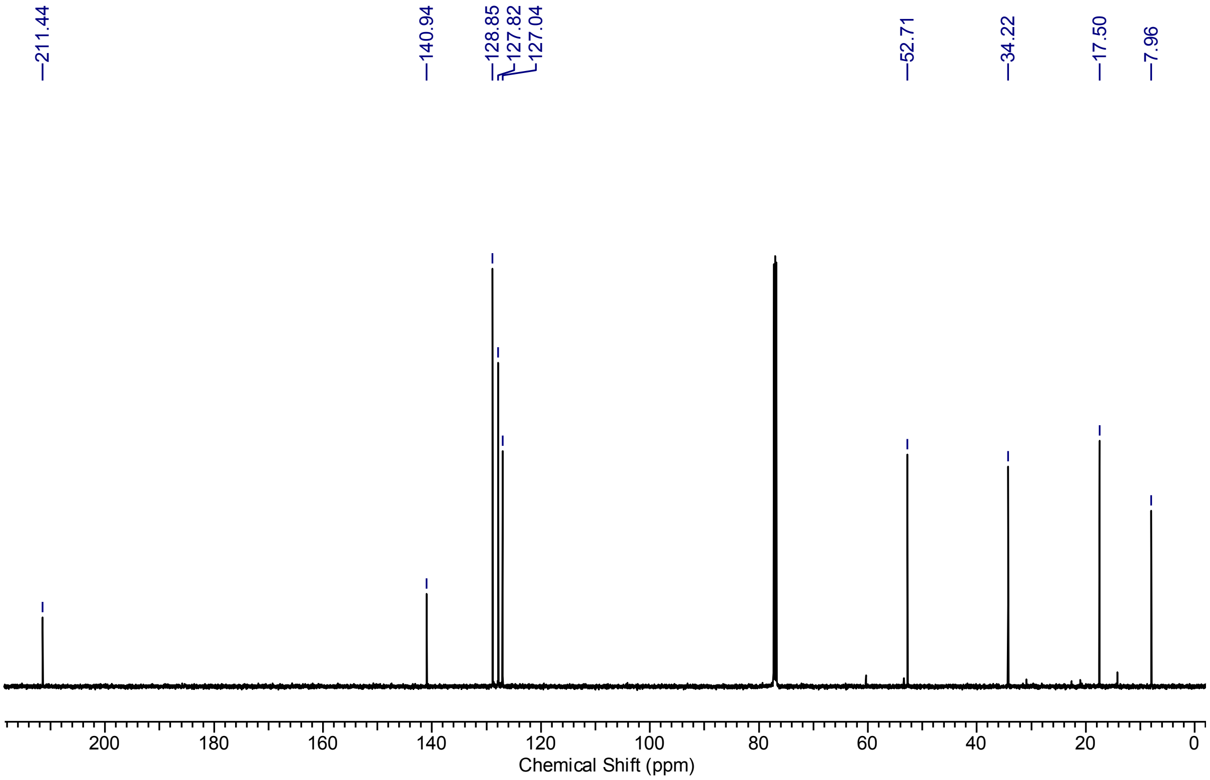
211.44
140.94
128.85
127.82
127.04
52.71
34.22
17.50
7.96
200 180 160 140 120 100 80 60 40 20 0
Chemical Shift (ppm)

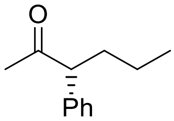

7l

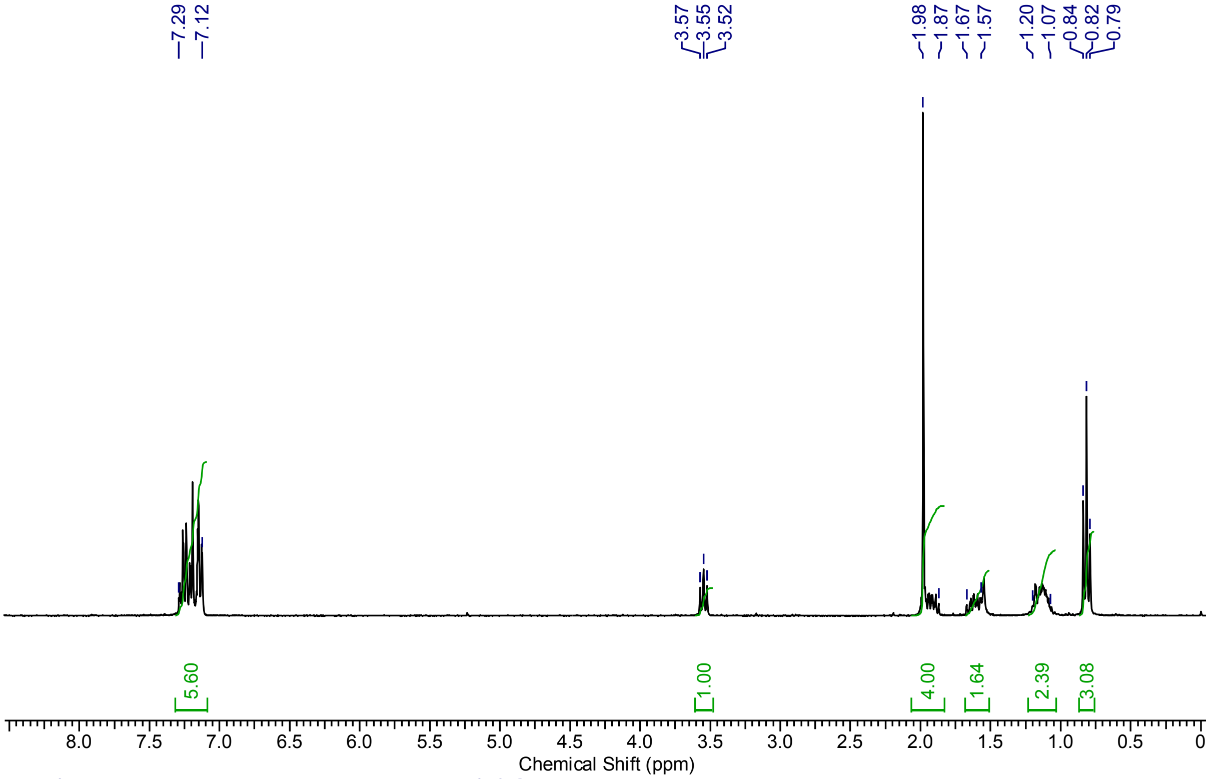

7.29
7.12
3.57
3.55
3.52
1.98
1.87
1.67
1.57
1.20
1.07
0.84
0.82
0.79
5.60
1.00
4.00
1.64
2.39
3.08
Chemical Shift (ppm)

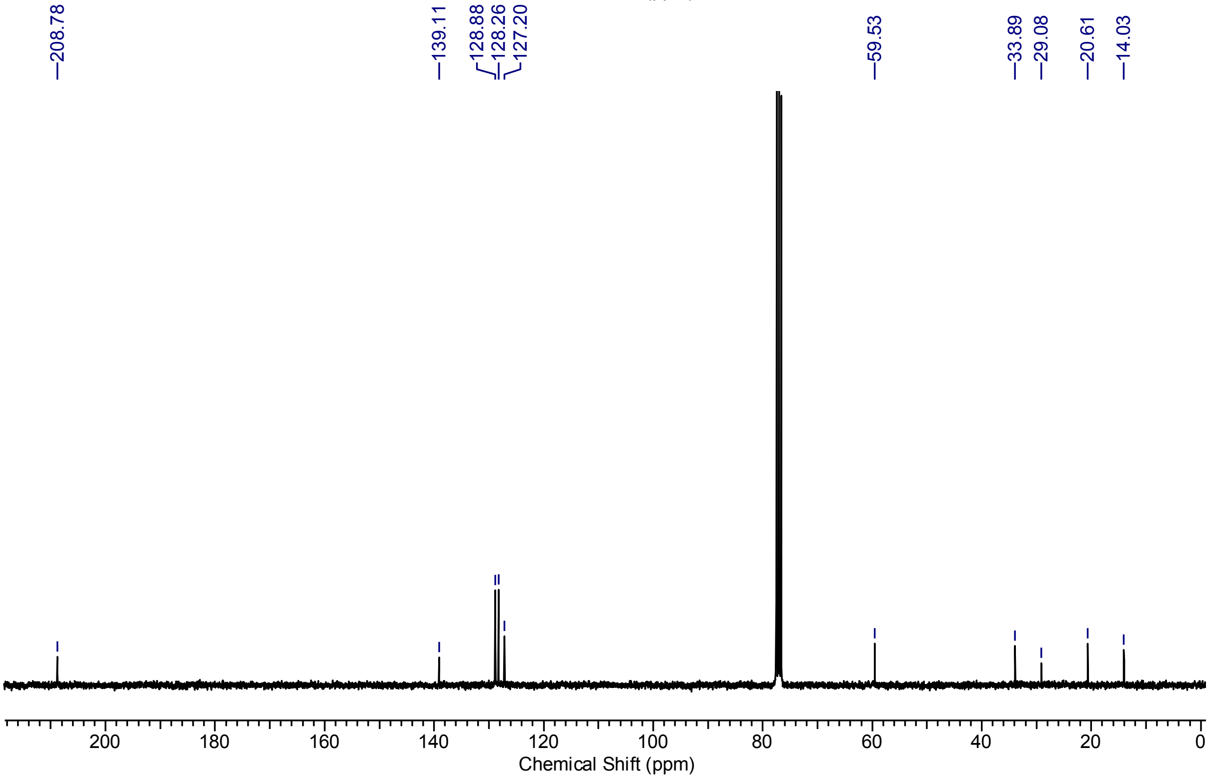

208.78
139.11
128.88
128.26
127.20
59.53
33.89
29.08
20.61
14.03
Chemical Shift (ppm)

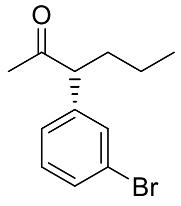
O
Br

7m

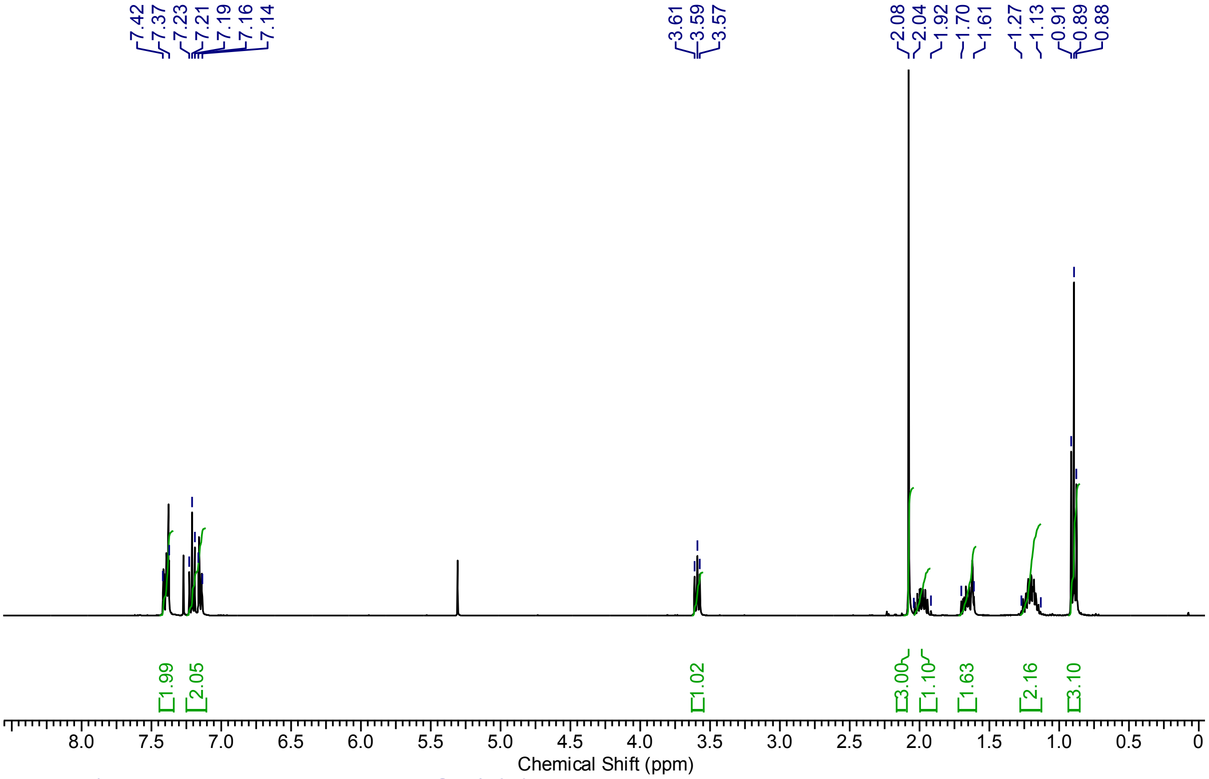
7.42
7.37
7.23
7.21
7.19
7.16
7.14
3.61
3.59
3.57
2.08
2.04
1.92
1.70
1.61
1.27
1.13
0.91
0.89
0.88
1.99
2.05
1.02
3.00
1.10
1.63
2.16
3.10
8.0
7.5
7.0
6.5
6.0
5.5
5.0
4.5
4.0
3.5
3.0
2.5
2.0
1.5
1.0
0.5
0
Chemical Shift (ppm)

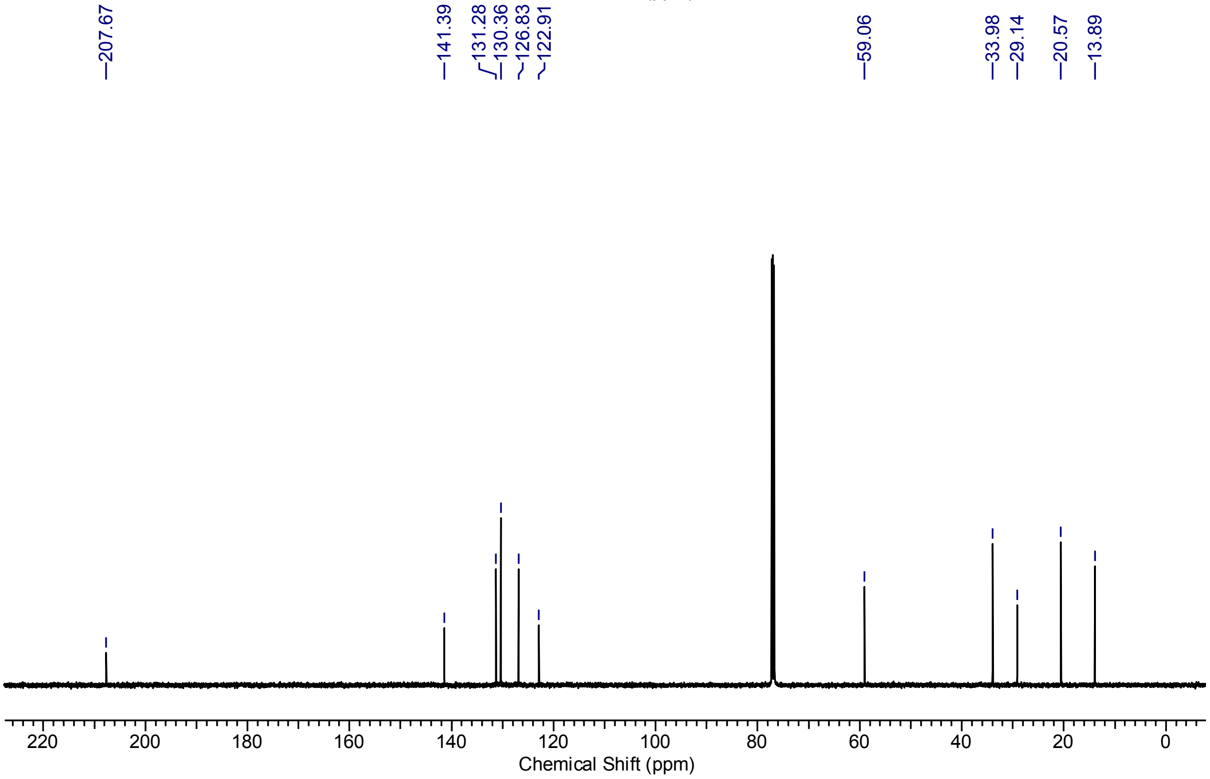
207.67
141.39
131.28
130.36
126.83
122.91
59.06
33.98
29.14
20.57
13.89
220
200
180
160
140
120
100
80
60
40
20
0
Chemical Shift (ppm)

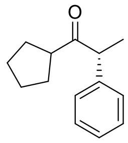

7n

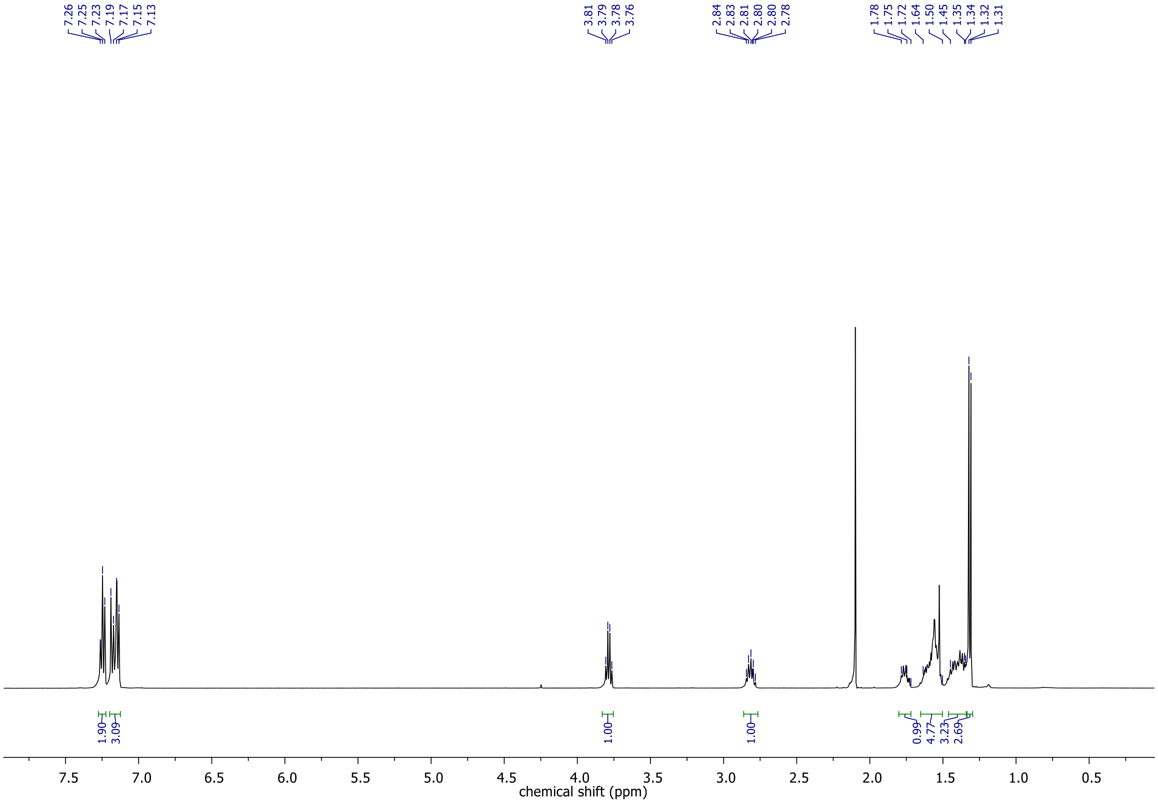
7.26 7.25 7.23 7.19 7.17 7.15 7.13
3.81 3.79 3.78 3.76
2.84 2.83 2.81 2.80 2.80 2.78
1.78 1.75 1.72 1.64 1.50 1.45 1.35 1.34 1.32 1.31
1.90 3.09
1.00
1.00
0.99 4.77 3.23 2.69
7.5 7.0 6.5 6.0 5.5 5.0 4.5 4.0 3.5 3.0 2.5 2.0 1.5 1.0 0.5
chemical shift (ppm)

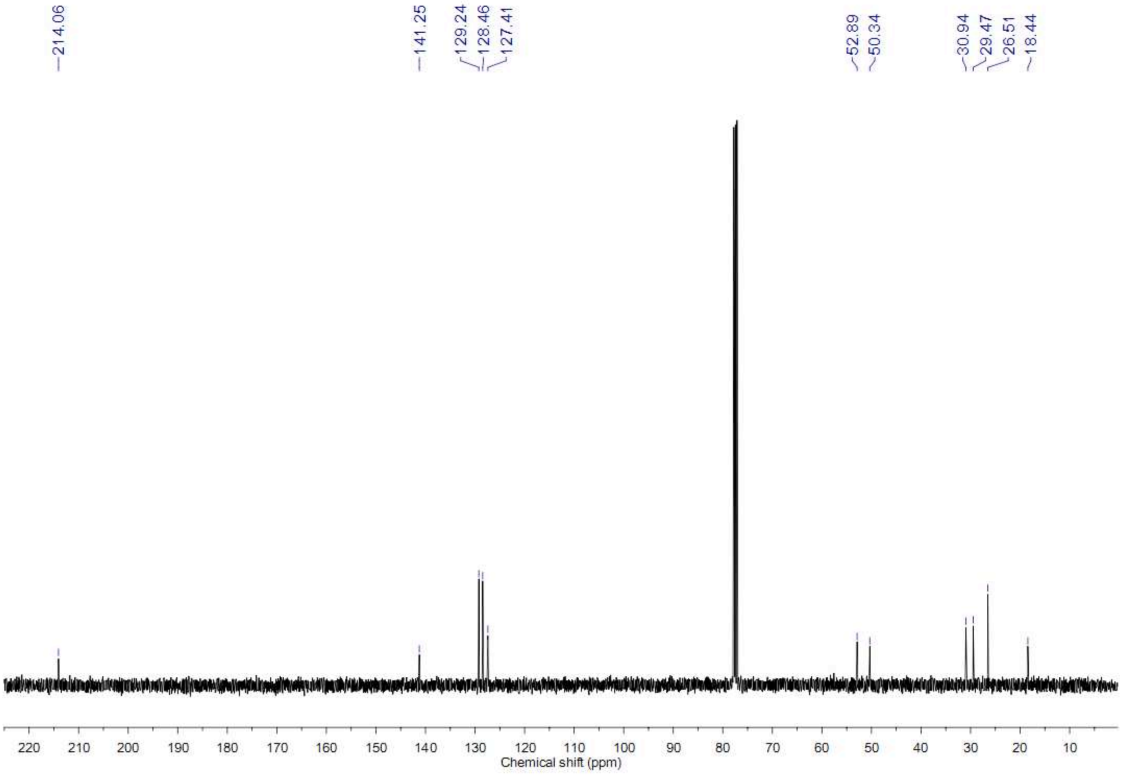
214.06
141.25
129.24
128.46
127.41
52.89
50.34
30.94
29.47
26.51
18.44
220 210 200 190 180 170 160 150 140 130 120 110 100 90 80 70 60 50 40 30 20 10
Chemical shift (ppm)

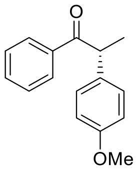
O
OMe

7o

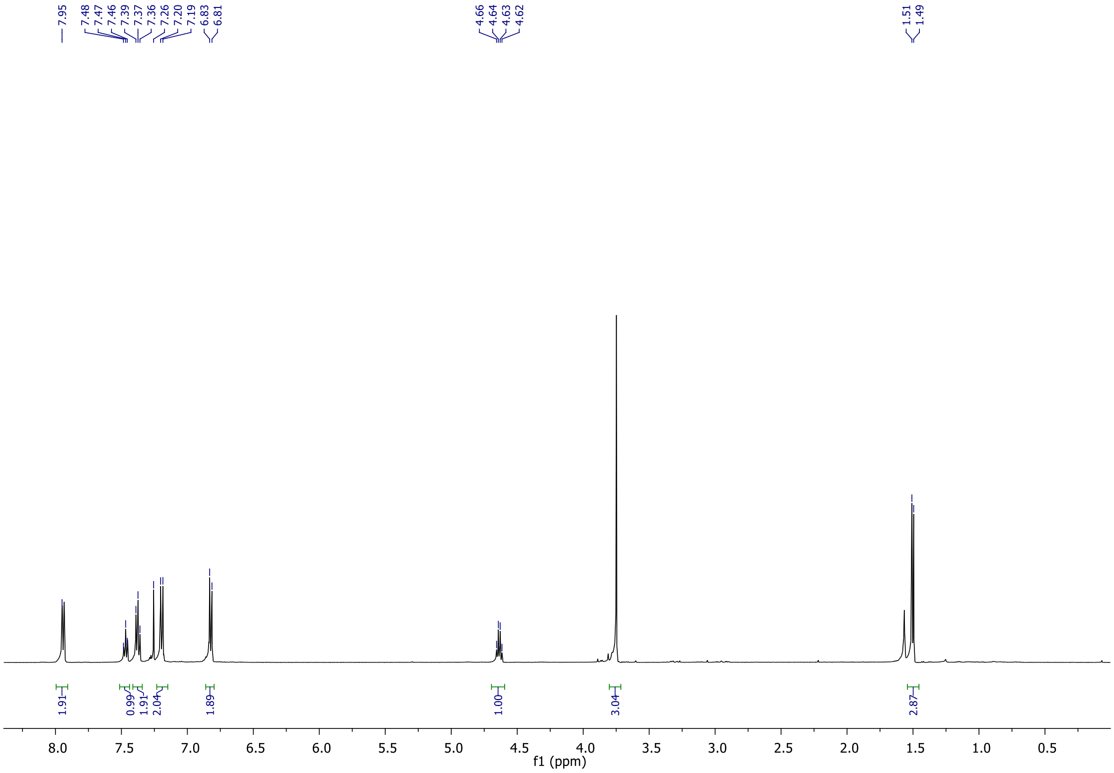
7.95
7.48
7.47
7.46
7.39
7.37
7.36
7.26
7.20
7.19
6.83
6.81
4.66
4.64
4.63
4.62
1.51
1.49
1.91
0.99
1.91
2.04
1.89
1.00
3.04
2.87
8.0 7.5 7.0 6.5 6.0 5.5 5.0 4.5 4.0 3.5 3.0 2.5 2.0 1.5 1.0 0.5
f1 (ppm)

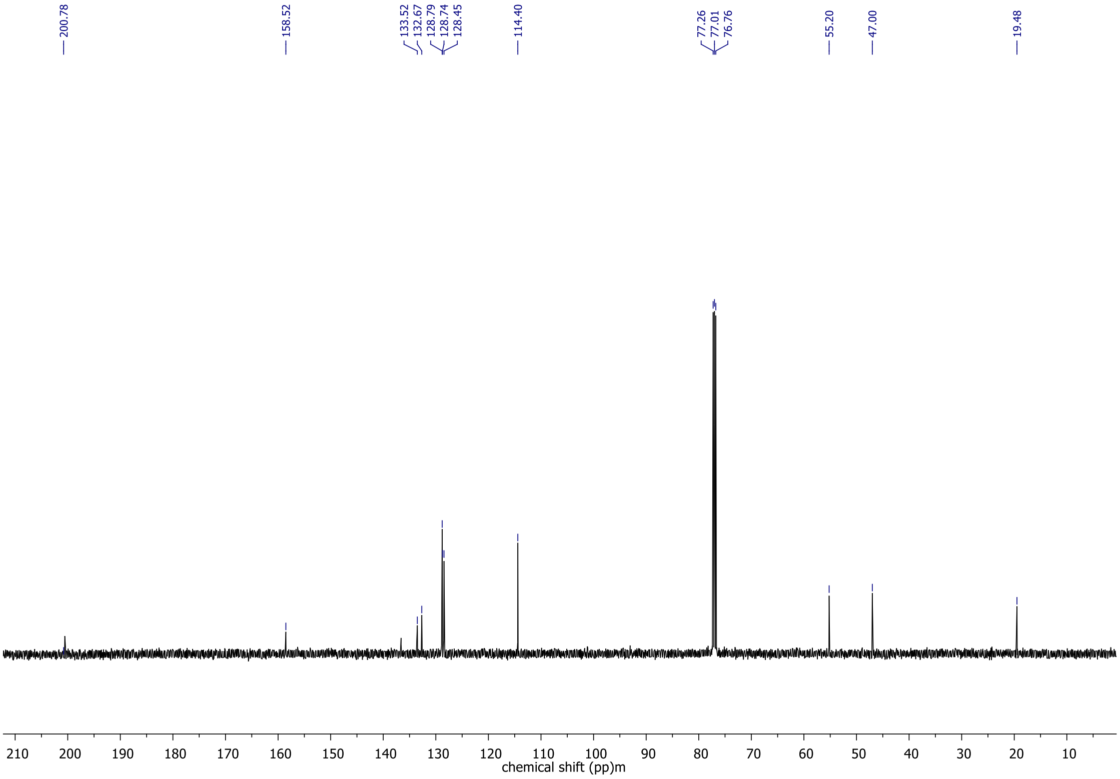
200.78
158.52
133.52
132.67
128.79
128.74
128.45
114.40
77.26
77.01
76.76
55.20
47.00
19.48
210 200 190 180 170 160 150 140 130 120 110 100 90 80 70 60 50 40 30 20 10
chemical shift (pp)m

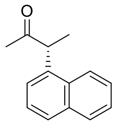
O

7p

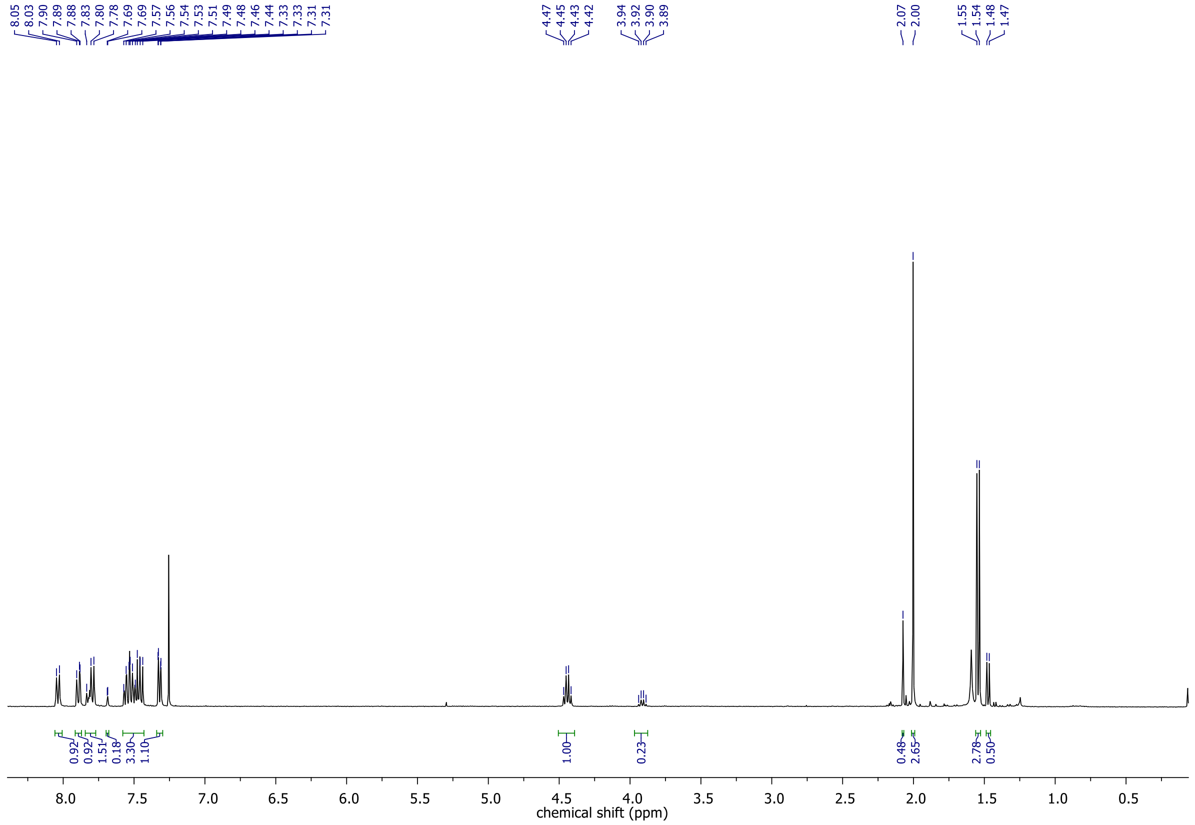
8.05 8.03 7.90 7.89 7.88 7.83 7.80 7.78 7.69 7.69 7.57 7.56 7.54 7.53 7.51 7.49 7.48 7.46 7.44 7.33 7.33 7.31 7.31
4.47 4.45 4.43 4.42 3.94 3.92 3.90 3.89
2.07 2.00
1.55 1.54 1.48 1.47
0.92 0.92 1.51 0.18 3.30 1.10
1.00
0.23
0.48 2.65
2.78 0.50
8.0 7.5 7.0 6.5 6.0 5.5 5.0 4.5 4.0 3.5 3.0 2.5 2.0 1.5 1.0 0.5
chemical shift (ppm)

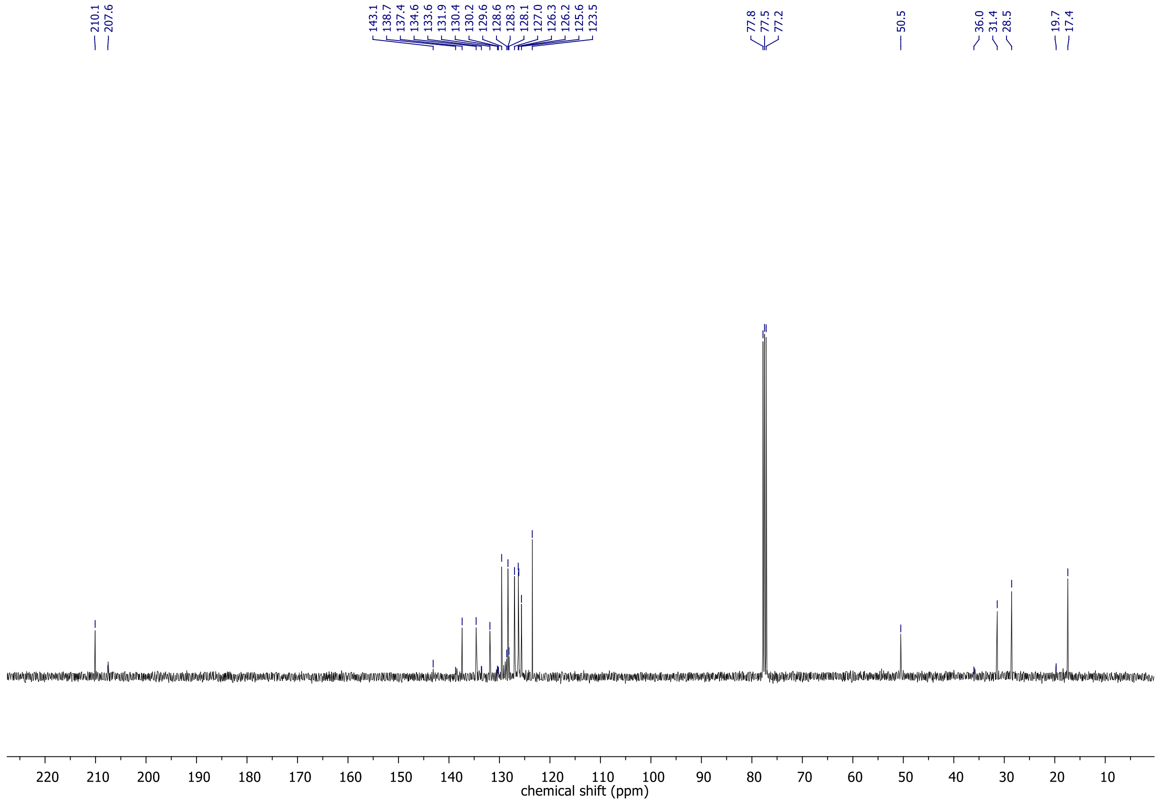
210.1 207.6
143.1 138.7 137.4 134.6 133.6 131.9 130.4 130.2 129.6 128.6 128.3 128.1 127.0 126.3 126.2 125.6 123.5
77.8 77.5 77.2
50.5
36.0 31.4 28.5
19.7 17.4
220 210 200 190 180 170 160 150 140 130 120 110 100 90 80 70 60 50 40 30 20 10
chemical shift (ppm)

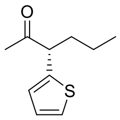
O
S

7q

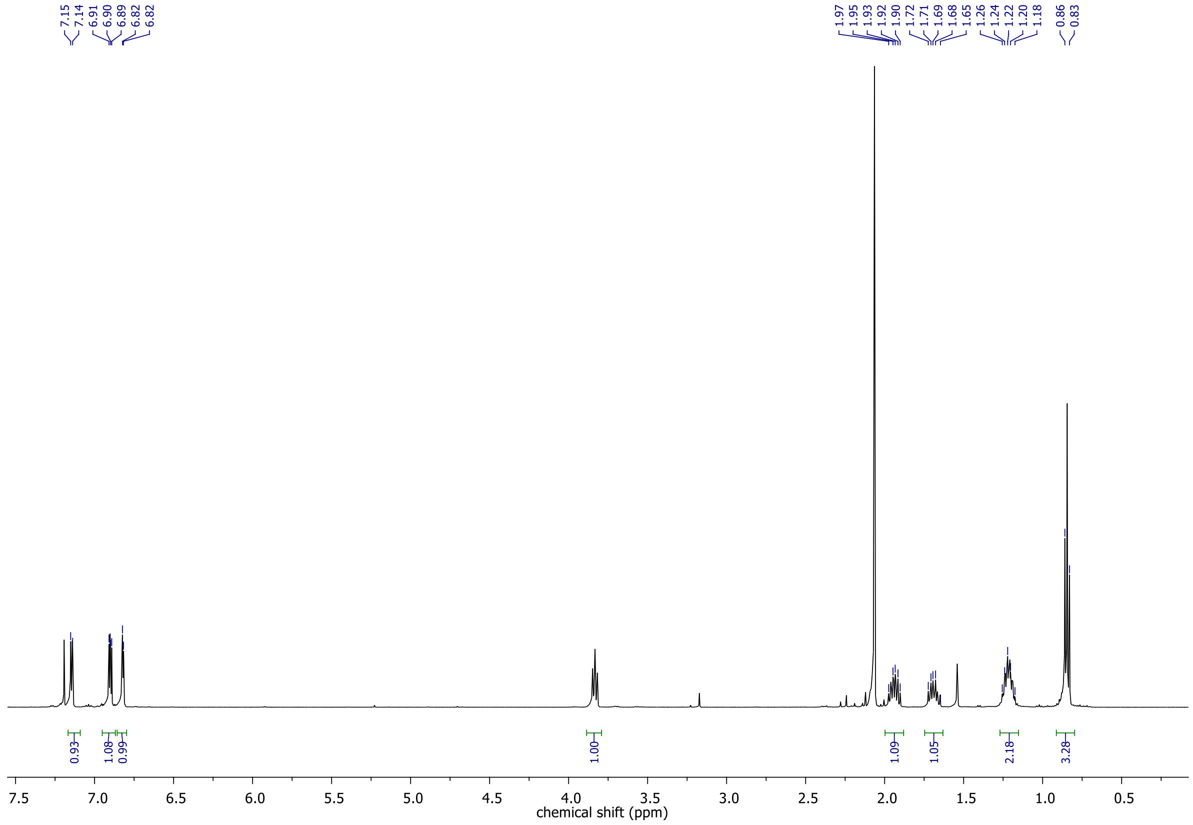
7.15
7.14
6.91
6.90
6.89
6.82
6.82
1.97
1.95
1.93
1.92
1.90
1.72
1.71
1.69
1.68
1.65
1.26
1.24
1.22
1.20
1.18
0.86
0.83
0.93
1.08
0.99
1.00
1.09
1.05
2.18
3.28
7.5 7.0 6.5 6.0 5.5 5.0 4.5 4.0 3.5 3.0 2.5 2.0 1.5 1.0 0.5
chemical shift (ppm)

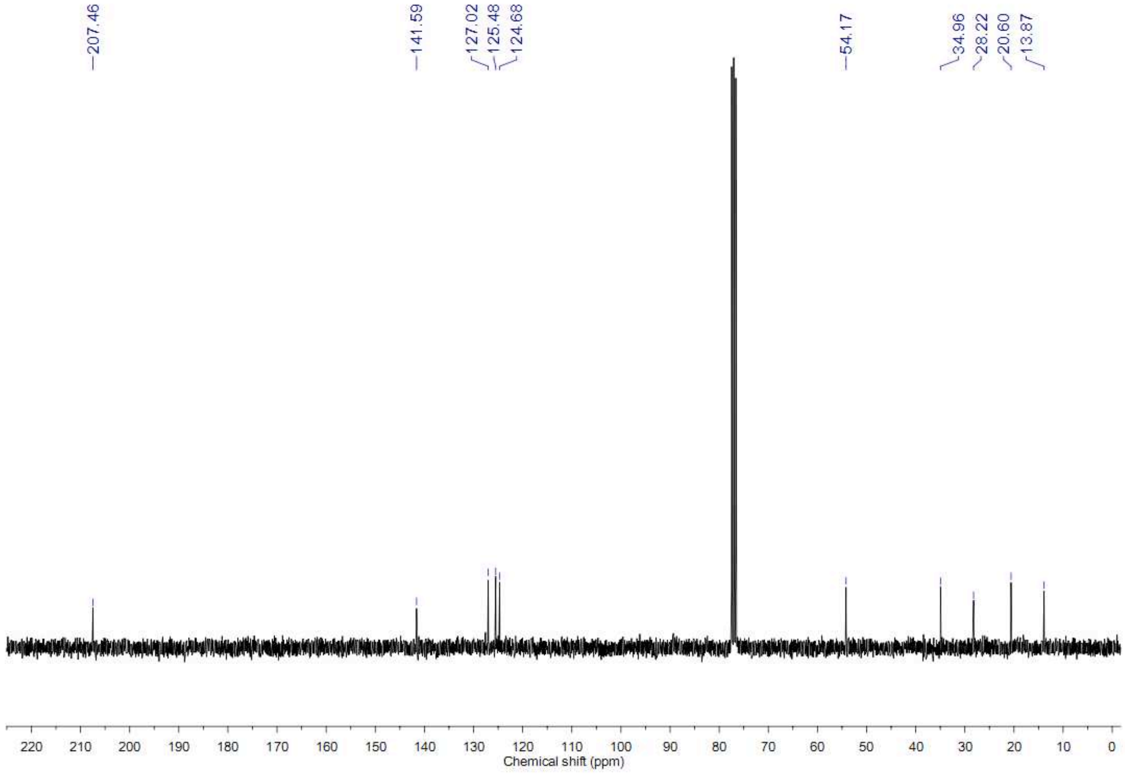
207.46
141.59
127.02
125.48
124.68
54.17
34.96
28.22
20.60
13.87
220 210 200 190 180 170 160 150 140 130 120 110 100 90 80 70 60 50 40 30 20 10 0
Chemical shift (ppm)

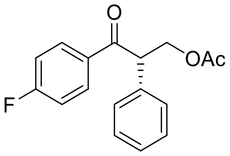

7t

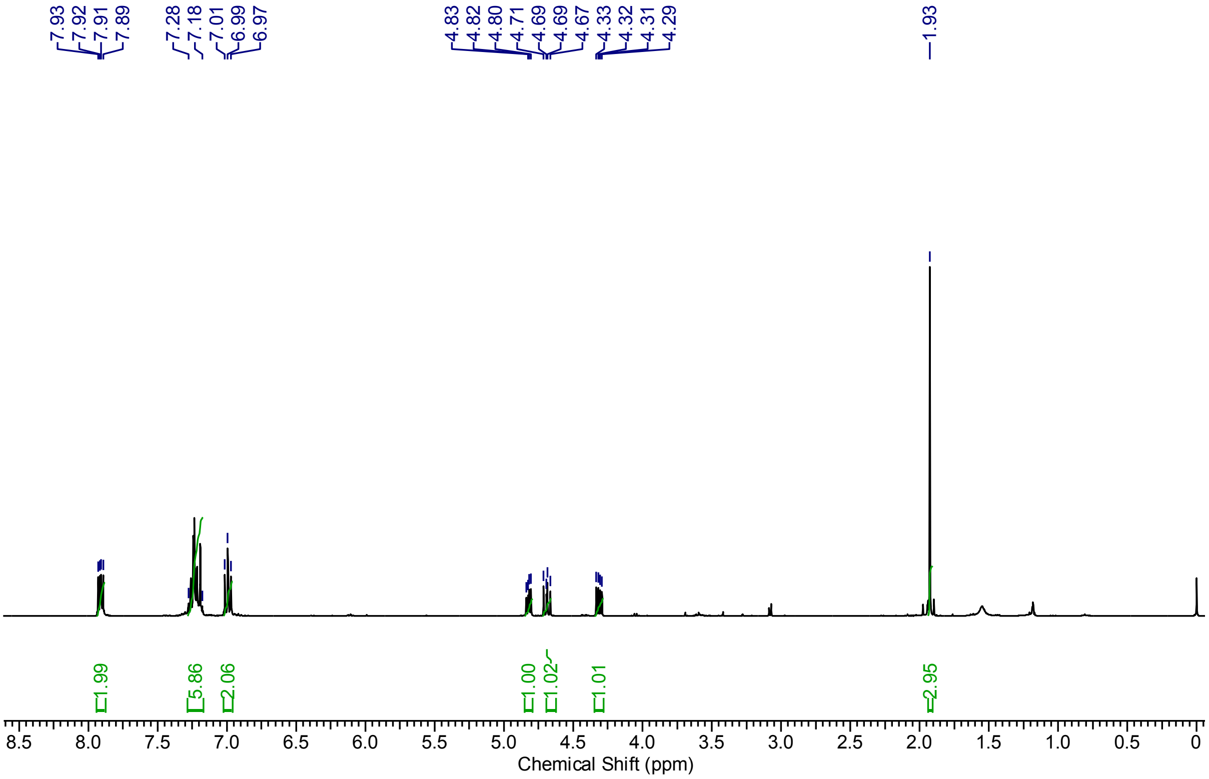
7.93
7.92
7.91
7.89
7.28
7.18
7.01
6.99
6.97
4.83
4.82
4.80
4.71
4.69
4.69
4.67
4.33
4.32
4.31
4.29
1.93
1.99
5.86
2.06
1.00
1.02
1.01
2.95
Chemical Shift (ppm)

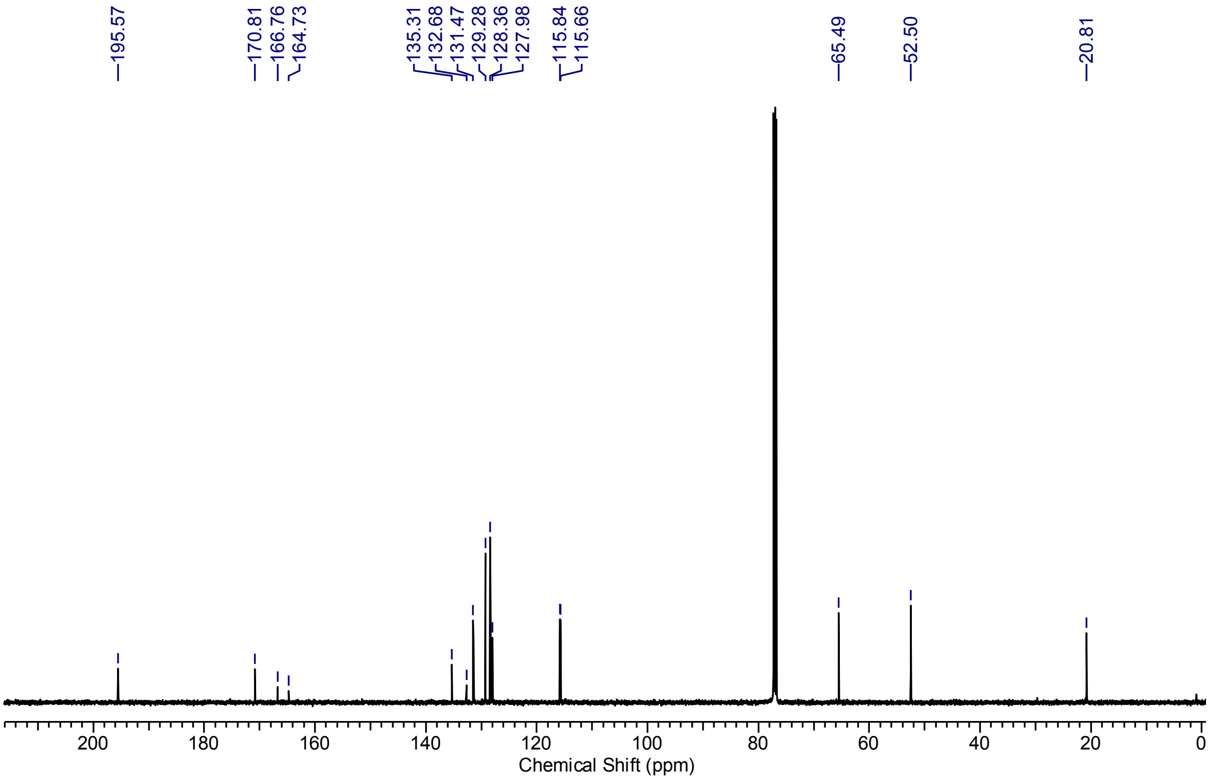
195.57
170.81
166.76
164.73
135.31
132.68
131.47
129.28
128.36
127.98
115.84
115.66
65.49
52.50
20.81
Chemical Shift (ppm)

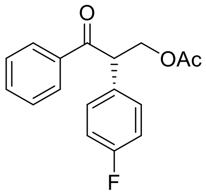
O
OAc
F

7t′

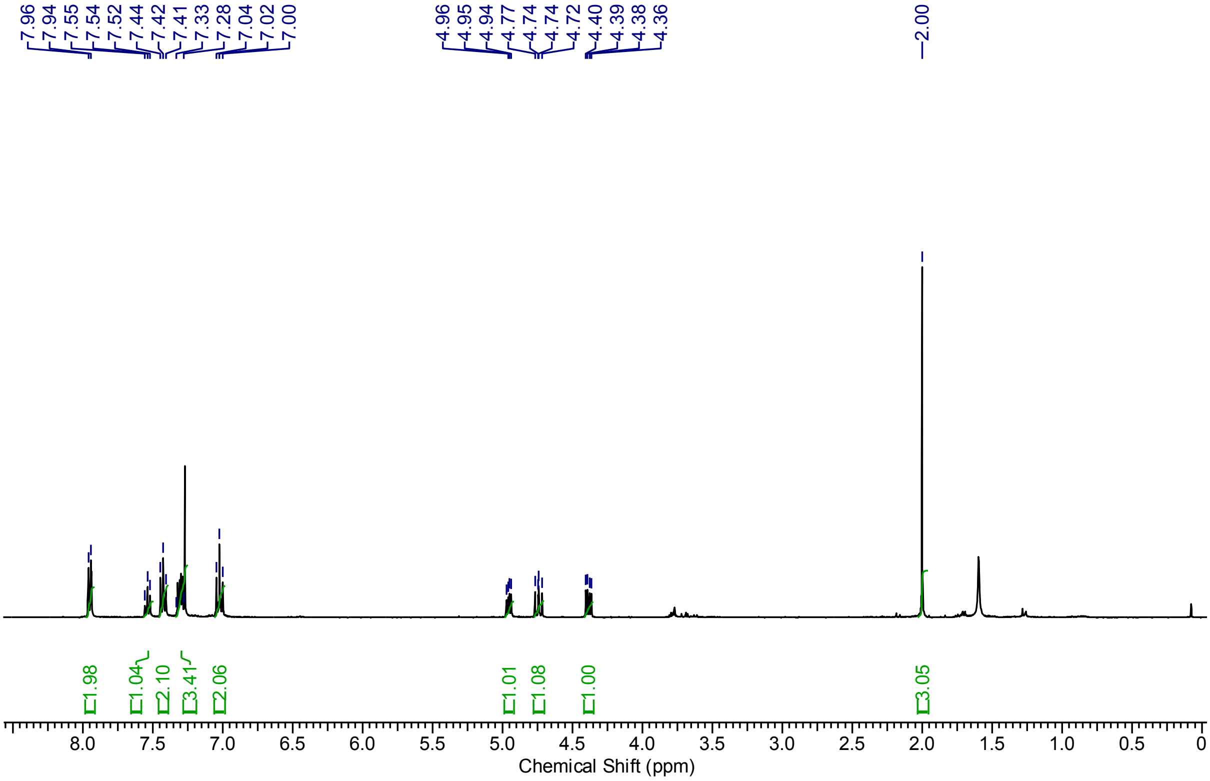
7.96 7.94 7.55 7.54 7.52 7.44 7.42 7.41 7.33 7.28 7.04 7.02 7.00
4.96 4.95 4.94 4.77 4.74 4.74 4.72 4.40 4.39 4.38 4.36
2.00
1.98 1.04 2.10 3.41 2.06 1.01 1.08 1.00 3.05
8.0 7.5 7.0 6.5 6.0 5.5 5.0 4.5 4.0 3.5 3.0 2.5 2.0 1.5 1.0 0.5 0
Chemical Shift (ppm)

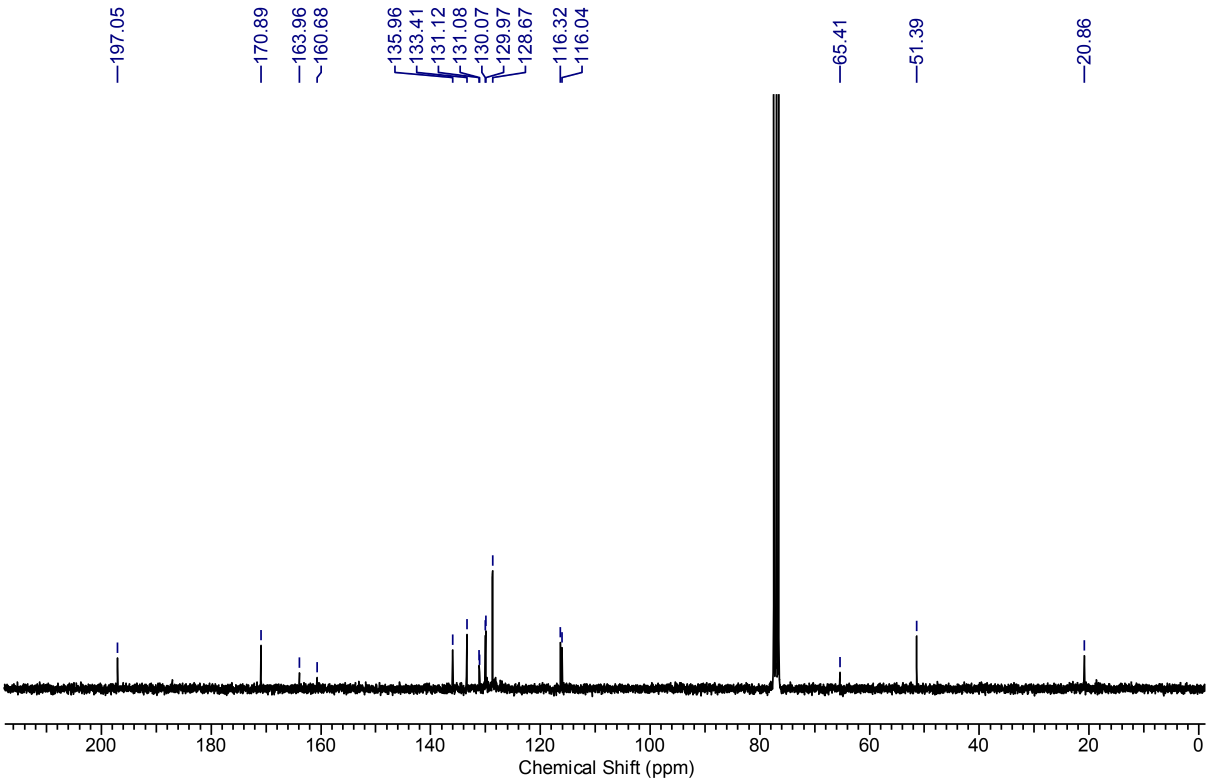
197.05
170.89 163.96 160.68
135.96 133.41 131.12 131.08 130.07 129.97 128.67 116.32 116.04
65.41
51.39
20.86
200 180 160 140 120 100 80 60 40 20 0
Chemical Shift (ppm)

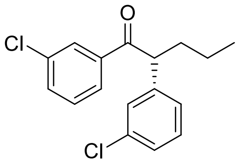
O
Cl
Cl

7u

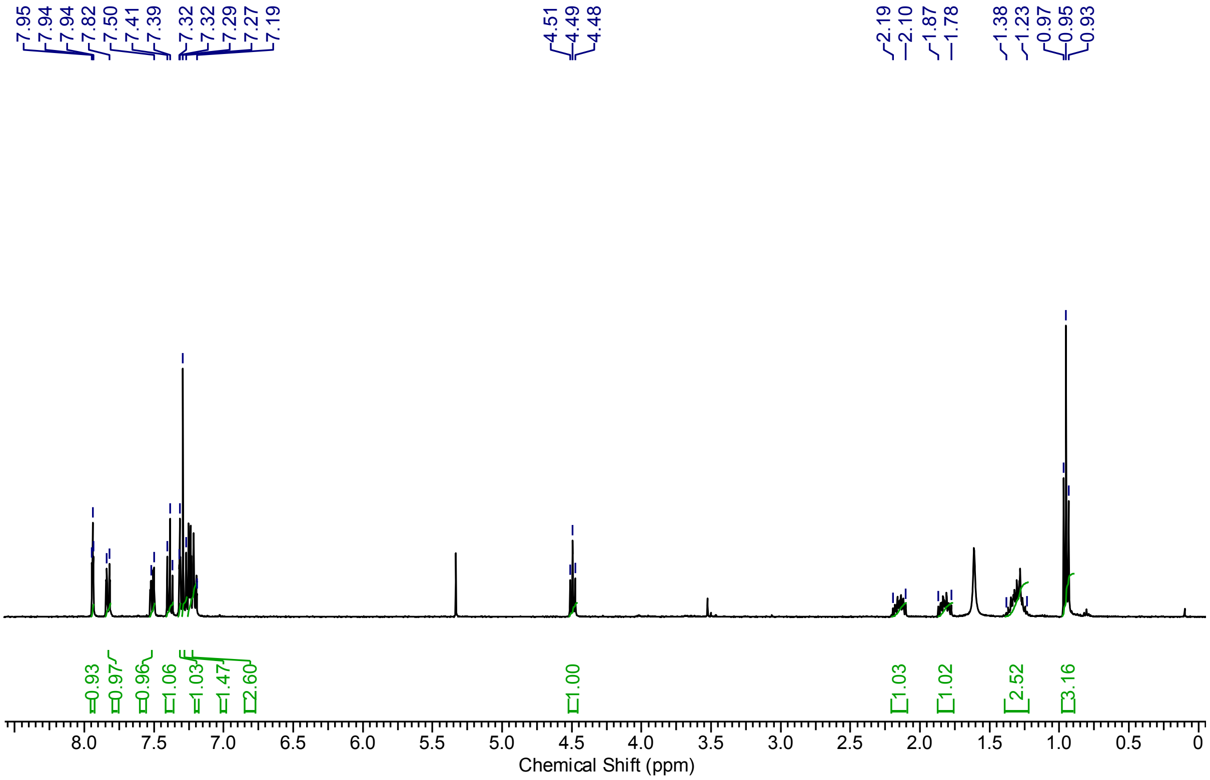
7.95
7.94
7.94
7.82
7.50
7.41
7.39
7.32
7.32
7.29
7.27
7.19
4.51
4.49
4.48
2.19
2.10
1.87
1.78
1.38
1.23
0.97
0.95
0.93
0.93
0.97
0.96
1.06
1.03
1.47
2.60
1.00
1.03
1.02
2.52
3.16
8.0
7.5
7.0
6.5
6.0
5.5
5.0
4.5
4.0
3.5
3.0
2.5
2.0
1.5
1.0
0.5
0
Chemical Shift (ppm)

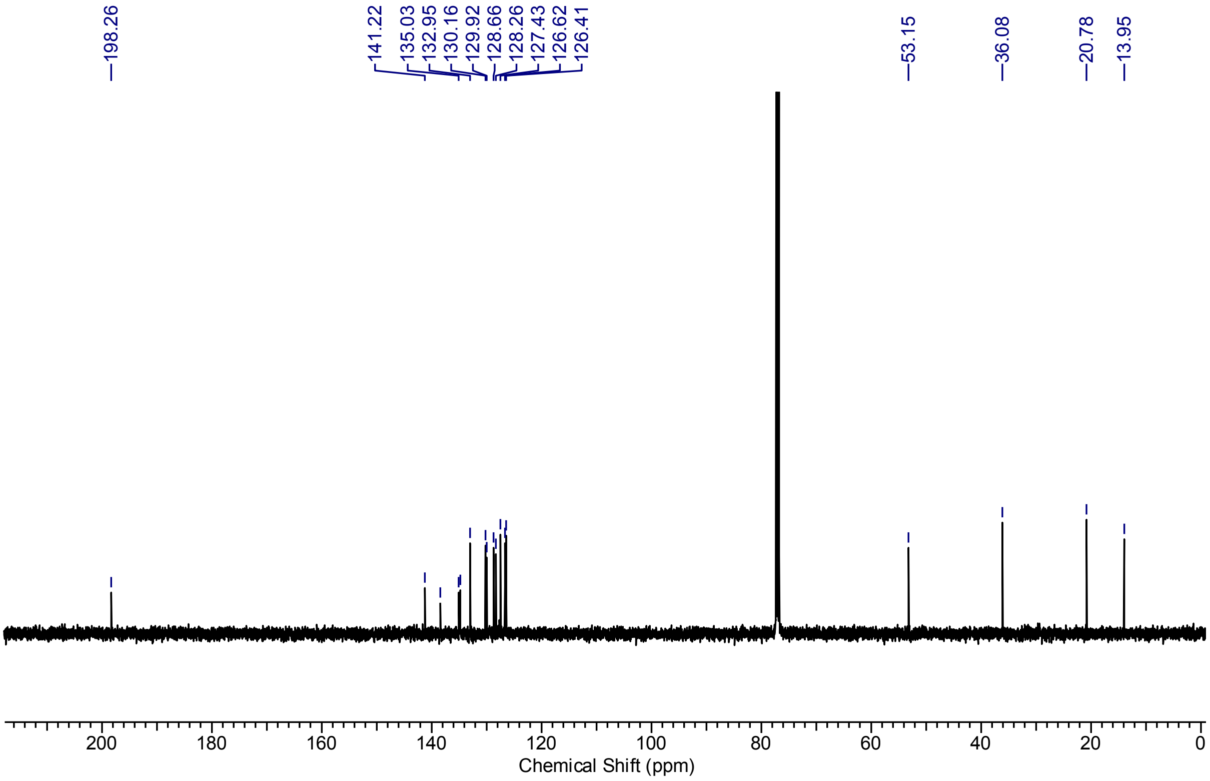
198.26
141.22
135.03
132.95
130.16
129.92
128.66
128.26
127.43
126.62
126.41
53.15
36.08
20.78
13.95
200
180
160
140
120
100
80
60
40
20
0
Chemical Shift (ppm)

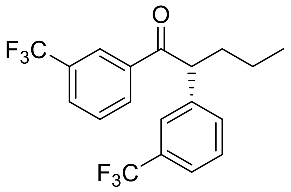
F3C
O
F3C

7v

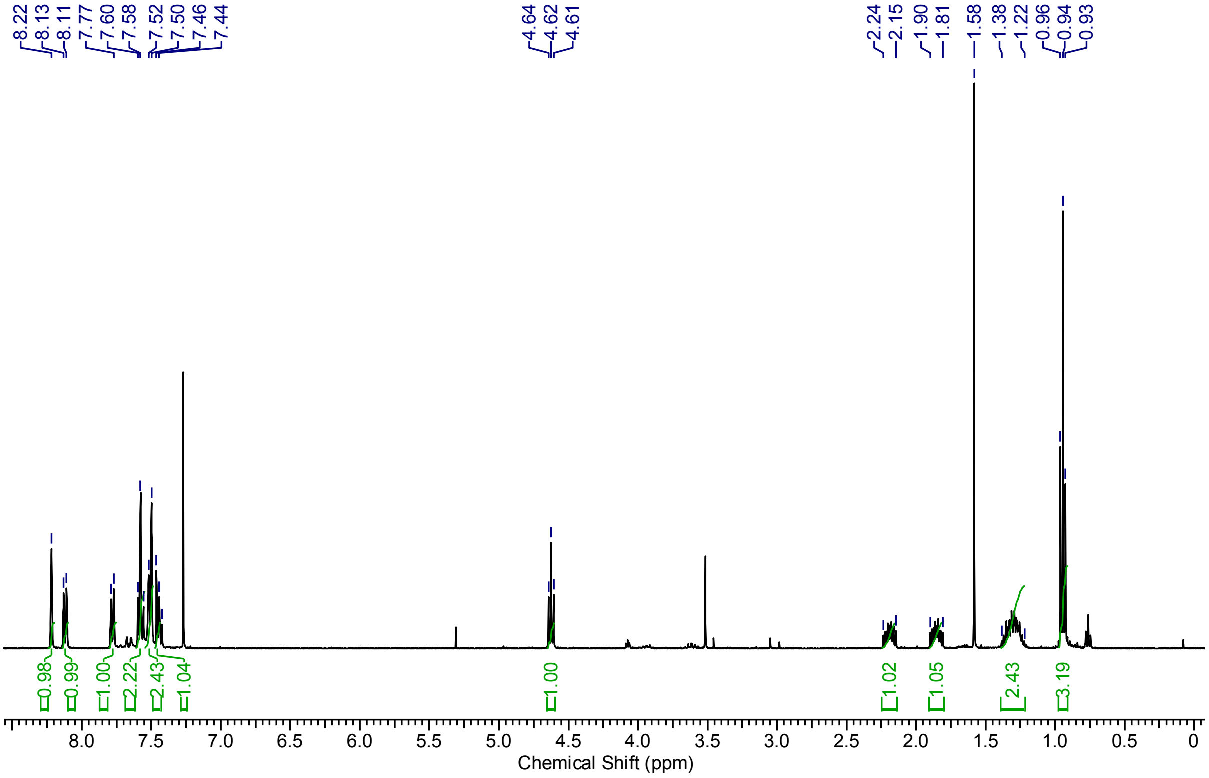
8.22
8.13
8.11
7.77
7.60
7.58
7.52
7.50
7.46
7.44
4.64
4.62
4.61
2.24
2.15
1.90
1.81
1.58
1.38
1.22
0.96
0.94
0.93
0.98
0.99
1.00
2.22
2.43
1.04
1.00
1.02
1.05
2.43
3.19
8.0
7.5
7.0
6.5
6.0
5.5
5.0
4.5
4.0
3.5
3.0
2.5
2.0
1.5
1.0
0.5
0
Chemical Shift (ppm)

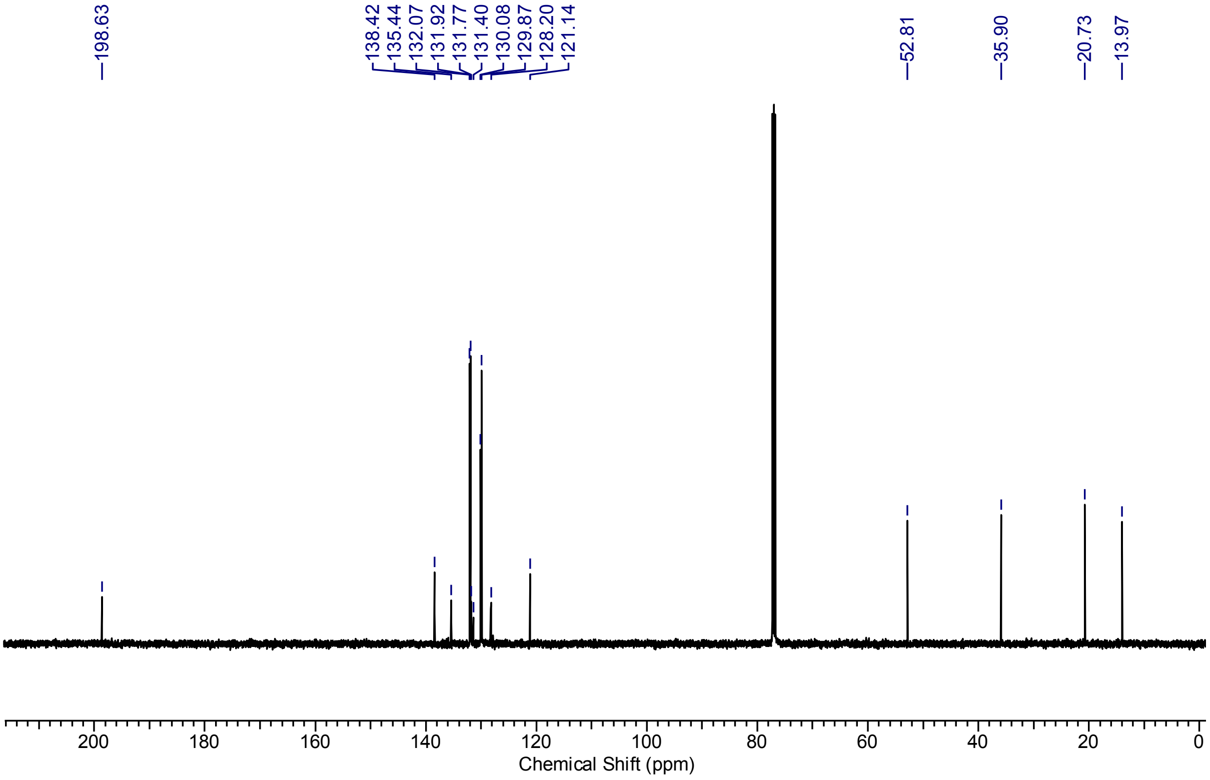
198.63
138.42
135.44
132.07
131.92
131.77
131.40
130.08
129.87
128.20
121.14
52.81
35.90
20.73
13.97
200
180
160
140
120
100
80
60
40
20
0
Chemical Shift (ppm)

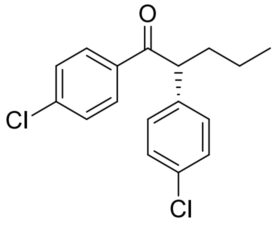
O
Cl
Cl

7w

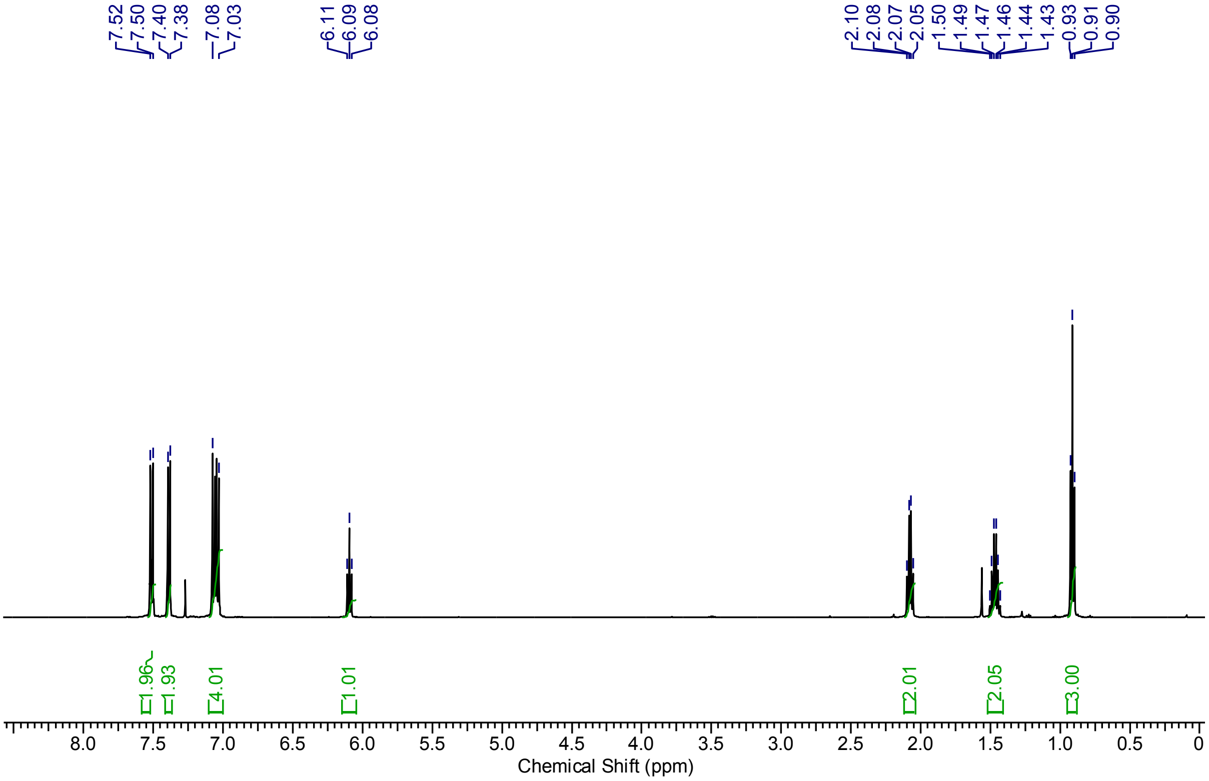
7.52
7.50
7.40
7.38
7.08
7.03
6.11
6.09
6.08
2.10
2.08
2.07
2.05
1.50
1.49
1.47
1.46
1.44
1.43
0.93
0.91
0.90
1.96
1.93
4.01
1.01
2.01
2.05
3.00
8.0
7.5
7.0
6.5
6.0
5.5
5.0
4.5
4.0
3.5
3.0
2.5
2.0
1.5
1.0
0.5
0
Chemical Shift (ppm)

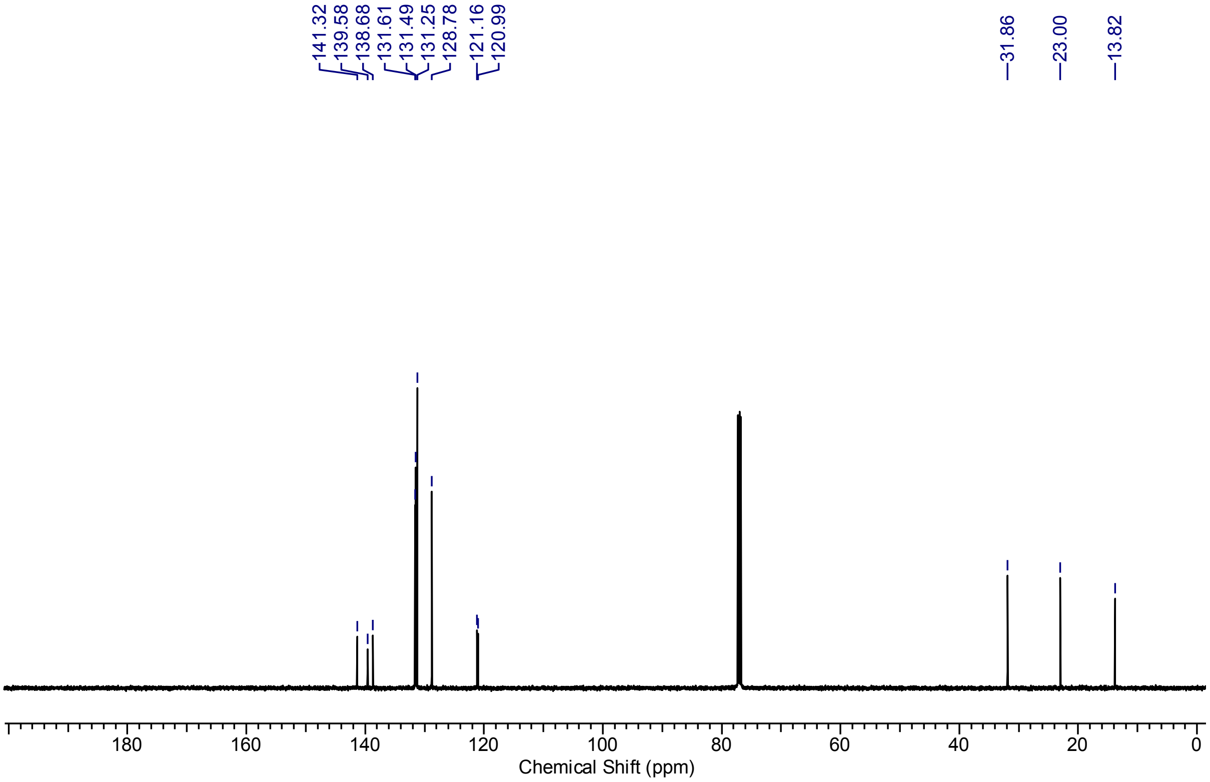
141.32
139.58
138.68
131.61
131.49
131.25
128.78
121.16
120.99
31.86
23.00
13.82
180
160
140
120
100
80
60
40
20
0
Chemical Shift (ppm)

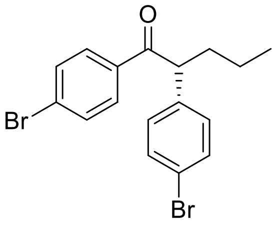
O
Br
Br

7x

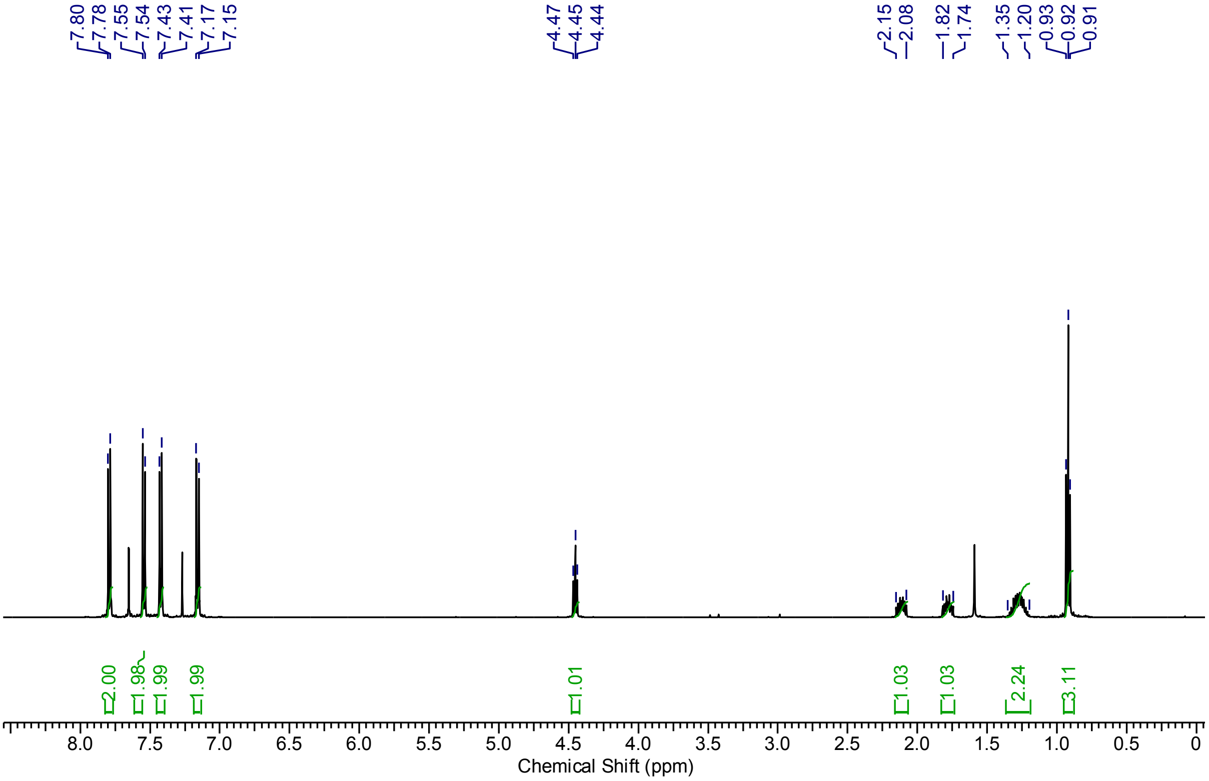
7.80
7.78
7.55
7.54
7.43
7.41
7.17
7.15
4.47
4.45
4.44
2.15
2.08
1.82
1.74
1.35
1.20
0.93
0.92
0.91
2.00
1.98
1.99
1.99
1.01
1.03
1.03
2.24
3.11
8.0 7.5 7.0 6.5 6.0 5.5 5.0 4.5 4.0 3.5 3.0 2.5 2.0 1.5 1.0 0.5 0
Chemical Shift (ppm)

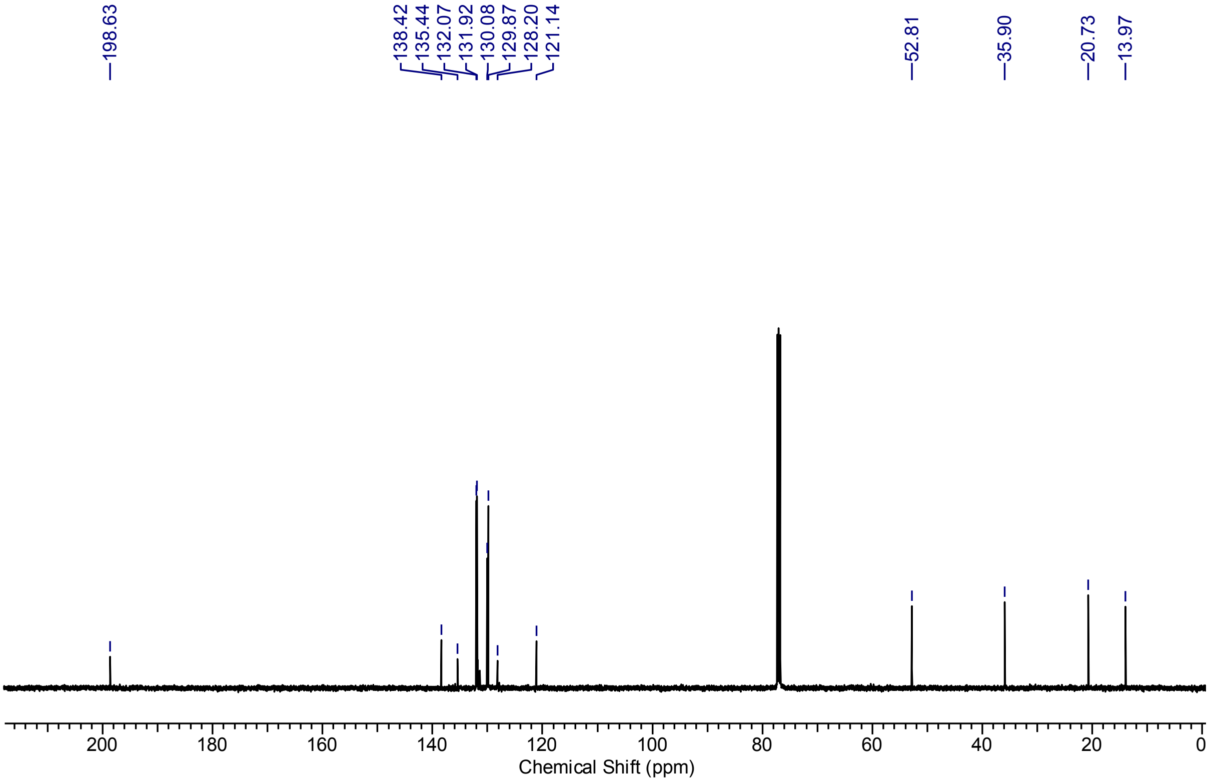
198.63
138.42
135.44
132.07
131.92
130.08
129.87
128.20
121.14
52.81
35.90
20.73
13.97
200 180 160 140 120 100 80 60 40 20 0
Chemical Shift (ppm)

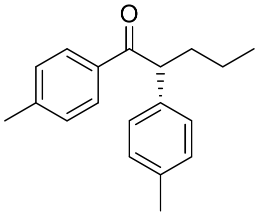
O

7y

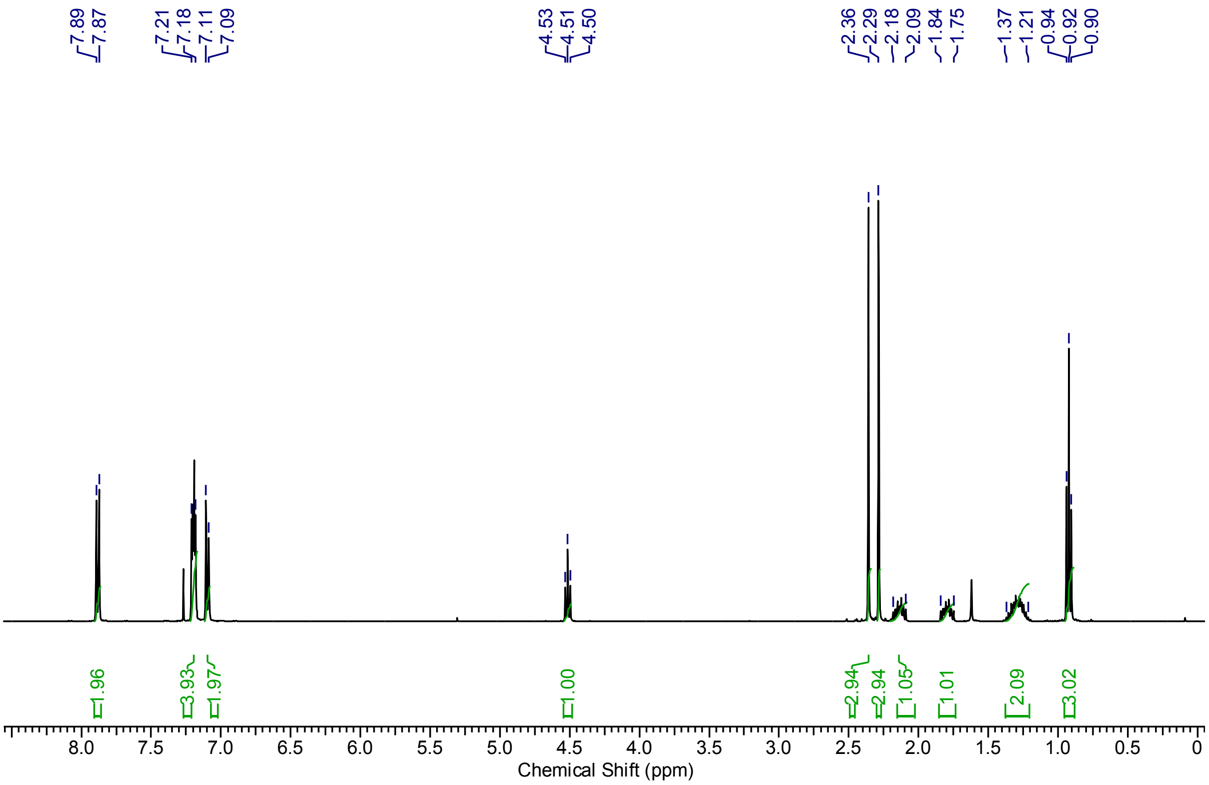
7.89
7.87
7.21
7.18
7.11
7.09
4.53
4.51
4.50
2.36
2.29
2.18
2.09
1.84
1.75
1.37
1.21
0.94
0.92
0.90
1.96
3.93
1.97
1.00
2.94
2.94
1.05
1.01
2.09
3.02
8.0 7.5 7.0 6.5 6.0 5.5 5.0 4.5 4.0 3.5 3.0 2.5 2.0 1.5 1.0 0.5 0
Chemical Shift (ppm)

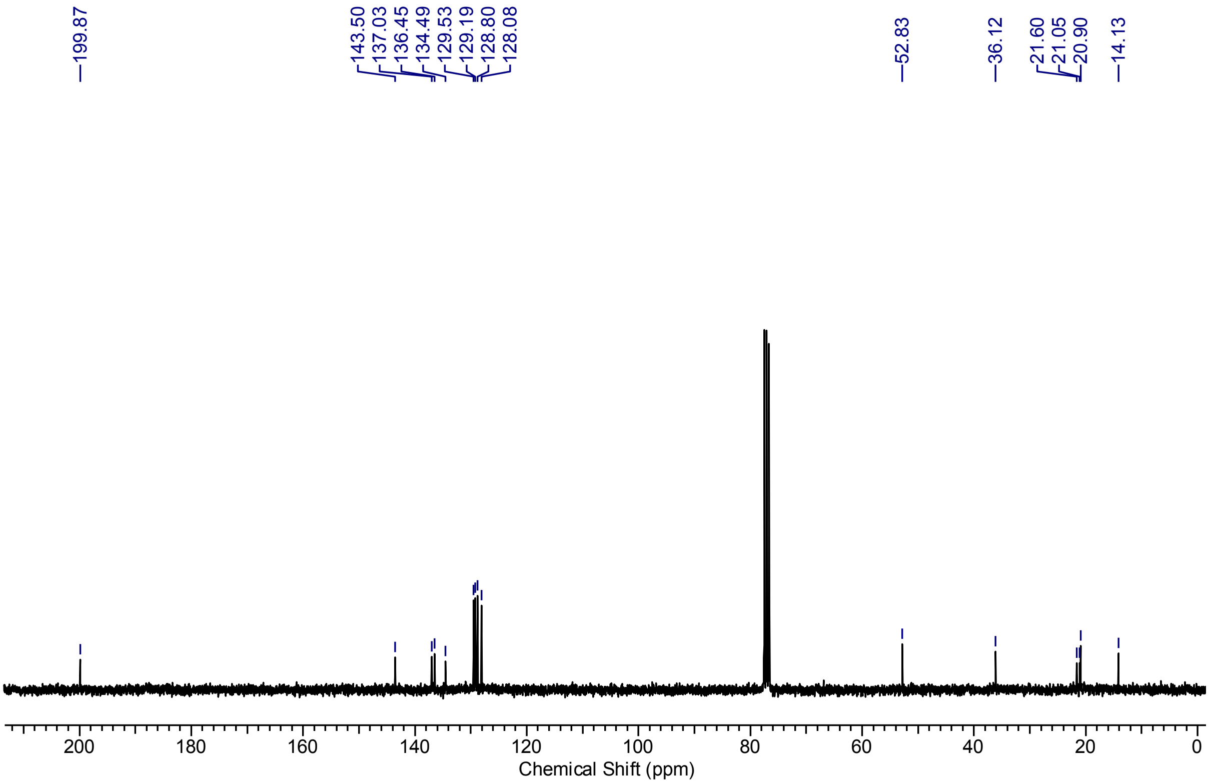
199.87
143.50
137.03
136.45
134.49
129.53
129.19
128.80
128.08
52.83
36.12
21.60
21.05
20.90
14.13
200 180 160 140 120 100 80 60 40 20 0
Chemical Shift (ppm)

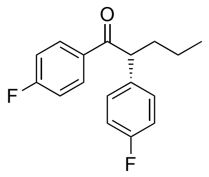
O
F
F

7z

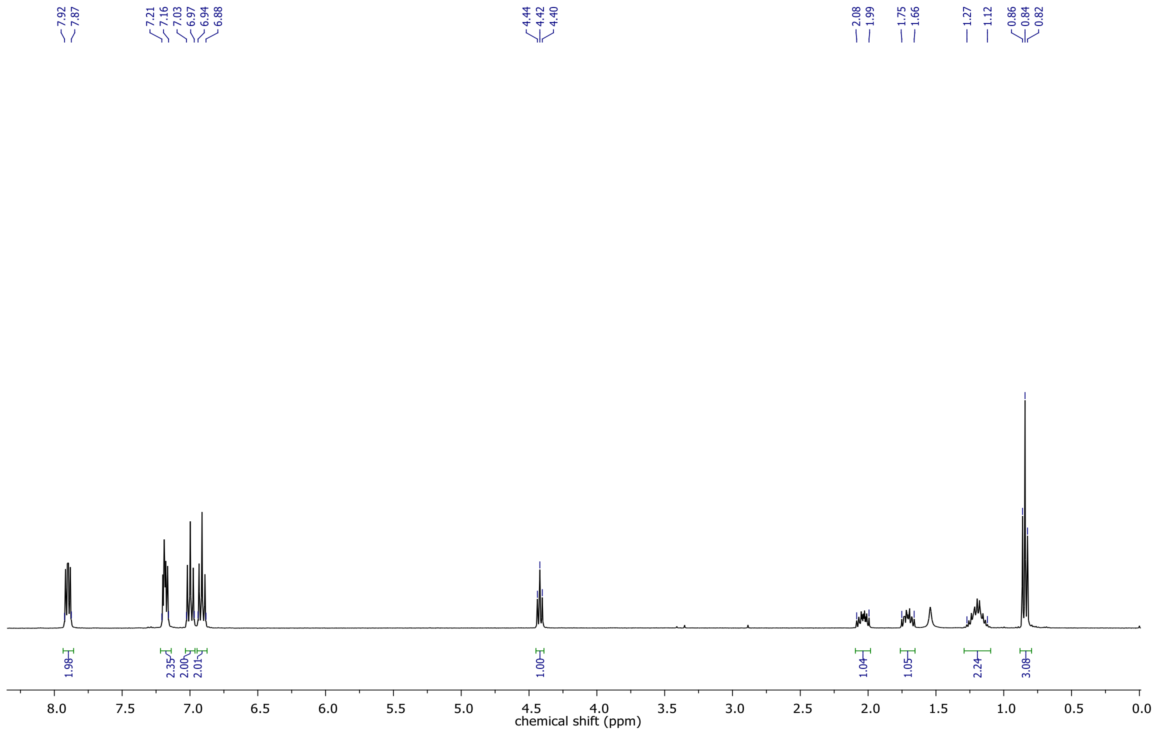
7.92
7.87
7.21
7.16
7.03
6.97
6.94
6.88
4.44
4.42
4.40
2.08
1.99
1.75
1.66
1.27
1.12
0.86
0.84
0.82
1.98
2.35
2.00
2.01
1.00
1.04
1.05
2.24
3.08
8.0 7.5 7.0 6.5 6.0 5.5 5.0 4.5 4.0 3.5 3.0 2.5 2.0 1.5 1.0 0.5 0.0
chemical shift (ppm)

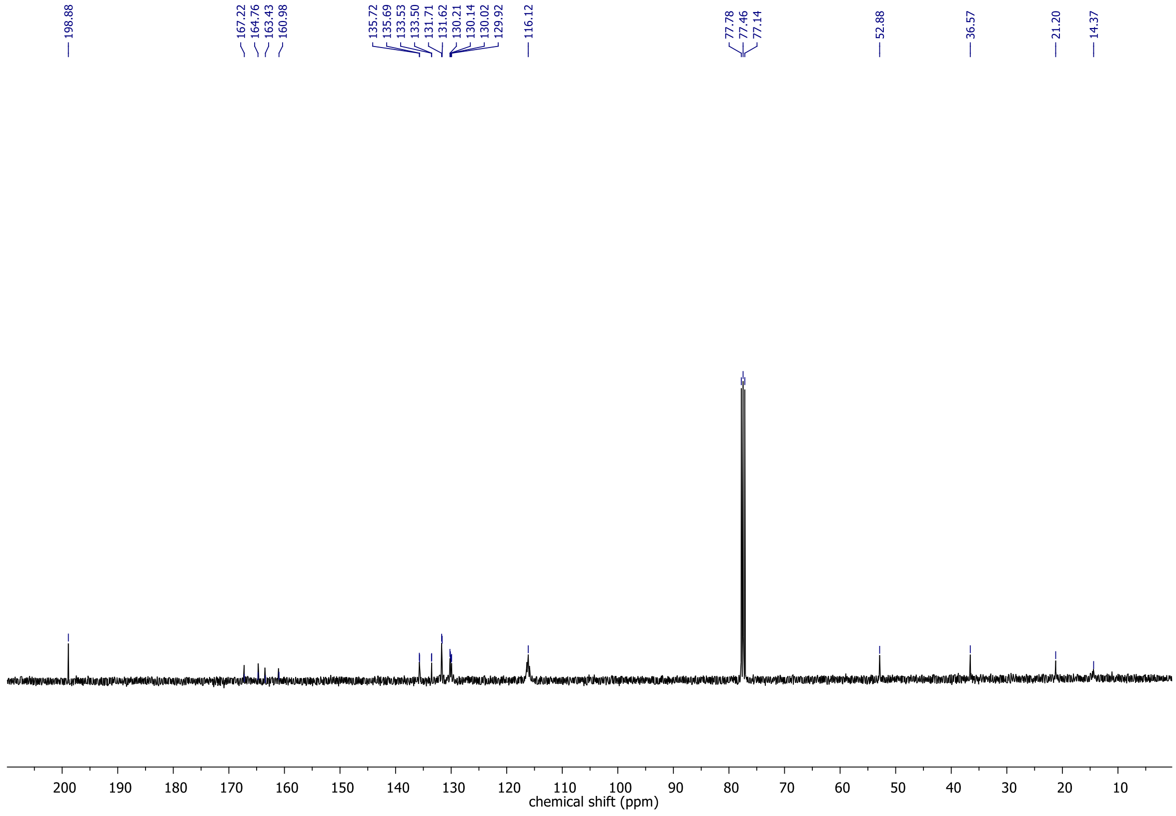
198.88
167.22
164.76
163.43
160.98
135.72
135.69
133.53
133.50
131.71
131.62
130.21
130.14
130.02
129.92
116.12
77.78
77.46
77.14
52.88
36.57
21.20
14.37
200 190 180 170 160 150 140 130 120 110 100 90 80 70 60 50 40 30 20 10
chemical shift (ppm)

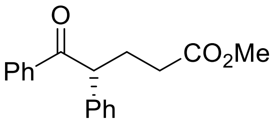
O
Ph
CO_2Me
Ph

11

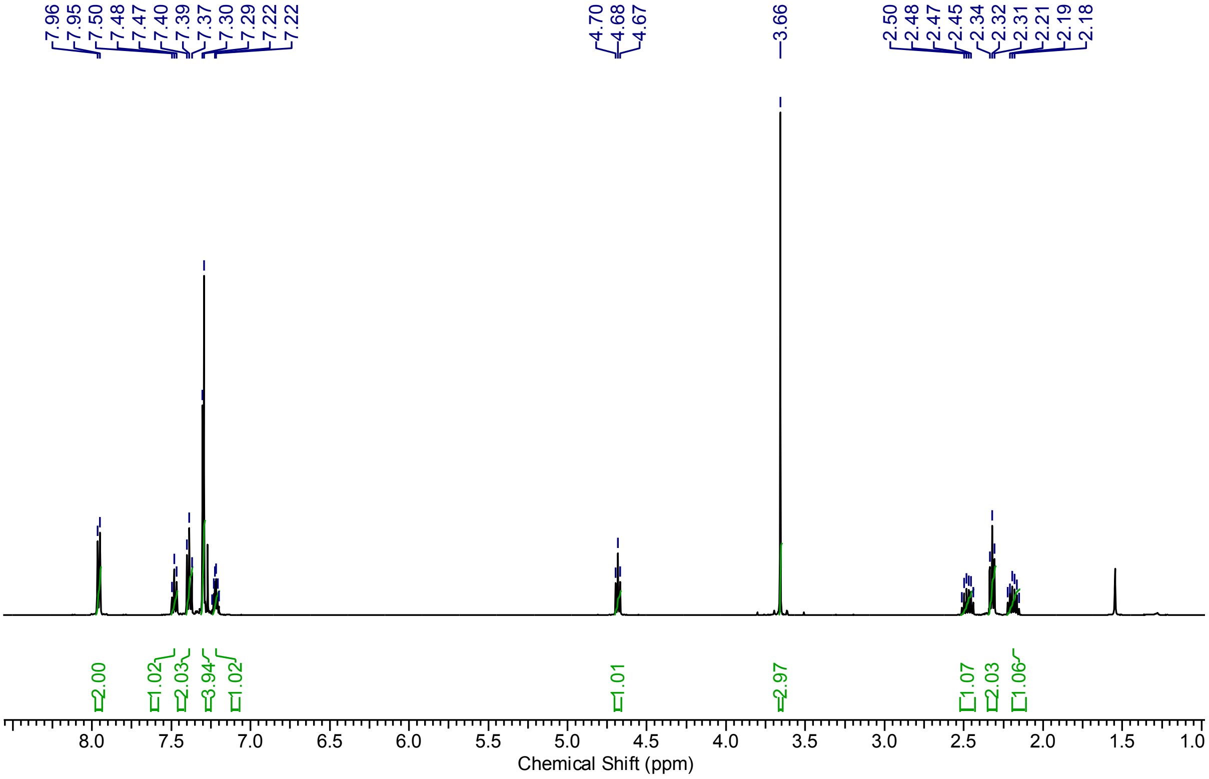
7.96 7.95 7.50 7.48 7.47 7.40 7.39 7.37 7.30 7.29 7.22 7.22
4.70 4.68 4.67
3.66
2.50 2.48 2.47 2.45 2.34 2.32 2.31 2.21 2.19 2.18
2.00 1.02 2.03 3.94 1.02
1.01
2.97
1.07 2.03 1.06
8.0 7.5 7.0 6.5 6.0 5.5 5.0 4.5 4.0 3.5 3.0 2.5 2.0 1.5 1.0
Chemical Shift (ppm)

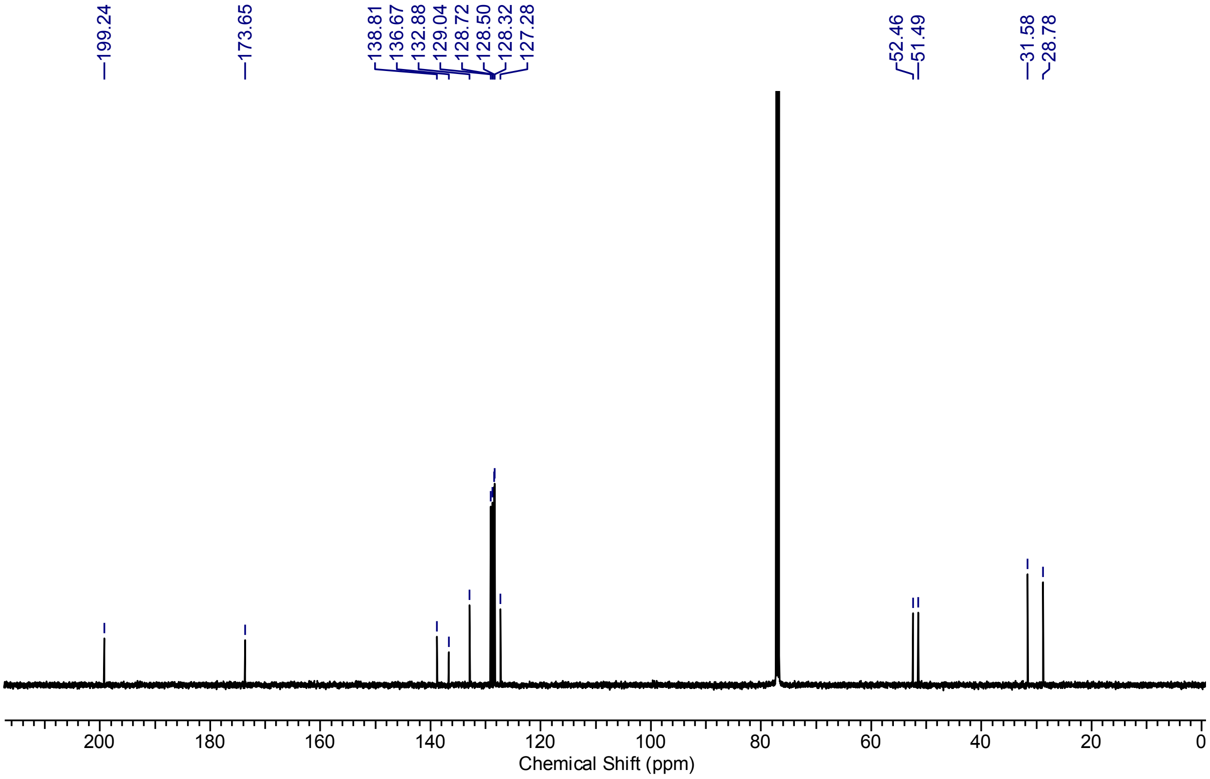
199.24
173.65
138.81 136.67 132.88 129.04 128.72 128.50 128.32 127.28
52.46 51.49
31.58 28.78
200 180 160 140 120 100 80 60 40 20 0
Chemical Shift (ppm)

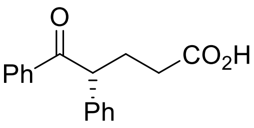
O
Ph
CO2H
Ph

8

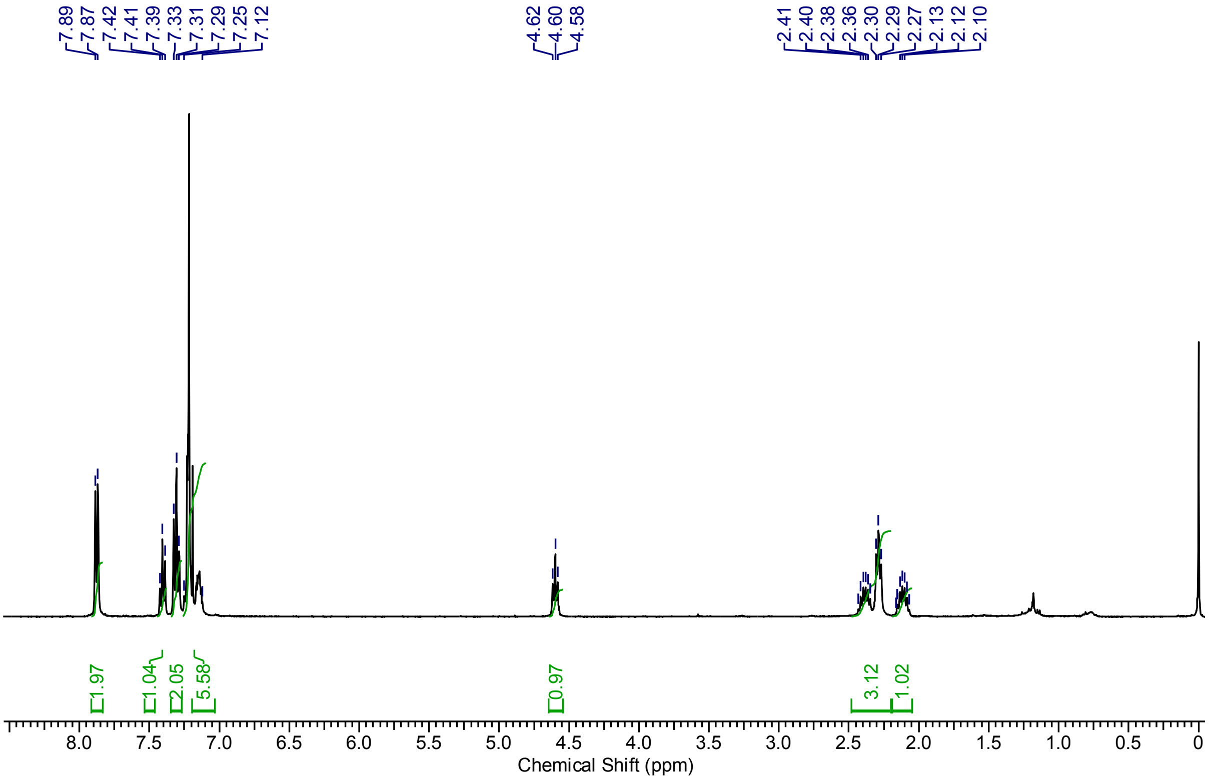
7.89
7.87
7.42
7.41
7.39
7.33
7.31
7.29
7.25
7.12
4.62
4.60
4.58
2.41
2.40
2.38
2.36
2.30
2.29
2.27
2.13
2.12
2.10
1.97
1.04
2.05
5.58
0.97
3.12
1.02
8.0 7.5 7.0 6.5 6.0 5.5 5.0 4.5 4.0 3.5 3.0 2.5 2.0 1.5 1.0 0.5 0
Chemical Shift (ppm)

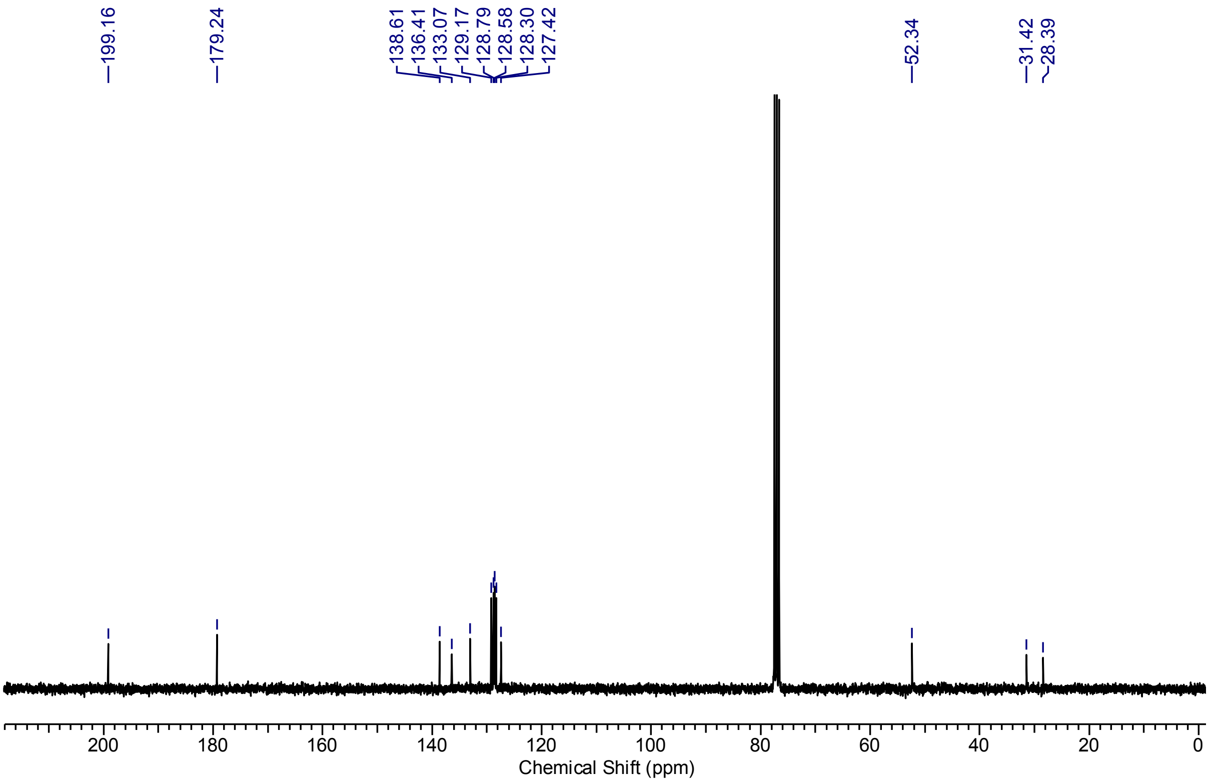
199.16
179.24
138.61
136.41
133.07
129.17
128.79
128.58
128.30
127.42
52.34
31.42
28.39
200 180 160 140 120 100 80 60 40 20 0
Chemical Shift (ppm)

Computational Details

All calculations have been conducted using the Gaussian09 suite of programs.[30] Stationary points were confirmed as ground states by calculated harmonic vibrational frequencies.[31] Approximate free energies were obtained through thermochemical analysis of the frequency calculations, using the thermal correction to the Gibbs free energy as reported in Gaussian09. This takes into account zero-point effects, thermal enthalpy corrections, and entropy. All energies reported in this paper are enthalpies in kcal mol^{-1} at 298 K if not stated otherwise. Frequencies remained unscaled.

M06-2X[32] has been selected to allow for inclusion of dispersion interactions.[33] A composite bases set was constructed using D95V for H atoms, D95V(d) for C and O atoms and LANL2DZ (p,d) for I atoms including LANL2DZ ECPs. To check if the dispersion correction in M06-2X biases iodonium ion structures over carbenium ion structures calculations were repeated with B3LYP[34] and the identical basis set as described above. Population analysis for **A1** was done using the keyword pop=full. NBO calculations were accomplished using the NBO program[35] implemented in Gaussian09. Visualization of geometries, vibrations and molecular orbitals was achieved with Gabedit.[36]

Optimized Geometries, Thermodynamic Data and Population Analysis

To understand the reactivity of **A1** and the observe selectivity bonding situation and charge distribution of **A1** was compared to the parent iodonium ion **A1-parent** (SI-79, 89, 93, 94). Whereas **A1-parent** has major carbenium ion character according to the NBO analysis, in **A1** the positive charge resides for ¾ on the [I] atom and only for ¼ in the former alkene part, with C1 only slightly more positively polarized than C2. The bonding situation reflects the different charge situation in **A1** and **A1-parent**. The C1-I bond length is not influenced by the nature of [I], but the C2-I bond length is, being 23% more elongated in **A1** than in **A1-parent** leading to a more 'symmetric' and at the same time less tight bonding in **A1**. Overall the hybridization of C1 and C2 in **A1** is still to greater 99% sp^2 leaving two p-orbitals that might interact with the π-system of the migrating phenyl ring. A reduced charge separation within the former alkene bond within the iodoniumion framework quite likely lowers the overall reactivity of C1 for a nucleophilic attack. The reduced reactivity may open the temporal window for necessary conformational adjustments as described in the main text.

Optimized Geometries at M06-2X

	**A1-parent**	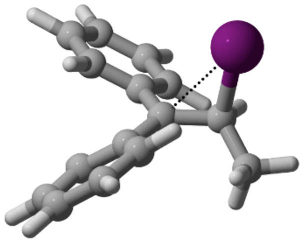

Xyz-Matrix

```
30
XYZ file generated by gabedit : coordinates in Angstrom
C        -0.2842040000         0.2341300000         0.5281080000
C         0.7080210000        -0.6497060000         1.2006330000
C         0.0336260000         1.6001350000         0.1793500000
C         1.7028320000        -0.0369840000         2.1796990000
I         1.6765640000        -1.6336570000        -0.4962460000
C        -0.9894370000         2.5789760000         0.3010080000
C        -0.7131920000         3.9216390000         0.0648270000
C         0.5706220000         4.3079750000        -0.3318860000
C         1.5892880000         3.3510170000        -0.4770990000
C         1.3347990000         2.0160950000        -0.2067570000
H         2.3913940000         0.6720920000         1.7198050000
H         1.1475280000         0.4851540000         2.9687600000
H         2.2883570000        -0.8364730000         2.6413420000
H        -1.4939040000         4.6659320000         0.1928810000
H         0.7835350000         5.3555190000        -0.5309380000
H         2.5777570000         3.6569580000        -0.8077380000
H         2.1182730000         1.2803240000        -0.3680380000
H         0.1963060000        -1.4940200000         1.6594340000
H        -1.9765200000         2.2839290000         0.6470750000
C        -1.6079290000        -0.2805360000         0.2636750000
C        -2.3056790000         0.1657570000        -0.8903680000
C        -3.5574910000        -0.3505170000        -1.1988240000
C        -4.1491580000        -1.2951330000        -0.3506310000
C        -3.4808650000        -1.7377880000         0.8012330000
C        -2.2144420000        -1.2557630000         1.0973830000
H        -1.8247660000         0.8672500000        -1.5679150000
H        -4.0705260000        -0.0293990000        -2.1009030000
H        -5.1328110000        -1.6934680000        -0.5881770000
H        -3.9540630000        -2.4587800000         1.4616770000
H        -1.7291960000        -1.5869960000         2.0116640000
```

Thermodynamic data

```
Zero-point  correction=                                          0.247190
(Hartree/Particle)
 Thermal correction to Energy=                        0.261487
 Thermal correction to Enthalpy=                      0.262431
 Thermal correction to Gibbs Free Energy=             0.204035
 Sum of electronic and zero-point Energies=               -590.655060
 Sum of electronic and thermal Energies=                  -590.640762
 Sum of electronic and thermal Enthalpies=                -590.639818
 Sum of electronic and thermal Free Energies=             -590.698214

                     E (Thermal)             CV                 S
```

	KCal/Mol	Cal/Mol-Kelvin	Cal/Mol-Kelvin
Total	164.086	54.874	122.904

	**A1-π-complex Iodine**	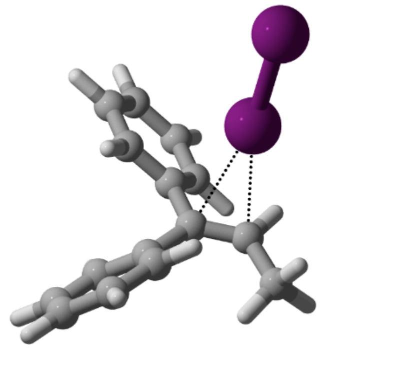

Xyz-Matrix

```
31
XYZ file generated by gabedit : coordinates in Angstrom
C          2.0833130000         0.0737800000         0.8450630000
C          1.5160290000        -0.0920010000         2.0658950000
C          2.6809950000        -1.0489920000         0.0690120000
C          1.4712320000        -1.3518250000         2.8876430000
I         -1.1894150000        -0.0515910000         0.4351300000
C          3.9346150000        -0.8762990000        -0.5416290000
C          4.5247250000        -1.9152640000        -1.2618880000
C          3.8605890000        -3.1383360000        -1.4029310000
C          2.6038070000        -3.3133090000        -0.8168110000
C          2.0199140000        -2.2769830000        -0.0843630000
H          2.1781820000        -2.1016710000         2.5231880000
H          1.7134280000        -1.1173140000         3.9303450000
H          0.4662010000        -1.7953480000         2.8850850000
H          5.5010760000        -1.7697760000        -1.7184090000
H          4.3161420000        -3.9455750000        -1.9719280000
H          2.0733600000        -4.2552570000        -0.9348600000
H          1.0310500000        -2.4135050000         0.3507500000
H          1.0860120000         0.7954580000         2.5344210000
H          4.4481190000         0.0784580000        -0.4437060000
C          2.1560890000         1.4329470000         0.2343510000
C          1.9408760000         1.5967870000        -1.1440780000
C          1.9621370000         2.8666890000        -1.7206650000
C          2.2146240000         3.9937170000        -0.9313580000
C          2.4483800000         3.8391860000         0.4376950000
C          2.4210320000         2.5677850000         1.0156820000
H          1.7359760000         0.7232820000        -1.7611680000
H          1.7780840000         2.9785440000        -2.7866300000
H          2.2372740000         4.9835140000        -1.3815320000
H          2.6639960000         4.7084730000         1.0547790000
H          2.6316150000         2.4494170000         2.0769730000
I         -3.7330500000        -0.2024850000        -0.4779040000
```

Thermodynamic data

```
Zero-point  correction=                                        0.246097
(Hartree/Particle)
 Thermal correction to Energy=                  0.263380
 Thermal correction to Enthalpy=                0.264324
```

```
Thermal correction to Gibbs Free Energy=          0.195023
Sum of electronic and zero-point Energies=           -602.267741
Sum of electronic and thermal Energies=              -602.250458
Sum of electronic and thermal Enthalpies=            -602.249513
Sum of electronic and thermal Free Energies=         -602.318814

                     E (Thermal)             CV                S
                      KCal/Mol        Cal/Mol-Kelvin    Cal/Mol-Kelvin
Total                   165.273             61.493           145.856
```

0.0 kcal/mol	**A1**<br>(synclinal C2-C_{Ph} – C2-H)	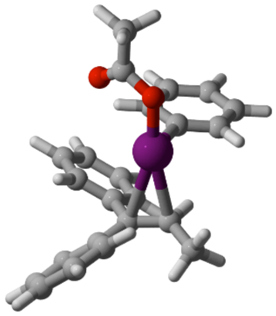

Xyz-Matrix
48
XYZ file generated by gabedit : coordinates in Angstrom

C	1.5916650000	-0.8051650000	-0.7042500000
C	0.9931270000	-0.4967890000	-1.9148840000
C	2.8452140000	-0.1623950000	-0.2495300000
C	1.6211510000	0.2414370000	-3.0740940000
I	-0.4318450000	1.2581010000	-0.4345760000
C	3.7783420000	-0.9169110000	0.4859280000
C	4.9795890000	-0.3450860000	0.9019950000
C	5.2556610000	0.9961130000	0.6180010000
C	4.3309930000	1.7611100000	-0.0998280000
C	3.1421860000	1.1829150000	-0.5412010000
C	-1.9539790000	-0.1579400000	-0.6132910000
C	-2.2867470000	-0.9316690000	0.4998960000
C	-3.3069760000	-1.8757970000	0.3493300000
C	-3.9550490000	-2.0309140000	-0.8788000000
C	-3.5986630000	-1.2387300000	-1.9775060000
C	-2.5859820000	-0.2859760000	-1.8550930000
O	-1.6799660000	2.6751390000	0.2960370000
O	-1.7099240000	1.5529270000	2.2463930000
C	-2.0571820000	2.5097350000	1.5951510000
C	-2.9372300000	3.6399480000	2.0566530000
H	2.7038560000	0.3322680000	-2.9578720000
H	1.4160560000	-0.3167900000	-3.9931160000
H	1.2072410000	1.2479540000	-3.2181050000
H	5.6993640000	-0.9446680000	1.4528200000
H	6.1878700000	1.4433690000	0.9537420000
H	4.5404110000	2.8041650000	-0.3218620000
H	2.4472890000	1.7948840000	-1.1158330000

H	-1.7902090000	-0.7933300000	1.4559020000
H	-3.5882820000	-2.4883110000	1.2018900000
H	-4.7462620000	-2.7689240000	-0.9832120000
H	-4.1107250000	-1.3547540000	-2.9288810000
H	-3.1790060000	3.4946650000	3.1096160000
H	-2.4240480000	4.5934450000	1.9056760000
H	-3.8504390000	3.6517240000	1.4543650000
H	0.0969100000	-1.0709050000	-2.1528560000
H	3.5692470000	-1.9598710000	0.7119440000
C	0.9685080000	-1.8256160000	0.1757220000
C	0.9663620000	-1.6415490000	1.5711080000
C	0.3369070000	-2.5667060000	2.4029680000
C	-0.2839480000	-3.6949390000	1.8537610000
C	-0.2742360000	-3.8924310000	0.4694810000
C	0.3429140000	-2.9605300000	-0.3663760000
H	1.4459460000	-0.7655490000	2.0043670000
H	0.3342850000	-2.4105520000	3.4785870000
H	-0.7587210000	-4.4250020000	2.5049410000
H	-0.7355680000	-4.7785820000	0.0413930000
H	0.3752160000	-3.1398410000	-1.4391820000
H	-2.3092960000	0.3380210000	-2.7021110000

Thermodynamic data

Zero-point correction= 0.390066 (Hartree/Particle)
Thermal correction to Energy= 0.415366
Thermal correction to Enthalpy= 0.416310
Thermal correction to Gibbs Free Energy= 0.332423
Sum of electronic and zero-point Energies= -1050.474719
Sum of electronic and thermal Energies= -1050.449418
Sum of electronic and thermal Enthalpies= -1050.448474
Sum of electronic and thermal Free Energies= -1050.532362

	E (Thermal) KCal/Mol	CV Cal/Mol-Kelvin	S Cal/Mol-Kelvin
Total	260.646	94.494	176.557

+ 0.8 kcal/mol	**A1-π** (π-stacking)	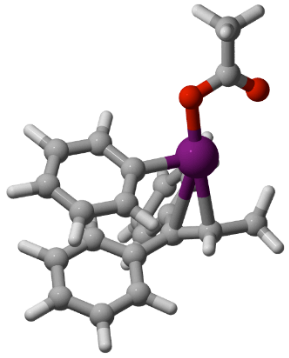

Xyz-Matrix

48

XYZ file generated by gabedit : coordinates in Angstrom

C	-1.4588160000	0.8450790000	-0.8885380000
C	-0.4526670000	1.0017760000	-1.8339480000
C	-1.8500390000	1.9223810000	0.0451620000
C	-2.2038590000	-0.4293030000	-0.8288730000
I	1.6086500000	0.1715220000	-0.4346930000
C	-2.6737330000	-0.9194810000	0.4054070000
C	-3.3559620000	-2.1326290000	0.4699430000
C	-3.5945550000	-2.8680070000	-0.6961190000
C	-3.1455600000	-2.3847900000	-1.9301860000
C	-2.4520560000	-1.1770060000	-1.9961880000
C	-3.2143930000	2.1007820000	0.3483200000
C	-3.6247840000	3.1354260000	1.1874920000
C	-2.6794790000	3.9916830000	1.7602500000
C	-1.3203140000	3.8181860000	1.4769960000
C	-0.9098050000	2.8008350000	0.6181220000
C	0.6530050000	-1.4097150000	0.5164140000
C	0.4084780000	-1.3033290000	1.8881130000
C	-0.1756220000	-2.3932160000	2.5384690000
C	-0.5209730000	-3.5400670000	1.8163290000
C	-0.2860450000	-3.6123200000	0.4393380000
C	0.3156450000	-2.5430420000	-0.2277870000
O	3.3300280000	-0.4856020000	0.4979340000
O	4.1344330000	1.2580100000	-0.6190840000
C	4.3372650000	0.3222500000	0.1344190000
C	5.6601150000	-0.0563610000	0.7418570000
H	-0.3315640000	0.1646930000	-2.5232490000
H	-2.4897060000	-0.3540530000	1.3165470000
H	-3.7075870000	-2.5030570000	1.4297340000
H	-4.1431400000	-3.8055040000	-0.6465050000
H	-3.3565560000	-2.9377180000	-2.8417920000
H	-2.1546060000	-0.7876060000	-2.9674370000
H	-3.9559320000	1.4430900000	-0.0988910000
H	-4.6824830000	3.2735860000	1.3952080000
H	-2.9991500000	4.7923940000	2.4224260000
H	-0.5818080000	4.4799530000	1.9218530000
H	0.1520300000	2.6836970000	0.4125910000
H	0.6776530000	-0.4082150000	2.4439700000
H	-0.3575620000	-2.3425850000	3.6086420000
H	-0.9760200000	-4.3832260000	2.3299740000
H	-0.5601350000	-4.5031160000	-0.1194620000
H	0.5194180000	-2.6041300000	-1.2942070000
H	5.5863900000	-0.0015910000	1.8318540000
H	5.9024070000	-1.0880530000	0.4721250000
H	6.4271630000	0.6265350000	0.3768290000
C	0.0998910000	2.3265870000	-2.3249990000
H	1.1709220000	2.4720430000	-2.1184360000
H	-0.4380640000	3.1718960000	-1.8903880000
H	-0.0102550000	2.3667000000	-3.4134430000

Thermodynamic data

Zero-point correction= 0.389846 (Hartree/Particle)
Thermal correction to Energy= 0.415203
Thermal correction to Enthalpy= 0.416147
Thermal correction to Gibbs Free Energy= 0.331883
Sum of electronic and zero-point Energies= -1050.472899
Sum of electronic and thermal Energies= -1050.447543
Sum of electronic and thermal Enthalpies= -1050.446598
Sum of electronic and thermal Free Energies= -1050.530862

	E (Thermal)	CV	S
	KCal/Mol	Cal/Mol-Kelvin	Cal/Mol-Kelvin
Total	260.544	94.447	177.347

–0.8 kcal/mol	**A1** (synclinal C2-C_{Ph} – C2-CH_3)	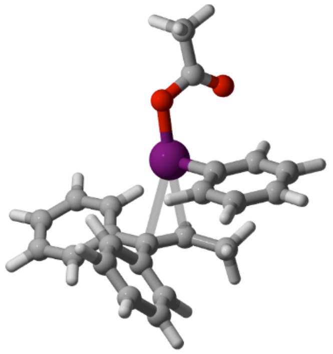

Xyz-Matrix

48

XYZ file generated by gabedit : coordinates in Angstrom

C	-1.7714760000	-0.2910560000	-0.8538210000
C	-0.9056370000	-0.1883280000	-1.9278750000
C	-2.6670600000	0.8446440000	-0.5079430000
C	-1.8598940000	-1.5232600000	-0.0265340000
C	-0.0664450000	-1.2699260000	-2.5560820000
I	0.7071110000	0.9722160000	-0.0095640000
C	-1.8883190000	-1.4221610000	1.3773960000
C	-1.9379380000	-2.5686730000	2.1693410000
C	-1.9835220000	-3.8333310000	1.5692970000
C	-1.9900670000	-3.9416800000	0.1763190000
C	-1.9239080000	-2.7940390000	-0.6176860000
C	-2.3792790000	2.1707360000	-0.8942090000
C	-3.2461460000	3.2130350000	-0.5724880000
C	-4.4279420000	2.9531050000	0.1290150000
C	-4.7362440000	1.6423930000	0.5011610000
C	-3.8628930000	0.5996780000	0.1928550000
H	-5.1053350000	3.7665220000	0.3757140000
C	1.8665680000	-0.7548810000	-0.1263180000
C	2.9158080000	-0.7969860000	-1.0508110000
C	3.6577730000	-1.9767330000	-1.1382690000
C	3.3417300000	-3.0737610000	-0.3283160000
C	2.2880790000	-3.0010180000	0.5882200000

C	1.5330480000	-1.8307720000	0.7021690000
O	1.9829720000	1.8936660000	1.2612350000
O	3.4015640000	2.1852560000	-0.4599980000
C	3.1363630000	2.3609780000	0.7052270000
C	3.9788960000	3.0836280000	1.7214190000
H	0.2356070000	-2.0505900000	-1.8530350000
H	-0.6411090000	-1.7375680000	-3.3665080000
H	0.8289140000	-0.8370100000	-3.0142880000
H	-1.9530820000	-2.4770020000	3.2524650000
H	-2.0332550000	-4.7274180000	2.1858100000
H	-2.0570810000	-4.9190250000	-0.2945360000
H	-1.4839510000	2.4111970000	-1.4670310000
H	-3.0030610000	4.2285850000	-0.8737270000
H	-5.6610720000	1.4289330000	1.0304640000
H	-4.1266900000	-0.4159860000	0.4749030000
H	3.1649820000	0.0660110000	-1.6621320000
H	4.4846990000	-2.0337540000	-1.8409680000
H	3.9247660000	-3.9878530000	-0.4075430000
H	2.0481350000	-3.8511850000	1.2211230000
H	0.7155430000	-1.7722570000	1.4159840000
H	4.8932800000	3.4327730000	1.2415910000
H	3.4140170000	3.9265540000	2.1297900000
H	4.2133340000	2.4057300000	2.5470790000
H	-1.9636010000	-2.8887780000	-1.6999240000
H	-1.0086950000	0.7057080000	-2.5428700000
H	-1.8684620000	-0.4419580000	1.8522190000

Thermodynamic data

Zero-point correction= 0.389925 (Hartree/Particle)
Thermal correction to Energy= 0.415101
Thermal correction to Enthalpy= 0.416045
Thermal correction to Gibbs Free Energy= 0.332519
Sum of electronic and zero-point Energies= -1050.473540
Sum of electronic and thermal Energies= -1050.448364
Sum of electronic and thermal Enthalpies= -1050.447420
Sum of electronic and thermal Free Energies= -1050.530946

	E (Thermal)	CV	S
	KCal/Mol	Cal/Mol-Kelvin	Cal/Mol-Kelvin
Total	260.480	94.496	175.795

Population-Analysis at M06-2X

**A1** (synclinal C2-C_{Ph} – C2-H)	
**HOMO**	**LUMO**

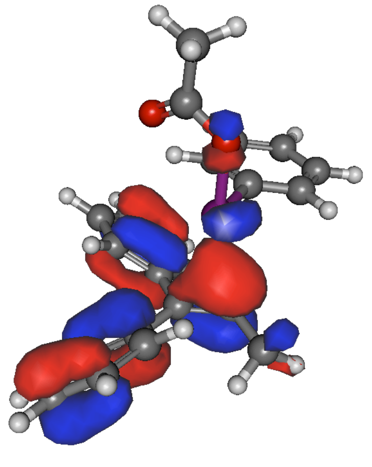	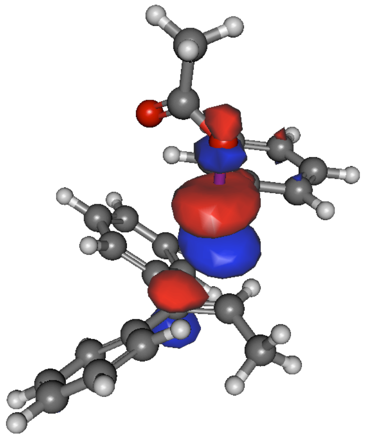

**2a** (synclinal C2-C_{Ph} – C2-CH_3)	
**HOMO**	**LUMO**
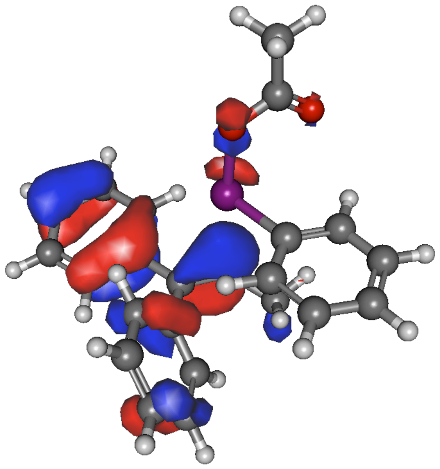	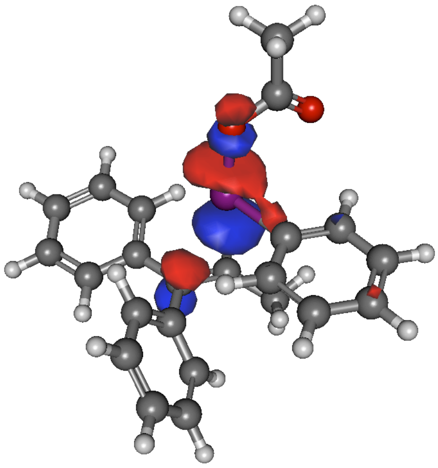

xyz-Matrixes of Final Point of PES SCAN and Local Minimum of PES Scan

	**Local minimum in PES SCAN**	

Xyz-Matrix

```
48
XYZ file generated by gabedit : coordinates in Angstrom
C          1.8745140000         0.4028330000         0.6868930000
C          1.1141620000        -0.0103710000         1.7723750000
C          3.0304470000        -0.3582610000         0.1790040000
C          1.5308730000        -1.0227640000         2.8200320000
I         -0.9389160000        -1.3110680000         0.4555530000
C          4.1442070000         0.3349170000        -0.3317150000
C          5.2575040000        -0.3637280000        -0.7953730000
C          5.2619070000        -1.7625450000        -0.7855900000
```

C	4.1532630000	-2.4626950000	-0.2984860000
C	3.0509190000	-1.7655820000	0.1916580000
C	-1.9287110000	0.5218370000	0.4826660000
C	-1.8890540000	1.3107480000	-0.6673030000
C	-2.5318790000	2.5492390000	-0.6166290000
C	-3.1799930000	2.9615770000	0.5526570000
C	-3.2141240000	2.1380030000	1.6861400000
C	-2.5856370000	0.8916870000	1.6661640000
O	-2.5308700000	-2.3627400000	-0.1982050000
O	-2.3431230000	-1.2899710000	-2.1675040000
C	-2.9020690000	-2.1178200000	-1.4904930000
C	-4.0579570000	-2.9922070000	-1.8951160000
H	2.5935400000	-1.2643510000	2.7349620000
H	1.3519700000	-0.6036770000	3.8151820000
H	0.9735220000	-1.9676630000	2.7715250000
H	6.1205530000	0.1813130000	-1.1689690000
H	6.1267790000	-2.3065450000	-1.1573750000
H	4.1513690000	-3.5495760000	-0.3007260000
H	2.1895090000	-2.3209550000	0.5615900000
H	-1.4072900000	0.9552360000	-1.5729770000
H	-2.5293060000	3.1841190000	-1.4984050000
H	-3.6788950000	3.9273360000	0.5785750000
H	-3.7395870000	2.4563180000	2.5830600000
H	-4.3009690000	-2.7946150000	-2.9390110000
H	-3.7902490000	-4.0431530000	-1.7539180000
H	-4.9173960000	-2.7757850000	-1.2536760000
H	0.3407390000	0.7052290000	2.0498540000
H	4.1425890000	1.4229300000	-0.3414270000
C	1.4920810000	1.6465010000	-0.0196270000
C	1.5622100000	1.7125910000	-1.4239020000
C	1.1675870000	2.8698320000	-2.0946620000
C	0.7260130000	3.9842980000	-1.3715170000
C	0.6748800000	3.9354950000	0.0255260000
C	1.0457010000	2.7716030000	0.6974360000
H	1.9134940000	0.8493230000	-1.9868150000
H	1.2168140000	2.9078870000	-3.1800370000
H	0.4463660000	4.8962990000	-1.8938370000
H	0.3559770000	4.8075230000	0.5907760000
H	1.0395620000	2.7542130000	1.7855790000
H	-2.6256700000	0.2357670000	2.5366220000

	**end point in PES SCAN**	

Xyz-Matrix

48

XYZ file generated by gabedit : coordinates in Angstrom

C	2.0817270000	-0.1443280000	0.6703500000
C	1.1940930000	-0.6345440000	1.5957990000
C	3.1220040000	-0.9992420000	0.0526980000
C	1.2963620000	-1.9737330000	2.2959850000
I	-1.6871210000	-1.1518930000	0.5706780000

C	4.4356530000	-0.5150740000	-0.0735060000
C	5.4340460000	-1.3193720000	-0.6229450000
C	5.1275230000	-2.6059450000	-1.0781860000
C	3.8185160000	-3.0881190000	-0.9766300000
C	2.8244220000	-2.2920870000	-0.4073750000
C	-1.6804140000	0.8860480000	0.2130880000
C	-1.1816710000	1.3418120000	-1.0123800000
C	-1.1802000000	2.7190770000	-1.2346040000
C	-1.6642570000	3.5930040000	-0.2553180000
C	-2.1517550000	3.1077030000	0.9639250000
C	-2.1583580000	1.7357580000	1.2187220000
O	-3.5827400000	-1.4971000000	0.1011230000
O	-3.0514080000	-1.0525420000	-2.0454380000
C	-3.8840570000	-1.3791590000	-1.2438360000
C	-5.3315430000	-1.7030670000	-1.4855370000
H	2.3154450000	-2.3677270000	2.2402180000
H	1.0261190000	-1.8603030000	3.3510640000
H	0.6400810000	-2.7518780000	1.8786780000
H	6.4503110000	-0.9417710000	-0.6998220000
H	5.9041240000	-3.2288680000	-1.5149750000
H	3.5741820000	-4.0823040000	-1.3420400000
H	1.8032270000	-2.6654180000	-0.3432610000
H	-0.8276060000	0.6510280000	-1.7730440000
H	-0.8007340000	3.1050860000	-2.1765490000
H	-1.6609070000	4.6636470000	-0.4438220000
H	-2.5274910000	3.7936260000	1.7181400000
H	-5.5525990000	-1.5450260000	-2.5411660000
H	-5.5209880000	-2.7438590000	-1.2069450000
H	-5.9608470000	-1.0656250000	-0.8586300000
H	0.5262420000	0.1027730000	2.0508720000
H	4.6759560000	0.4868370000	0.2771300000
C	2.0128860000	1.2780280000	0.2541730000
C	2.3445890000	1.6408760000	-1.0658480000
C	2.2846460000	2.9734900000	-1.4741450000
C	1.9029970000	3.9687890000	-0.5683380000
C	1.5776230000	3.6207750000	0.7473840000
C	1.6316120000	2.2883580000	1.1558700000
H	2.6525670000	0.8719440000	-1.7716880000
H	2.5495510000	3.2371510000	-2.4952820000
H	1.8805330000	5.0109400000	-0.8789370000
H	1.3066340000	4.3934870000	1.4632380000
H	1.4325090000	2.0409000000	2.1966700000
H	-2.5343810000	1.3485510000	2.1626550000

Optimized Geometries at B3LYP

	**A1-parent**	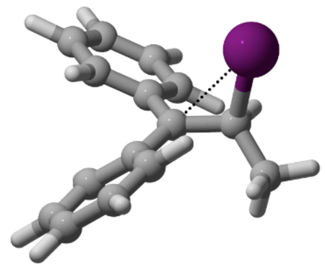

Xyz-Matrix

```
30
XYZ file generated by gabedit : coordinates in Angstrom
C        -0.3733190000        0.1255730000        0.5157870000
C         0.8529070000       -0.4150980000        1.1884930000
C        -0.5049230000        1.5291360000        0.1831990000
C         1.5768540000        0.4390450000        2.2288560000
I         2.1796990000       -1.0053570000       -0.4946490000
C        -1.7893480000        2.1455290000        0.2953220000
C        -1.9489030000        3.5099640000        0.0597790000
C        -0.8464760000        4.2885590000       -0.3244600000
C         0.4263970000        3.6981440000       -0.4562730000
C         0.6029860000        2.3462590000       -0.1890270000
H         1.9658430000        1.3795900000        1.8356040000
H         0.8764860000        0.6733170000        3.0431950000
H         2.4102230000       -0.1311840000        2.6507890000
H        -2.9274790000        3.9694040000        0.1821610000
H        -0.9736050000        5.3520240000       -0.5207760000
H         1.2738010000        4.3010290000       -0.7760080000
H         1.5805590000        1.8987460000       -0.3444520000
H         0.6392000000       -1.3992490000        1.6009750000
H        -2.6385670000        1.5613650000        0.6392660000
C        -1.4684450000       -0.7835760000        0.2464640000
C        -2.2883780000       -0.5774330000       -0.9039520000
C        -3.3207810000       -1.4601870000       -1.2091330000
C        -3.5828230000       -2.5518300000       -0.3641020000
C        -2.7947360000       -2.7682860000        0.7824000000
C        -1.7381640000       -1.9126400000        1.0753320000
H        -2.0580540000        0.2371250000       -1.5858590000
H        -3.9151710000       -1.3106430000       -2.1079600000
H        -4.3949510000       -3.2379880000       -0.5992200000
H        -3.0123160000       -3.6058080000        1.4417250000
H        -1.1670930000       -2.0827530000        1.9848570000
```

Thermodynamic Data

```
Zero-point  correction=                                     0.244944
(Hartree/Particle)
 Thermal correction to Energy=                    0.259344
 Thermal correction to Enthalpy=                  0.260288
 Thermal correction to Gibbs Free Energy=         0.201651
 Sum of electronic and zero-point Energies=           -590.952864
 Sum of electronic and thermal Energies=              -590.938465
 Sum of electronic and thermal Enthalpies=            -590.937520
```

Sum of electronic and thermal Free Energies= -590.996157

	E (Thermal) KCal/Mol	CV Cal/Mol-Kelvin	S Cal/Mol-Kelvin
Total	162.741	55.396	123.411

	**A1-π-complex iodine**	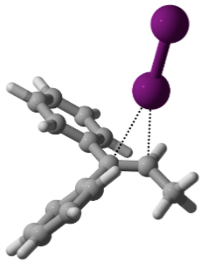

Xyz-Matrix

31
XYZ file generated by gabedit : coordinates in Angstrom

C	2.1101890000	0.0565750000	0.7874100000
C	1.4222740000	-0.1271520000	1.9572920000
C	2.7790860000	-1.0661870000	0.0644830000
C	1.3444770000	-1.3742930000	2.8015240000
I	-1.2876280000	-0.0823020000	0.4074760000
C	4.1255210000	-0.9360740000	-0.3367950000
C	4.7842870000	-1.9839580000	-0.9903290000
C	4.1036370000	-3.1769270000	-1.2764820000
C	2.7594500000	-3.3107480000	-0.9009790000
C	2.1050530000	-2.2672430000	-0.2343580000
H	1.9534180000	-2.1897180000	2.4029320000
H	1.6979980000	-1.1421840000	3.8166920000
H	0.3104720000	-1.7303790000	2.9025410000
H	5.8279030000	-1.8678110000	-1.2801530000
H	4.6127340000	-3.9889570000	-1.7942290000
H	2.2154270000	-4.2253080000	-1.1334720000
H	1.0543330000	-2.3737830000	0.0289790000
H	0.9738330000	0.7594580000	2.4079740000
H	4.6604300000	-0.0117700000	-0.1251050000
C	2.2692340000	1.4290050000	0.2219340000
C	2.2671210000	1.6327180000	-1.1740580000
C	2.3851340000	2.9185320000	-1.7126200000
C	2.5234090000	4.0295990000	-0.8674520000
C	2.5459010000	3.8398220000	0.5217110000
C	2.4221650000	2.5535540000	1.0600030000
H	2.1527940000	0.7796180000	-1.8402830000
H	2.3651030000	3.0541880000	-2.7931870000
H	2.6200770000	5.0303650000	-1.2865460000
H	2.6708530000	4.6934670000	1.1867920000
H	2.4733850000	2.4182950000	2.1391600000
I	-3.9061530000	-0.1537130000	-0.4859630000

Thermodynamic Data

Zero-point correction= 0.243428 (Hartree/Particle)
Thermal correction to Energy= 0.260979

```
Thermal correction to Enthalpy=              0.261923
Thermal correction to Gibbs Free Energy=     0.191690
Sum of electronic and zero-point Energies=         -602.607563
Sum of electronic and thermal Energies=            -602.590011
Sum of electronic and thermal Enthalpies=          -602.589067
Sum of electronic and thermal Free Energies=       -602.659301

                    E (Thermal)         CV              S
                     KCal/Mol     Cal/Mol-Kelvin  Cal/Mol-Kelvin
Total                 163.767          62.129         147.819
```

	**A1**	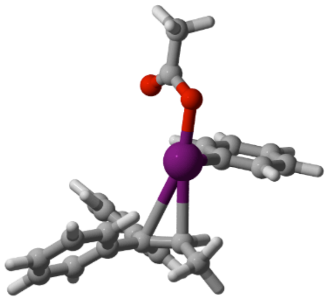

Xyz-Matrix

```
48
XYZ file generated by gabedit : coordinates in Angstrom
C          1.7885550000         0.6331130000         0.6860960000
C          1.0100470000         0.3941680000         1.8282650000
C          2.9486640000        -0.2035500000         0.3186670000
C          1.4278550000        -0.3767140000         3.0657550000
I         -0.7098460000        -1.1712900000         0.3921280000
C          4.0754450000         0.3952360000        -0.2960600000
C          5.2009690000        -0.3658160000        -0.6227410000
C          5.2167430000        -1.7459430000        -0.3732680000
C          4.1018130000        -2.3596930000         0.2197500000
C          2.9882540000        -1.5961510000         0.5742260000
C         -2.1388840000         0.3580380000         0.6152680000
C         -2.3660440000         1.2210040000        -0.4650870000
C         -3.3349280000         2.2216580000        -0.3062470000
C         -4.0433890000         2.3422530000         0.8982240000
C         -3.7948990000         1.4602970000         1.9618840000
C         -2.8322950000         0.4506580000         1.8316620000
O         -2.1151310000        -2.5352790000        -0.4036050000
O         -1.9926640000        -1.3834000000        -2.3536130000
C         -2.4303690000        -2.3173120000        -1.7077120000
C         -3.3962820000        -3.3700680000        -2.2157110000
H          2.4251250000        -0.8122180000         2.9742230000
H          1.4403930000         0.3223430000         3.9131380000
H          0.7267600000        -1.1776730000         3.3339010000
H          6.0654870000         0.1169160000        -1.0744960000
H          6.0893800000        -2.3401980000        -0.6389400000
H          4.1047220000        -3.4317940000         0.4074370000
H          2.1403250000        -2.0988110000         1.0360280000
H         -1.8306920000         1.1063410000        -1.4031910000
```

H	-3.5362300000	2.9004980000	-1.1326710000
H	-4.7961670000	3.1209550000	1.0078310000
H	-4.3528470000	1.5486340000	2.8921400000
H	-3.5430460000	-3.2284870000	-3.2888550000
H	-3.0100330000	-4.3735220000	-2.0088470000
H	-4.3537060000	-3.2665950000	-1.6915760000
H	0.2117340000	1.1168890000	1.9957400000
H	4.0791120000	1.4651190000	-0.4890590000
C	1.4419200000	1.7807030000	-0.1850760000
C	1.5160140000	1.6644500000	-1.5930040000
C	1.1695610000	2.7404550000	-2.4144090000
C	0.7685880000	3.9606430000	-1.8465920000
C	0.7043340000	4.0941250000	-0.4513960000
C	1.0268210000	3.0115780000	0.3729570000
H	1.8236450000	0.7228900000	-2.0433750000
H	1.2170250000	2.6311240000	-3.4963630000
H	0.5180360000	4.8043530000	-2.4877110000
H	0.4152640000	5.0446310000	-0.0067060000
H	1.0069850000	3.1396210000	1.4538400000
H	-2.6480090000	-0.2420380000	2.6497390000

Thermodynamic Data

```
Zero-point  correction=                                        0.384755
(Hartree/Particle)
 Thermal correction to Energy=                      0.411024
 Thermal correction to Enthalpy=                    0.411968
 Thermal correction to Gibbs Free Energy=           0.323175
 Sum of electronic and zero-point Energies=            -1050.941989
 Sum of electronic and thermal Energies=               -1050.915720
 Sum of electronic and thermal Enthalpies=             -1050.914776
 Sum of electronic and thermal Free Energies=          -1051.003570

                     E (Thermal)             CV                 S
                      KCal/Mol        Cal/Mol-Kelvin    Cal/Mol-Kelvin
 Total                  257.922             96.000            186.882
```

4.0 NBO analysis

4.1 Charge Distribution

A1–parent (B3LYP)

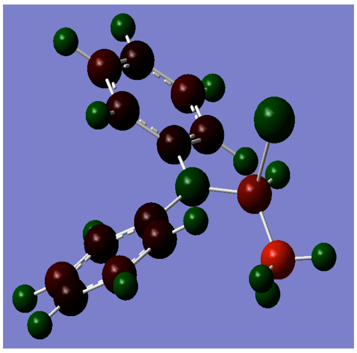

Charge (NBO) distribution
From □1 (light red) to +1 (light [illegible]

(B3LYP)

A1-π-complex iodine (B3LYP)

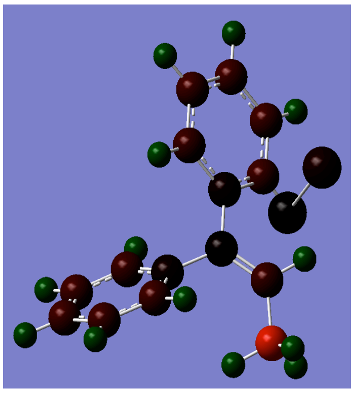

Charge (NBO) distribution
From □1 (light red) to +1 (light green)

A1

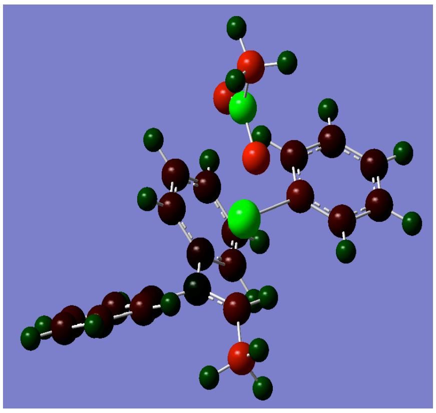

Charge (NBO) distribution
From □1 (light red) to +1 (light [illegible]

A1–parent (M06-2X)

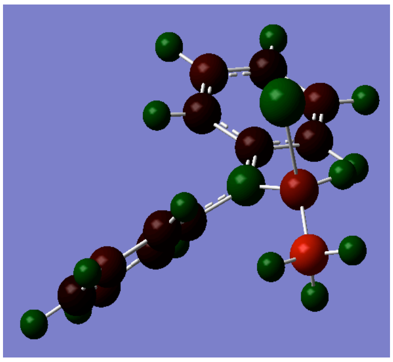

Charge (NBO) distribution
From □1 (light red) to +1 (light green)

A1-π-complex iodine (M06-2X)

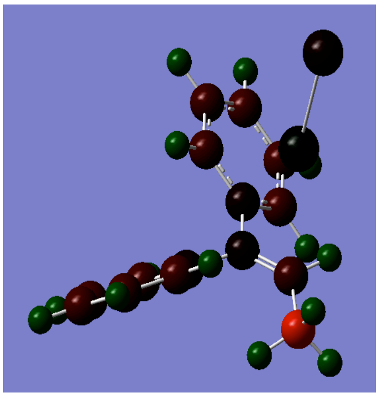

Charge (NBO) distribution
From □1 (light red) to +1 (light green)

A1 (M06-2X)

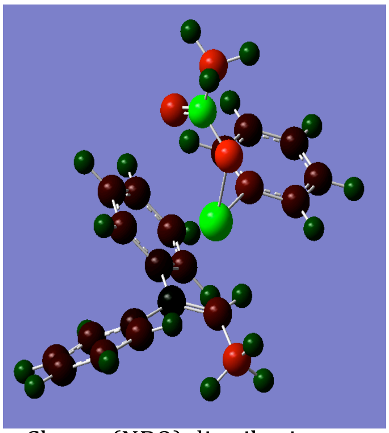

Charge (NBO) distribution
From □1 (light red) to +1 (light green)

Orbital Interactions

Bonding in the iodoniumion **A1** in comparison to the π-complex and **A1-parent**

M06-2X

A1: C1-C2 π-bond donates into σ*-orbital of I-O bond with E2 = 34.1 kcal/mol, also entailing a lower bond order of C1-C2 π-bond ⇔ occupancy of 1.70 a.u.. Partial charge of alkene part = + 0.24 a.u. and of the I($OCOCH_3$)Ph-part = +0.76 a.u.

A1-parent: No electron density between C1 and I => stabilization of the carbeniumion by donation of electron density of the iodine lone pair LP3 (> 95% p-character; E2-energy = 7.42 kcal/mol) and by drawing on electron density from adjacent C2-I-bond (E2-energy = 27.11 kcal/mol)

A1-π-complex: C1-C2 π-bond donates into σ*- orbital of I-I bond with E2 = 6.0 kcal/mol, also entailing a lower bond order of C1-C2 π-bond ⇔ occupancy of 1.86 a.u.. Partial charge of alkene part = + 0.04 a.u. and of I_2-part –0.04 a.u..

B3LYP

A1: C1-C2 π-bond donates into σ*- orbital of I-O bond with E2 = 27.43 kcal/mol also entailing a lower bond order of C1-C2 π-bond ⇔ occupancy of 1.65 a.u.. Partial charge of alkene part = + 0.34 a.u. and of I($OCOCH_3$)Ph-part +0.66 a.u..

A1-parent: No electron density between C1 and I => stabilization of the carbeniumion by donation of electron density of the lone pair LP3 of I-atom (> 95% p-character; E2-energy = 4.40 kcal/mol) and by drawing on electron density from adjacent C2-I-bond (E2-energy = 19.20 kcal/mol)

A1-π-complex: C1-C2 π-bond donates into σ*- orbital of I-I bond with E2 = 6.4 kcal/mol also entailing a lower bond order of C1-C2 π-bond ⇔ occupancy of 1.82 a.u.. Partial charge of alkene part = + 0.1 a.u. and of I_2-part –0.1 a.u..

References

[1] M. Uyanik, T. Yasui, K. Ishihara, *Angew. Chem.* **2010**, *122*, 2221–2223; *Angew. Chem. Int. Ed.* **2010**, *49*, 2175–2177.

[2] M. Fujita, Y. Yoshida, K. Miyata, A. Wakisaka, T. Sugimura, *Angew. Chem.* **2010**, *122*, 7222–7225; *Angew. Chem. Int. Ed.* **2010**, *49*, 7068–7071.

[3] M. Fujita, S. Okuno, H. J. Lee, T. Sugimura, T. Okuyama, *Tetrahedron Lett.* **2007**, *48*, 8691–8694.

[4] X. Zhao, J. Jing, K. Lu, Y. Zhang, J. Wang, *Chem. Commun.* **2010**, *46*, 1724–1726.

[5] P. Beak, P. D. Becker, *J. Org. Chem.* **1982**, *47*, 3855–3861.

[6] P. M. Gannett, D. L. Nagel, P. J. Reilly, T. Lawson, J. Sharpe, B. Toth, *J. Org. Chem.* **1988**, *53*, 1064–1071.

[7] A. J. A. Van der Weerdt, H. Cerfontain, *Tetrahedron* **1981**, *37*, 2121–2130.

[8] F. Gualtieri, E. Teodori, C. Bellucci, E. Pesce, G. Piacenza, *J. Med. Chem.* **1985**, *28*, 1621–1628.

[9] Z.-X. Wang, Y. Shi, *J. Org. Chem.* **1998**, *63*, 3099–3104.

[10] A. van der Bent, A. G. S. Blommaert, C. T. M. Melman, A. P. IJzerman, I. van Wijngaarden, W. Soudijn, *J. Med. Chem.* **1992**, *35*, 1042–1049.

[11] M. Rueping, M. Leiendecker, A. Das, T. Poisson, L. Bui, *Chem. Commun.* **2011**, *47*, 10629–10631.

[12] M. Ochiai, T. Ito, H. Takahashi, A. Nakanishi, M. Toyonari, T. Sueda, S. Goto, M. Shiro, *J. Am. Chem. Soc.* **1996**, *118*, 7716–7730.

[13] A. P. Uijttewaal, F. L. Jonkers, A. Van der Gen, *J. Org. Chem.* **1979**, *44*, 3157–3168.

[14] C. M. Latham, A. J. Blake, W. Lewis, M. Lawrence, S. Woodward, *Eur. J. Org. Chem.* **2012**, 699–707.

[15] G. Zhu, W. Kong, H. Feng, Z. Qian, *J. Org. Chem.* **2014**, *79*, 1786–1795.

[16] H. J. Lim, C. R. Smith, T. V. RajanBabu, *J. Org. Chem.* **2009**, *74*, 4565–4572.

[17] U. Scheffler, R. Mahrwald, *Helv. Chim. Acta* **2012**, *95*, 1970–1975.

[18] C. Ebner, A. Pfaltz, *Tetrahedron* **2011**, *67*, 10287–10290.

[19] C. Pan, F. Luo, W. Wang, Z. Ye, J. Cheng, *Tetrahedron Lett.* **2009**, *50*, 5044–5046.

[20] Y. Yamamoto, N. Kirai, Y. Harada, *Chem. Commun.* **2008**, 2010–2012.

[21] M. Al-Masum, Y. Yamamoto, *J. Am. Chem. Soc.* **1998**, *120*, 3809–3810.

[22] J. Y. L. Chung, D. Mancheno, P. G. Dormer, N. Variankaval, R. G. Ball, N. N. Tsou, *Org. Lett.* **2008**, *10*, 3037–3040.

[23] P. M. Lundin, J. Esquivias, G. C. Fu, *Angew. Chem.* **2009**, *121*, 160–162; *Angew. Chem. Int. Ed.* **2009**, *48*, 154–156.

[24] S. Fukuda, K. Tsuji, J. Musashi, R. Nonaka, T. Kimura, T. Satoh, *Synthesis* **2011**, 3615–3626.

[25] S. Lou, G. C. Fu, *J. Am. Chem. Soc.* **2010**, *132*, 1264–1266.

[26] W. Krasodomski, M. K. Łuczyński, J. Wilamowski, J. J. Sepioł, *Tetrahedron* **2003**, *59*, 5677–5683.

[27] Y. T. Hong, A. Barchuk, M. J. Krische, *Angew. Chem.* **2006**, *118*, 7039–7042; *Angew. Chem. Int. Ed.* **2006**, *45*, 6885–6888.

[28] P. A. Evans, S. Oliver, J. Chae, *J. Am. Chem. Soc.* **2012**, *134*, 19314–19317.

[29] Y. Nakamura, S. Takeuchi, Y. Ohgo, M. Yamaoka, A. Yoshida, K. Mikami, *Tetrahedron* **1999**, *55*, 4595–4620.

[30] Gaussian 09, Revision D.01 M. J. Frisch, G. W. Trucks, H. B. Schlegel, G. E. Scuseria, M. A. Robb, J. R. Cheeseman, G. Scalmani, V. Barone, B. Mennucci, G. A. Petersson, H. Nakatsuji, M. Caricato, X. Li, H. P. Hratchian, A. F. Izmaylov, J. Bloino, G. Zheng, J. L. Sonnenberg, M. Hada, M. Ehara, K. Toyota, R. Fukuda, J. Hasegawa, M. Ishida, T. Nakajima, Y. Honda, O. Kitao, H. Nakai, T. Vreven, J. A. Montgomery, Jr., J. E. Peralta, F. Ogliaro, M. Bearpark, J. J. Heyd, E. Brothers, K. N. Kudin, V. N. Staroverov, R. Kobayashi, J. Normand, K. Raghavachari, A. Rendell, J. C. Burant, S. S. Iyengar, J. Tomasi, M. Cossi, N. Rega, J. M. Millam, M. Klene, J. E. Knox, J. B. Cross, V. Bakken, C. Adamo, J. Jaramillo, R. Gomperts, R. E. Stratmann, O. Yazyev, A. J. Austin, R. Cammi, C. Pomelli, J. W. Ochterski, R. L. Martin, K. Morokuma, V. G. Zakrzewski, G. A. Voth, P. Salvador, J. J. Dannenberg, S. Dapprich, A. D. Daniels, O. Farkas, J. B. Foresman, J. V. Ortiz, J. Cioslowski, and D. J. Fox, Gaussian, Inc., Wallingford CT, 2009.

[31] J. W. McIver, A. Komornicki, *J. Am. Chem. Soc.* **1972**, *94*, 2625–2633.

[32] Y. Zhao, D. G. Truhlar, *Theor. Chem. Acc.* **2008**, *120*, 215–241.

[33] V. G. Zakrzewski, J. V. Ortiz, J. A. Nichols, D. Heryadi, D. L. Yeager, J. T. Golab, *Int. J. Quant. Chem.* **1996**, *60*, 29–36.

[34] a) C. T. Lee, W. T. Yang, R. G. Parr, *Phys. Rev. B* **1988**, *37*, 785–789; b) A. D. Becke, *J. Chem. Phys.* **1993**, *98*, 5648–5652; c) P. J. Stephens, F. J. Devlin, C. F. Chabalowski, M. J. Frisch, *J. Phys. Chem.* **1994**, *98*, 11623–11627; d) A. D. Becke, *Phys. Rev. A* **1988**, *38*, 3098–3100.

[35] NBO Version 3.1, E. D. Glendening, A. E. Reed, J. E. Carpenter, F. Weinhold as implemented in Gaussian09.D01.

[36] A.-R. Allouche, *J. Comput. Chem.* **2011**, *32*, 174–182.
